# Concise synthesis of simplified aogacillin analogs reveals innate reactivity and synergy with aminoglycosides

**DOI:** 10.1039/d6sc04046b

**Published:** 2026-06-19

**Authors:** Andrew R. LeBlanc, Jacqueline R. Smith, Audrey J. Isakov, John Bacsa, William M. Wuest

**Affiliations:** a Department of Chemistry, Emory University Atlanta GA 30322 USA wwuest@emory.edu

## Abstract

Aogacillins (AOGs) A–B, two diastereomeric natural products isolated from *Simplicillium* sp. FKI-5985, were reported to have potent synergism with aminoglycoside antibiotics. Herein, we show that structural simplification expedites the synthesis of AOG analogs in 3–4 steps. Furthermore, we demonstrate that these compounds irreversibly trap cysteine nucleophiles at an electrophilic enone warhead but are also susceptible to [4 + 2] cycloadditions. Therefore, strategically designed AOG analogs were constructed to mitigate side reactivity and sensitize MRSA to aminoglycosides at sub-MIC concentrations.

## Abstract 2

Aogacillins (AOGs) A–B, two diastereomeric natural products isolated from *Simplicillium* sp. FKI-5985, are shown to have innate reactivity in biological systems and possess potent synergism with aminoglycoside antibiotics.

## Graphical abstract

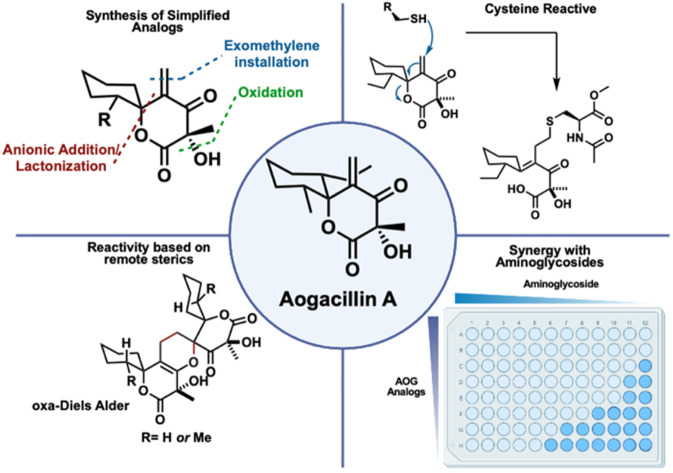

## Introduction

Natural products (NPs) are uniquely privileged in the realm of antibiotics, with over 65% of FDA approved antibiotics being NPs or NP-derived.^1^ Historically, NPs such as vancomycin, linezolid, and gentamicin have acted as a frontline defense for Gram-positive bacteria, including methicillin-resistant *Staphylococcus aureus* (MRSA); however, continued resistance to these therapeutics is pressing. The United States Center for Disease Control (CDC) classifies MRSA as a serious threat with over 80 000 severe infections in the United States each year.^2–4^ Taken together, there is a significant urgency for the community to develop new approaches to tackle antimicrobial resistance (AMR).

There are two main avenues through which NPs can combat AMR. They can possess novel mechanisms of action (MOAs) not used in the clinic, thereby limiting the likelihood of being predisposed, as bacteria may not have evolved mechanisms against them. For example, lefamulin is a pleuromutilin-derived NP that targets the bacterial peptidyl transferase center on the 50S subunit of the ribosome and was first approved by the FDA in 2019.^5,6^ Its novel MOA and short time in the clinic have resulted in low levels of reported resistance.^7^ Alternatively, NPs can sensitize bacteria to previously approved antibiotics by targeting the resistance mechanism itself.^8^ This approach was the foundation for the FDA-approved combination therapy Avycaz™, composed of ceftazidime/avibactam. Ceftazidime is a third-generation β-lactam that was FDA approved in 1985 but shortly became ineffective due to the overexpression of β-lactamase enzymes.^9^ Avibactam, a β-lactamase inhibitor, targets the enzyme responsible for ceftazidime resistance; therefore, when used in combination, ceftazidime/avibactam regains its efficacy.

**Fig. 1:**
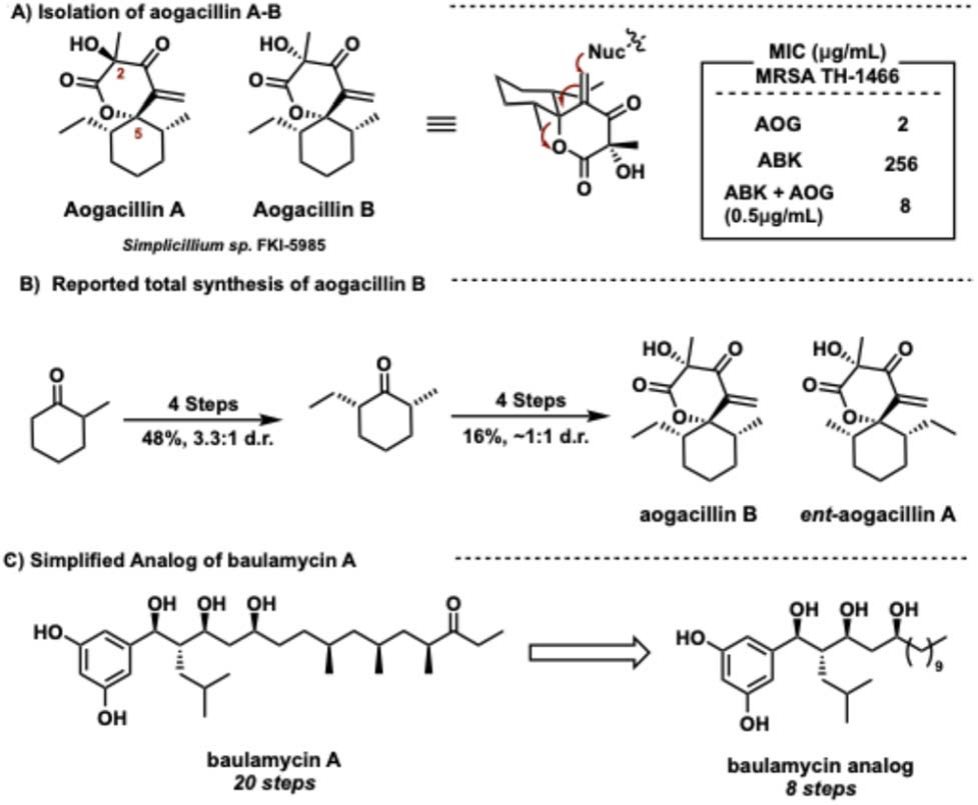
(A) Aogacillins A–B and their reported biological activity. (B) Zhang's total synthesis of AOG B and *ent*-AOG A. (C) Simplified analog of baulamycin A.

Aided by thousands of years of evolutionary pressure, antimicrobial NPs exhibit diverse biological activity. Aogacillins (AOGs) A–B, isolated in 2013 from *Simplicillium* sp. FKI 5985, are diastereomeric NPs only differing in the relative stereochemistry of the tertiary alcohol (Fig. 1a).^10^ Furthermore, AOG A–B were reported to have a minimum inhibitory concentration (MIC) of 2 µg mL^−1^ against MRSA. Interestingly, AOG A–B were also found to sensitize aminoglycoside-resistant MRSA to arbekacin (ABK) at sub-MIC concentrations, lowering the MIC of ABK from 256 to 8 µg mL^−1^.^10^ The biological activity, coupled with its unique chemical structure, has made AOG a desirable synthetic target. In 2026, Zhang, Tong, and co-workers published the first total synthesis of aogacillin B and *ent*-aogacillin A in 8 steps and 5% overall yield.^11^ Their route highlights a strategic aldol and lactonization sequence to both install the tertiary alcohol and close the spirocycle in one step (Fig. 1b).

We became interested in AOG for its biological activity and hypothesized that a simplified analog would be both viable and useful. We realized that the exocyclic enone is likely the molecular warhead, capable of accepting biological nucleophiles and contributing to its MOA. Unlike common electrophilic warheads, AOG possesses a β-leaving group (C5 carboxylate) that could be expelled after Michael addition, leading to an irreversible covalent adduct (Fig. 1a).

As such, we proposed that the exocyclic enone/spirocyclic motif was the main pharmacophore responsible for its biological activity and set out on a campaign to design analogs around this scaffold.

Examining AOG A–B from a retrosynthetic perspective, we identified that the main synthetic hurdle was not the spirocycle/enone motif, but the unique methyl/ethyl substitution pattern. In the case of Zhang's total synthesis, four steps were used to install the ethyl group.^11^ As such, we hypothesized that deletion of the methyl and/or ethyl group could drastically decrease the synthetic burden while still accessing the same chemical space and maintaining biological activity.

Our group and others have had a successful track record of leveraging NP simplification.^12–17^ One specific example relates to the anti-MRSA NP baulamycin A, whose total synthesis took 20 total synthetic steps, whereas the simplified analogs only took 8 (Fig. 1c). Furthermore, the simplified analogs displayed a decreased MIC against *S. aureus*.^18^ With this approach in mind, we set out to synthesize and biologically evaluate simplified AOG analogs.

**Fig. 2:**
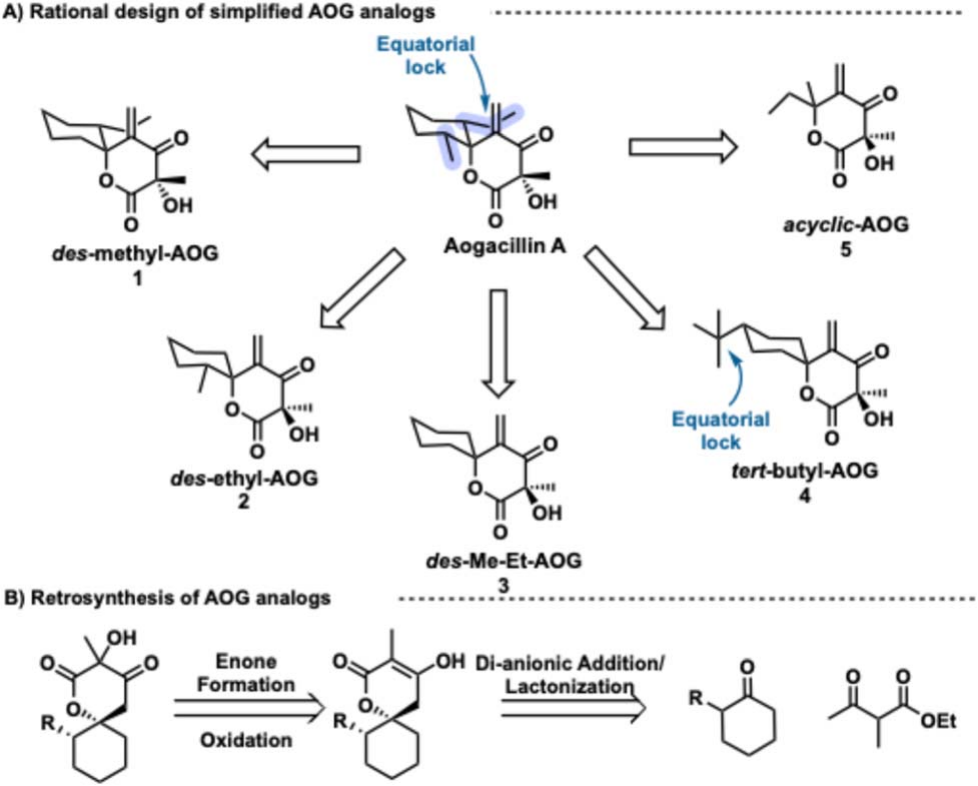
(A) Rational design of simplified AOG analogs. (B) Retrosynthetic approach for AOG analogs.

Initially, we imagined that deletion of the methyl and/or ethyl groups would provide three analogous compounds: *des*-methyl-AOG 1, *des*-ethyl-AOG 2, and *des*-methyl-ethyl-AOG 3 (Fig. 2a). Conformationally, however, the methyl/ethyl groups are both equatorial, locking the resulting chair conformation, which we speculated is critical for the biological activity. Therefore, many of our designs sought to preserve this key structural feature. For example, installing a remote *tert*-butyl group would similarly lock the chair conformation 4. Lastly, we envisioned removing the spirocyclic nature of the scaffold altogether to access an acyclic analog, 5. Retrosynthetically, access to the core scaffold could be forged *via* a di-anionic addition-lactonization sequence to efficiently install the spirocycle, an alternative approach in comparison to Zhang.^11^ From this advanced intermediate, subsequent oxidation of the enone to the tertiary alcohol and methylene installation would provide rapid access to the simplified AOG scaffold (Fig. 2b).

## Results and discussion

**Fig. 3:**
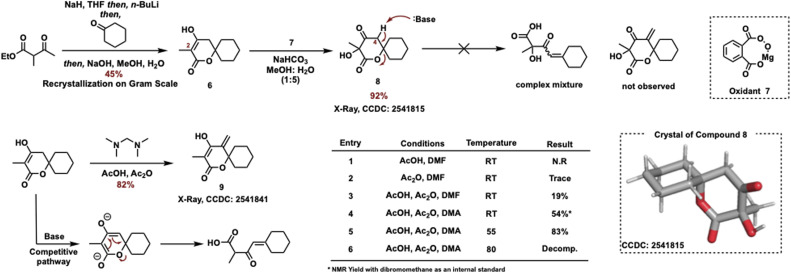
Synthetic route towards *des*-Me-Et-AOG and exocyclic alkene optimization.

We initially targeted *des*-methyl-ethyl 3 and began with the di-anionic addition of ethyl-2-methylacetoacetate to cyclohexanone to afford the desired 1,2-addition. Subsequent treatment with NaOH resulted in lactonization to form 5 in 45% yield over the sequence (Fig. 3). To access the first AOG analog, we needed to (1) oxidize C2 to the tertiary alcohol and (2) install the C4 exocyclic enone. With those transformations in mind, we screened a variety of oxidants (SI Tables 1–3) and found that magnesium bis(monoperoxyphthalate) with NaHCO_3_ in aqueous methanol cleanly provided 8 (92%; confirmed by X-ray crystallography, CCDC: 2541815). Omission of base or increased methanol concentrations was deleterious to the reaction (SI Fig. 1). Installation of the exocyclic alkene also proved challenging. Utilization of various formaldehyde surrogates under several conditions proved unsuccessful. Instead, we observed irreversible elimination of the lactone, as deprotonation at C4 enables rapid E1cB elimination, which outcompetes the desired condensation.

**Fig. 4:**
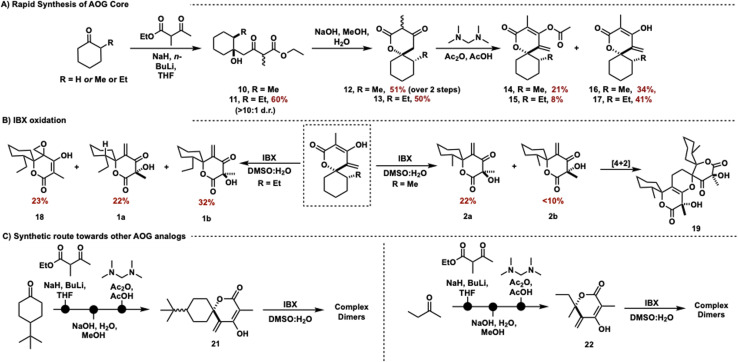
(A) Synthesis towards the core of *des*-Me-AOG, *des*-Et-AOG, and *des*-Et-Me-AOG. (B) IBX oxidation to access *des*-Me-AOG and *des*-Et-AOG. (C) Synthesis towards the core of *tert*-butyl-AOG and acyclic-AOG.

To overcome this, we first installed the enone and then oxidized the core scaffold. Again, this unique spirocyclic core at the β-position made alkene installation difficult as competing elimination reopened the spirocycle. However, after multiple rounds of optimization (SI Tables 4–6), we found that treatment of 6 with *N*,*N*,*N*,*N*′-tetramethyldiaminomethane, acetic acid and acetic anhydride in DMA resulted in formation of the exocyclic enone (Fig. 3, Optimization Table). With the enone installed we set out to perform the last oxidation. Surprisingly, initial attempts at oxidation were unsuccessful and either resulted in recovered starting material or a complex mixture of dimers (SI Table 7). Concurrently, we were also investigating the synthesis of compounds 1 and 2, which underwent the same di-anionic addition followed by subsequent lactonization to form the spirocyclic core. We were able to confirm the relative stereochemistry of 12*via* X-ray crystallography (CCDC: 2543179). Treatment of both 12 and 13 under the optimized enone formation conditions resulted in formation of 16 and 17, respectively (Fig. 4a). We also found side formation of the acetyl-protected enones, 14 and 15. These results demonstrate the robust nature of this method which could lend itself to other applications for the installation of exocyclic enones with β-leaving groups.

Toward the completion of simplified AOG analogs, treatment of 17 with IBX provided conversion to both diastereomers of *des*-methyl-AOG (1a, 22%; 1b, 32%). We also isolated 18 as a minor side product resulting from epoxidation across the exocyclic alkene. These same conditions work well with 16, providing both diastereomers of *des*-ethyl-AOG in 40% and <10%. Interestingly, we attribute the poor yield of 2b to an alternate reactivity profile, where it undergoes an oxa-Diels–Alder cycloaddition to form 19.

We attribute this selectivity between 2a and 2b to the *syn*-relationship between the methyl and α-methyl groups in 2b, whereby this relationship blocks the front face of the compound leaving the back face sterically accessible for the cycloaddition (Fig. 4b). Alternatively, 2a possesses an anti*-*relationship between the methyl and α-methyl group, each blocking differing faces of the enone, preventing the [4 + 2] cycloaddition and providing the scaffold with a privileged reactivity profile. Furthermore, the relative regiochemistry of this dimer was unexpected and counterintuitive, with the enone reacting at the α-position rather than the expected, and polarity matched, β-position. We found that this regiochemistry is reported in other NP dimerizations. (SI Fig. 3).^19–21^ Attempts to take this unique side product and undergo a retro-[4 + 2] cycloaddition were explored but resulted in decomposition. We also saw evidence for dimerization with compounds 21 and 22; however, the lack of substitution resulted in a non-diastereoselective [4 + 2] cycloaddition and a complex mixture of diastereotopic dimers (SI Fig. 4) (Fig. 4c). Of note, we found that no AOG dimer has ever been reported or commented on. Taken together, we hypothesize that the remote methyl and ethyl substituents in the NP likely provide steric interference, thereby decreasing its propensity to dimerize.

### Steric analogs

**Fig. 5:**
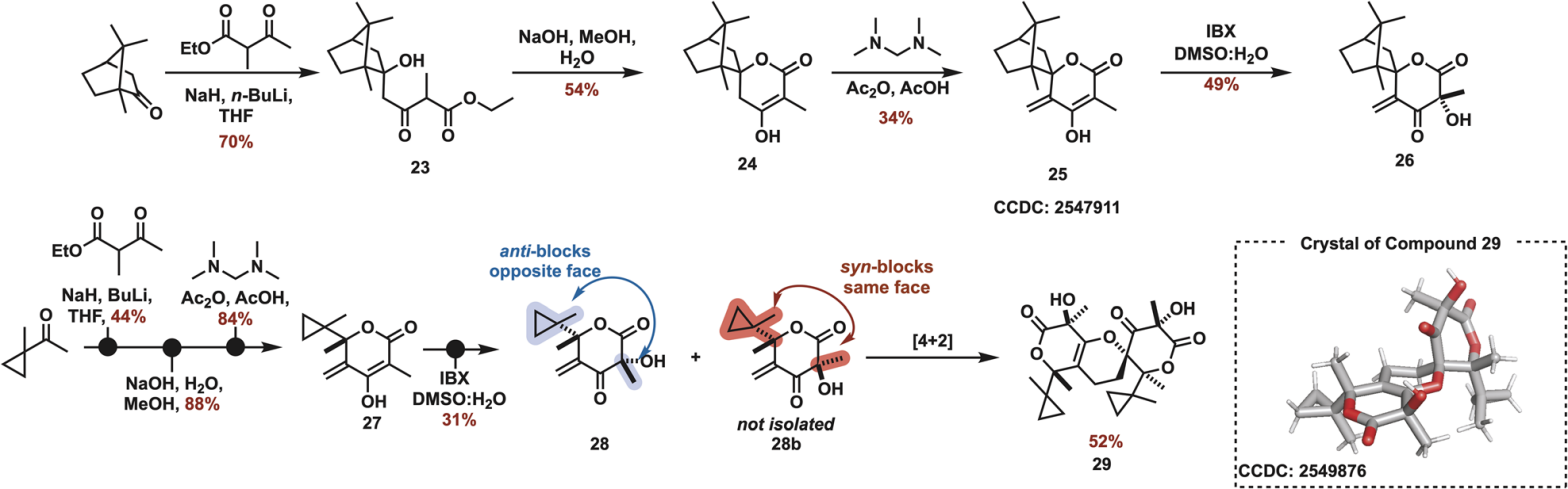
Synthesis of sterically hindered AOG analogs to prevent dimerization.

Due to the prevalence of oxa-Diels–Alder dimeric natural products, we rationalized that the propensity for dimerization was not only a chemical liability but may also dimerize under biological conditions. As such, we aimed to synthesize new AOG inspired analogs with increased steric demand to prevent dimerization. We first selected a camphor derivative due to its rigid and sterically demanding core. Furthermore, the natural concavity of camphor would direct the first di-anionic addition to the convex face and result in the exocyclic alkene being buried in the convex face of the scaffold. Taking camphor through the di-anionic addition, lactonization, and alkene installation, we accessed 25 in 4 steps (confirmed *via* X-ray, CCDC: 2547911). Treatment of 25 with IBX proceeded smoothly to give the oxidized derivative in 49% yield (5.7 : 1 d.r). Importantly, we found no evidence of dimerization, further highlighting the role of remote sterics (Fig. 5).

Next, we designed a cyclopropyl analog, 27, that would juxtapose 22. We rationalized that the increase in sterics might be enough to prevent the dimerization. To this end, we synthesized a cyclopropyl analog, 27, in three steps. Excitingly, we found that 27 readily undergoes IBX oxidation; however, one diastereomer (28b) dimerizes to 29 (confirmed by X-ray, CCDC: 2549876). Comparable to 2a–b, we attribute this selectivity to the *syn*-relationship between the cyclopropyl and methyl group, whereby the back face is accessible for the cycloaddition.

### Covalent reactivity

**Fig. 6:**
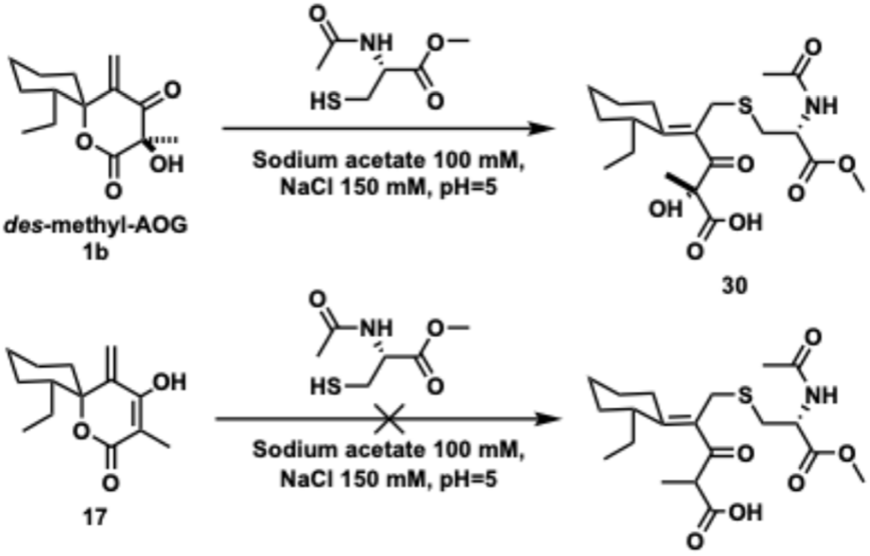
Covalent reactivity of *des*-methyl-AOG and 17.

With a series of AOG analogs in hand, we wanted to test our initial hypothesis that the enone and spirocycle acted as an irreversible covalent trap. To investigate the chemoselectivity of AOG analogs, compound 1b was mixed with an equimolar ratio of Boc-Lys-OMe, Boc-Ser-OMe, and Ac-Cys-Ome, in pH 7.4 PBS buffer. After 10 min, the reaction was analyzed directly by LCMS (SI Fig. 5). A mass adduct matching that of a covalent reaction with cysteine was the sole product identified. To determine the structure of the cysteine-bound adduct, the reaction was scaled up and probed over a range of pH values. At pH 5, the cleanest conversion was seen, and the irreversibly bound adduct was isolated (Fig. 6). In contrast, we found that treating 17 with Ac-Cys-OMe resulted in no reaction. We hypothesize that the lack of oxidation at the α-position allows for extended conjugation throughout the π-system of the exocyclic alkene, thus decreasing the electrophilicity of the enone itself. Qualitatively, examination of ^1^H-NMR shows that the vinyl protons of 17 are drastically shielded in comparison to those on 1b. The requirement for oxidation at the α-position to permit covalent reactivity, coupled with the fact that the ethyl/methyl aid in preventing dimerization, suggests that this unique functionality may be a result of evolutionary refinement of the scaffold.

### Biological activity

**Fig. 7:**
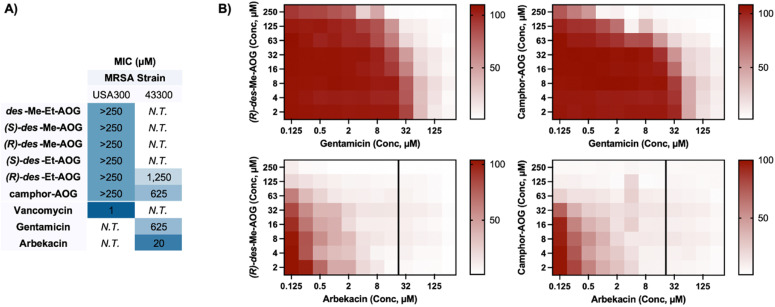
(A) MICs of AOG analogs and antibiotics against MRSA USA3-0114 and MRSA ATCC43300. N.T. = not tested. (B) Synergy heatmaps, % growth of MRSA ATCC43300 when treated with varying concentrations of AOG analogs and aminoglycoside. All reported data are the average of 3 biological replicates.

With synthetic analogs in hand, we set out to test the biological activity of these natural product-inspired compounds. We found that our synthetic derivatives showed poor activity against methicillin-resistant *S. aureus* (MRSA) on their own, with all MIC values exceeding 250 µM, in contrast to the reported data (Fig. 7a). When analogs were treated in conjunction with aminoglycoside antibiotics against a resistant strain (ATCC43300), the analogs were found to restore aminoglycoside activity. Checkerboard assays reveal (*R*)-*des*-methyl-AOG (1b) to have synergy with arbekacin, which aligns with literature reports of the natural product activity.^10^ The AOG analogs lowered doses from 20 µM to 2 µM (Fig. 7b, (*R*)-*des*-methyl-AOG 63 µM, arbekacin 2 µM, FIC = 0.150; camphor-AOG 63 µM, arbekacin 2 µM, FIC = 0.201). Excitingly, synergy was also found between 1b (125 µM) and gentamicin, a widely used aminoglycoside, granting a gentamicin MIC of 63 µM (FIC = 0.201) when this strain was previously resistant (MIC = 625 µM). Similarly, camphor-AOG (26 at 125 µM) is synergistic with gentamicin, lowering the MIC from 625 to 32 µM (FIC = 0.251). These findings expand the synergistic scope of these simplified analogs through checkerboard synergy with aminoglycosides, while the activity of natural or fully synthetic aogacillin was not directly evaluated. Future work will investigate the mechanism of this synergistic effect.

## Conclusion

**Fig. 8:**
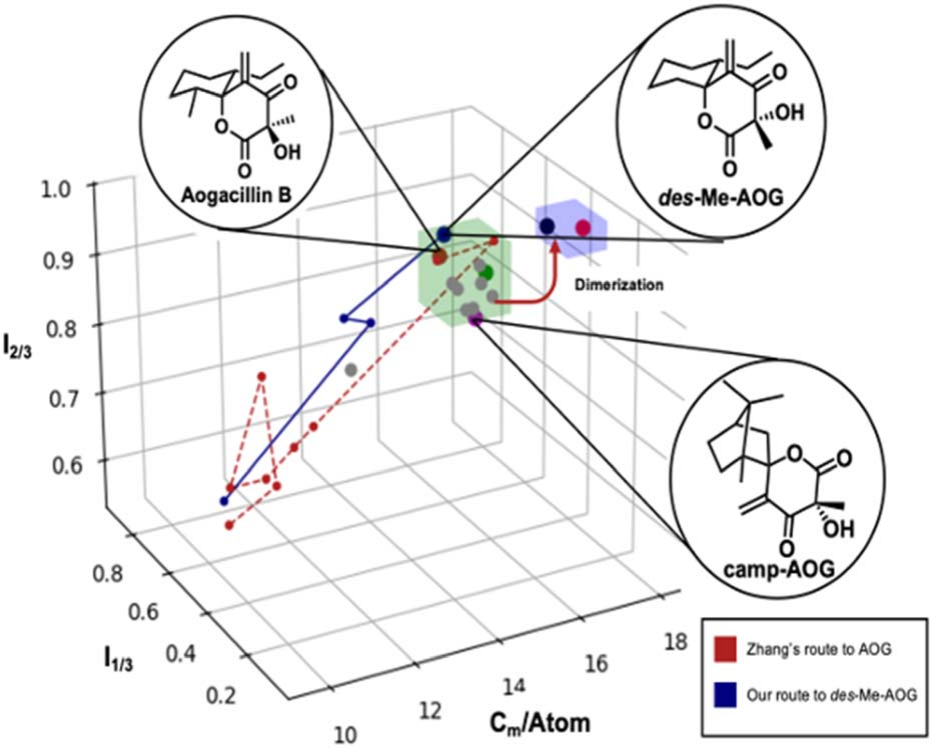
The chemical space of AOG and AOG analogs, overlayed with Zhang's route to AOG B and our route to *des*-methyl-AOG.

At the outset of the project, we aimed to access a similar chemical space to AOG while decreasing the synthetic burden to access these compounds. Visually representing this idea of chemical space has been pioneered by Shenvi.^22,23^ To exemplify our approach, we examined the structures of AOG, *des*-methyl-AOG, *des-*ethyl-AOG, and camphor-AOG and calculated their principal moment of inertia (*I*_1_/*I*_3_ and *I*_2_/*I*_3_) and the Bottcher complexity per atom (*C*_m_/atom).^22,24^ As expected, when graphed on a 3D plot, the AOG analogs occupy a similar region in chemical space (Fig. 8).^23,25^ We then plotted all synthetic compounds and intermediates (black line) of our approach alongside those for Zhang's total synthesis (red dotted line). Both our starting material and that of Zhang's synthesis originate in a similar area, thereby demonstrating how we can access the same region of chemical space in half the number of synthetic steps. That being said, our di-anionic route was unable to access AOG due to its inability to lactonize following the 1,2-addition. (SI Fig. 6). This exemplifies how slight changes to the targeted chemical space can unlock alternative synthetic routes. Finally, we show that dimerization allows access to yet another region of chemical space that we initially did not anticipate.

In conclusion, we have synthesized a series of AOG analogs that access the same chemical space as the corresponding NP in 3–4 synthetic steps. Throughout our synthetic approach, we observed key structural features of this scaffold that impart privileged reactivity. For example, the methyl/ethyl substituents also appear to temper the side-reactivity of the off-pathway [4 + 2] dimerization of the enone itself. We also demonstrated how the unique spirocyclic enone motif reacts with cysteine and expels the carboxylate only when the α-oxidation is present. Taken together, this work highlights the intricacies of the seemingly simple AOG scaffold and uncovers unique principles clearly harnessed by nature. These reactive structural features and key structural changes drastically reduce the synthetic complexity, enabling a concise synthesis of compounds that confer synergistic interactions with clinically relevant aminoglycoside antibiotics against resistant MRSA strains.

## Author contributions

The manuscript was written through contributions of all authors. All authors have given approval to the final version of the manuscript.

## Conflicts of interest

There are no conflicts to declare.

## Supplementary Material

SC-017-D6SC04046B-s001

SC-017-D6SC04046B-s002

## Data Availability

CCDC 2541815 (8), 2541841 (9), 2543179 (12), 2547911 (25) and 2549876 (29) contain the supplementary crystallographic data for this paper.^26*a*–*e*^ The data underlying this study are available in the published article and its supplementary information (SI). Supplementary information: experimental procedures, characterization data, and NMR spectra. See DOI: https://doi.org/10.1039/d6sc04046b.
