# Supplementary material for: Concise synthesis of simplified aogacillin analogs reveals innate reactivity and synergy with aminoglycosides
Source: Chem Sci. 2026 Jun 19;17(31):15080–6. doi: 10.1039/d6sc04046b (PMC13312816; doi:10.1039/d6sc04046b)
Supplement: Supplementary file 1 [file SC-017-D6SC04046B-s001.pdf]

Supplementary Information (SI) for Chemical Science.
This journal is © The Royal Society of Chemistry 2026

Supporting Information

Concise Synthesis of Simplified Aogacillin Analogs Reveals Innate Reactivity and Synergy with Aminoglycosides

Andrew R. LeBlanc, Jacqueline R. Smith, Audrey J. Isakov, John Bacsa, William M. Wuest*

Department of Chemistry, Emory University, 1515 Dickey Dr, Atlanta, GA 30322, United States

Table of Contents

A. Instrumentation and General Notes **2**

B. Optimization **3**

C. Synthetic Procedures **14**

D. Biological Procedures **32**

E. Chemical Space **33**

F. NMR Spectra **40**

SI Figure 1: 96-well plate screen for cleanest oxidation screen. 5
SI Figure 2: 1H analysis highlighting the shielding effect of the C2 hydroxyl group. 9
SI Figure 3: Similar 4+2 oxa-Diels alder dimers reported. 10
SI Figure 4: Highlighting the complex mixture of [4+2] dimers when there is no facial selectivity. 11
SI Figure 5: Amino acid competition to determine AOG selectivity 12
SI Figure 6: Attempts towards AOG, highlighting how the bis-substation prevents lactonization likely through a Thorpe-Ingold Effect. 13

SI Table 1: Oxidation Screen of **6.** 3
SI Table 2: Oxidation screen of TIPS protected **6**. 4
SI Table 3: Solvent and additive screen for magnesium bis(monoperoxyphthalate) screen. (monitored by LCMS) 4
SI Table 4: Initial screen for formation of 9. 6
SI Table 5: Additive screen for the formation of 9. 6
SI Table 6: Temperature screen for the formation of 9. 7
SI Table 7: Attempted oxidations of 9 8

A. Instrumentation and General Notes

Non-aqueous reactions were carried out under an atmosphere of argon in flame-dried glassware. Tetrahydrofuran (THF), dichloromethane (DCM), diethyl ether (Et_2O), toluene, and N,N- dimethylformamide (DMF) were dried by passage through alumina. All other commercial reagents and anhydrous solvents were used as received (from Sigma Aldrich, Oakwood Chemical, Combi Blocks, Alfa Aesar, Fisher Scientific, TCI America, or AK Scientific) without further purification.. Reactions were monitored by analytical thin layer chromatography (TLC) using EMD Millipore silica gel 60 F254 precoated plates and visualized using UV and/or ninhydrin, vanillin, or KMnO4 stains. Flash chromatography employing Siliaflash silica gel (40-63 µm) was performed on a Biotage Isolera One instrument with a linear normal-phase gradient.

Nuclear magnetic resonance (^1H, ^{13}C NMR) spectra were recorded using the following spectrometers: Bruker Avance NEO800 (800 MHz), Varian INOVA600 (600 MHz), Bruker Ascend (600 MHz), Varian INOVA500 (500 MHz), Varian INOVA400 (400 MHz), Bruker Avance NEO400 (400 MHz), and Bruker Avance III (400 MHz). Chemical shifts are reported in parts per million (ppm) relative to tetramethylsilane and referenced to the residual solvent signal. Signal patterns are indicated as follows: s (singlet), d (doublet), t (triplet), q (quartet), m (multiplet), br (broad signal). High-resolution mass spectra were measured on a Thermo LTQ-FTMS using either APCI or ESI techniques.

B. Optimization

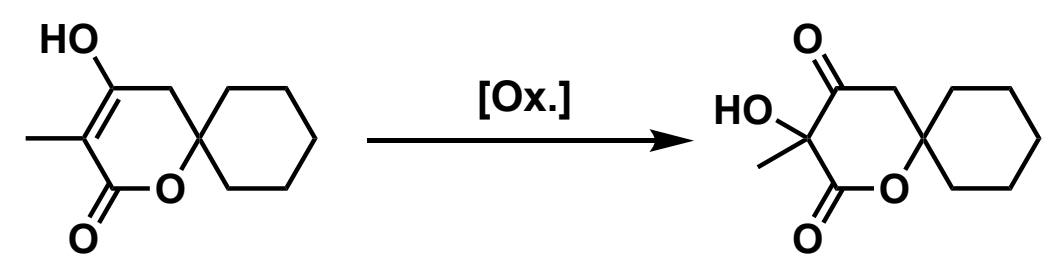

Entry	Oxidant	Solvent	Additive	*Result*
1	O_2	DMSO	$P(OEt)_3$	N.R.
2	Davis Ox.	THF	DBU	N.R.
3	Davis Ox.	THF	NaH	N.R.
4	Davis Ox.	THF	K_2CO_3	N.R.
5	Davis Ox.	THF	LiHMDS	N.R.
6	*m*CPBA	Chloroform	AcOH, RT	N.R.
7	*m*CPBA	Toluene	AcOH, RT	N.R.

Entry	Oxidant	Solvent	Additive	*Result*
8	*m*CPBA	Chloroform	AcOH, 60°C	N.R.
9	*m*CPBA	Toluene	AcOH, 60°C	N.R.
10	*m*CPBA	Chloroform	RT	Trace
11	*m*CPBA	Toluene	RT	N.R.
12	*m*CPBA	Chloroform	60 ^{o}C	N.R.
13	*m*CPBA	Toluene	60 ^{o}C	N.R.

SI Table 1: Oxidation Screen of **6.**

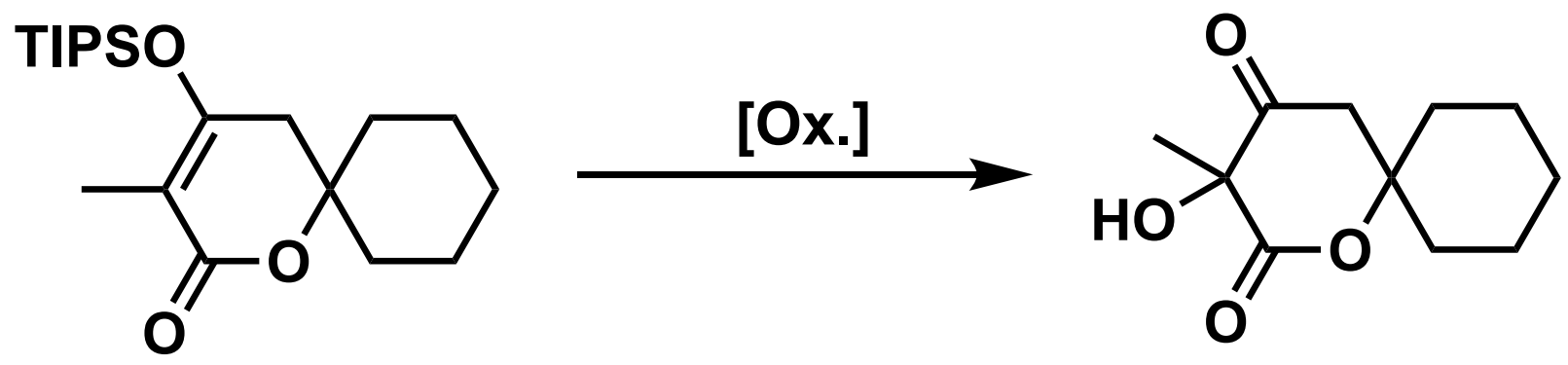

Entry	Oxidant	Solvent	Additive	*Result*
1	*m*CPBA	DCM	N/A	N.R.
2	*m*CPBA	DCM	AcOH	N.R.
3	*m*CPBA	DCM	K_2CO_3	N.R.
4	Davis Ox.	DCM	N/A	N.R.
5	Davis Ox.	DCM	AcOH	N.R.
6	Davis Ox.	DCM	K_2CO_3	N.R.

SI Table 2: Oxidation screen of TIPS protected **6**.

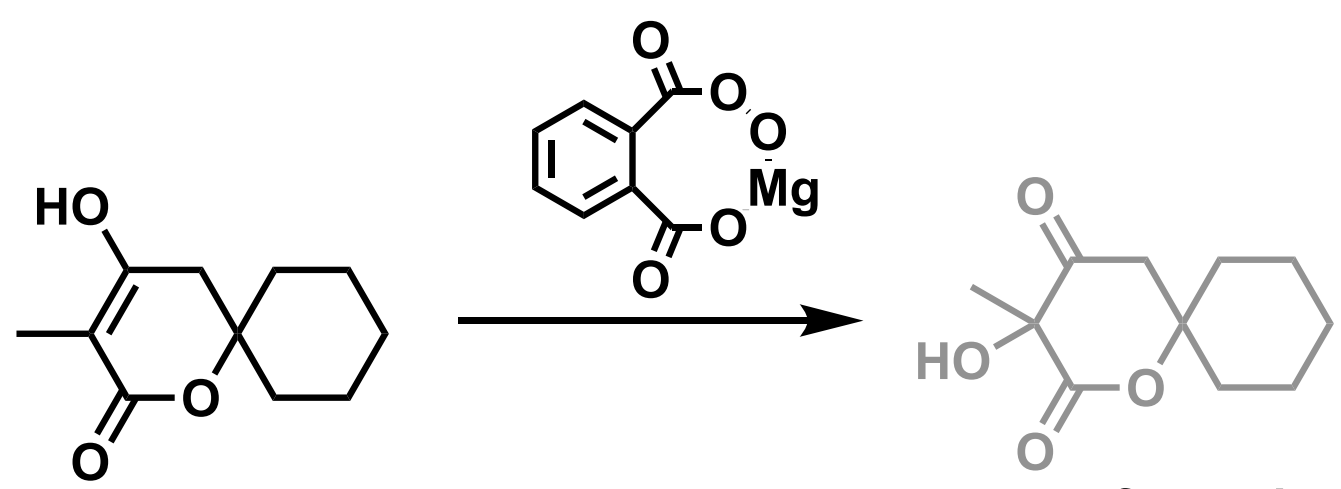

Entry	Solvent	Additive	*Result*
1	MeOH	N/A	Me + [O] (227 m/z)
2	MeOH	Cs_2CO_3	Me + [O] (227 m/z)
3	MeOH	$NaHCO_3$	Me + [O] (227 m/z)
4	EtOH	N/A	Complex
5	EtOH	Cs_2CO_3	Complex
6	EtOH	$NaHCO_3$	Et + [O] (241 m/z)

SI Table 3: Solvent and additive screen for magnesium bis(monoperoxyphthalate) screen. (monitored by LCMS).

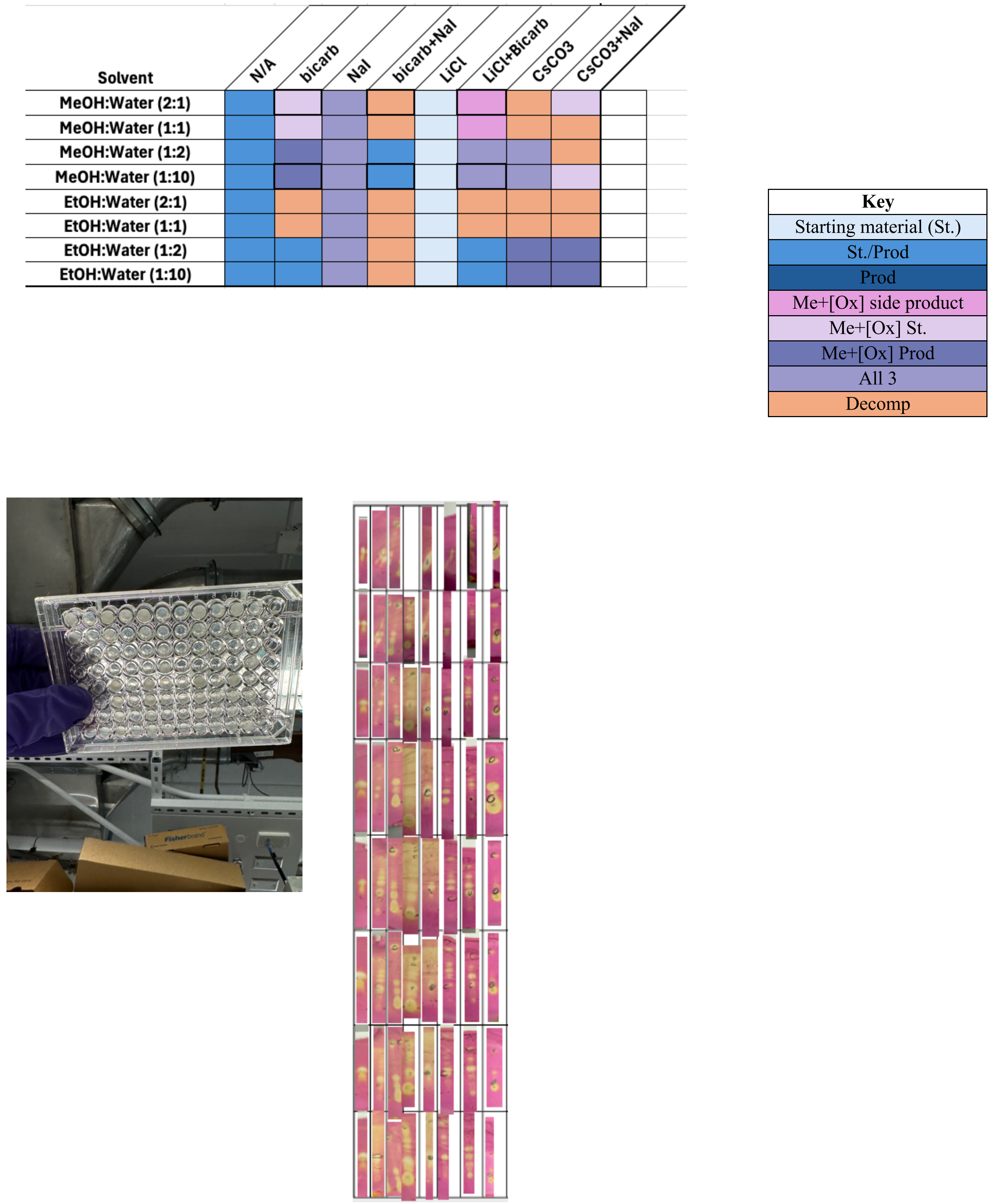

SI Figure 1: 96-well plate screen for cleanest oxidation screen. TLC show reaction profile of each reaction conditions to rapidly screen for cleanest reaction profile.

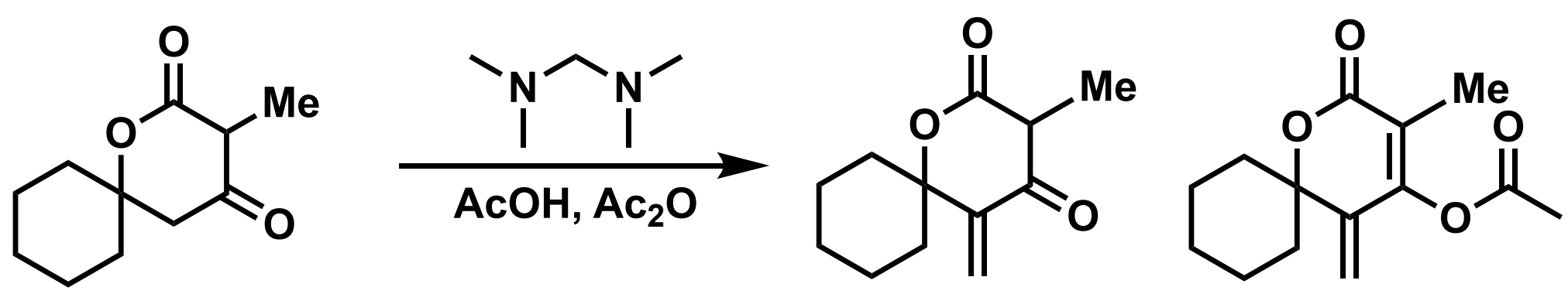

Entry	Additive	Solvent	Temperature	Result
1	AcOH	DMF	RT	N.R.
2	Ac_2O	DMF	RT	Mass hit***
3	AcOH, Ac_2O	DMF	RT	Mass hit- 19% isolated**
4	AcOH, Ac_2O	$CHCl_3$	RT	Decomp.
5	AcOH, Ac_2O	ACN	RT	Decomp.
6	AcOH, Ac_2O	Dioxane	RT	70% (LCMS Conversion)
7	AcOH, Ac_2O	DMA	RT	80% (LCMS), 54 NMR Yield

*** Reproducibility Challenge

SI Table 4: Initial screen for formation of 9.

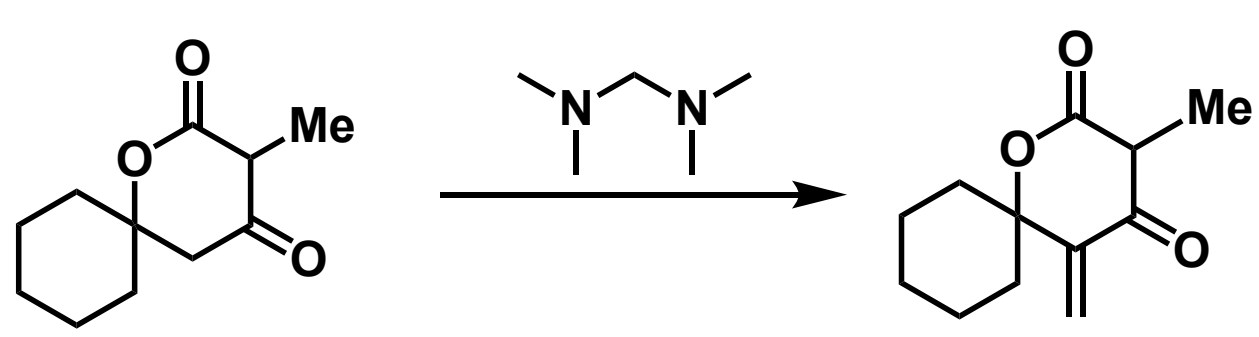

	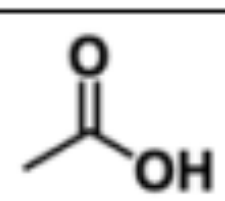	H--Cl	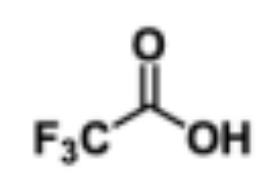	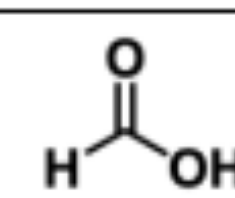
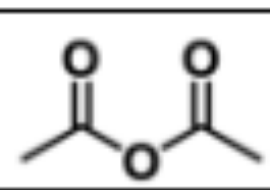	90	14	56	38
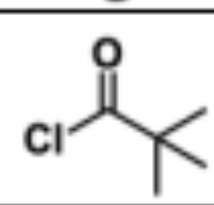	13	3	7	6
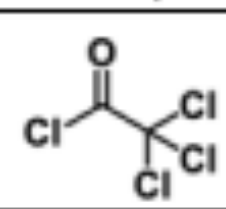	0	0	45	25
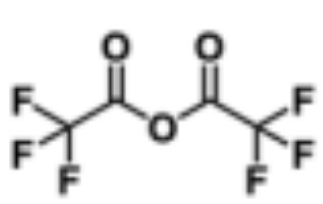	61	37	86	2

SI Table 5: Additive screen for the formation of 9.

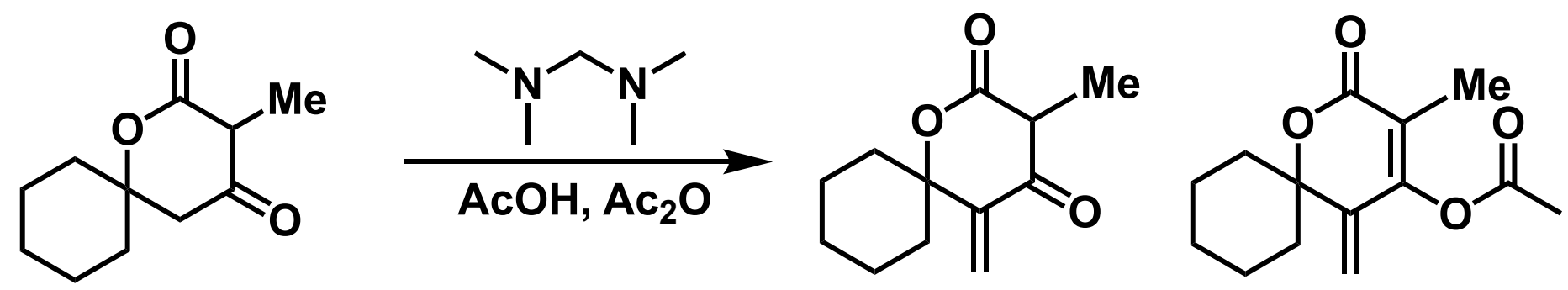

Entry	Additive	Solvent	Temperature	Result
1	AcOH, Ac_2O	DMA	RT	42% A, 15%B —LCMS
2	AcOH, Ac_2O	DMA	55	83% A, Trace B —isolation
3	AcOH, Ac_2O	DMA	65	52% A, 48% B —LCMS
4	AcOH, Ac_2O	DMA	80	Decomp.

SI Table 6: Temperature screen for the formation of 9.

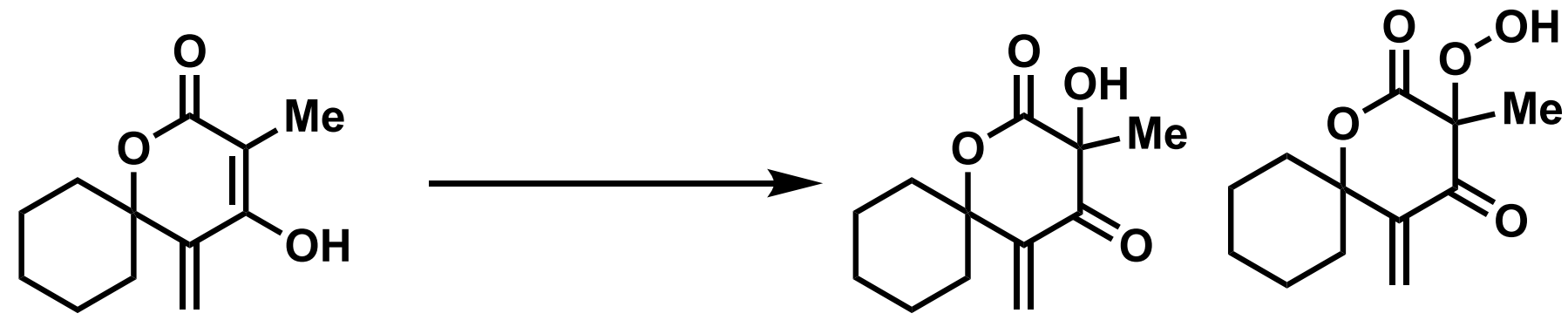

Entry	Oxidant	Additive	Solvent	Result
1	mCPBA	---	DCM	NR
2	mCPBA	bicarb	DCM	NR
3	mCPBA	AcOH	DCM	NR
4	1	bicarb	H_2O:MeOH	NR
5	IBX	---	—	Dimer
6	O_2	$CeCl_3$	H_2O:MeOH	decomp.
7	TBHP	$Mg(CF_3SO_3)_2$, EDTA	DCM	NR
8	Oxone	—	EtOAc:H_2O	NR
9	Urea H_2O_2	TFA	ACN	decomp.
10	O_2	DBU	ACN	N.R.
11	O_2	$Mn(OAc)_2$	DCM	decomp.
12	O_2	$CoCl_2$	IPA	complex dimers
13	O_2	NaOAc, I_2	THF	405 mass +ST
14	O_2	Rose-Bengal, UV	MeOH	432 mass clean
15	O_2	$Cu(NO_3)_2$	ACN	Trace hit?

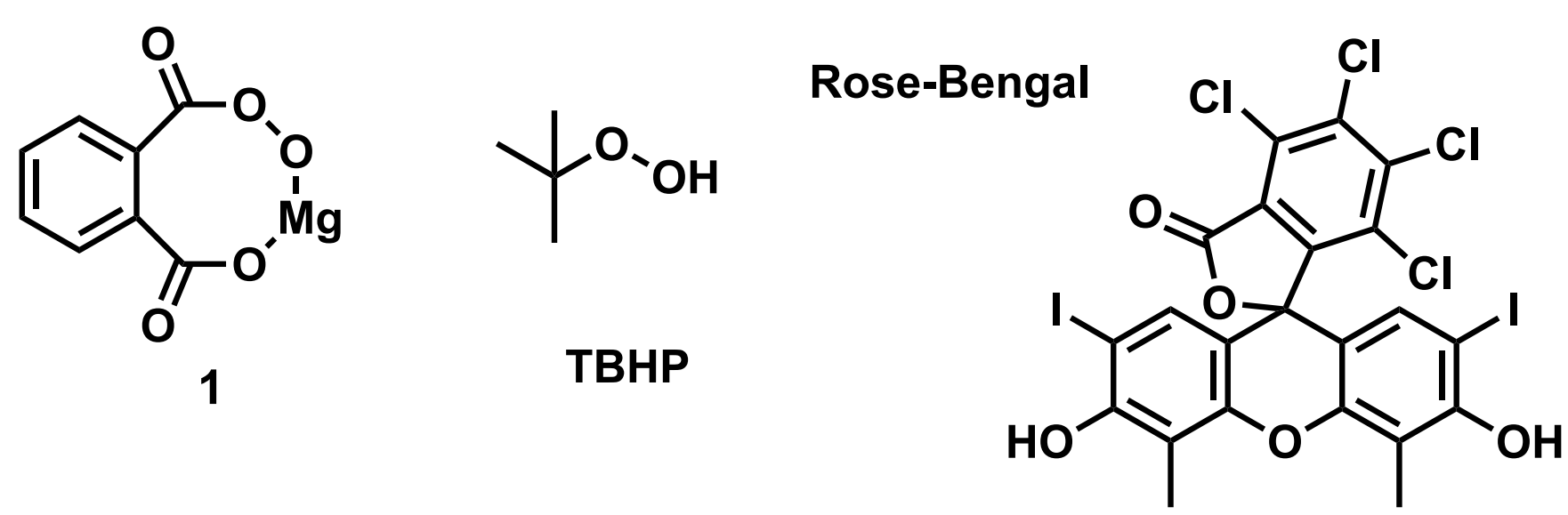

SI Table 7: Attempted oxidations of 9

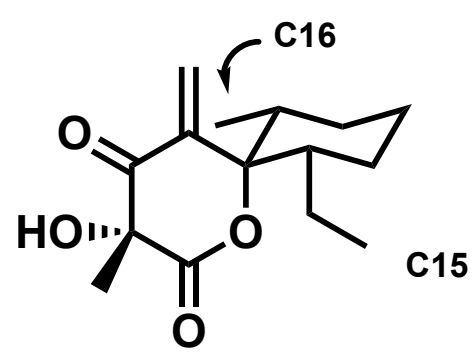

AOG B

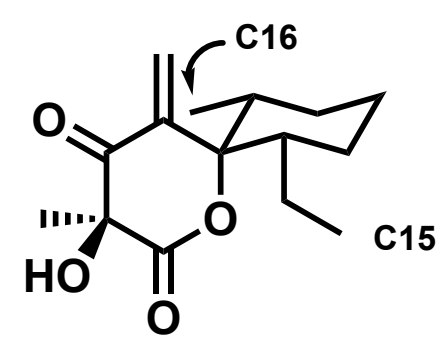

AOG A

Proton Shift (PPM)	AOG A	AOG B
C15	0.94	0.82
C16	0.71	0.98

Isolation paper[1]

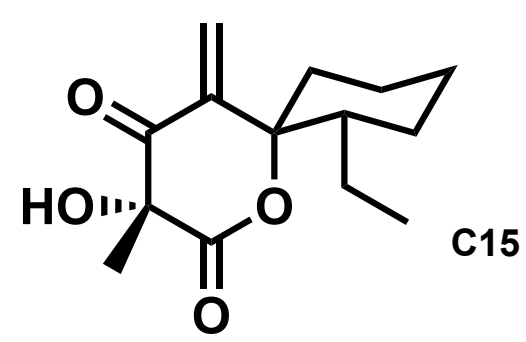

1a

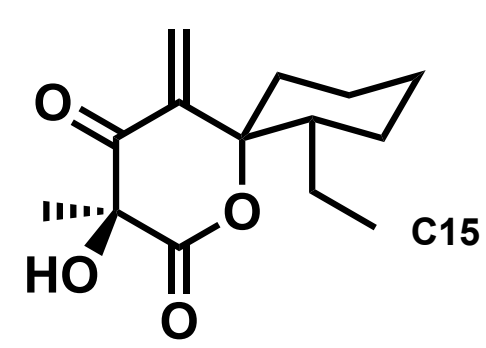

1b

Proton Shift (PPM)	1a	1b
C15	0.94	0.79

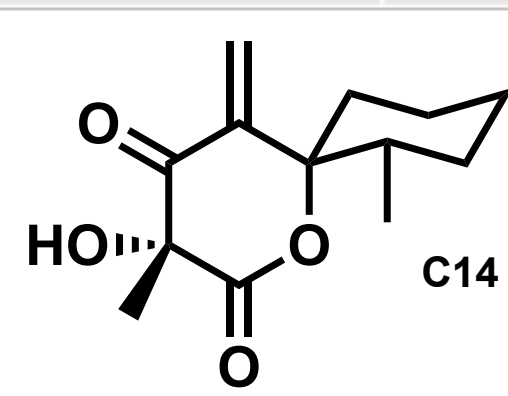

2b

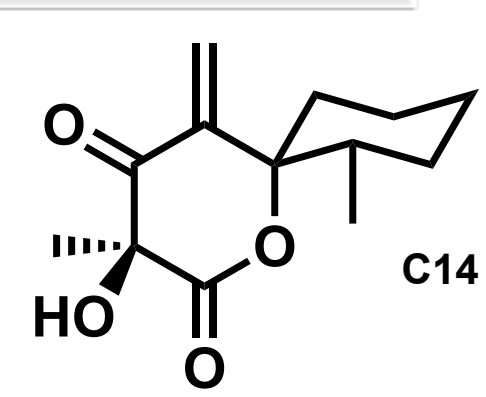

2a

Proton Shift (PPM)	2b	2a
C14	1.01	0.73

SI Figure 2: 1H analysis highlighting the shielding effect of the C2 hydroxyl group.

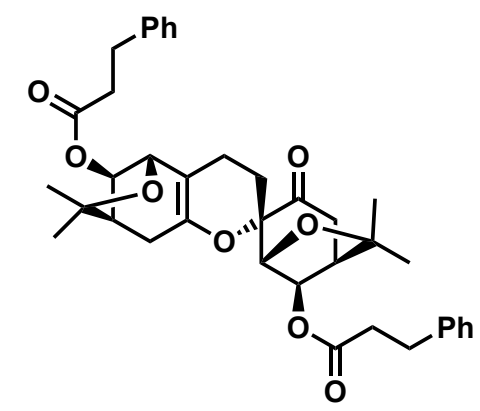

no name reported
RSC Adv., 2017, 7, 38956

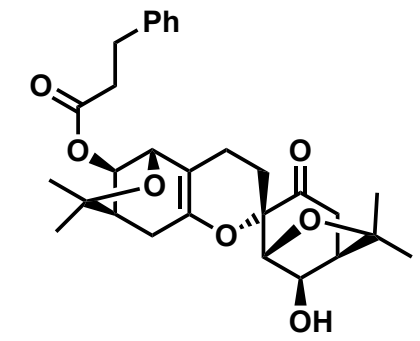

no name reported
RSC Adv., 2017, 7, 38956

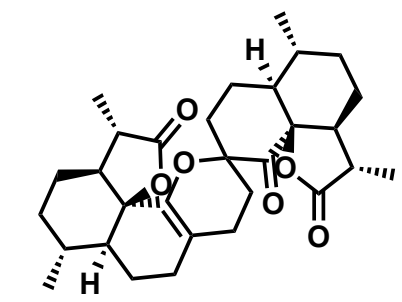

artemordin G
Chin. J. Chem., 42: 1493-1508.

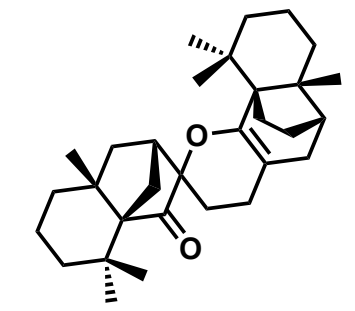

Chiastaucolin A
Phytochemistry 179 (2020) 112495

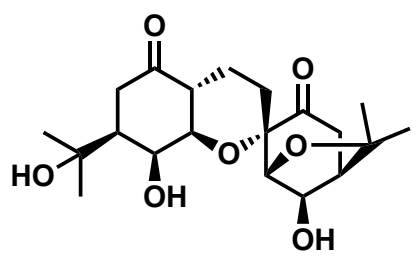

no name reported
RSC Adv., 2017, 7, 38956

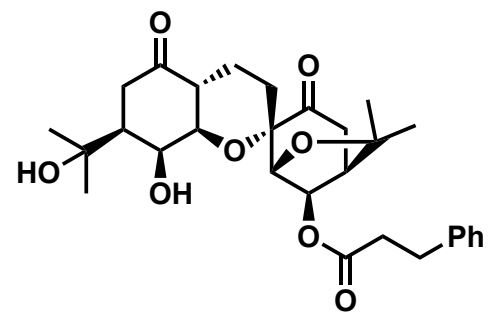

no name reported
RSC Adv., 2017, 7, 38956

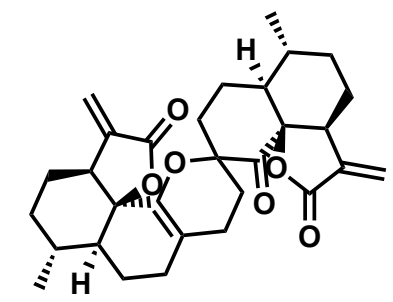

artemordin H
Chin. J. Chem., 42: 1493-1508.

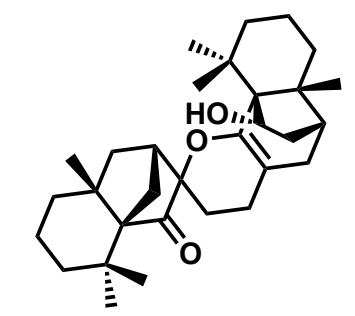

Chiastaucolin B
Phytochemistry 179 (2020) 112495

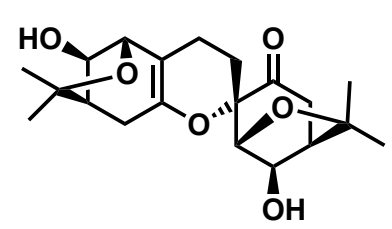

dimericilligerate E
Bioorganic Chemistry 86 (2019) 674–678

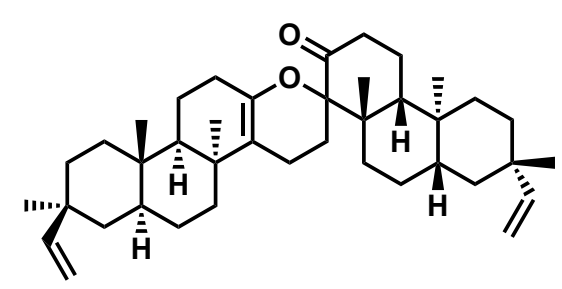

Tagalsins I
Org. Lett. 2005, 7, 14, 3037–3040

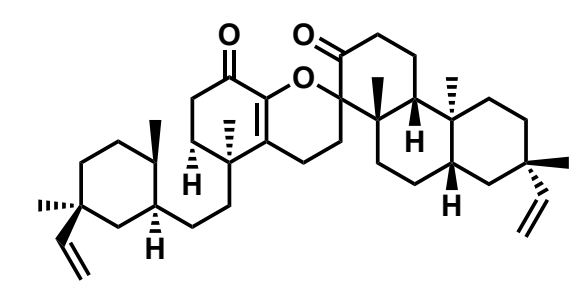

Tagalsins J
Org. Lett. 2005, 7, 14, 3037–3040

SI Figure 3: Similar 4+2 oxa-Diels alder dimers reported.

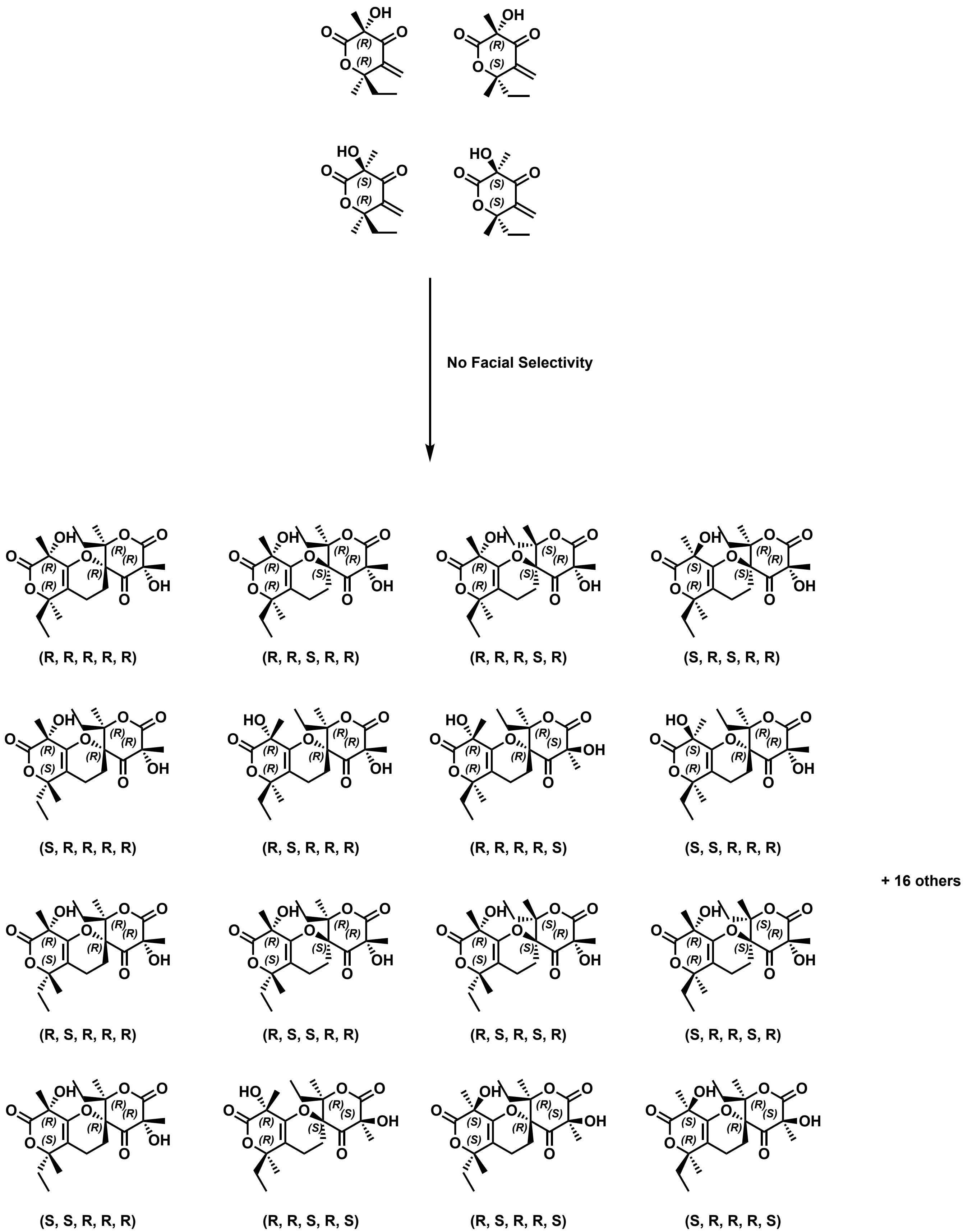

SI Figure 4: Highlighting the complex mixture of [4+2] dimers when there is no facial selectivity.

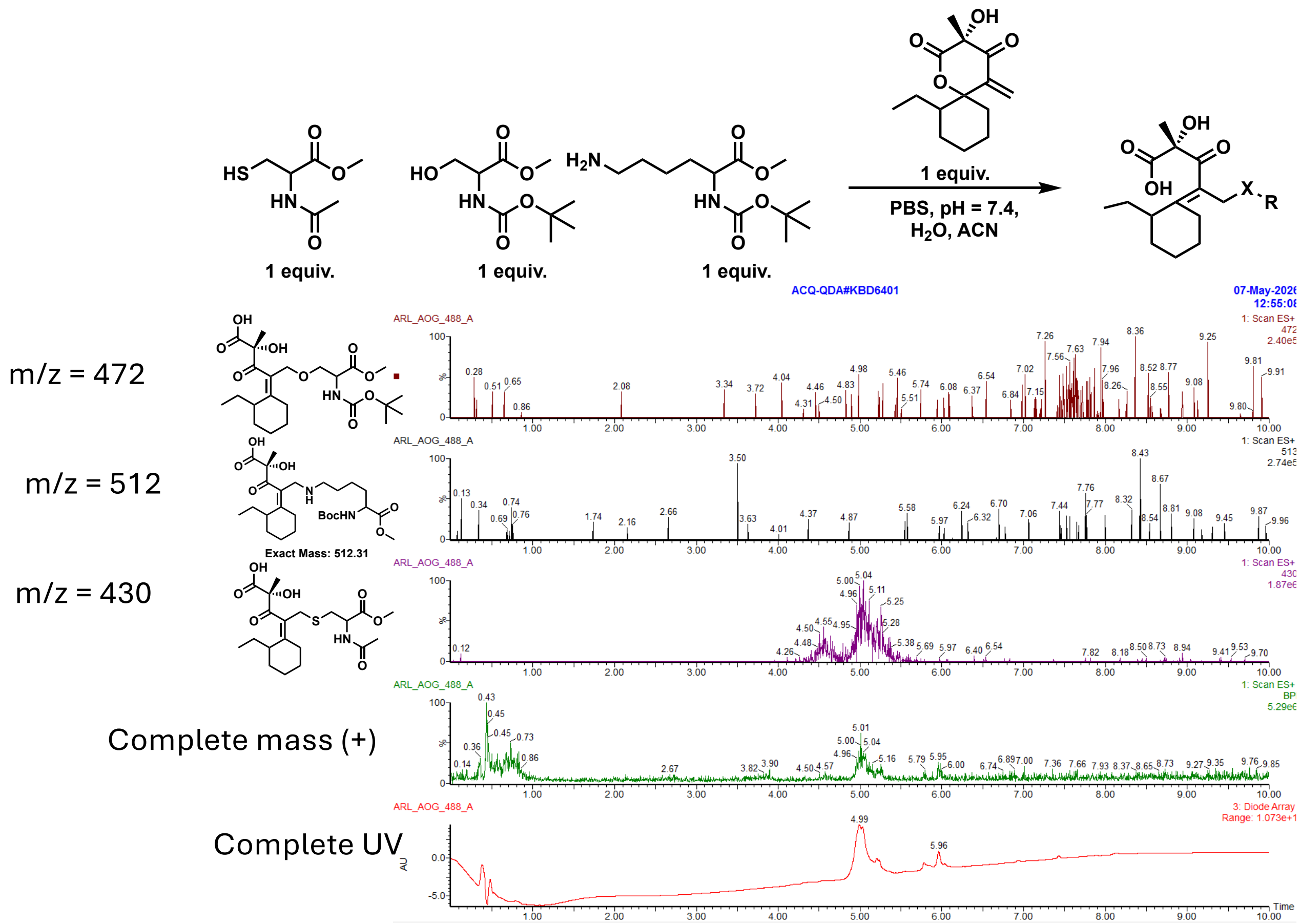

SI Figure 5: Amino acid competition to determine AOG selectivity.

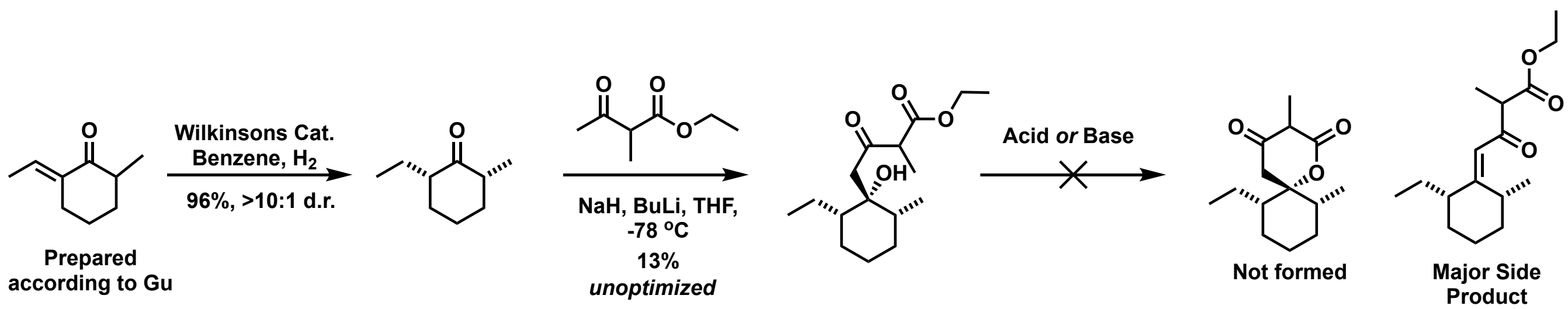

SI Figure 6: Attempts towards AOG, highlighting how the bis-substation prevents lactonization likely through a Thorpe-Ingold Effect.

C. Synthetic Procedures

General Procedure A:

To a flame dried flask anhydrous THF (0.2 M) was added and cooled to 0 °C. 1 equivalent of ethyl 2-methyl-3-oxobutanoate was added followed by 1.2 equivalents of NaH (60% wt). The reaction stirred for 15 minutes before the slow addition of 1.2 equivalents of BuLi (2.2M). The reaction stirred for 30 minutes before the addition of 1.1 equivalents of ketone. The reaction then ran for 3 hours and was quenched with 1M HCl. The reaction was extracted with diethyl ether, dried with $MgSO_4$, filtered, and concentrated under reduced pressure. Depending on the substrate (details below), the crude material was either carried forward to the next procedure (General procedure B) or purified via column chromatography.

General Procedure B:

The product of general procedure A was dissolved in 2:1 MeOH: H_2O followed by the slow addition of NaOH (1M, 5-10 equiv). The reaction then stirred for 1 hour. The reaction was carefully quenched with HCl and extracted with ether, dried with $MgSO_4$, and filter. The ether layer was then concentrated under reduced pressure. The title compound was isolated either by recrystallization or column chromatography (details below).

General Procedure C:

To a flame dried vial 1 equivalent of spirocycle was added along with DMA (0.1 M). 4 equivalents AcOH were added followed by 4 equivalents of acetic anhydride. Finally, 3 equivalents of N,N,N',N' -tetramethylmethanediamine was added. *Note: The order of addition was important to avoid complex reaction mixtures.* The reaction was then heated to 55 °C and stirred for 16 hours. The reaction was then cooled to room temperature and quenched with water and extracted with EtOAc. The organic layer was then washed with water (x5), dried with $MgSO_4$, filtered, and concentrated under reduced pressure. The title compounds were purified via column chromatography.

General Procedure D:

To a vial 1 equivalent (of conjugated enone was added followed by the addition of 3:1 (DMSO: H_2O, 0.1 M). 1.5 equiv. of IBX was added and the reaction was heated to 55 °C overnight. The reaction was then quenched with water and extracted with EtOAc. The organic layer was then washed with sodium bicarbonate (x3) to remove any IBX/IBA. The organic layer was dried with $MgSO_4$, filtered, and concentrated under reduced pressure.

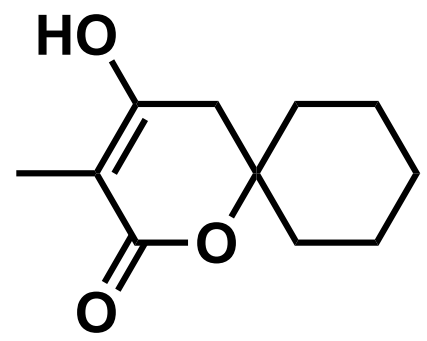

4-hydroxy-3-methyl-1-oxaspiro[5.5]undec-3-en-2-one (6): Prepared according to procedure A and B. 2-methyl-3-oxobutanoate (6.94 mmol, 1 equiv). The crude product of procedure A was taken directly into procedure B. Modified work up: Ether was then used to extract from the mixture. The resulting aqueous layer was then acidified with HCl and extracted again with ether. This ether layer was then concentrated under reduced pressure. Attempts to purify by chromatography initially used but the compound had the propensity to streak (00 to 100% EtOAc). The title compound **6** was isolated as a crystalline solid. (609 mg, 45% 31.0 mmol)

On larger scale (13.9 mmol, 2 grams scale and 48.6 mmol, 7 grams scale) we found could recrystallize the pure compound by treatment with cold ether filtering followed by washing with cold water, hexanes and methanol with minimal loss in yield (41% and 38% yield respectively).

^{1}H NMR (400 MHz, DMSO) δ 10.40 (s, 1H), 1.82 – 1.71 (m, 2H), 1.62 (d, *J* = 1.5 Hz, 3H), 1.59 – 1.20 (m, 10H).

^{13}C NMR (101 MHz, DMSO) δ 167.2, 163.3, 96.5, 76.7, 37.6, 35.5, 24.8, 21.4, 8.5.

HRMS (ESI+): calcd for $C_{11}H_{17}O_3$ $[M+1]^+$ 197.11722, found 197.11764.

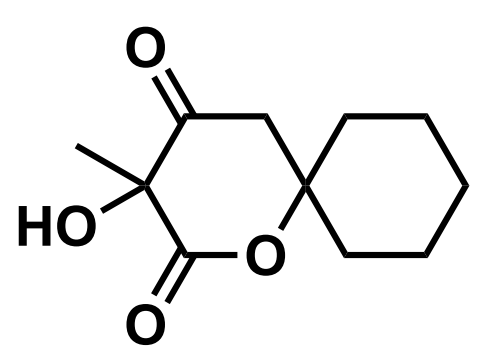

3-hydroxy-3-methyl-1-oxaspiro[5.5]undecane-2,4-dione (8): To a vial charged with a stir bar, 100 mg (0.501 mmol) of **6** was added followed by 1 mL of MeOH and 5 mL of H_2O (0.08M). 42.8 mg of $NaHCO_3$ (0.510 mmol, 1 equiv.) was added followed the 175 of magnesium monoperoxyphthalate hexahydrate (0.561 mmol, 1.1 equiv). The reaction ran for 16 hours before being quenched with DI water. The reaction was extracted with EtOAc (x3). The organic layers were dried with $MgSO_4$, filtered and concentrated under reduced pressure. The crude material was then purified via column chromatography (0 to 100% EtOAc: Hexanes) to afford 99 mg (92%, 0.47 mmol) as a white solid.

^{1}H NMR (600 MHz, $CDCl_3$) δ 3.56 (s, 1H), 3.04 (d, *J* = 14.3 Hz, 1H), 2.79 (d, *J* = 14.3 Hz, 1H), 2.00 – 1.93 (m, 1H), 1.85 (dtt, *J* = 14.4, 10.7, 3.5 Hz, 1H), 1.68 (s, 3H), 1.64 – 1.55 (m, 5H), 1.53 – 1.46 (m, 1H), 1.42 (ddd, *J* = 14.3, 10.6, 4.0 Hz, 1H), 1.38 – 1.22 (m, 1H).

^{13}C NMR (151 MHz, $CDCl_3$) δ 203.30, 172.91, 80.72, 78.12, 47.36, 37.64, 37.27, 28.04, 24.53, 21.51,

HRMS (APCI+): calcd for $C_{11}H_{17}O_4$ [M+1] 213.11214, found 213.11234

CCDC: 2541815

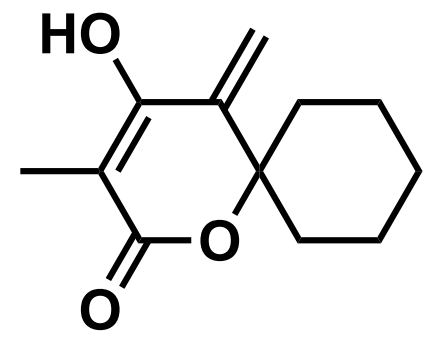

4-hydroxy-3-methyl-5-methylene-1-oxaspiro[5.5]undec-3-en-2-one (9): The title compound was prepared according to general procedure C. 100 mg of **6** (0.510 mmol, 1 equiv.) The title compound was purified via column chromatography (0 to 100% EtOAc: Hexanes) to afford 87 mg of **9** (82%, 0.42 mmol).

^{1}H NMR (400 MHz, $CDCl_3$) δ 5.75 (s, 1H), 5.35 (s, 1H), 2.14 – 2.04 (m, 2H), 1.93 (s, 3H), 1.87 – 1.80 (m, 1H), 1.77 – 1.70 (m, 1H), 1.62 – 1.56 (m, 4H), 1.27 – 1.20 (m, 2H).

^{13}C NMR (101 MHz, $CDCl_3$) δ 166.91, 156.94, 141.85, 112.73, 101.01, 80.98, 37.05, 25.14, 21.19, 9.15.

HRMS (APCI-): calcd for $C_{12}H_{15}O_3$ [M-1] 207.10267, found 207.10300

CCDC: 2541841

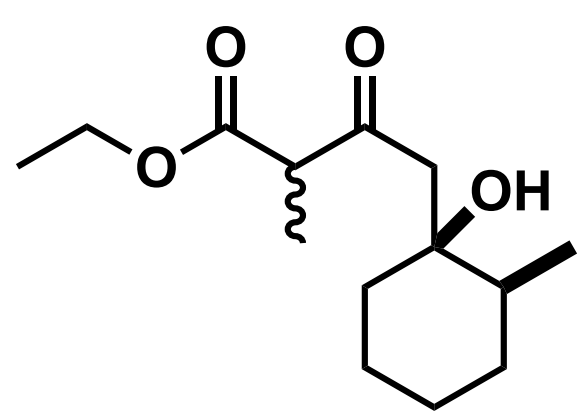

ethyl 4-(1-hydroxy-2-methylcyclohexyl)-2-methyl-3-oxobutanoate (10):

The title compound was prepared according to general procedure A: ethyl 2-methyl-3-oxobutanoate (6.94 mmol, 1 equiv.) Note: This reaction was cooled to -78 °C. The title compound was purified via column chromatography (0 to 100% EtOAc: Hexanes) to afford 0.810 g (46%). The title compound was isolated as a mixture of diastereomers at the alpha position(~1:1 d.r.). However, we see excellent diastereoselectivity (>10:1) at the quaternary alcohol.

Note: The reaction proceeded in higher yield (51%) when run at 0 ℃, however, the relative d.r. at the quaternary alcohol suffered as a result. We found running the reaction at -78 ℃ circumvented this poor d.r.

^{1}H NMR (400 MHz, $CDCl_3$) δ 4.26 – 4.09 (m, 2H), 3.61 – 3.43 (m, 1H), 3.21 (dd, J = 12.6, 1.0 Hz, 1H), 2.93 (dd, J = 22.7, 16.8 Hz, 1H), 2.62 (dd, J = 27.4, 16.8 Hz, 1H), 2.27 – 2.12 (m, 1H), 1.86 – 1.69 (m, 1H), 1.66 – 1.49 (m, 2H), 1.48 – 1.37 (m, 1H), 1.35 – 1.24 (m, 8H), 1.22 – 1.06 (m, 1H), 0.93 – 0.83 (m, 3H).

13C NMR (101 MHz, CDCl3) δ 208.90, 208.43, 170.24, 170.19, 73.38, 73.31, 61.65, 61.62, 54.81, 54.42, 49.90, 49.66, 46.74, 46.70, 37.20, 37.03, 26.10, 26.09, 25.26, 25.15, 21.87, 21.83, 21.76, 21.73, 14.23, 14.21, 12.65, 12.53.

HRMS (APCI+): calcd for $C_{13}H_{19}O_4$ $[M+1]^+$ 239.12779, found 239.12771

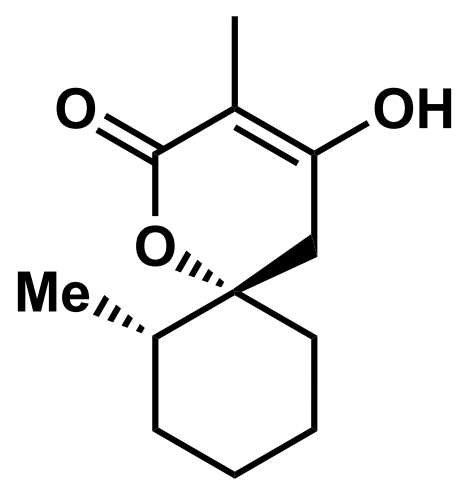

(6*S*,7*S*)-4-hydroxy-3,7-dimethyl-1-oxaspiro[5.5]undec-3-en-2-one (12): The title compound was prepared according to general procedure A and B: ethyl 2-methyl-3-oxobutanoate (6.94 mmol, 1 equiv.) The reaction was cooled to -78 °C. The reaction was worked up and concentrated as described in procedure A. The crude material was then subjected to procedure B. The resulting crude material was purified via column chromatography (00 to 100% EtOAc: Hexanes) to produce the title compound in 51% yield (0.740 grams, 3.52 mmol) as an oil over two steps.

^{1}H NMR (400 MHz, DMSO) δ 10.40 (s, 1H), 2.85 (dd, *J* = 17.5, 1.9 Hz, 1H), 2.17 (dd, *J* = 17.6, 1.5 Hz, 1H), 2.11 – 2.03 (m, 1H), 1.61 (t, *J* = 1.4 Hz, 3H), 1.59 – 1.54 (m, 2H), 1.41 (ddt, *J* = 12.5, 8.0, 4.1 Hz, 4H), 1.32 – 1.21 (m, 2H), 0.88 (d, *J* = 6.6 Hz, 3H).

^{13}C NMR (101 MHz, DMSO) δ 167.20, 163.69, 96.35, 78.13, 38.24, 35.54, 34.74, 29.53, 24.34, 21.15, 15.72, 8.54.

HRMS (APCI+): calcd for $C_{12}H_{19}O_3$ $[M+1]^+$ 211.13287, found 211.13303

CCDC: 2543179

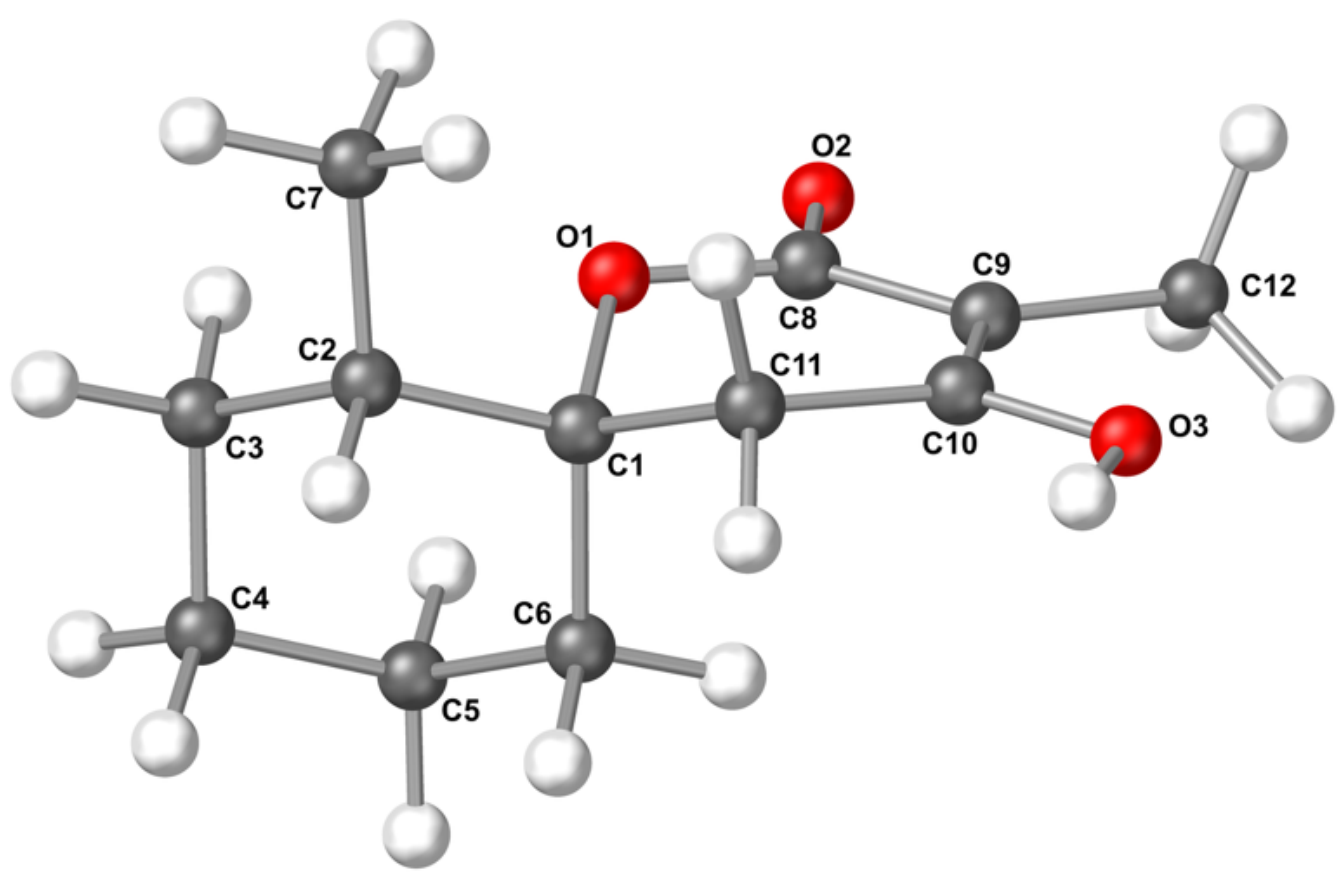

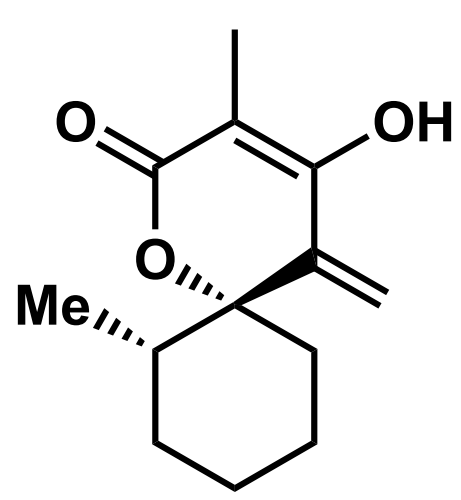

(6*R*,7*S*)-4-hydroxy-3,7-dimethyl-5-methylene-1-oxaspiro[5.5]undec-3-en-2-one (16): The title compound was prepared according to general procedure C. 526 mg (2.5 mmol) of **12**. The title compound was purified via column chromatography (0 to 100% EtOAc: Hexanes) to afford 223 mg of **16** (34%, 0.84 mmol).

^{1}H NMR (400 MHz, $CDCl_3$) δ 5.85 (s, 1H), 5.25 (s, 1H), 2.17 – 2.10 (m, 1H), 1.95 (s, 3H), 1.82 – 1.63 (m, 3H), 1.56 – 1.46 (m, 2H), 1.44 – 1.21 (m, 3H), 0.83 (d, *J* = 6.5 Hz, 3H).

^{13}C NMR (101 MHz, $CDCl_3$) δ 166.87, 156.88, 139.81, 112.68, 101.21, 84.35, 41.38, 40.72, 29.43, 25.78, 21.29, 16.03, 9.40.

HRMS (APCI+): calcd for $C_{13}H_{19}O_3$ [M+1]$^+$ 223.13287, found 223.13322

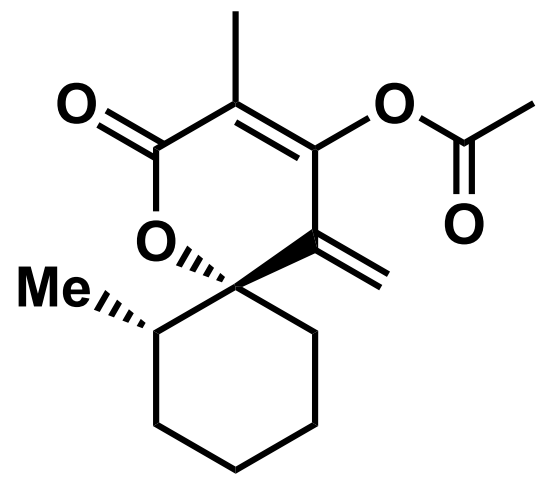

(6*R*,7*S*)-3,7-dimethyl-5-methylene-2-oxo-1-oxaspiro[5.5]undec-3-en-4-yl acetate (14): Following the procedure outline above for compound **16**, we isolated 11mg of **14** (21%, 0.52 mmol).

^{1}H NMR (400 MHz, CDCl₃) δ 5.37 (s, 1H), 5.15 (s, 1H), 2.28 (s, 3H), 2.21 – 2.12 (m, 1H), 1.81 (s, 3H), 1.78 – 1.58 (m, 4H), 1.56 – 1.08 (m, 4H), 0.83 (d, *J* = 6.6 Hz, 3H).

^{13}C NMR (101 MHz, CDCl₃) δ 166.66, 164.73, 152.96, 139.74, 116.85, 112.29, 85.54, 41.02, 29.25, 25.53, 20.92, 20.38, 15.84, 11.04.

HRMS (APCI+): calcd for $C_{13}H_{17}O_4$ [M+1]$^+$ 237.11323, found 237.11327

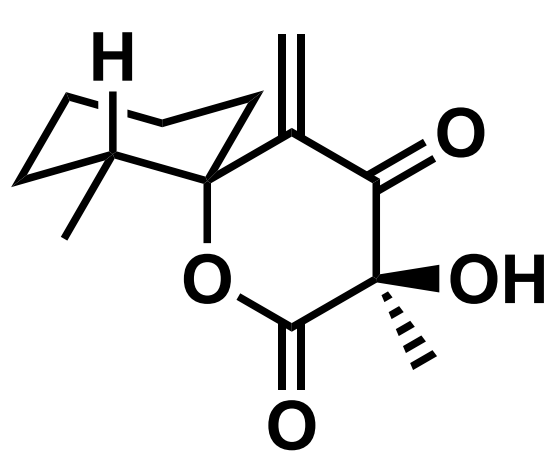

3-hydroxy-3,7-dimethyl-5-methylene-1-oxaspiro[5.5]undecane-2,4-dione (2a): The title compound was prepared according to general procedure D. 100 mg (0.49 mmol) of **16.** 24 mg of the title compound was isolated (22%). The title compound was purified via column chromatography (0 to 100% EtOAc: Hexanes).

^{1}H NMR (400 MHz, CDCl₃) δ 6.39 (s, 1H), 5.60 (s, 1H), 2.28 – 2.16 (m, 1H), 1.85 – 1.76 (m, 3H), 1.67 (ddd, *J* = 7.4, 3.5, 2.0 Hz, 2H), 1.63 (s, 3H), 1.59 – 1.47 (m, 1H), 1.43 – 0.98 (m, 2H), 0.73 (d, *J* = 6.0 Hz, 3H).

^{13}C NMR (101 MHz, CDCl₃) δ 195.13, 171.39, 145.18, 124.69, 87.32, 77.54, 45.02, 39.81, 29.91, 28.12, 25.43, 21.19, 15.49.

HRMS (APCI+): calcd for $C_{13}H_{19}O_4$ [M+1]$^+$ 239.12779, found 239.12732

Key HMQC Correlations:

	^{1}H Chemical Shift (ppm)	HMBC Correlation (ppm)
**1**	6.41	87.11
**2**	6.41	194.97
**3**	6.40	44.99
**4**	6.40	145.12

**5**	5.61	77.65
**6**	5.60	87.11
**7**	5.60	194.97
**8**	5.58	144.91
**9**	1.65	171.34
**10**	1.60	196.18
**11**	0.74	29.95
**12**	0.73	87.11
**13**	0.73	44.99

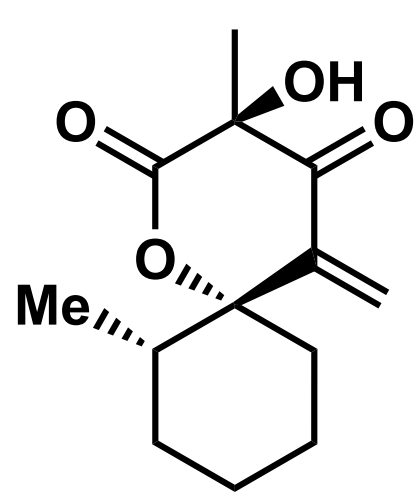

(3*S*,6*S*,7*R*)-3-hydroxy-3,7-dimethyl-5-methylene-1-oxaspiro[5.5]undecane-2,4-dione (2b): Compound **2b** was isolated in trace amounts due to decomposition into compound **19**. Multiple rounds of column chromatography and pTLC allowed for characterization. We found that compound 2b was unstable and decomposed readily into **19.**

^{1}H NMR (400 MHz, $CDCl_3$) δ 6.27 (s, 1H), 5.54 (s, 1H), 3.51 (s, 1H), 2.08 – 1.68 (m, 7H), 1.63 (s, 3H), 1.48 – 1.22 (m, 2H), 1.01 (d, J = 6.7 Hz, 3H).

^{13}C NMR (101 MHz, $CDCl_3$) δ 196.56, 172.16, 144.44, 123.01, 86.55, 77.92, 43.15, 37.57, 29.38, 28.17, 25.41, 21.23, 16.08.

HRMS (APCI+): calcd for $C_{13}H_{19}O_4$ $[M+1]^+$ 239.12779, found 239.12732

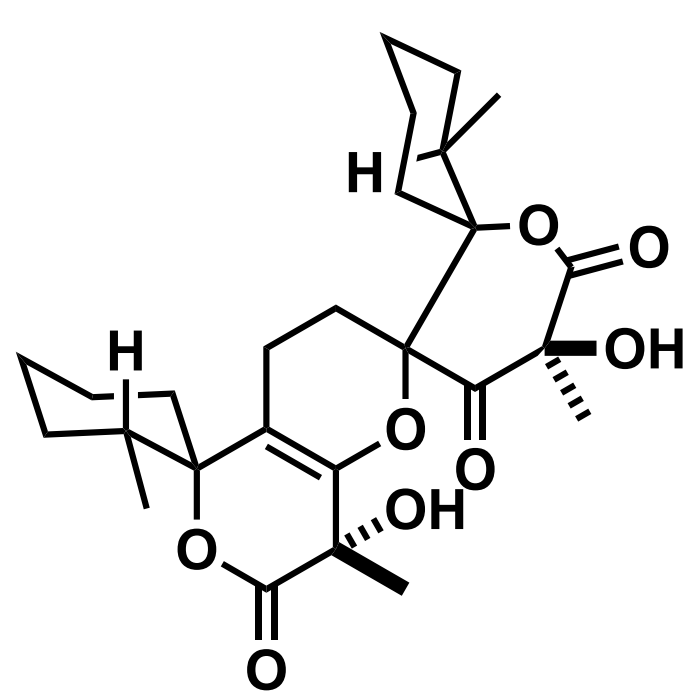

(1*S*,2*R*,2'''*R*,5'*S*,5''*S*,8''*S*)-5',8''-dihydroxy-2,2''',5',8''-tetramethyl-4'',8''-dihydro-4'*H*-trispiro[cyclohexane-1,2'-pyran-3',2''-pyrano[4,3-*b*]pyran-5'',1'''-cyclohexane]-4',6',7''(3''*H*,5'*H*)-trione (19): Compound 19 was isolated as a side product of the IBX oxidation of **16**. It appeared to be catalyzed in both $CDCl_3$ and SiO_2.

^{1}H NMR (400 MHz, $CDCl_3$) :δ 5.50 (d, *J* = 14.4 Hz, 1H), 3.61 (s, 1H), 2.60 (ddd, *J* = 17.7, 11.3, 7.3 Hz, 1H), 2.38 (dd, *J* = 13.8, 6.4 Hz, 2H), 2.08 – 2.03 (m, 1H), 2.02 – 1.86 (m, 2H), 1.75 (m, 6H), 1.68 (s, 3H), 1.64 (m,2H), 1.60 (s, 3H), 1.58 – 1.49 (m, 3H), 1.46 – 1.34 (m, 1H), 1.37 – 1.15 (m, 5H), 0.78 (d, *J* = 6.6 Hz, 3H), 0.73 (d, *J* = 6.7 Hz, 3H).

^{13}C NMR (101 MHz, $CDCl_3$) δ 202.42, 174.08, 172.15, 140.18, 111.16, 89.05, 87.73, 80.16, 74.28, 67.87, 53.57, 38.47, 37.39, 37.06, 31.10, 31.02, 28.97, 26.62, 25.59, 23.31, 21.00, 20.83, 20.65, 17.75, 16.90, 16.02.

HRMS: HRMS (ESI+): calcd for $C_{26}H_{36}O_8{}^{23}Na$ $[M+Na]^+$ 499.23024, found 499.23018

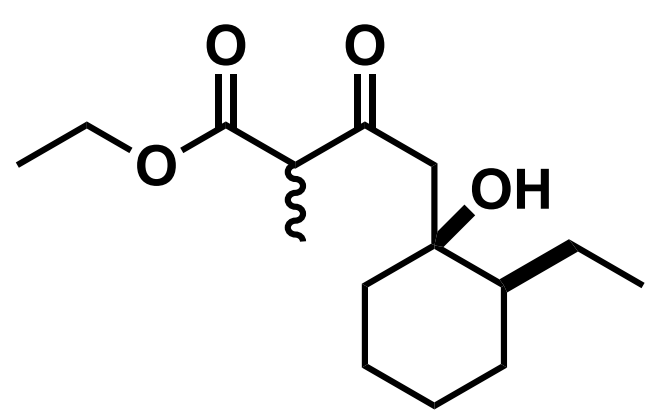

ethyl 4-((1*S*,2*S*)-2-ethyl-1-hydroxycyclohexyl)-2-methyl-3-oxobutanoate (11):

The title compound was prepared according to general procedure A using 6.94 mmol of ethyl 2-methyl-3-oxobutanoate (1 equiv., **S10**) The title compound was purified via column chromatography (0 to 100% EtOAc: Hexanes) to afford 1.12g (60%, 4.14 mmol). The title compound was isolated as a mixture of diastereomers (~1:1 d.r.).

^{1}H NMR (400 MHz, $CDCl_3$) δ 4.25 – 4.05 (m, 2H), 3.51 (dq, *J* = 12.0, 7.0 Hz, 1H), 2.91 (dd, *J* = 23.0, 16.9 Hz, 1H), 2.60 (dd, *J* = 28.2, 16.9 Hz, 1H), 1.81 – 1.51 (m, 6H), 1.44 – 1.33 (m, 1zH), 1.29 (dd, *J* = 7.1, 5.2 Hz, 3H), 1.24 (t, *J* = 7.1 Hz, 4H), 1.19 – 1.06 (m, 4H), 0.89 – 0.81 (m, 4H).

^{13}C NMR (101 MHz, $CDCl_3$) δ 208.95, 208.47, 170.17, 170.13, 73.31, 73.23, 61.60, 61.57, 54.70, 54.30, 49.83, 49.56, 46.63, 46.58, 37.09, 36.92, 25.98, 25.97, 25.20, 25.08, 21.79, 21.74, 21.67, 21.63, 14.16, 14.14, 12.58, 12.48, 12.47, 12.45.

HRMS (APCI+): calcd for $C_{15}H_{25}O_4$ $[M+1]^+$ 269.17583, found 269.17585

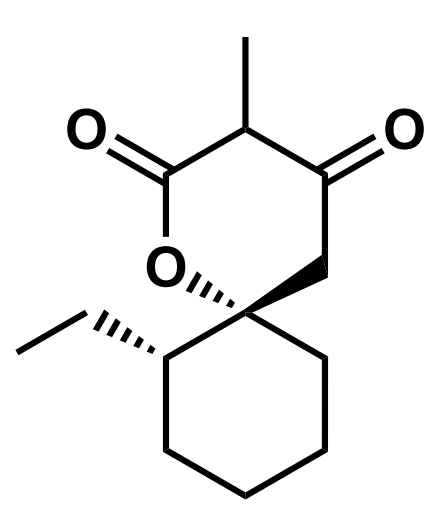

ethyl 4-((1*S*,2*S*)-2-ethyl-1-hydroxycyclohexyl)-2-methyl-3-oxobutanoate (13): The title compound was prepared according to general procedure B. 0.250 grams (0.925 mmols) of **11**. The crude material was purified via column

chromatography (0 to 100% EtOAc: Hexanes) to produce the title compound in 50% yield (0.105 grams, 0.47 mmol) as an oil.

^{1}H NMR (400 MHz, DMSO) δ 2.97 – 2.83 (m, 1H), 2.26 – 2.15 (m, 1H), 2.08 – 1.95 (m, 1H), 1.60 (s, 3H), 1.50 – 1.13 (m, 8H), 1.09 (t, J = 7.0 Hz, 2H), 0.85 (t, J = 7.4 Hz, 3H).

^{13}C NMR (101 MHz, DMSO) δ 167.61, 164.36, 96.78, 79.11, 65.41, 45.39, 35.94, 25.88, 21.93, 21.67, 15.65, 12.69, 9.05.

HRMS (APCI+): calcd for $C_{13}H_{21}O_3$ $[M+1]^+$ 225.14852, found 225.14779

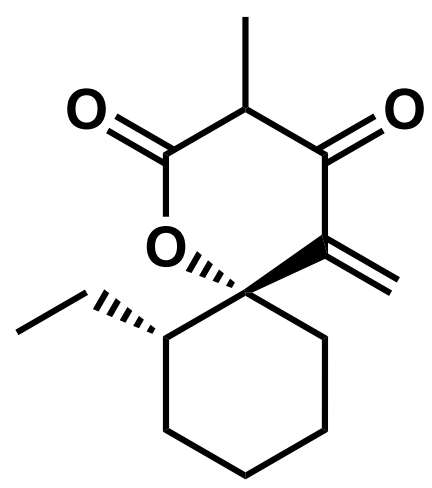

(6*R*,7*S*)-7-ethyl-3-methyl-5-methylene-1-oxaspiro[5.5]undecane-2,4-dione (17): The title compound was prepared according to general procedure C: 1.09 grams of **13.** The title compound was isolated via column chromatography (0 to 100% EtOAc: Hexanes) to produce 41% yield (0.470 grams, 1.99 mmol) of **17** as a foamy solid.

^{1}H NMR (400 MHz, CDCl$_3$) δ 5.89 (s, 1H), 5.21 (s, 1H), 2.20 – 2.06 (m, 1H), 1.95 (s, 3H), 1.81 – 1.71 (m, 3H), 1.55 – 1.36 (m, 5H), 1.28 – 1.02 (m, 2H), 0.83 (t, J = 7.5 Hz, 3H).

^{13}C NMR (101 MHz, CDCl$_3$) δ 167.40, 157.57, 140.31, 112.63, 101.12, 85.05, 47.66, 41.89, 25.70, 25.56, 23.01, 21.38, 12.10, 9.51.

HRMS (APCI+): calcd for $C_{14}H_{21}O_3$ $[M+1]^+$ 237.14852, found 237.14806

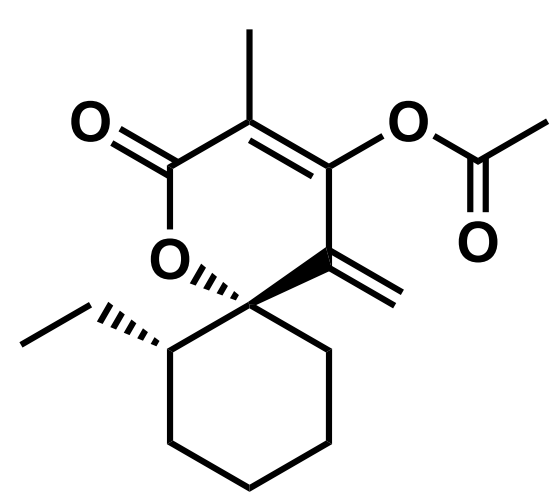

(6*R*,7*S*)-7-ethyl-3-methyl-5-methylene-2-oxo-1-oxaspiro[5.5]undec-3-en-4-yl acetate (15): Following the procedure outline above for compound **17**, we isolated 110 mg of **15** (8%, 0.36 mmol).

^{1}H NMR (400 MHz, CDCl$_3$) δ 5.39 (s, 1H), 5.15 (s, 1H), 2.31 (s, 3H), 2.26 – 2.16 (m, 1H), 1.85 (s, 3H), 1.82 – 1.70 (m, 2H), 1.60 – 1.37 (m, 6H), 1.33 – 1.19 (m, 1H), 1.17 – 0.98 (m, 1H), 0.85 (t, J = 7.4 Hz, 3H).

^{13}C NMR (101 MHz, CDCl$_3$) δ 166.76, 164.66, 153.06, 140.26, 117.10, 112.11, 86.28, 47.68, 41.83, 25.62, 25.52, 22.99, 21.19, 20.51, 12.08, 11.22.

HRMS (APCI+): calcd for $C_{16}H_{23}O_4$ $[M+1]^+$ 279.15909, found 279.15942

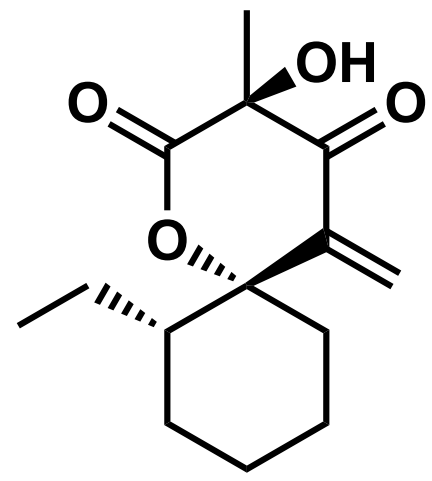

(3*R*,6*R*,7*S*)-7-ethyl-3-hydroxy-3-methyl-5-methylene-1-oxaspiro[5.5]undecane-2,4-dione (1a): The title compound was prepared according to general procedure D: 170 mg grams of **17.** The title compound was isolated via column chromatography (0 to 100% EtOAc: Hexanes) to produce 40.4 mg (22.3 %0.16 mmol) of **1a** an a oil.

^{1}H NMR (400 MHz, $CDCl_3$) δ 6.28 (s, 1H), 5.51 (s, 1H), 3.47 (s, 1H), 2.00 – 1.64 (m, 5H), 1.63 (s, 3H), 1.49 – 1.17 (m, 6H), 0.94 (t, *J* = 7.4 Hz, 3H).

^{13}C NMR (101 MHz, $CDCl_3$) δ 196.65, 172.13, 144.55, 122.87, 87.17, 77.92, 44.34, 43.45, 28.27, 25.44, 25.26, 23.17, 21.27, 12.06.

HRMS (APCI+): calcd for $C_{14}H_{21}O_4$ $[M+1]^+$ 253.14344, found 253.14364

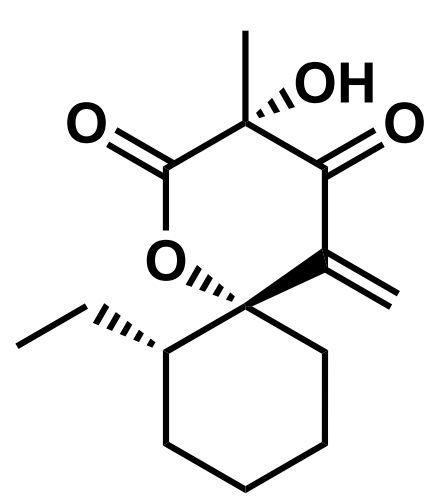

(3*R*,6*R*,7*S*)-7-ethyl-3-hydroxy-3-methyl-5-methylene-1-oxaspiro[5.5]undecane-2,4-dione (1b): The title compound was isolated from the reaction described above. The title compound was isolated via column chromatography (0 to 100% EtOAc: Hexanes) to produce 58.7 mg (32.3%, 0.23 mmol) of **1a** as an foamy solid

^{1}H NMR (400 MHz, $CDCl_3$) δ 6.40 (s, 1H), 5.60 (s, 1H), 3.72 (s, 1H), 2.24 (d, *J* = 10.7 Hz, 1H), 1.89 – 1.75 (m, 5H), 1.63 (s, 3H), 1.53 – 1.15 (m, 5H), 1.01 (ddt, *J* = 16.8, 13.8, 7.3 Hz, 1H), 0.79 (t, *J* = 7.4 Hz, 3H).

^{13}C NMR (101 MHz, $CDCl_3$) δ 196.65, 172.13, 144.55, 122.87, 87.17, 77.36, 44.34, 43.45, 28.27, 25.44, 25.26, 23.17, 21.27, 12.06.

HRMS (APCI+): calcd for $C_{14}H_{21}O_4$ $[M+1]^+$ 253.14344, found 253.14392

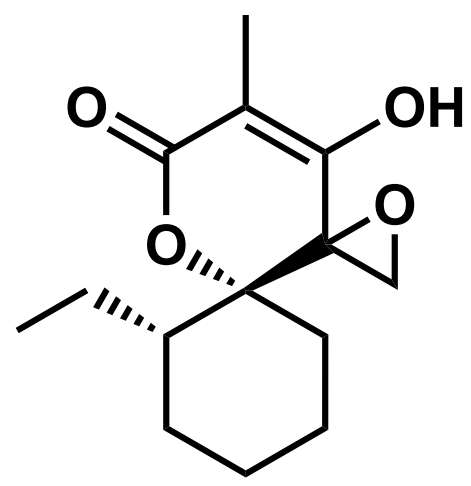

(3*S*,4*R*,5*S*)-5-ethyl-13-hydroxy-12-methyl-1,10-dioxadispiro[2.0.5^4.4^3]tridec-12-en-11-one (18): The title compound was isolated from the reaction described above. The title compound was isolated via column chromatography (0 to 100% EtOAc: Hexanes) to produce 48.7 mg (23.2%, 0.23 mmol) of **18** as an oil. The title compound was isolated an inseparable mixture of diastereomers.

^{1}H NMR (400 MHz, CDCl$_3$) δ 4.15 (d, *J* = 13.4 Hz, 1H), 3.87 (d, *J* = 11.7 Hz, 1H), 2.38 (s, 1H), 1.99 – 1.84 (m, 2H), 1.69 (dddd, *J* = 29.1, 24.9, 11.8, 7.7 Hz, 2H), 1.52 (s, 3H), 1.38 (td, *J* = 12.7, 4.9 Hz, 1H), 1.19 – 1.08 (m, 1H), 0.89 (dt, *J* = 20.4, 7.3 Hz, 6H).

^{13}C NMR (101 MHz, CDCl$_3$) δ 210.93, 210.48, 174.89, 174.79, 98.85, 98.20, 76.03, 70.02, 69.60, 64.19, 63.22, 44.43, 43.43, 34.02, 33.44, 26.65, 26.55, 25.30, 24.59, 23.01, 22.77, 21.25, 21.20, 20.15, 19.00, 11.98, 11.88.

HRMS (APCI+): calcd for 253.14344 $[M+1]^+$ 253.14353, found 253.14353

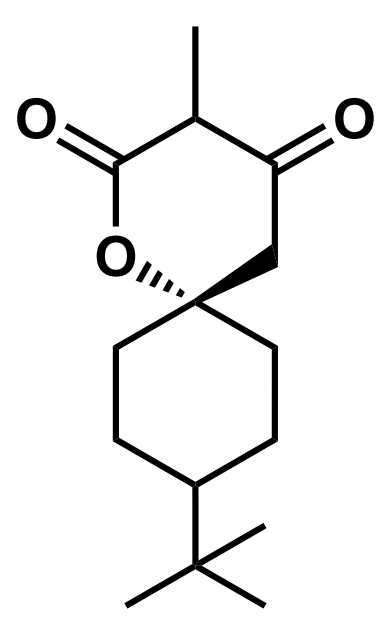

9-(*tert*-butyl)-4-hydroxy-3-methyl-1-oxaspiro[5.5]undec-3-en-2-one (S1): The title compound was prepared according to general procedure A-B with a slight modification. Ethyl 2-methyl-3-oxobutanoate (6.94 mmol, 1 equiv.) was used. During procedure A the reaction was quenched with 1M HCl and extracted with diethyl ether. The ether layer was dried with sodium sulfate and concentrated under reduced pressure. The concentrated material was carried forward to procedure B but only in 1M NaOH overnight (no MeOH). The reaction was quenched with 1M HCl and extracted and concentrated. The title compound was purified via column chromatography (0 to 80%, Diethyl Ether: Hexanes) to produce 557 mg (55.7 % over two steps).

^{1}H NMR (400 MHz, DMSO) δ 10.40 (s, 1H), 2.62 (d, *J* = 1.8 Hz, 1H), 2.43 (d, *J* = 1.8 Hz, 1H), 1.99 (td, *J* = 11.0, 2.9 Hz, 2H), 1.67 (d, *J* = 11.5 Hz, 1H), 1.61 (q, *J* = 1.3 Hz, 3H), 1.55 – 1.20 (m, 4H), 1.03 (dt, *J* = 16.3, 6.6 Hz, 2H), 0.84 (s, 9H).

^{13}C NMR (101 MHz, DMSO) δ 167.38, 167.23, 163.49, 163.37, 96.78, 96.48, 78.05, 75.76, 46.77, 46.44, 35.95, 35.72, 33.54, 32.13, 31.98, 27.42, 27.37, 23.38, 21.70, 8.60, 8.52.

HRMS: APCI (m/z) calcd. for $C_{15}H_{24}O_3$ [M+H+]: 253.17, found 253.17955.

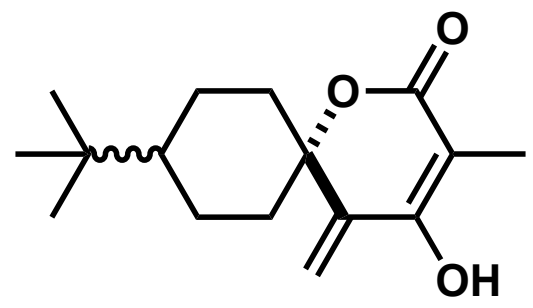

9-(*tert*-butyl)-4-hydroxy-3-methyl-5-methylene-1-oxaspiro[5.5]undec-3-en-2-one (21): The title compound was prepared according to procedure C: 544 mg (2.16 mmol) of **S1.** The title compound was isolated via column chromatography (0 to 100%, Diethyl Ether: Hexanes) to isolate 79.3 mg (14.6% yield). We isolated one diastereomers for the purposes of characterization (likely attributing to the poor yield). Because 21 was not product in the following oxidation we did not determine which diastereomer we isolated.

^{1}H NMR (400 MHz, DMSO) δ 10.07 (s, 1H), 5.72 (s, 1H), 5.45 (s, 1H), 2.03 – 1.94 (m, 2H), 1.77 (s, 3H), 1.70 – 1.53 (m, 4H), 1.38 (qd, J = 12.7, 3.0 Hz, 2H), 0.86 (s, 9H).

^{13}C NMR (101 MHz, DMSO) δ 165.77, 157.49, 142.10, 113.17, 100.24, 79.27, 46.42, 36.65, 32.17, 27.42, 21.89, 9.81.

HRMS: APCI (m/z) calcd for C16H24O3 [M+H+]: 265.17, found 265.1797.

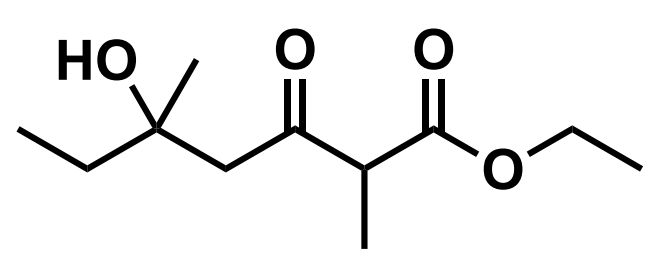

ethyl 5-hydroxy-2,5-dimethyl-3-oxoheptanoate (S2): The title compound was prepared according to general procedure A with ethyl 2-methyl-3-oxobutanoate (6.94 mmol, 1 equiv.) The title compound was purified via column chromatography (0 to 100% EtOAc: Hexanes) to afford 0.58 g (38.5%). The title compound was isolated as a mixture of diastereomers at the alpha position (~1:1 d.r.).

^{1}H NMR (400 MHz, $CDCl_3$) δ 4.19 (qd, J = 7.1, 1.2 Hz, 2H), 3.58 – 3.42 (m, 1H), 2.84 – 2.57 (m, 2H), 1.53 (pd, J = 7.4, 3.4 Hz, 2H), 1.33 (dd, J = 7.1, 0.8 Hz, 3H), 1.26 (t, J = 7.1 Hz, 3H), 1.19 (d, J = 4.2 Hz, 3H), 1.01 – 0.79 (m, 3H).

^{13}C NMR (101 MHz, $CDCl_3$) δ 208.07, 208.01, 170.21, 72.05, 61.65, 54.24, 54.20, 50.28, 50.24, 34.84, 34.53, 26.31, 26.14, 14.20, 12.64, 12.59, 8.36, 8.31.

HRMS (APCI-): calcd for $C_{11}H_{19}O_4$ [M-1] 215.12888, found 215.12923

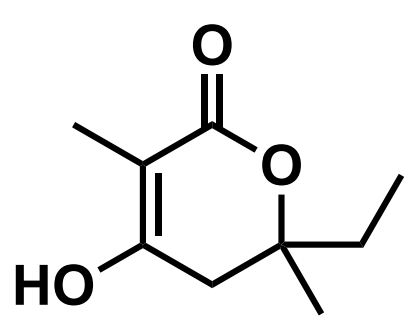

6-ethyl-4-hydroxy-3,6-dimethyl-5,6-dihydro-2*H*-pyran-2-one (S3): The title compound was prepared according to general procedure B with 200 mg of **S2** (0.86mmol). The title compound was purified via column chromatography (0 to 100% EtOAc: Hexanes) as a white solid.

Note: Analysis of compound S3 was difficult it excited in both enol and ketone form (d.r.: 1:1).

^{1}H NMR (400 MHz, DMSO) δ 2.57 (dq, J = 17.2, 1.6 Hz, 1H), 2.39 (dq, J = 17.2, 1.3 Hz, 1H), 1.73 – 1.53 (m, 5H), 1.26 (s, 3H), 0.86 (t, J = 7.5 Hz, 3H).

^{13}C NMR (101 MHz, DMSO) δ 167.85, 164.19, 97.03, 78.56, 37.21, 33.07, 24.67, 9.03, 8.41.

HRMS (APCI+): calcd for $C_9H_{15}O_3$ [M+1 171.10157, found 171.10205

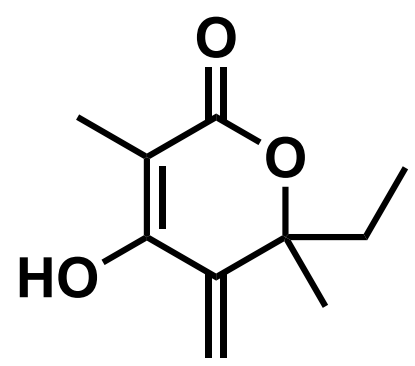

6-ethyl-4-hydroxy-3,6-dimethyl-5-methylene-5,6-dihydro-2*H*-pyran-2-one (22): The title compound was prepared according to general procedure C with 0.200 grams of **S3**. The title compound was purified via column chromatography (0 to 100% EtOAc: Hexanes) to afford 51 mg (24%) of **22** as a white solid.

^{1}H NMR (400 MHz, CDCl$_3$) δ 5.85 (s, 1H), 5.25 (s, 1H), 1.93 (s, 3H), 1.88 – 1.76 (m, 2H), 1.53 (s, 3H), 0.88 (t, *J* = 7.4 Hz, 3H).

^{13}C NMR (101 MHz, CDCl$_3$) δ 167.39, 157.21, 139.44, 113.65, 101.54, 83.57, 34.65, 27.59, 9.37, 8.45

HRMS (APCI+): calcd for $C_{10}H_{15}O_3$ [M+1] 183.10157 found 183.10104

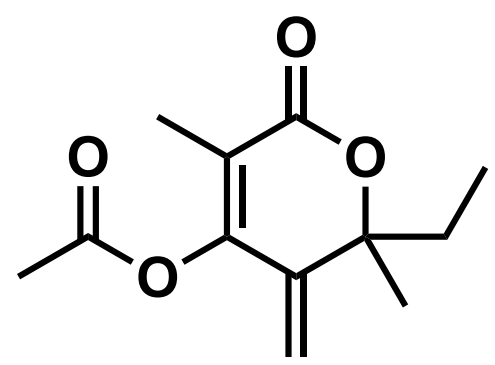

2-ethyl-2,5-dimethyl-3-methylene-6-oxo-3,6-dihydro-2*H*-pyran-4-yl acetate (S4): the title compound was isolated as a minor side product for the reaction described above.

^{1}H NMR (400 MHz, CDCl$_3$) δ 5.40 (s, 1H), 5.19 (s, 1H), 2.31 (s, 3H), 1.96 – 1.86 (m, 2H), 1.85 (s, 3H), 1.57 (s, 3H), 0.90 (t, *J* = 7.4 Hz, 3H).

^{13}C NMR (101 MHz, CDCl$_3$) δ 166.95, 165.12, 152.98, 139.37, 117.48, 113.11, 84.82, 34.73, 27.30, 20.55, 11.05, 8.33.

HRMS (APCI+): calcd for $C_{12}H_{17}O_4$ [M+1] 225.11214, found 225.11184

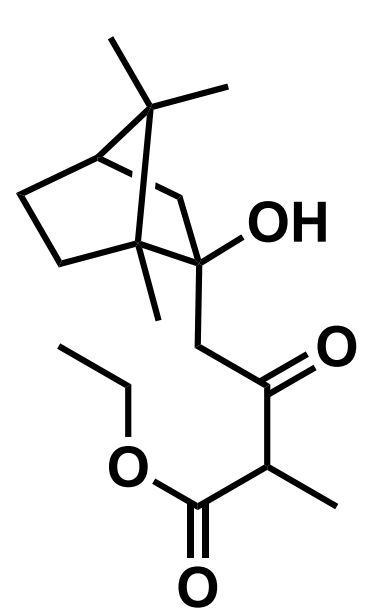

ethyl 4-((1*S*,2*S*,4*R*)-2-hydroxy-1,7,7-trimethylbicyclo[2.2.1]heptan-2-yl)-2-methyl-3-oxobutanoate (23): The title compound was prepared according to general procedure A using 6.94 mmol of ethyl 2-methyl-3-oxobutanoate (1 equiv.) The title compound was purified via column chromatography (0 to 100% EtOAc: Hexanes) to afford 1.43g (69.6%, 4.82 mmol) of the product. The product was isolated as a complex mixture of diastereomers at the alpha position along with Z/E enone isomers. As a result, we carried this material forward crude to lactonization for cleaner spectral analysis.

^{1}H NMR (400 MHz, CDCl$_3$) δ 4.19 (qdd, *J* = 7.1, 2.4, 1.7 Hz, 2H), 3.60 (d, *J* = 20.2 Hz, 1H), 3.54 (td, *J* = 7.1, 3.9 Hz, 1H), 2.98 – 2.62 (m, 2H), 2.19 – 2.06 (m, 1H), 1.75 – 1.67 (m, 2H), 1.47 – 1.36 (m, 2H), 1.34 (t, *J* = 7.1 Hz, 3H), 1.27 (td, *J* = 7.2, 2.4 Hz, 3H), 1.12 (d, *J* = 1.6 Hz, 3H), 0.98 – 0.87 (m, 2H), 0.85 (d, *J* = 2.5 Hz, 6H).

^{13}C NMR (101 MHz, CDCl$_3$) δ 208.97, 208.93, 170.65, 170.60, 80.03, 79.96, 61.98, 54.45, 54.20, 52.93, 52.82, 49.78, 49.52, 49.39, 49.37, 47.80, 47.51, 45.55, 45.54, 31.10, 31.04, 27.41, 27.34, 22.00, 21.97, 21.50, 21.49, 14.59, 14.55, 13.19, 12.97, 11.02

HRMS (APCI+): calcd for 295.19148 [M+1] $C_{17}H_{27}O_4$, found 295.19111

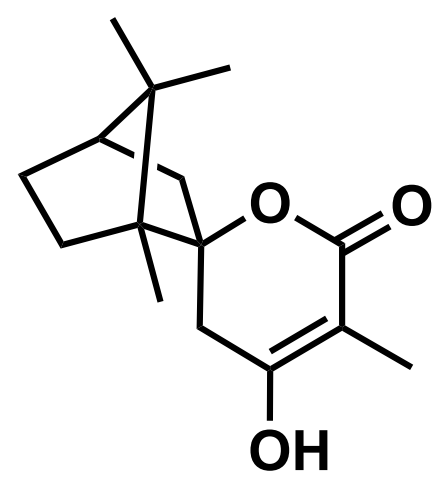

(1*S*,2*S*,4*R*)-4'-hydroxy-1,5',7,7-tetramethylspiro[bicyclo[2.2.1]heptane-2,2'-pyran]-6'(3'*H*)-one (24): The title compound was prepared via general procedure B with 0.500 grams (1.69 mmol) of **23**. The title compound was purified via column chromatography (0 to 100% EtOAc: Hexanes) to afford 227 mg of **24** (54%).

^{1}H NMR (400 MHz, CDCl$_3$) δ 3.43 (q, *J* = 6.7 Hz, 1H), 2.65 (d, *J* = 7.7 Hz, 2H), 2.47 – 2.35 (m, 1H), 1.90 (t, *J* = 4.6 Hz, 1H), 1.81 – 1.75 (m, 2H), 1.66 (d, *J* = 13.0 Hz, 1H), 1.60 – 1.43 (m, 2H), 1.36 (d, *J* = 6.6 Hz, 3H), 1.15 (s, 3H), 0.96 (d, *J* = 9.8 Hz, 6H).

^{13}C NMR (101 MHz, CDCl$_3$) δ 203.22, 169.68, 87.45, 53.27, 51.74, 49.40, 46.26, 45.56, 45.14, 30.16, 26.39, 21.00, 20.88, 10.26, 8.51.

HRMS (APCI+): calcd for $C_{15}H_{23}O_3$ [M+1] 251.16417, found 251.16428

$[\alpha]_D^{25}$ = -120.44(*c* = 2.1g/100 mL, CHCl$_3$)

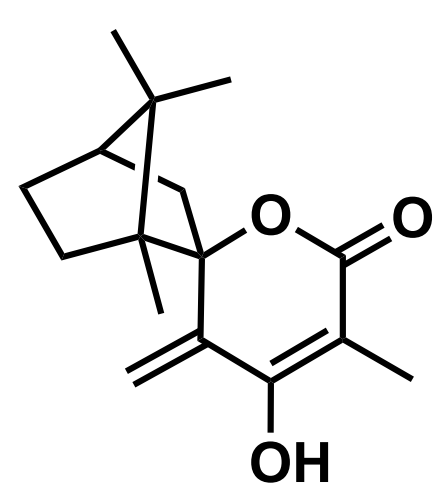

(1*S*,2*R*,4*R*)-4'-hydroxy-1,5',7,7-tetramethyl-3'-methylenespiro[bicyclo[2.2.1]heptane-2,2'-pyran]-6'(3'*H*)-one (25): The title compound was prepared according to general procedure C with 0.15 grams of **24.** The title compound was purified via column chromatography (0 to 100% EtOAc: Hexanes) to produce 54 mg of **25** (34%, 1.99 mmol) as a white solid.

^{1}H NMR (400 MHz, CDCl$_3$) δ 5.94 (s, 1H), 5.44 (s, 1H), 2.43 (dt, *J* = 14.6, 3.9 Hz, 1H), 1.96 (d, *J* = 14.6 Hz, 1H), 1.90 (s, 3H), 1.85 (t, *J* = 4.4 Hz, 1H), 1.80 – 1.64 (m, 2H), 1.43 – 1.29 (m, 2H), 1.24 (s, 3H), 1.07 (ddd, *J* = 12.6, 9.1, 5.2 Hz, 1H), 0.90 (s, 3H), 0.79 (s, 3H).

^{13}C NMR (101 MHz, CDCl$_3$) δ 166.73, 157.25, 137.29, 115.56, 102.31, 92.54, 55.02, 51.16, 44.91, 43.83, 29.67, 26.49, 22.03, 21.32, 12.35, 8.93.

1H NMR (800 MHz, DMSO) δ 5.89 (s, 1H), 5.57 (s, 1H), 2.15 (ddd, *J* = 14.3, 4.3, 3.1 Hz, 1H), 2.05 (d, *J* = 14.3 Hz, 1H), 1.78 (t, *J* = 4.4 Hz, 1H), 1.72 (s, 3H), 1.63 (tq, *J* = 12.0, 3.9 Hz, 1H), 1.32 – 1.21 (m, 2H), 1.12 (m, 4H), 0.85 (s, 3H), 0.70 (s, 3H).

^{13}C NMR (201 MHz, DMSO) δ 166.25, 158.59, 138.03, 116.05, 101.13, 91.07, 54.20, 50.31, 44.19, 42.41, 29.24, 25.44, 21.66, 20.91, 12.12, 9.43.

HRMS (APCI-): calcd for $C_{16}H_{21}O_3$ [M-1] 261.14962, found 261.14911

$[\alpha]_D^{25}$ = +51.867(*c* = 0.60g/100 mL, $CHCl_3$)

CCDC: 2547911

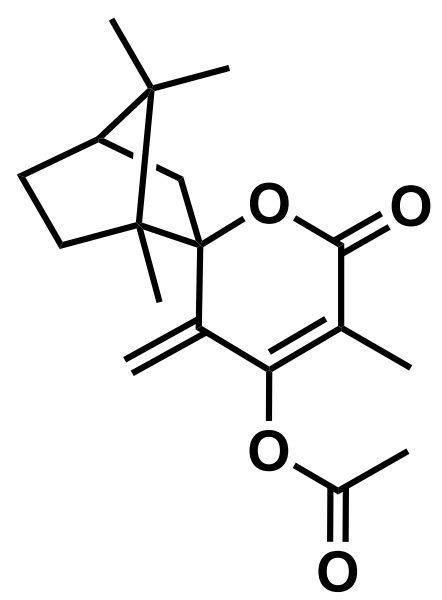

(1*S*,2*R*,4*R*)-1,5',7,7-tetramethyl-3'-methylene-6'-oxo-3',6'-dihydrospiro[bicyclo[2.2.1]heptane-2,2'-pyran]-4'-yl acetate (S5): The title compound was isolated as a minor side product from the reaction below 20%.

^{1}H NMR (400 MHz, $CDCl_3$) δ 5.55 (s, 1H), 5.36 (s, 1H), 2.43 (ddd, *J* = 14.7, 4.4, 3.2 Hz, 1H), 2.31 (s, 3H), 1.94 (d, *J* = 14.7 Hz, 1H), 1.82 (s, 3H), 1.72 (ddt, *J* = 20.1, 11.9, 3.1 Hz, 2H), 1.47 – 1.28 (m, 2H), 1.23 (s, 3H), 1.02 (ddd, *J* = 12.8, 9.2, 5.2 Hz, 1H), 0.90 (d, *J* = 6.2 Hz, 6H).

^{13}C NMR (101 MHz, $CDCl_3$) δ 167.45, 165.26, 153.80, 137.21, 117.83, 115.23, 93.77, 55.27, 51.14, 44.76, 43.91, 28.92, 26.39, 21.95, 21.18, 20.66, 11.83, 10.58.

HRMS (APCI+): calcd for $C_{18}H_{25}O_4$ [M+1] 305.17474, found 305.17439

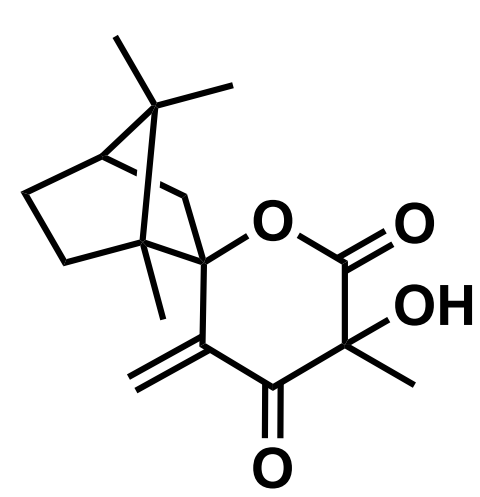

(1*S*,2*R*,4*R*)-5'-hydroxy-1,5',7,7-tetramethyl-3'-methylenespiro[bicyclo[2.2.1]heptane-2,2'-pyran]-4',6'(3'*H*,5'*H*)-dione (26): The title compound was prepared according to general procedure D with 0.050 grams (0.19 mmol) of **25.** The title compound was purified via column chromatography (0 to 100% EtOAc: Hexanes) to produce 26 mg of (49% 0.19 mmol) of **26** as an amorphous solid. The compound was isolated in 5.75:1 dr.

^{1}H NMR (400 MHz, $CDCl_3$) δ 6.20 (s, 1H), 5.70 (s, 1H), 2.49 (dt, *J* = 14.6, 3.9 Hz, 1H), 2.02 (d, *J* = 14.5 Hz, 1H), 1.91 (t, *J* = 4.5 Hz, 1H), 1.81 – 1.70 (m, 1H), 1.69 (d, *J* = 4.5 Hz, 3H), 1.44 (ddd, *J* = 14.1, 11.8, 5.2 Hz, 1H), 1.32 (ddd, *J* = 14.1, 9.2, 4.0 Hz, 1H), 1.27 – 1.18 (m, 5H), 1.05 (td, *J* = 8.6, 4.6 Hz, 1H), 0.91 (s, 3H), 0.79 (s, 3H).

^{13}C NMR (101 MHz, $CDCl_3$) δ 197.07, 172.35, 141.79, 124.92, 94.01, 74.02, 54.55, 51.01, 44.84, 44.56, 30.41, 26.31, 22.92, 21.77, 21.34, 12.22.

HRMS (APCI+): calcd for $C_{16}H_{23}O_4$ [M+1] 279.15909, found 279.15927

[α]$_D^{25}$ = -21.71(*c* = 0.15g/100 mL, CHCl$_3$)

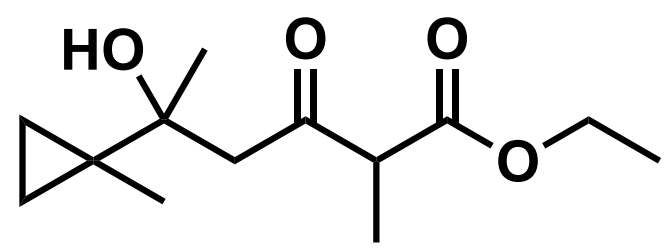

ethyl 5-hydroxy-2-methyl-5-(1-methylcyclopropyl)-3-oxohexanoate (S6): The title compound was prepared according to general procedure A with ethyl 2-methyl-3-oxobutanoate (6.94 mmol, 1 equiv.) The title compound was purified via column chromatography (0 to 100% EtOAc: Hexanes) to afford 0.739 g (44.0%, 3.05 mmol). The title compound was isolated as a mixture of diastereomers at the alpha position.

^{1}H NMR (400 MHz, CDCl$_3$) δ 4.27 – 4.13 (m, 2H), 3.57 (dq, *J* = 12.3, 7.1 Hz, 1H), 3.50 (d, *J* = 2.9 Hz, 1H), 2.90 – 2.73 (m, 2H), 1.33 (t, *J* = 6.9 Hz, 3H), 1.29 – 1.19 (m, 6H), 1.03 (d, *J* = 4.0 Hz, 3H), 0.79 – 0.63 (m, 2H), 0.12 (dddd, *J* = 10.9, 8.7, 7.6, 1.6 Hz, 2H).

^{13}C NMR (101 MHz, CDCl$_3$) δ 208.46, 208.29, 170.22, 72.19, 72.13, 61.64, 61.61, 54.48, 54.37, 48.87, 48.61, 25.62, 25.46, 23.63, 23.58, 21.64, 21.57, 14.20, 12.72, 12.46, 10.38, 10.28, 9.34, 9.29.

HRMS (APCI+): calcd for $C_{13}H_{21}O_4$ [M+1] 241.14453, found 241.14456

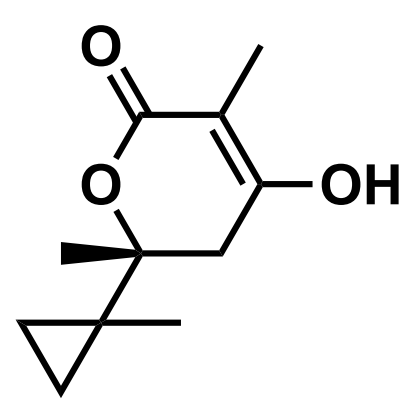

(*R*)-4-hydroxy-3,6-dimethyl-6-(1-methylcyclopropyl)-5,6-dihydro-2*H*-pyran-2-one (S7): The title compound was prepared via general procedure B with 0.739 grams (3.05 mmol) of **S6**. The title compound was purified via column chromatography (0 to 100% EtOAc: Hexanes) to afford 527 mg of **S7** (88%). We found the compound **S7** existed in the enol and ketone form. In the ketone form, the compound existed as a mixture of diastereomers making the analysis of **S7**, very difficult. As such, we carried **S9** forward before complete characterization.

HRMS (APCI+): calcd for $C_{11}H_{17}O_3$ [M+1] 197.11722, found 197.11689

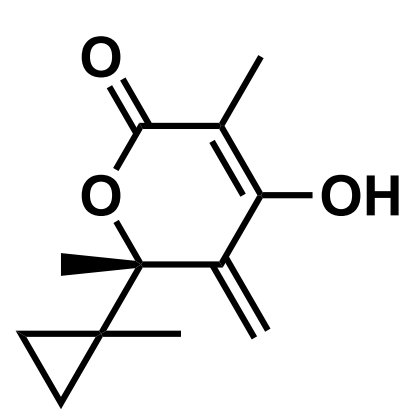

(4-hydroxy-3,6-dimethyl-6-(1-methylcyclopropyl)-5-methylene-5,6-dihydro-2*H*-pyran-2-one (27): The title compound was prepared according to general procedure C with 0.390 grams of **S7.** The title compound was purified via column

chromatography (0 to 100% EtOAc: Hexanes) to produce 349 mg of **27** (84.3%, 1.67 mmol mmol). In solution, compound **21** existed as a 3:1 ratio between the ketone and enol form. The peaks below only denote the enol confirmer.

1H NMR (400 MHz, CDCl3) δ 5.86 (s, 1H), 5.39 (s, 1H), 1.94 (s, 3H), 1.45 (s, 3H), 1.07 (s, 3H), 0.70 – 0.48 (m, 2H), 0.41 (dt, *J* = 9.0, 4.6 Hz, 1H), 0.31 (dt, *J* = 9.6, 4.8 Hz, 1H).

^{13}C NMR (101 MHz, CDCl$_3$) δ 191.75, 138.48, 124.77, 114.83, 101.55, 85.08, 25.06, 23.15, 21.16, 11.70, 10.26, 9.29.

HRMS (APCI+): calcd for $C_{12}H_{17}O_3$ [M+1] 209.11722, found 209.11685

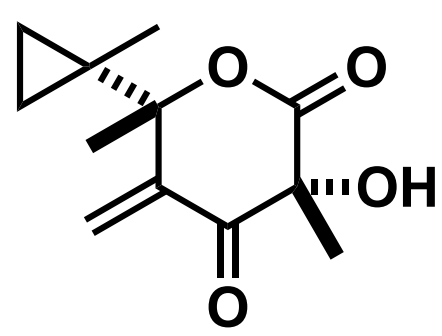

(3*R*,6*S*)-3-hydroxy-3,6-dimethyl-6-(1-methylcyclopropyl)-5-methylenedihydro-2*H*-pyran-2,4(3*H*)-dione (28):

The title compound was prepared according to general procedure D with 0.100 grams (0.48 mmol) of **27.** The title compound was purified via column chromatography (0 to 100% EtOAc: Hexanes) to produce 33 mg of (31%, 0.147 mmol) of **28** as an amorphous solid. The compound was isolated in 5.75:1 dr.

^{1}H NMR (400 MHz, CDCl$_3$) δ 6.22 (s, 1H), 5.57 (s, 1H), 3.54 (s, 1H), 1.67 (d, *J* = 1.2 Hz, 3H), 1.62 (d, *J* = 1.2 Hz, 3H), 1.06 (s, 3H), 0.67 (dt, *J* = 9.5, 4.9 Hz, 1H), 0.52 (dt, *J* = 10.4, 4.9 Hz, 1H), 0.37 (dtd, *J* = 19.3, 9.3, 5.1 Hz, 2H).

^{13}C NMR (101 MHz, CDCl$_3$) δ 196.96, 172.20, 143.70, 124.11, 87.16, 78.19, 29.04, 25.38, 23.92, 20.85, 10.50, 10.29,

HRMS (APCI+): calcd for $C_{12}H_{17}O_4$ [M+1] 225.11214, found 225.11145

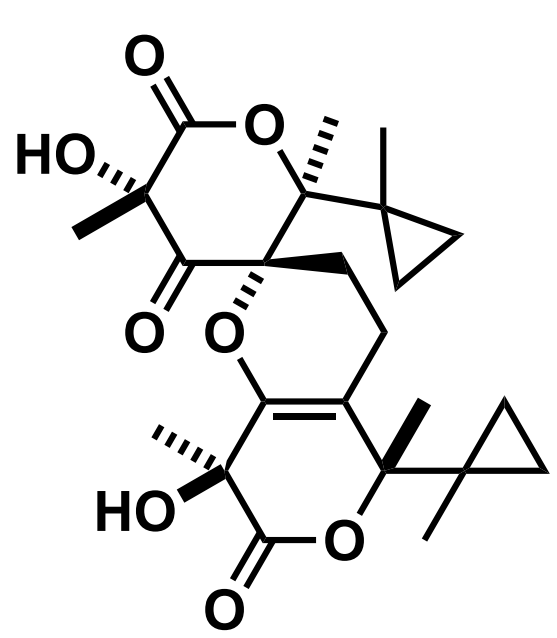

5,8'-dihydroxy-2,5,5',8'-tetramethyl-2,5'-bis(1-methylcyclopropyl)-3',4',5,5',8'-pentahydro-2*H*,4*H*,7'*H*-spiro[pyran-3,2'-pyrano[4,3-*b*]pyran]-4,6,7'-trione (29).

The title compound was isolated as the major byproduct for the reaction described above (52%, 57 mg, 0.13mmol). It was isolated as a white powder.

^{1}H NMR (400 MHz, Acetone) δ 2.50 (d, *J* = 6.2 Hz, 2H), 2.46 (t, *J* = 3.6 Hz, 0H), 1.61 (s, 3H), 1.58 (d, *J* = 2.6 Hz, 6H), 1.18 (s, 6H), 0.93 (s, 3H), 0.82 (ddt, *J* = 24.5, 10.1, 4.7 Hz, 2H), 0.72 (dt, *J* = 9.8, 5.1 Hz, 1H), 0.55 – 0.45 (m, 2H), 0.34 – 0.20 (m, 2H).

^{13}C NMR (101 MHz, Acetone) δ 204.24, 170.30, 143.46, 110.81, 86.95, 78.45, 77.80, 74.87, 67.15, 24.92, 23.32, 22.45, 22.21, 21.61, 21.48, 21.13, 20.79, 20.19, 18.60, 12.12, 11.62, 9.64, 7.74.

HRMS (APCI+): calcd for $C_{24}H_{33}O_8$ [M+1] 449.21699, found 449.21604

CCDC: 2549876

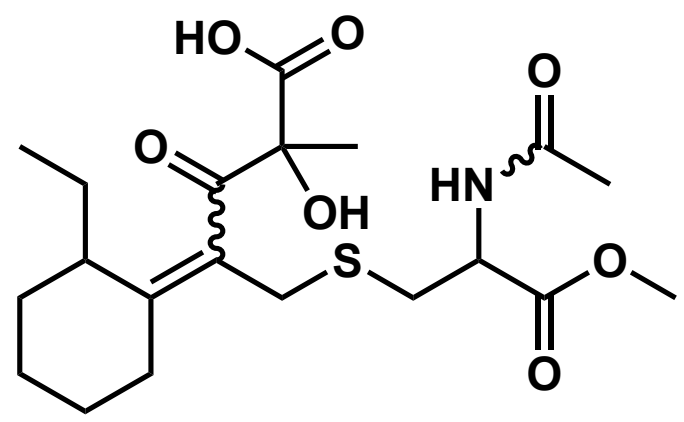

5-((2-acetamido-3-methoxy-3-oxopropyl)thio)-4-(2-ethylcyclohexylidene)-2-hydroxy-2-methyl-3-oxopentanoic acid (30).

To a vial N-acetyl-cysteine-OMe (14.05 mg, 0.079 mmol) was added followed by 6.4 mL of 100 mM sodium acetate, 150 mM sodium chloride pH = 5.0 buffer. Then 1.6 mL of ACN was added to increase solubility. Finally, **1b** (24 mg, 0.95 mmol, 1.2 equiv.,) was added. The reaction ran overnight. The following morning the ACN was removed via speedvac. The aqueous mixture was then extracted with EtOAc (X3) and DCM (X3). The combined organic layer was dried with MgSO4, filtered, and concentrated under reduced pressure. The Crude mixture was then passed through a silica plug (DCM:MeOH, 0→10%).

HRMS (ESI+): calcd for $C_{20}H_{31}O_7N^{23}Na^{32}S$ [M-1] 452.17134, found 452.17143

Analytical Scale Competition:

A 10 mM solution of **1b** was prepared in 80:20 H2O:ACN along with a 10mM solution of N-acetyl-cysteine-OMe, Boc-Lys-OMe, and Boc-Lys-OMe. To a LCMS vial, 100 µL of PBS phosphate buffer, pH = 7.4 buffer was added followed by 25 µL of **1b** and then 25 µL of each amino acid solution. The reaction was directly monitored at different time points for the completion of the reaction.

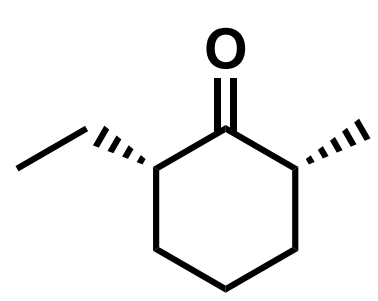

2-ethyl-6-methylcyclohexan-1-one (S8)

To a flame dried RBF, 0.500 grams of 2-ethylidene-6-methylcyclohexan-1-one (prepared according to Zhang, 3.62 mmol), was added followed by 20 mL of benzene (0.18 M). 335 mg of Wilkinson catalyst (10 mol%, 0.36 mmol) was added. The reaction was then sparged with hydrogen gas (x3) and let run overnight. The reaction was then loaded directly onto a silica column (pentane: ether 00->50%). Compound S10 was *carefully* concentrated under reduce pressure. 488 mg of **S9** was isolated (96% yield) in >10:1 d.r. The compound matched literature reference.[2]

^{1}H NMR (400 MHz, CDCl$_3$) δ 2.29 (dp, *J* = 12.6, 6.3 Hz, 1H), 2.13 – 1.93 (m, 2H), 1.79 – 1.56 (m, 3H), 1.31 – 1.01 (m, 5H), 0.90 (dd, *J* = 6.5, 1.5 Hz, 3H), 0.83 – 0.75 (m, 3H).

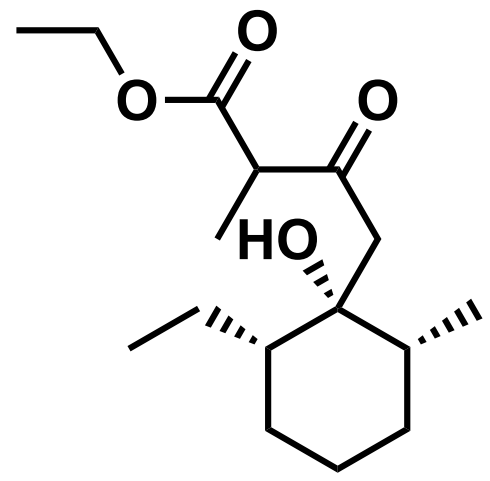

ethyl 4-((2*S*,6*R*)-2-ethyl-1-hydroxy-6-methylcyclohexyl)-2-methyl-3-oxobutanoate (S9): The title compound was prepared according to general procedure A except the initial addition was run at -78 °C using 1.07 mmol mmol of ethyl 2-methyl-3-oxobutanoate (1 equiv.) The title compound was purified via column chromatography (0 to 100% EtOAc: Hexanes) to afford 37mg (13%, 0.13 mmol) of the product.

^{1}H NMR (600 MHz, CDCl$_3$) δ 4.19 (qd, *J* = 7.2, 2.0 Hz, 2H), 3.53 (qd, *J* = 7.1, 1.0 Hz, 1H), 2.93 (dd, *J* = 18.5, 7.4 Hz, 1H), 2.83 (dd, *J* = 18.5, 5.1 Hz, 1H), 2.57 (d, *J* = 7.1 Hz, 1H), 1.77 – 1.64 (m, 3H), 1.51 – 1.37 (m, 4H), 1.33 (d, *J* = 7.1 Hz, 3H), 1.27 (td, *J* = 7.1, 0.8 Hz, 3H), 1.22 – 1.10 (m, 2H), 0.89 – 0.82 (m, 6H).

^{13}C NMR (151 MHz, CDCl$_3$) δ 206.27, 206.26, 170.37, 170.35, 75.73, 61.64, 61.62, 54.06, 54.05, 46.44, 46.42, 46.12, 46.04, 39.52, 39.50, 30.56, 30.54, 26.17, 26.12, 25.69, 22.83, 16.28, 14.21, 13.03, 12.64, 12.58.

HRMS (APCI-): calcd for $C_{16}H_{27}O_4$ [M-1] 283.19148, found 283.1913

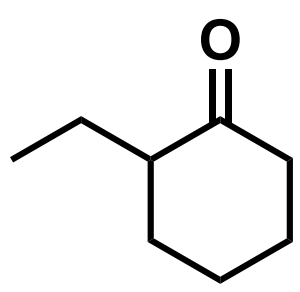

2-ethylcyclohexan-1-one (S10): to a flame dried RBF charged with a stir bar 2.2 mL (15.6 mmol) of 2-ethylcyclohexan-1-ol was added along with 100 ml of DCM (0.16M). The reaction was cooled to 0 °C. 7.28 g (1.72 mmol, 1.1 equiv.) of Dess-Martin-Periodinane was added directly followed by sodium carbonate (1.98 g, 18.7 mmol, 1.2 equiv). The reaction ran over night and allowed to warm to room temperature. The reaction was directly filtered over a pad of celite and carefully concentrated under reduced pressure. The crude compound was then loaded onto a silica column (pentane : ether 0→50%). The title compound S10 was isolated as a clear oil in 99% yield (1.95 grams, 15.5 mmol). The title compound match literature.[3]

^{1}H NMR (400 MHz, CDCl$_3$) δ 2.36 – 2.30 (m, 1H), 2.24 (td, *J* = 12.4, 5.8 Hz, 1H), 2.15 (dq, *J* = 12.0, 6.2 Hz, 1H), 2.11 – 2.03 (m, 1H), 1.98 (q, *J* = 5.5 Hz, 1H), 1.86 – 1.78 (m, 1H), 1.69 – 1.56 (m, 2H), 1.40 – 1.27 (m, 1H), 1.21 (dp, *J* = 14.2, 7.3 Hz, 1H), 0.84 (dd, *J* = 8.1, 6.9 Hz, 3H).

D. Biological Procedures

Broth Microdilution Antimicrobial Susceptibility Testing:

Compounds were serially diluted two-fold from stock solutions (10 mM in DMSO) to yield twelve 100-µL test concentrations. Freezer stocks of bacteria were used to inoculate Mueller Hinton broth (MHB, 5 mL) and cultures were grown at 37 °C for 18 hours with shaking (200 rpm). Overnight cultures were diluted 1:100 in MHB and regrown to mid-exponential phase as determined by optical density at 600 nm (OD_{600}). All cultures were diluted to 10^4 CFU/mL in MHB and 100 µL were inoculated into each well of a U-bottom 96-well plate (Avantor) containing 100 µL of antibiotic solution. Plates were incubated at 37 °C for 24 hours upon which wells were evaluated visually for bacterial growth. The MIC was determined as the lowest concentration of compound resulting in no bacterial growth visible to the naked eye based on the mean of three independent experiments. MHB, DMSO, bacteria, and known antimicrobial controls were conducted for each of replicates.

Checkerboard Assay (Synergy):

One compound was serially diluted 2-fold in media across in rows to yield twelve 100-µL test concentrations. The second compound was added to the top row and subsequently serially diluted down the columns of the plate. Freezer stocks of bacteria were used to inoculate Mueller Hinton broth (MHB, 5 mL) and cultures were grown at 37 °C for 18 hours with shaking (200 rpm). Overnight cultures were diluted 1:100 in MHB and regrown to mid-exponential phase as determined by optical density at 600 nm (OD_{600}). All cultures were diluted to 10^4 CFU/mL in MHB and 100 µL were inoculated into each well of a U-bottom 96-well plate (Avantor) containing 100 µL of the mixed compound solutions. Plates were incubated at 37 °C for 24 hours upon which the OD_{600} of each well was measured. Growth was normalized to media control and negative control. The assay was tested in triplicate from three separate overnight cultures.

E. Chemical Space

General optimization procedure:

The natural product and AOG analogs were minimized using Jaguar within Maestro the following parameters were used:

Theory = B3LYP-D3

Basis set = 6-31G**

SCF spin treatment = automatic

Properties selected:

Vibration frequency

Surfaces (MO, density, potential)

Atomic electrostatic potential charges (ESP)

Solent model = none

For synthetic intermediates the structures were quickly minimized using MacroModel within Maestro with a OPLS4 force field.

All resulting minimized structures were exported as a .smiles file, a XYZ coordinate files, and a .mol2 file.

The chemical complexity for each compound was calculated using https://forlilab.org/code/bottcher/.[4,5]

The Moments of interia were calculate using https://www.colby.edu/chemistry/PChem/scripts/ABC.html

Compound		Böttcher score	Ix	Iy	Iz		I1/3	I2/3	Chemical Complexity
AOG 1		12.62	1361.25	1123.52	562.342		0.41311	0.82536	12.62
AOG 2		12.62	1361.24	1123.29	562.415		0.41316	0.8252	12.62
AOG 3		12.41	2798.05	2524.07	705.744		0.25223	0.90208	12.41
AOG 4		12.41	2796.77	2521.06	707.025		0.2528	0.90142	12.41
AOG 5		16.14	1419.2	1277.56	650.514		0.45837	0.9002	16.14
AOG 6		16.14	1419.22	1243.09	666.11		0.46935	0.8759	16.14
AOG 7		15.58	1529.78	1438.16	829.55		0.54227	0.94011	15.58
AOG 8		15.58	1623.56	1426.31	819.219		0.50458	0.87851	15.58
AOG 9		16.92	882.817	755.751	543.12		0.61521	0.85607	16.92
AOG 10		16.92	934.863	780.077	520.481		0.55675	0.83443	16.92
AOG 11		15.55	1238.93	1095.29	578.445		0.46689	0.88406	15.55
AOG 12		15.55	1305.12	1133.56	558.894		0.42823	0.86855	15.55
AOG 13		15.72	1923.5	1646.63	807.949		0.42004	0.85606	15.72
AOG 14		15.72	1869.5	1615.87	810.312		0.43344	0.86433	15.72
AOG A		15.68	1641.73	1474.95	962.654		0.58637	0.89842	15.68
AOG B		15.68	1641.6	1458.39	965.104		0.5879	0.88839	15.68

Compound	Böttcher score	Ix	Iy	Iz		npr1	npr2	Chemical Complexity
Gu_1	9.83	403.7943	256.8447	182.6209		0.4522622	0.6360781	9.83
Gu_2	10.42	711.9868	510.6767	240.1563		0.3373044	0.7172559	10.42
Gu_3	9.47	1690.4175	1532.8999	318.5504		0.1884448	0.9068173	9.47
Gu_4	9.38	712.3419	514.7937	242.4676		0.3403809	0.7226778	9.38
Gu_5	10.22	738.1007	533.6607	249.9254		0.3386061	0.7230188	10.22
Gu_6	12.09	981.0992	656.0042	579.9791		0.5911524	0.6686421	12.09
Gu_7	12.62	1143.6638	788.4152	683.6551		0.5977763	0.6893767	12.62
Gu_8	16.57	1798.9506	1676.8999	879.7005		0.4890076	0.9321545	16.57
Gu_9	15.68	1710.6953	1549.9729	976.8317		0.5710144	0.9060485	15.68
ARL_1	9.41	576.1557	400.516	218.0786		0.3785064	0.6951524	9.41
ARL_2	13.26	1526.7733	1330.6243	671.2769		0.4396703	0.8715271	13.26
ARL_3	13.01	1501.3082	1297.1338	784.2875		0.5224027	0.8640023	13.01
ARL_4	15.58	1675.9782	1541.6546	822.3349		0.4906597	0.9198536	15.58

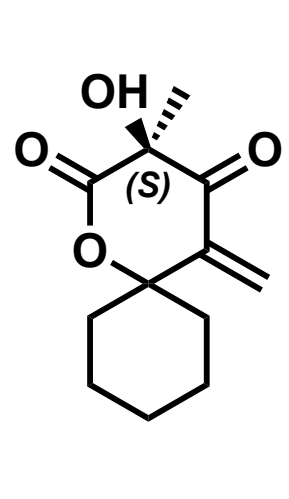

AOG_1

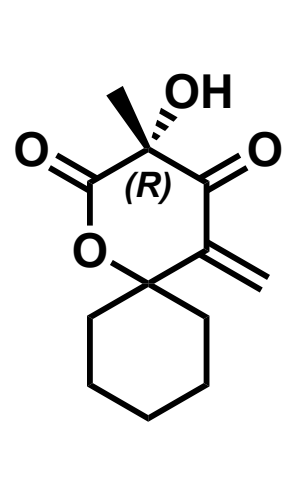

AOG_2

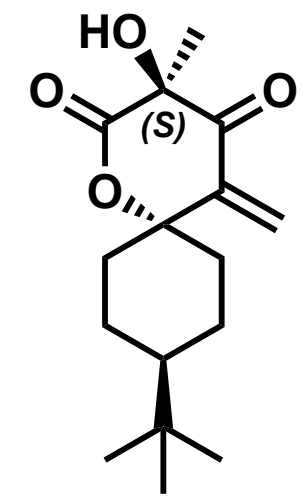

AOG_3

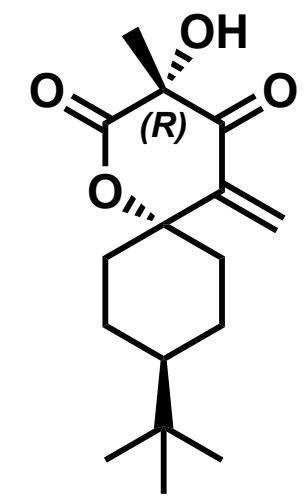

AOG_4

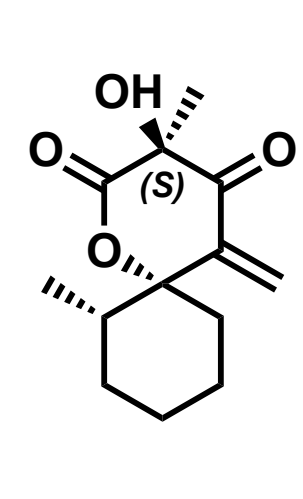

AOG_5

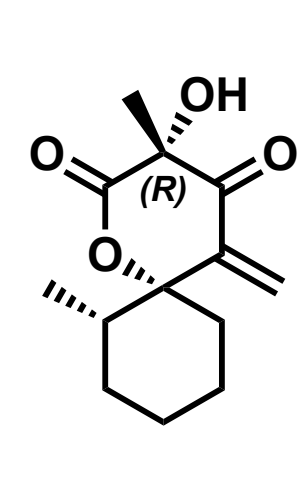

AOG_6

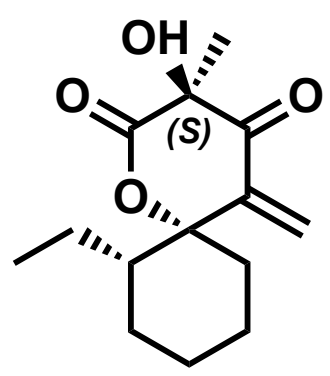

AOG_7

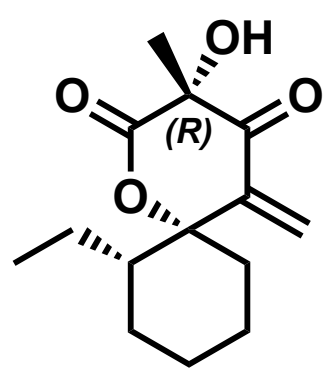

AOG_8

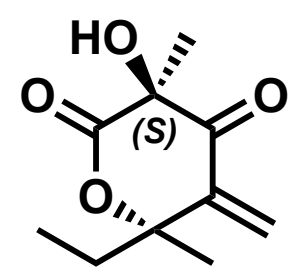

AOG_9

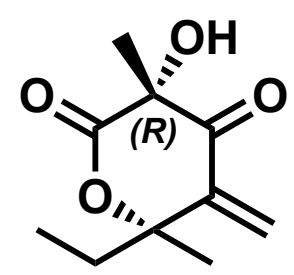

AOG_10

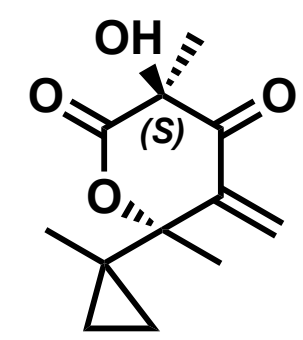

AOG_11

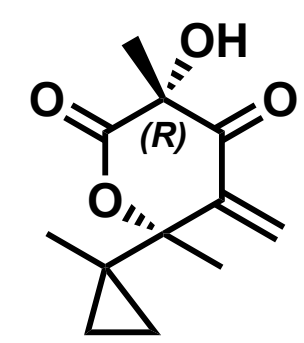

AOG_12

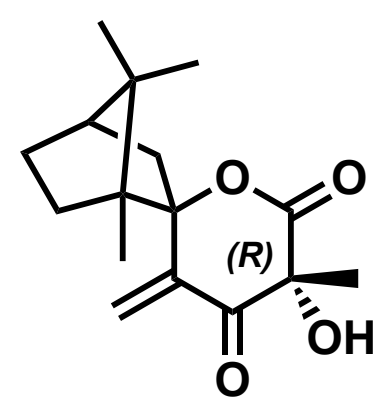

AOG_13

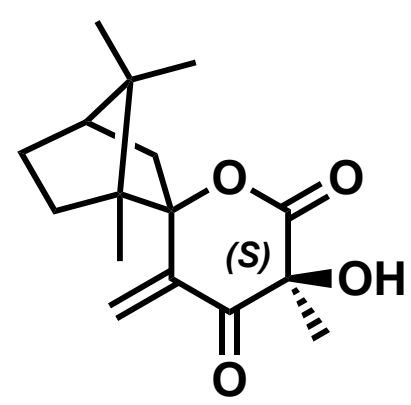

AOG_14

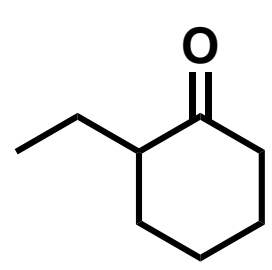

ARL_1

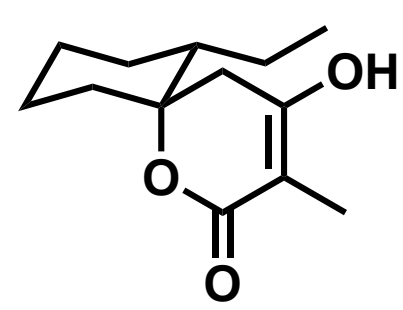

ARL_2

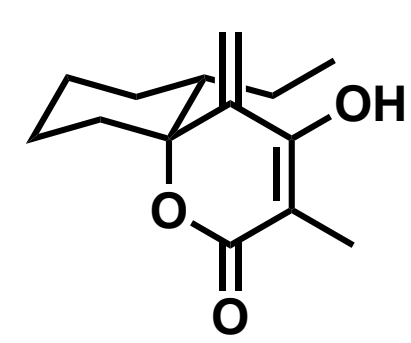

ARL_3

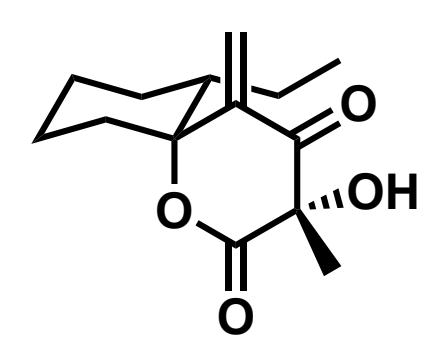

ARL_4

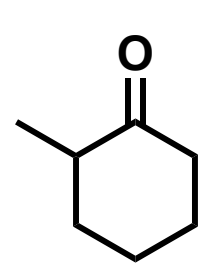

Gu_1

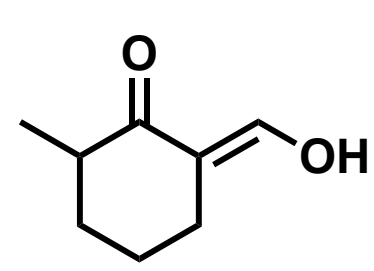

Gu_2

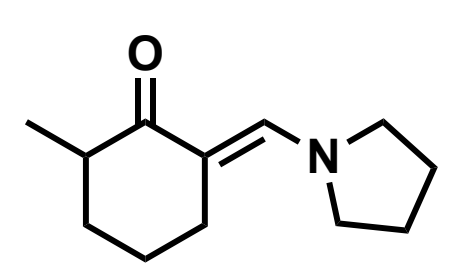

Gu_3

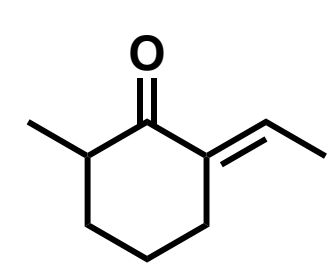

Gu_4

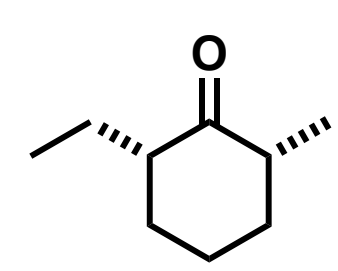

Gu_5

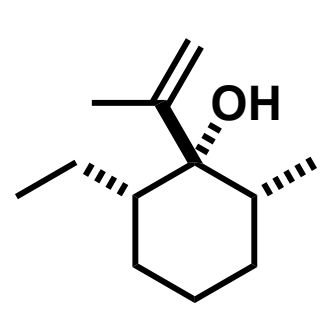

Gu_6

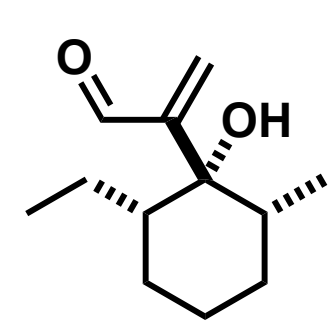

Gu_7

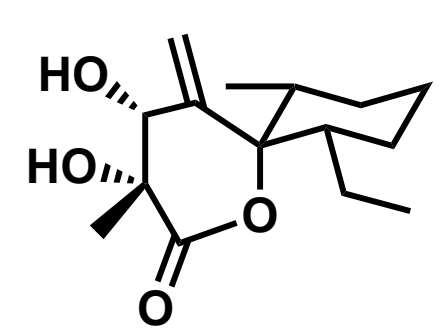

Gu_8

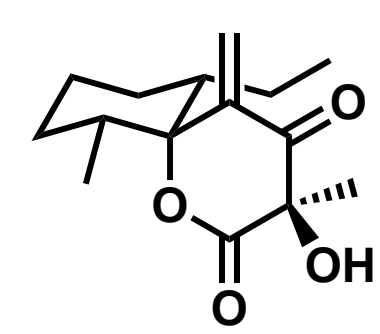

Aogacillin B
Gu-9

Code: [6]

```
import numpy as np

import matplotlib.pyplot as plt

from mpl_toolkits.mplot3d import Axes3D


fig = plt.figure()

plt.rcParams.update({'font.size': 10})

ax = fig.add_subplot(111, projection='3d')


p = np.array([15.68]); q = np.array([0.58]); r = np.array([0.90])

ax.scatter(p, q, r, s=40, label='AOG A', marker='o', c='firebrick', alpha=1.0)


s = np.array([15.58]); t = np.array([0.54]); u = np.array([0.94])

ax.scatter(s, t, u, s=40, label='AOG-Et', marker='o', c='navy', alpha=1.0)


d = np.array([16.14]); e = np.array([0.45]); f = np.array([0.90])

ax.scatter(d, e, f, s=40, label='AOG-Me', marker='o', c='g', alpha=1.0)


g = np.array([15.72]); h = np.array([0.42]); i = np.array([0.85])

ax.scatter(g, h, i, s=40, label='AOG-CAMP', marker='o', c='m', alpha=1.0)


j = np.array([18.06]); k = np.array([0.36]); l = np.array([0.96])

ax.scatter(j, k, l, s=40, label='AOG-me-Dimer', marker='o', c='red', alpha=1.0)


j = np.array([17.59]); k = np.array([0.44]); l = np.array([0.95])

ax.scatter(j, k, l, s=40, label='AOG-prop-Dimer', marker='o', c='black', alpha=1.0)
```

```
l = np.array([12.62, 16.14, 15.58, 16.92, 16.92, 15.55, 15.55, 15.72])
m = np.array([0.41, 0.47, 0.50, 0.62, 0.56, 0.47, 0.43, 0.43])
n = np.array([0.82, 0.88, 0.88, 0.86, 0.83, 0.88, 0.86, 0.86])
ax.scatter(l, m, n, s=25, marker='o', c='gray', alpha=1.0)

# red series
x = np.array([9.83, 10.42, 9.47, 9.38, 10.22, 12.09, 12.62, 16.57, 15.68])
y = np.array([0.45, 0.33, 0.19, 0.34, 0.34, 0.59, 0.60, 0.49, 0.59])
z = np.array([0.63, 0.71, 0.91, 0.72, 0.72, 0.67, 0.69, 0.93, 0.89])
ax.scatter(x, y, z, s=10, marker='o', c='firebrick', alpha=1.0)
ax.plot(x, y, z, linewidth=1, c='firebrick', linestyle='--')

# Black series
a = np.array([9.41, 13.26, 13.01, 15.58])
b = np.array([0.38, 0.44, 0.52, 0.54])
c = np.array([0.69, 0.87, 0.86, 0.94])
ax.scatter(a, b, c, s=10, marker='o', c='navy', alpha=1.0)
ax.plot(a, b, c, linewidth=1.0, c='navy')

from mpl_toolkits.mplot3d.art3d import Poly3DCollection

# Define box corners
x_min, x_max = 15, 16.5
y_min, y_max = 0.4, 0.55
z_min, z_max = 0.85, 0.95

verts = [
  # bottom
```

```
    [(x_min,y_min,z_min),(x_max,y_min,z_min),(x_max,y_max,z_min),(x_min,y_max,z_min)],
    # top
    [(x_min,y_min,z_max),(x_max,y_min,z_max),(x_max,y_max,z_max),(x_min,y_max,z_max)],
    # sides
    [(x_min,y_min,z_min),(x_max,y_min,z_min),(x_max,y_min,z_max),(x_min,y_min,z_max)],
    [(x_max,y_min,z_min),(x_max,y_max,z_min),(x_max,y_max,z_max),(x_max,y_min,z_max)],
    [(x_max,y_max,z_min),(x_min,y_max,z_min),(x_min,y_max,z_max),(x_max,y_max,z_max)],
    [(x_min,y_max,z_min),(x_min,y_min,z_min),(x_min,y_min,z_max),(x_min,y_max,z_max)]
]

box = Poly3DCollection(verts, alpha=0.15, facecolor='green')
ax.add_collection3d(box)


x_min, x_max = 17.5, 18.5
y_min, y_max = 0.33, 0.47
z_min, z_max = 0.93, 0.97
verts = [
    # bottom
    [(x_min,y_min,z_min),(x_max,y_min,z_min),(x_max,y_max,z_min),(x_min,y_max,z_min)],
    # top
    [(x_min,y_min,z_max),(x_max,y_min,z_max),(x_max,y_max,z_max),(x_min,y_max,z_max)],
    # sides
    [(x_min,y_min,z_min),(x_max,y_min,z_min),(x_max,y_min,z_max),(x_min,y_min,z_max)],
    [(x_max,y_min,z_min),(x_max,y_max,z_min),(x_max,y_max,z_max),(x_max,y_min,z_max)],
    [(x_max,y_max,z_min),(x_min,y_max,z_min),(x_min,y_max,z_max),(x_max,y_max,z_max)],
    [(x_min,y_max,z_min),(x_min,y_min,z_min),(x_min,y_min,z_max),(x_min,y_max,z_max)]
]
box = Poly3DCollection(verts, alpha=0.15, facecolor='blue')
```

```
ax.add_collection3d(box)

ax.set_xlabel('Cm/atom')
ax.set_ylabel('I1/3')
ax.set_zlabel('I2/I3')
ax.set_xlim([8, 18])
ax.set_ylim([0, 1])
ax.set_zlim([0.5, 1])

ax.legend(loc=2, prop={'size': 6})

axes = plt.gca()
axes.xaxis.label.set_size(10)
axes.yaxis.label.set_size(10)
axes.zaxis.label.set_size(10)

ax.xaxis.set_pane_color((1.0, 1.0, 1.0, 0.0))
ax.yaxis.set_pane_color((1.0, 1.0, 1.0, 0.0))
ax.zaxis.set_pane_color((1.0, 1.0, 1.0, 0.0))

# View from a fixed angle — edit these to change the perspective
ax.view_init(elev=20, azim=250)

plt.tight_layout()
plt.show()
```

F. Spectra

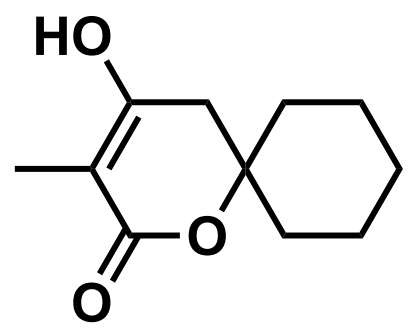

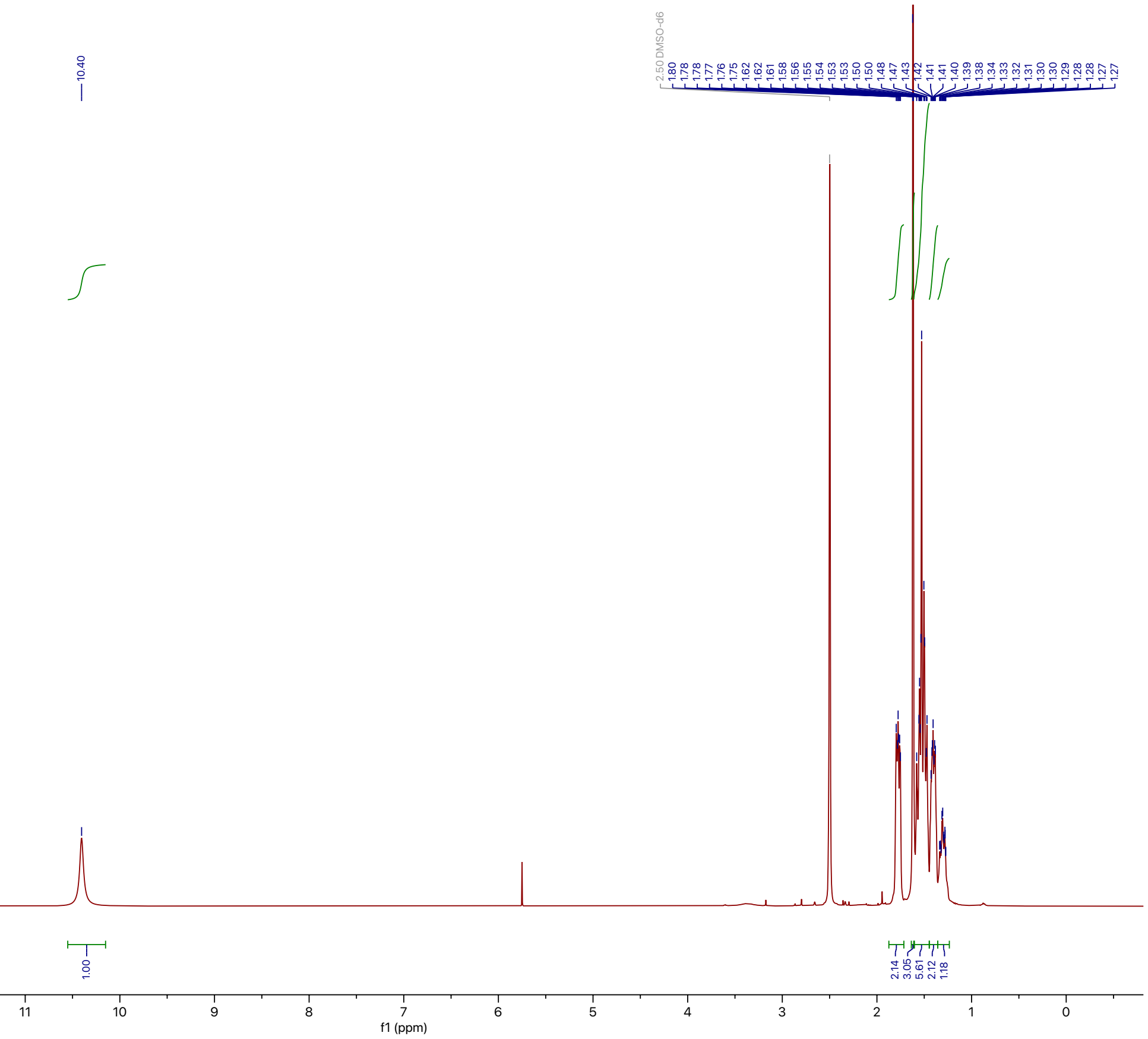

^{1}H NMR (400 MHz, DMSO)

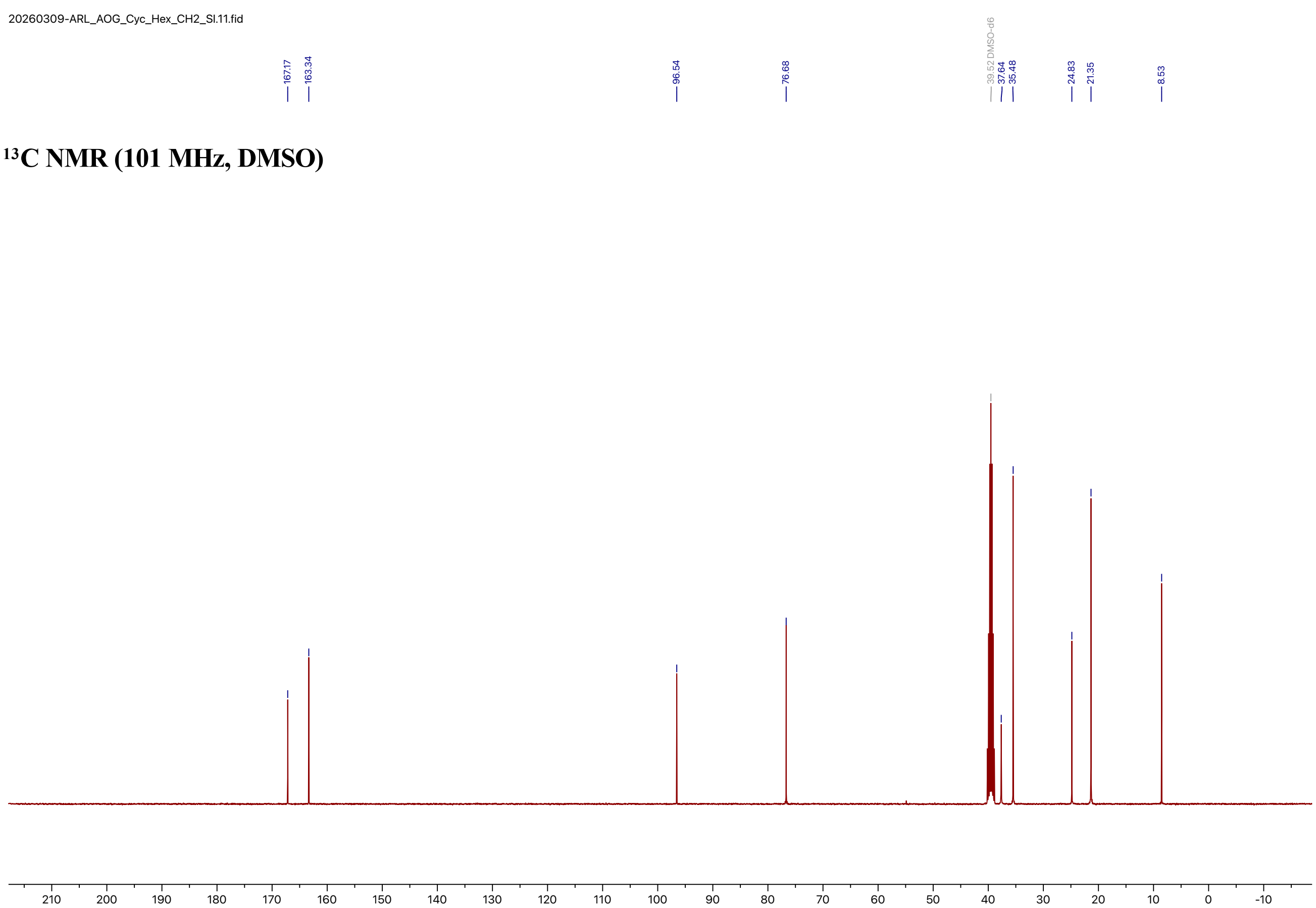

^{13}C NMR (101 MHz, DMSO)

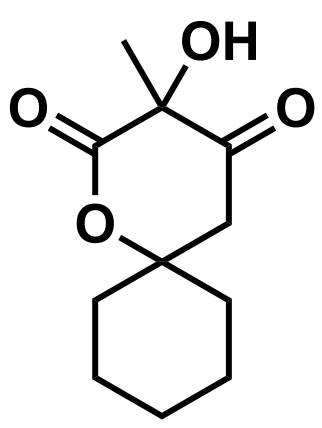

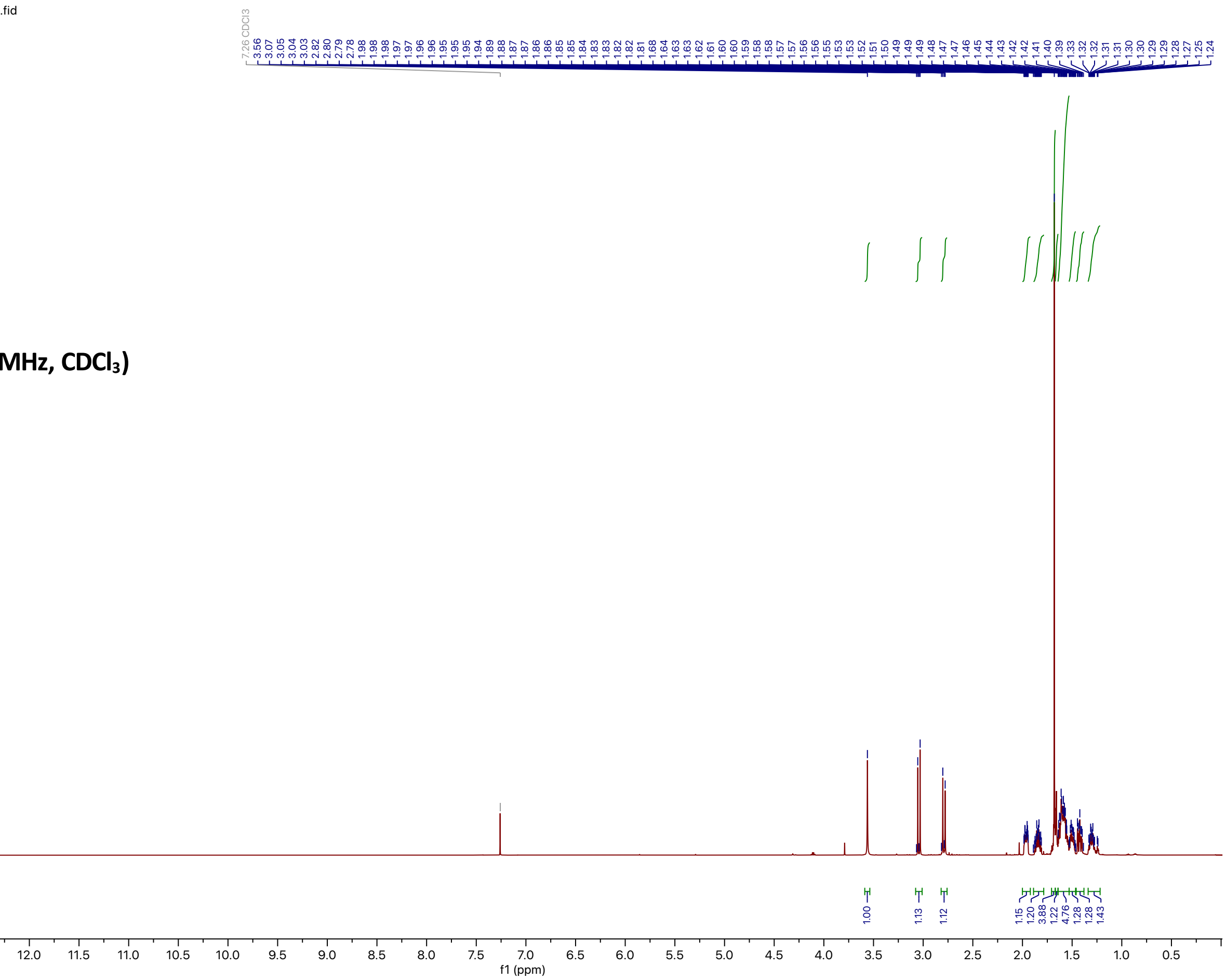

^{1}H NMR (600 MHz, $CDCl_3$)

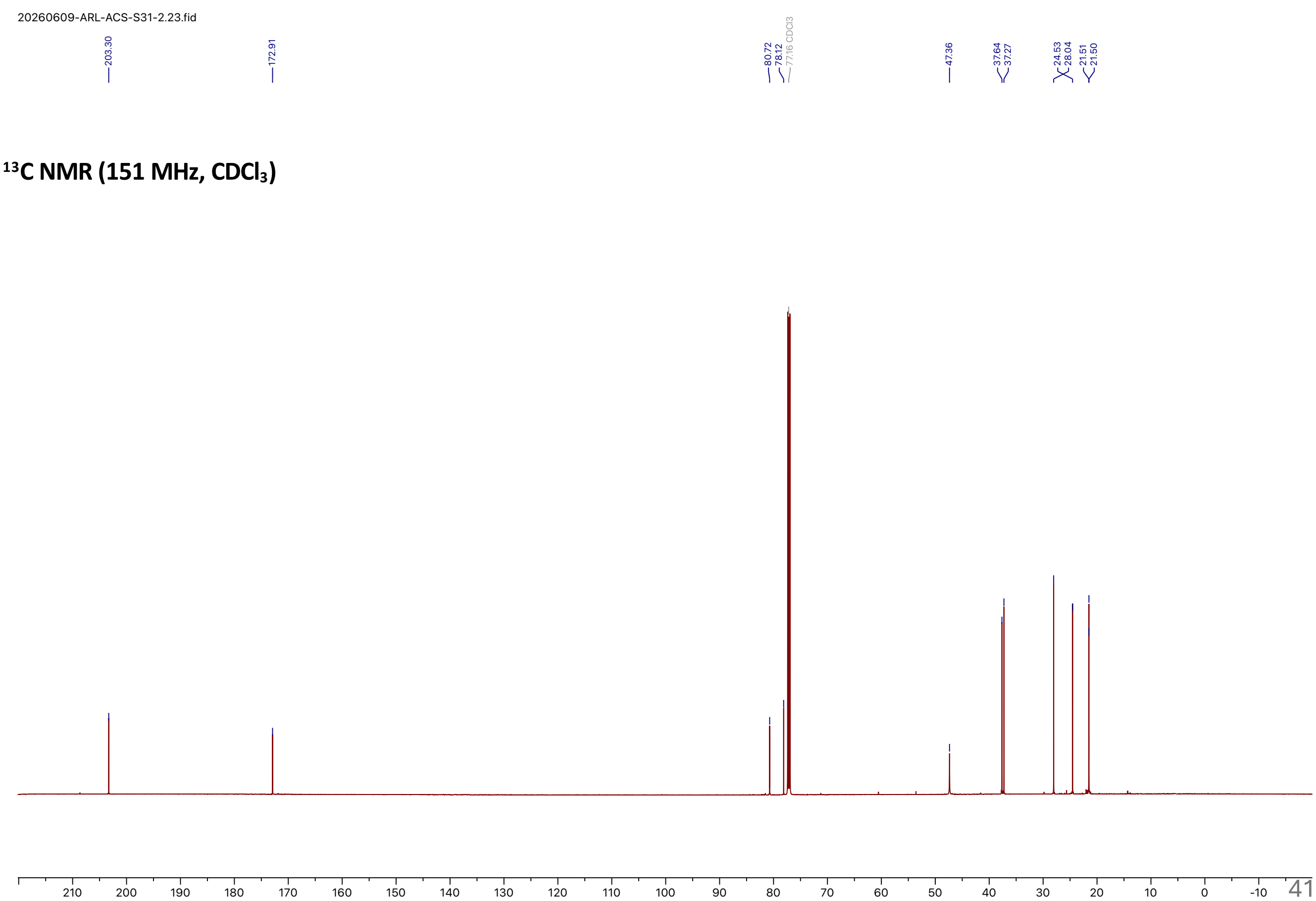

^{13}C NMR (151 MHz, $CDCl_3$)

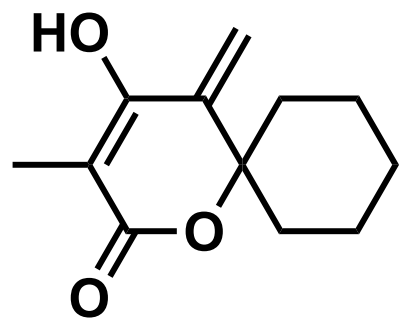

^{1}H NMR (400 MHz, $CDCl_3$),

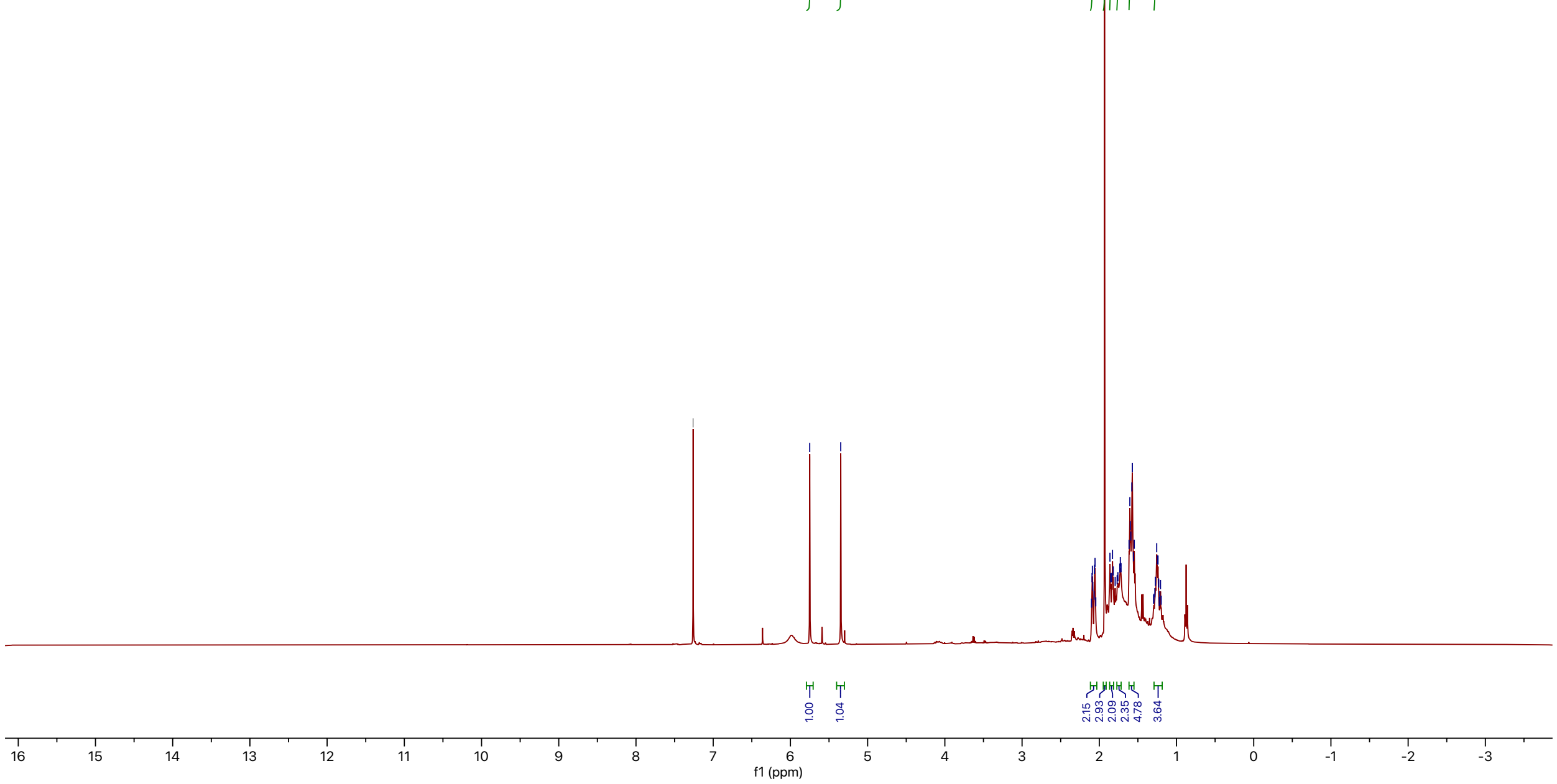

^{13}C NMR (101 MHz, $CDCl_3$)

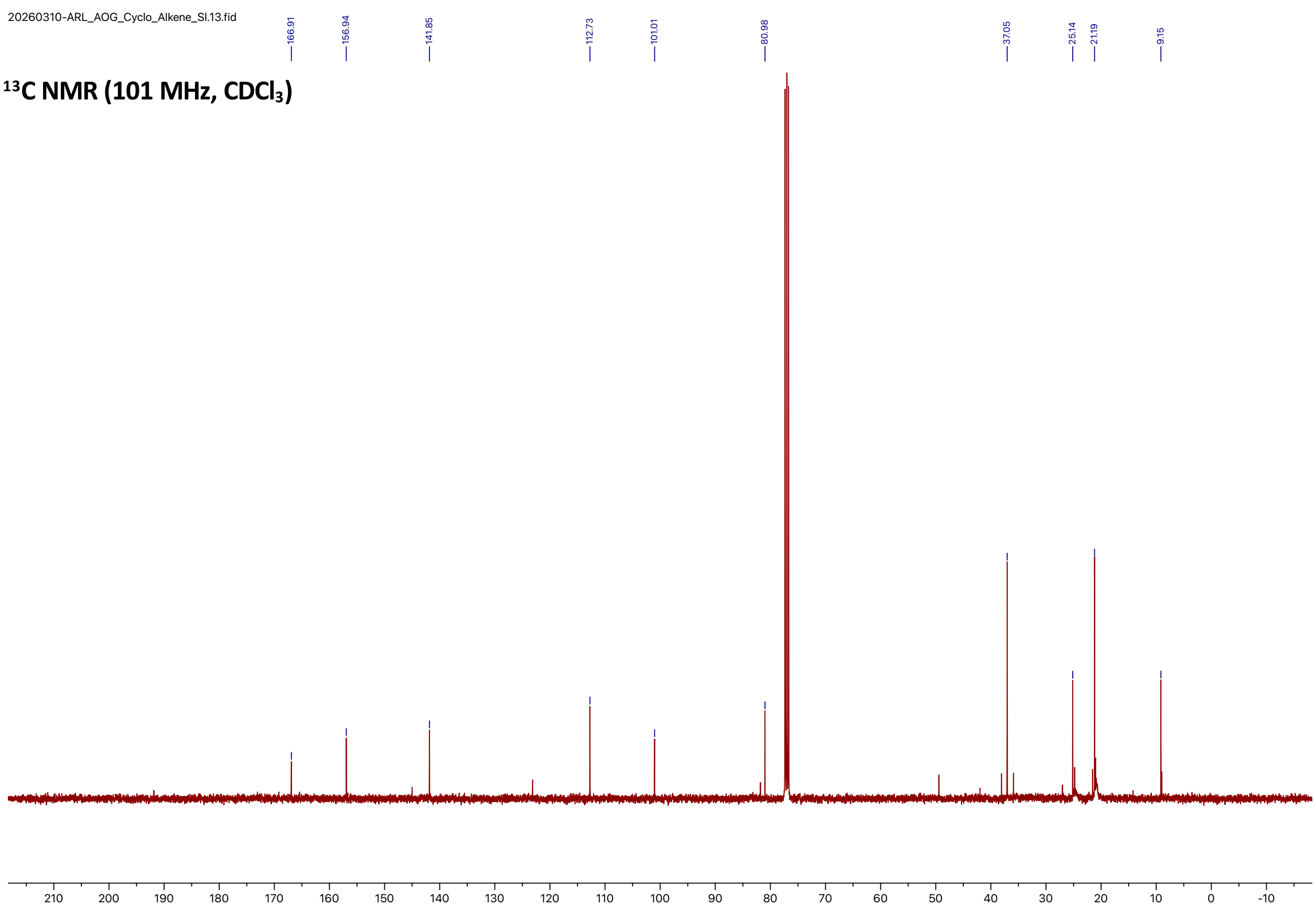

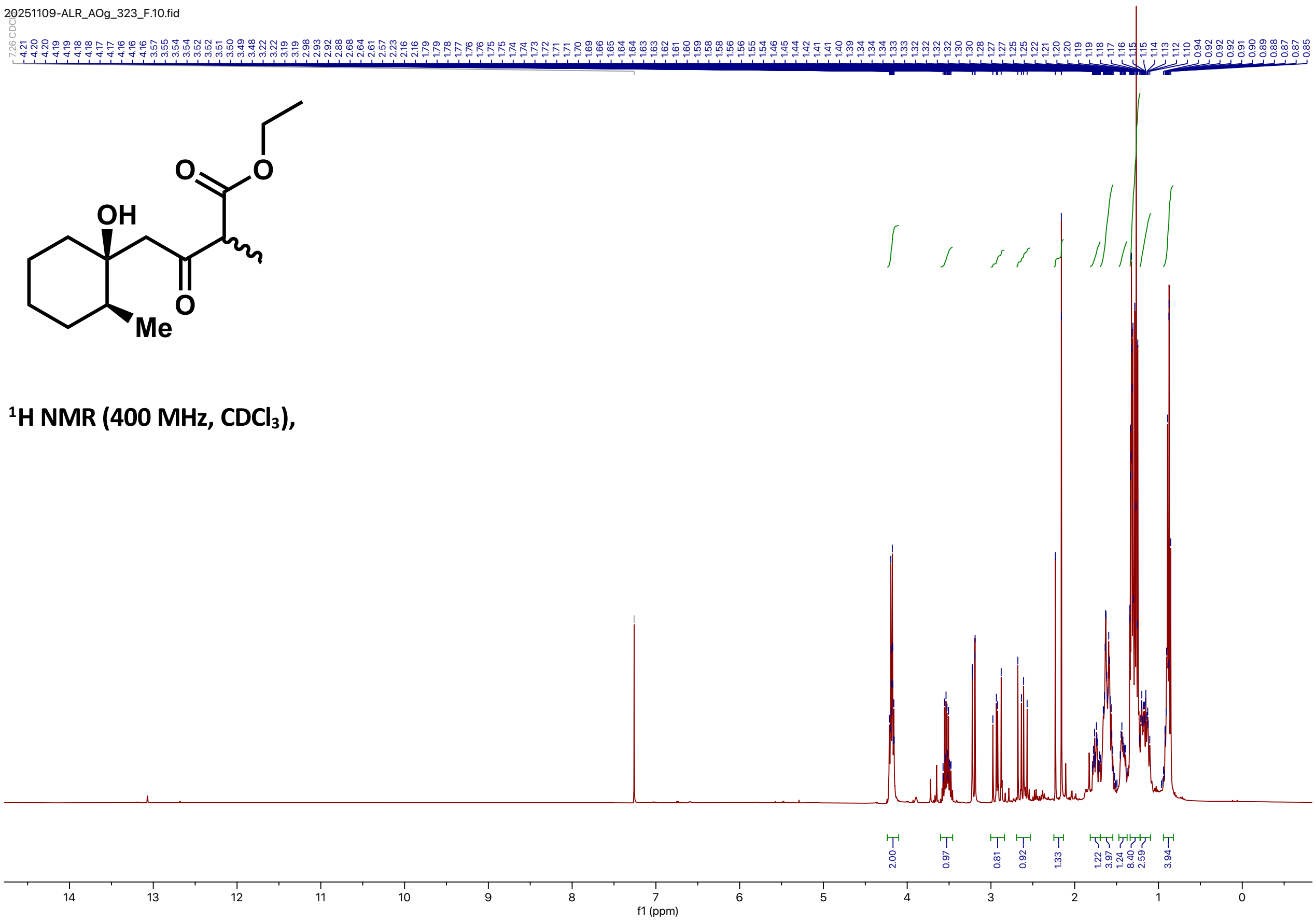
20251109-ALR_AOg_323_F.10.fid
OH
O
O
O
Me
1H NMR (400 MHz, CDCl3),
2.00
0.97
0.81
0.92
1.33
1.22
3.97
1.24
8.40
2.59
3.94
f1 (ppm)
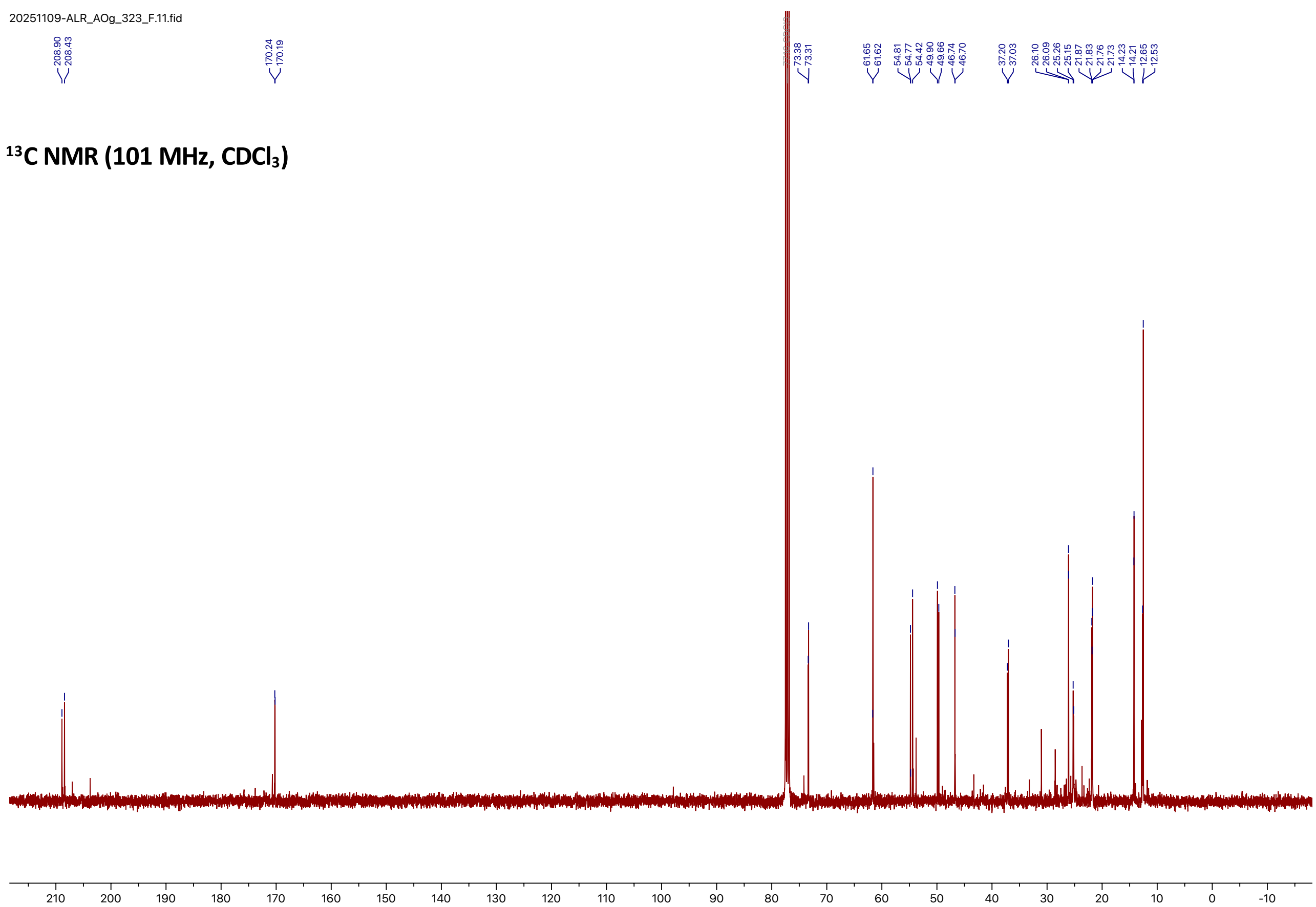
20251109-ALR_AOg_323_F.11.fid
208.90
208.43
170.24
170.19
73.38
73.31
61.65
61.62
54.81
54.77
54.42
49.90
49.66
46.74
46.70
37.20
37.03
26.10
26.09
25.26
25.15
21.87
21.83
21.76
21.73
14.23
14.21
12.65
12.53
13C NMR (101 MHz, CDCl3)
f1 (ppm)

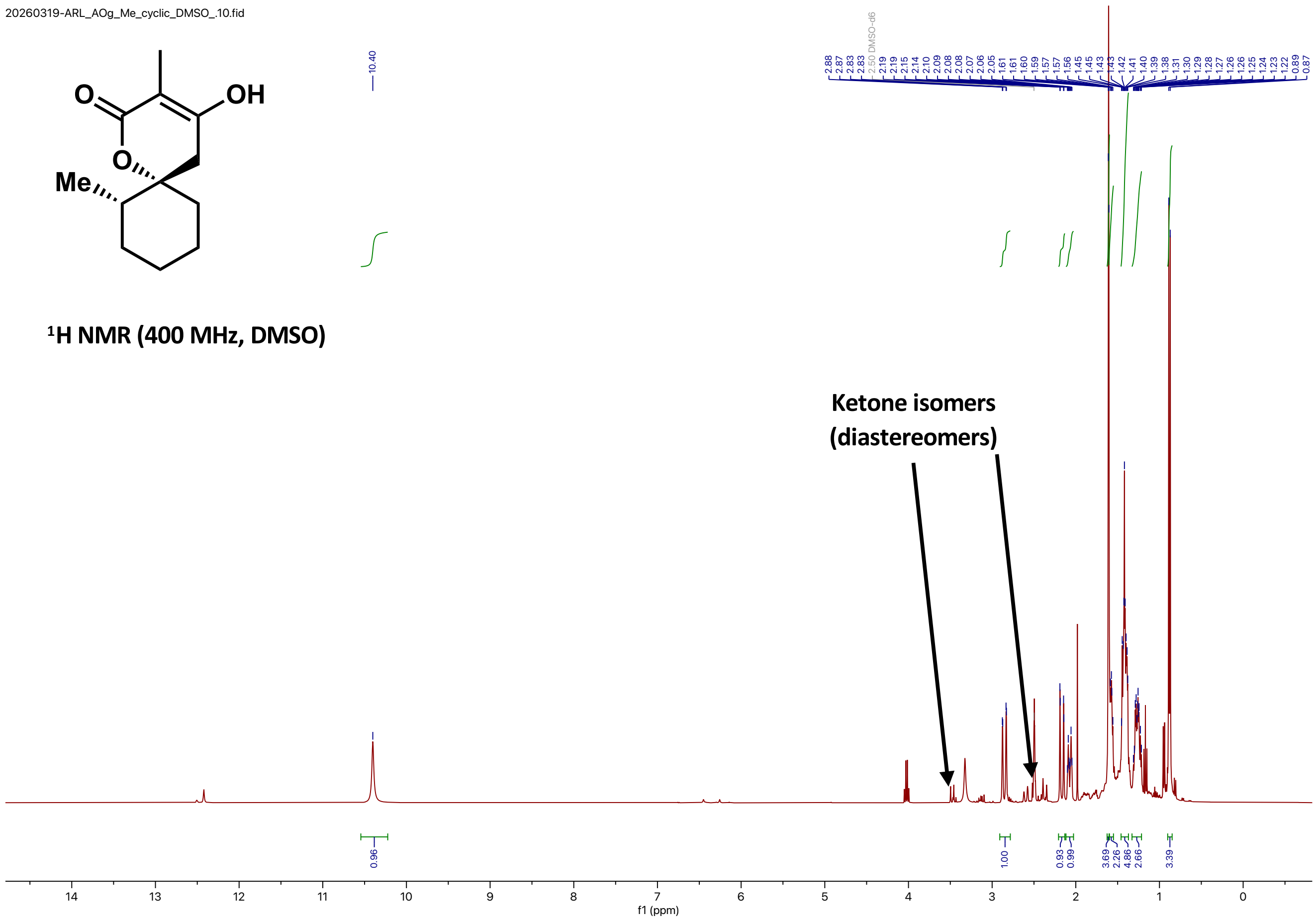

¹H NMR (400 MHz, DMSO)

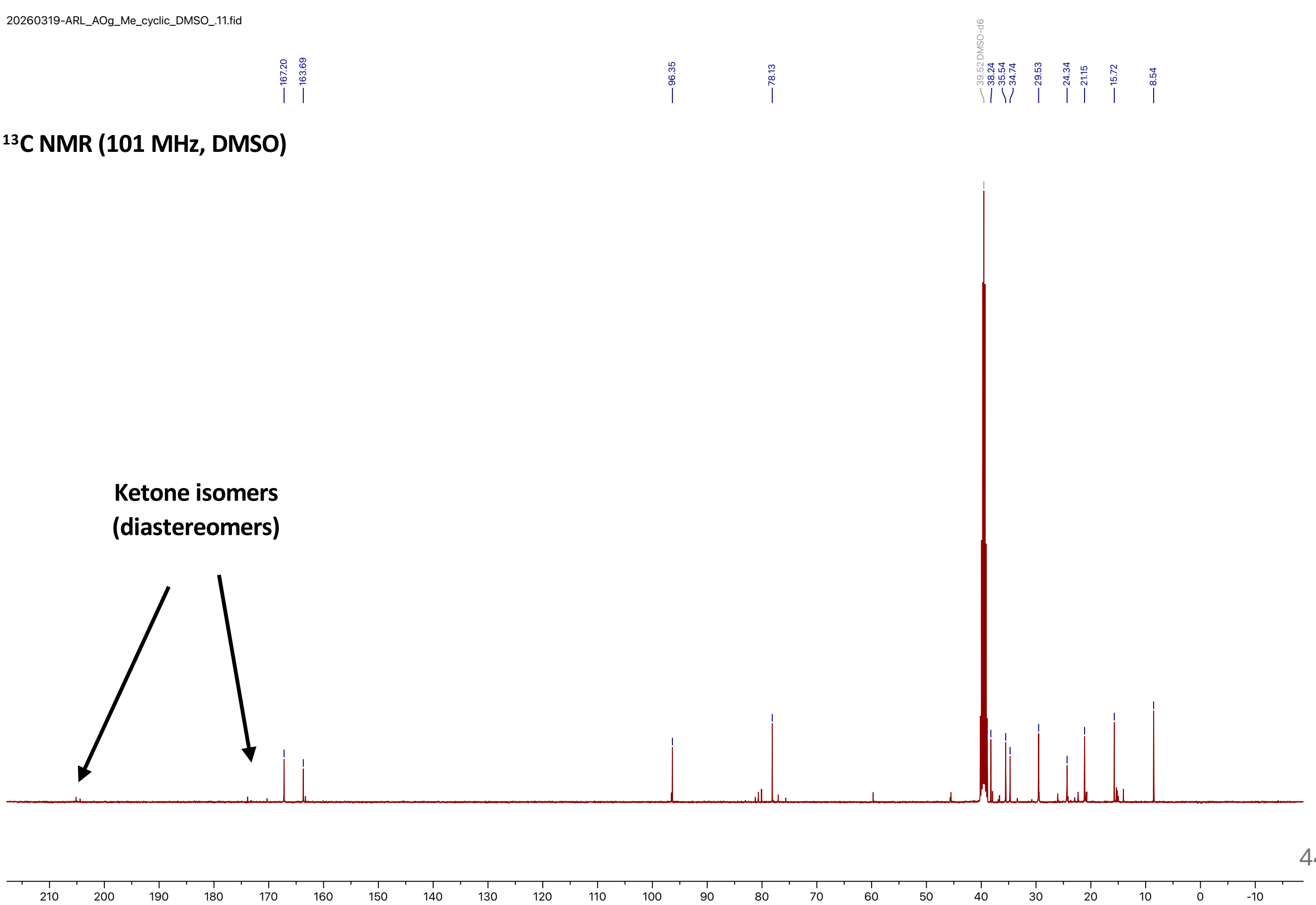

¹³C NMR (101 MHz, DMSO)

HMBC, ^{1}H NMR (400 MHz, DMSO), ^{13}C NMR (101 MHz, DMSO)

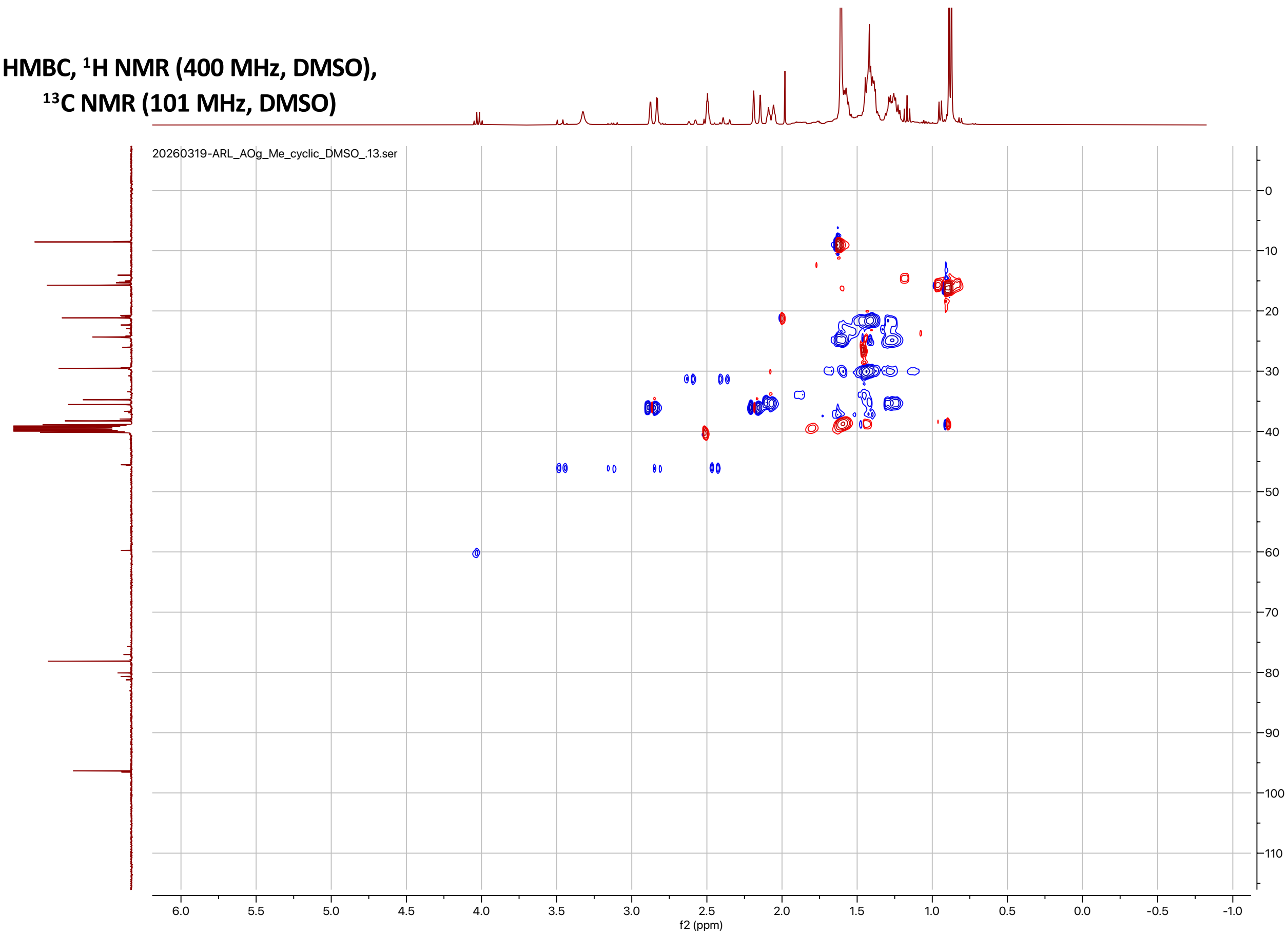

20251213-ARL_AOG_372_1.1.fid

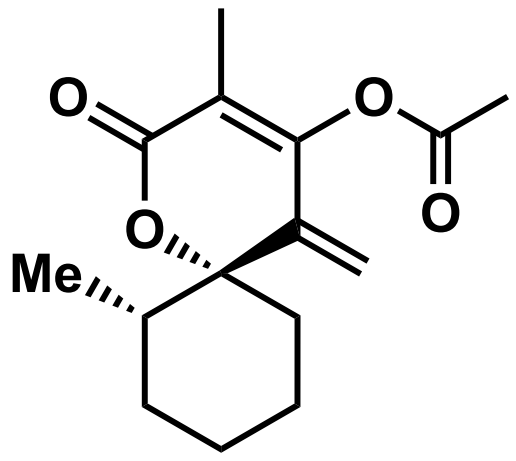

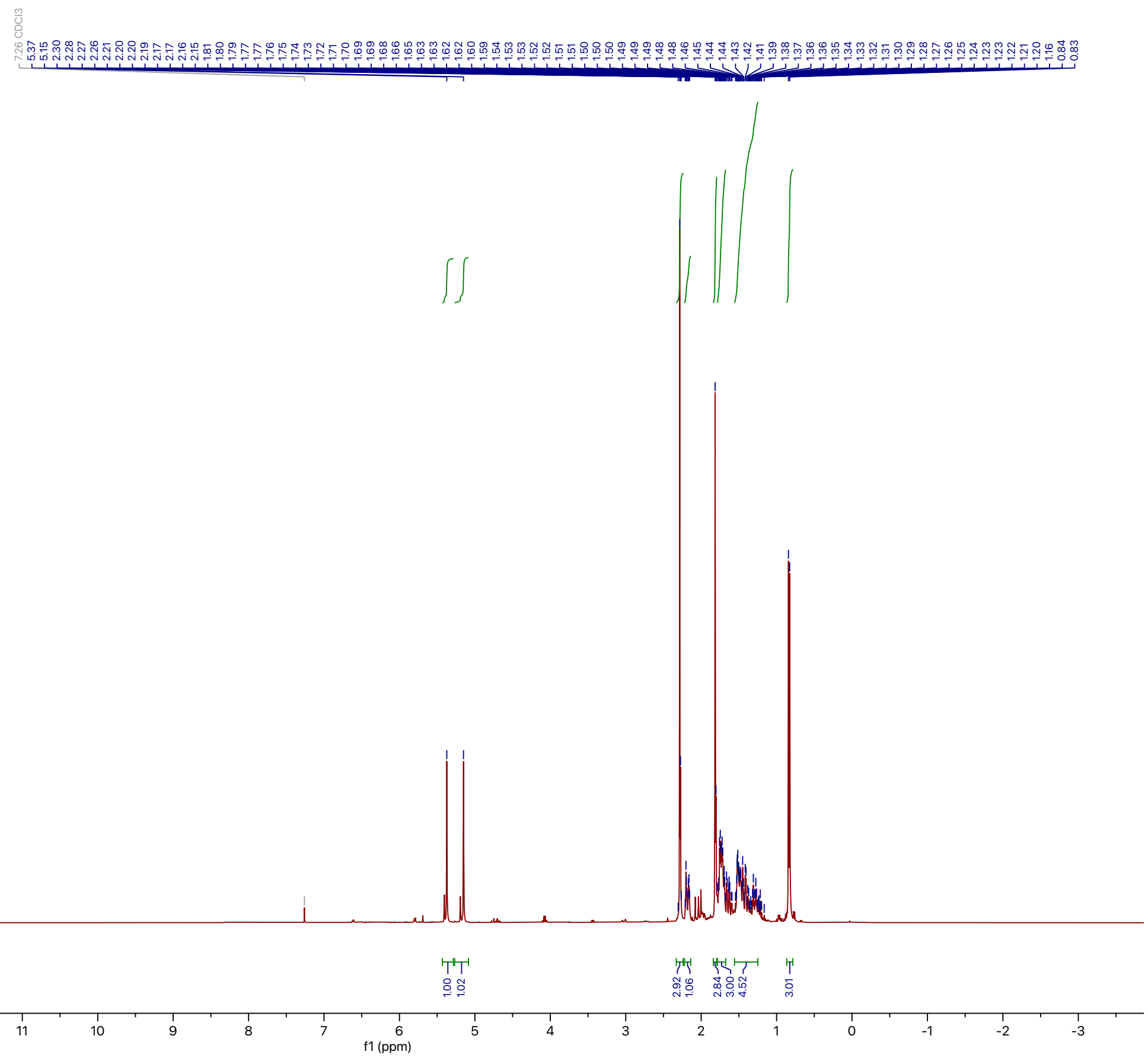

^{1}H NMR (400 MHz, $CDCl_3$),

20251213-ARL_AOG_372_1.2.fid

^{13}C NMR (101 MHz, $CDCl_3$)

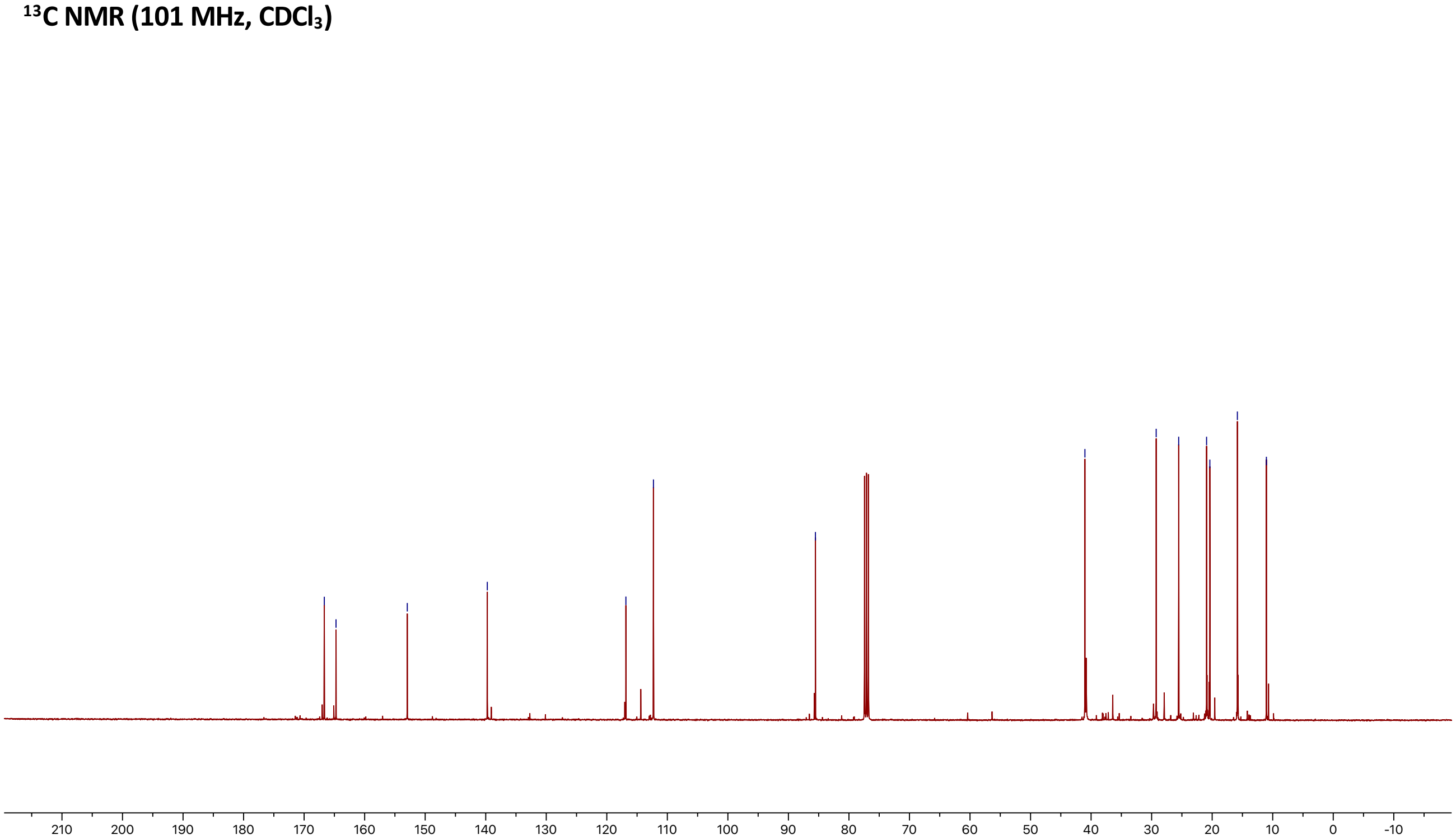

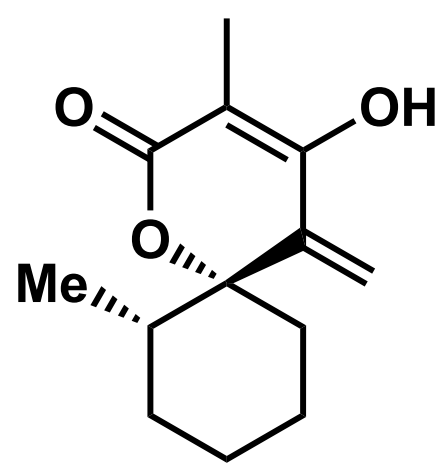

¹H NMR (400 MHz, CDCl₃),

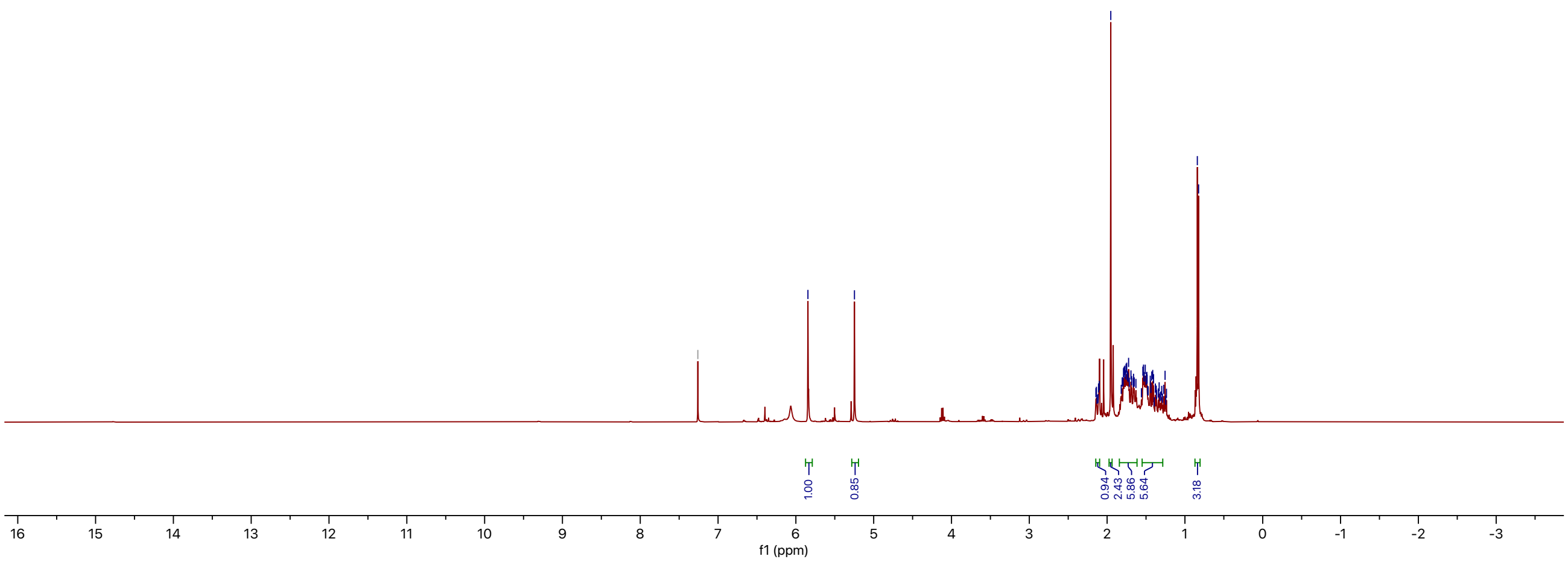

¹³C NMR (101 MHz, CDCl₃)

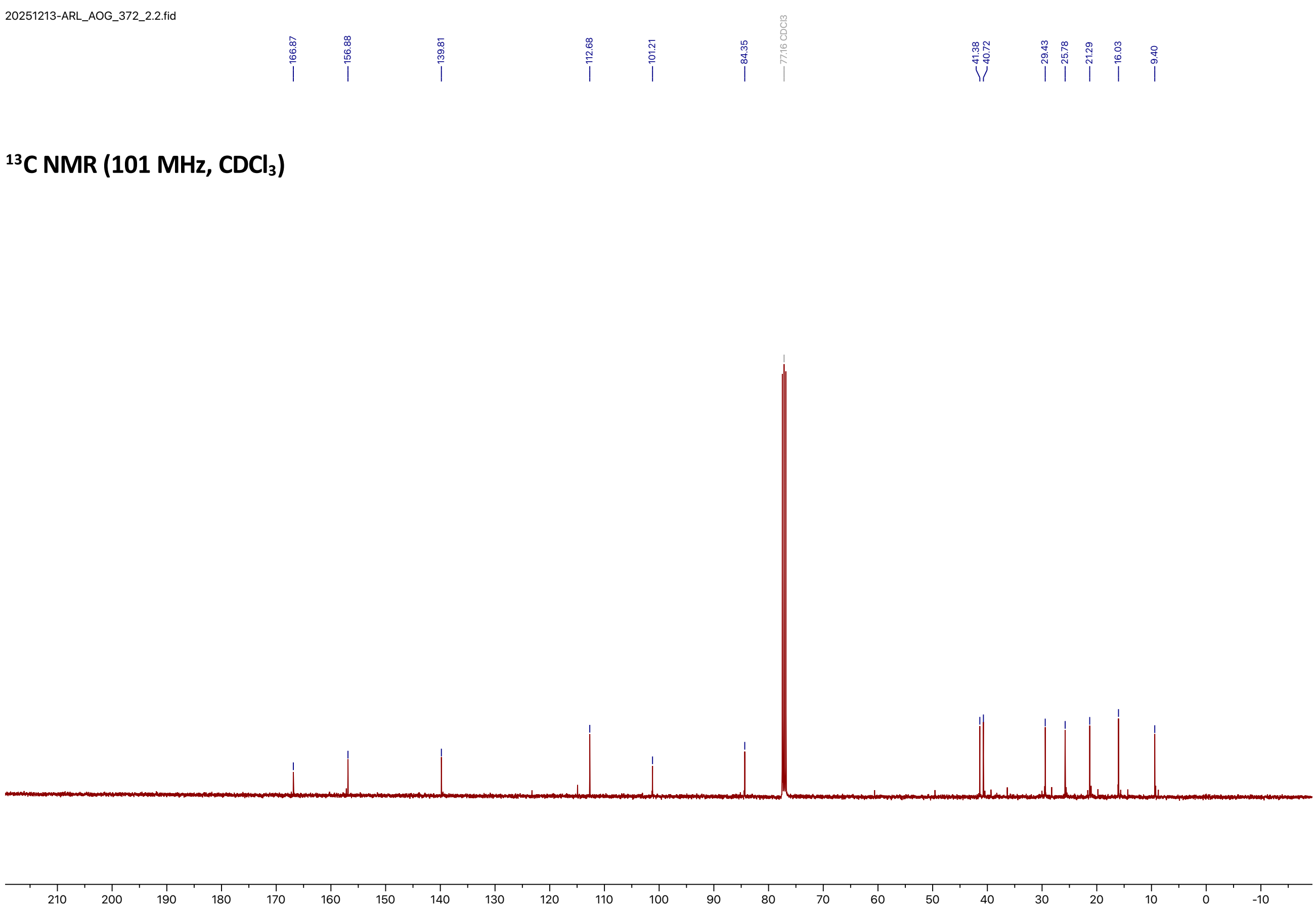

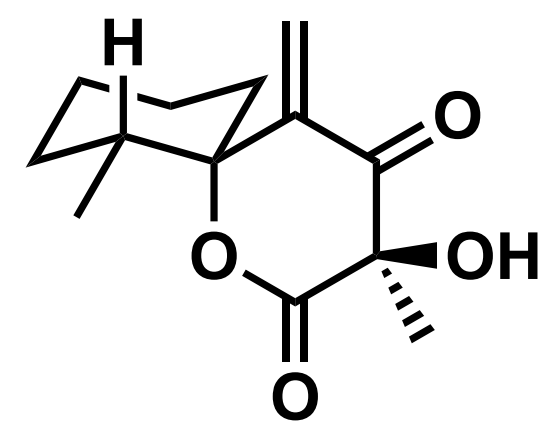

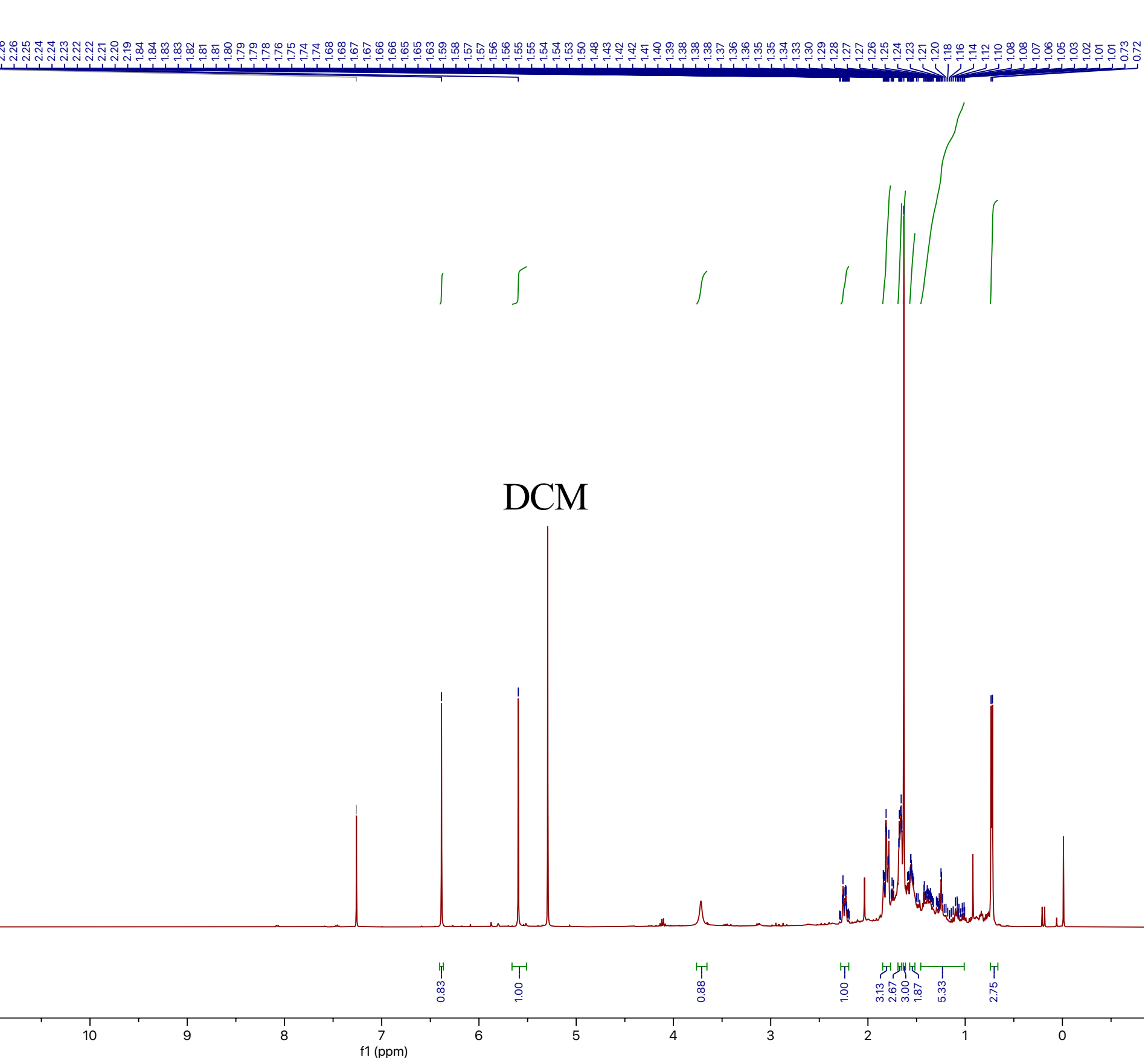

^{1}H NMR (400 MHz, $CDCl_3$),

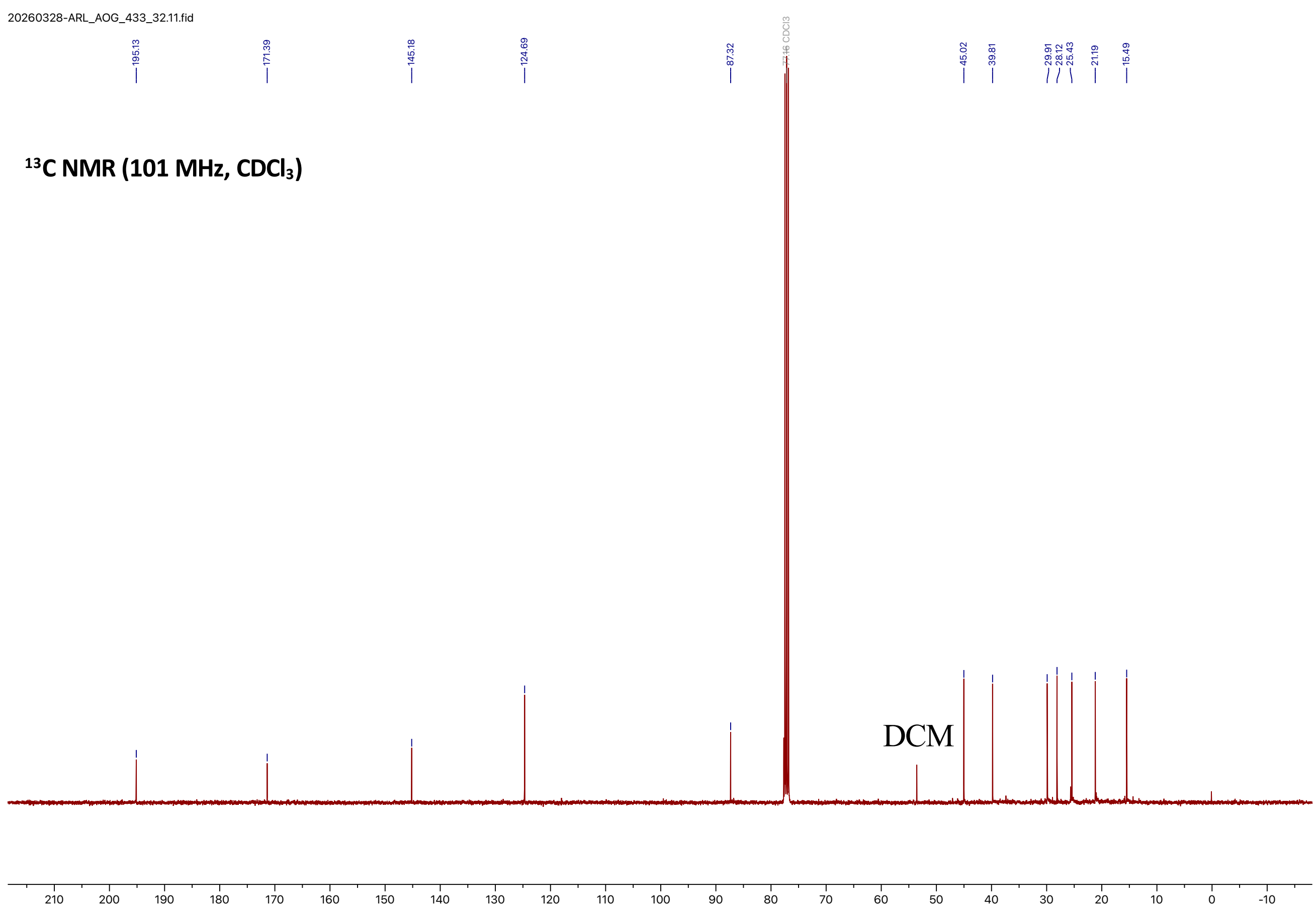

^{13}C NMR (101 MHz, $CDCl_3$)

^{1}H NMR (400 MHz, $CDCl_3$) δ 2.25, 1.92, 1.81, 1.67, 0.74

^{1}H NMR (400 MHz, $CDCl_3$) δ 2.27, 1.82, 1.67, 1.66, 0.73.

COSEY, ^{1}H NMR (400 MHz, $CDCl_3$), ^{1}H NMR (400 MHz, $CDCl_3$),

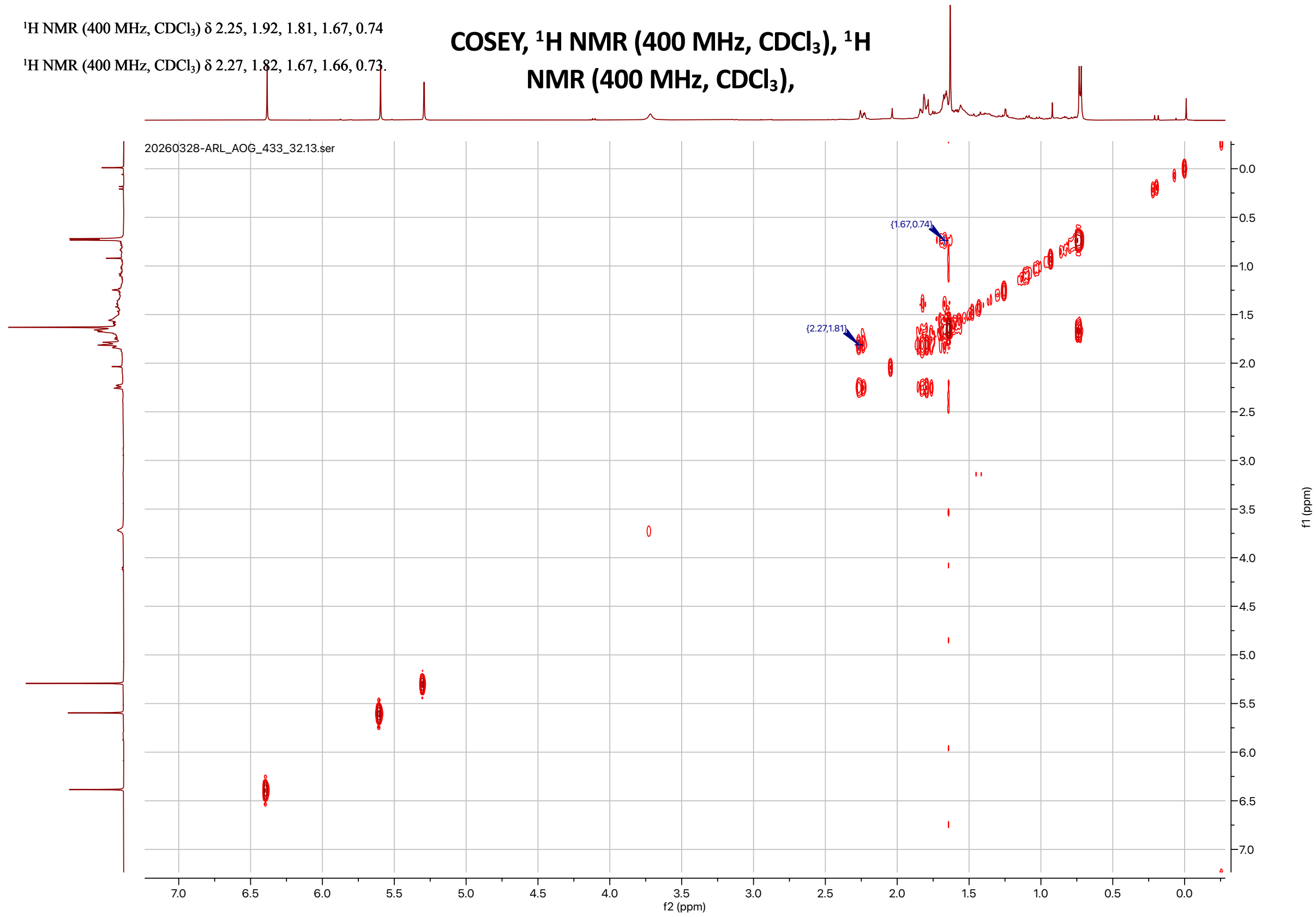

HMBC, ^{1}H NMR (400 MHz, $CDCl_3$), ^{13}C NMR (101 MHz, $CDCl_3$)

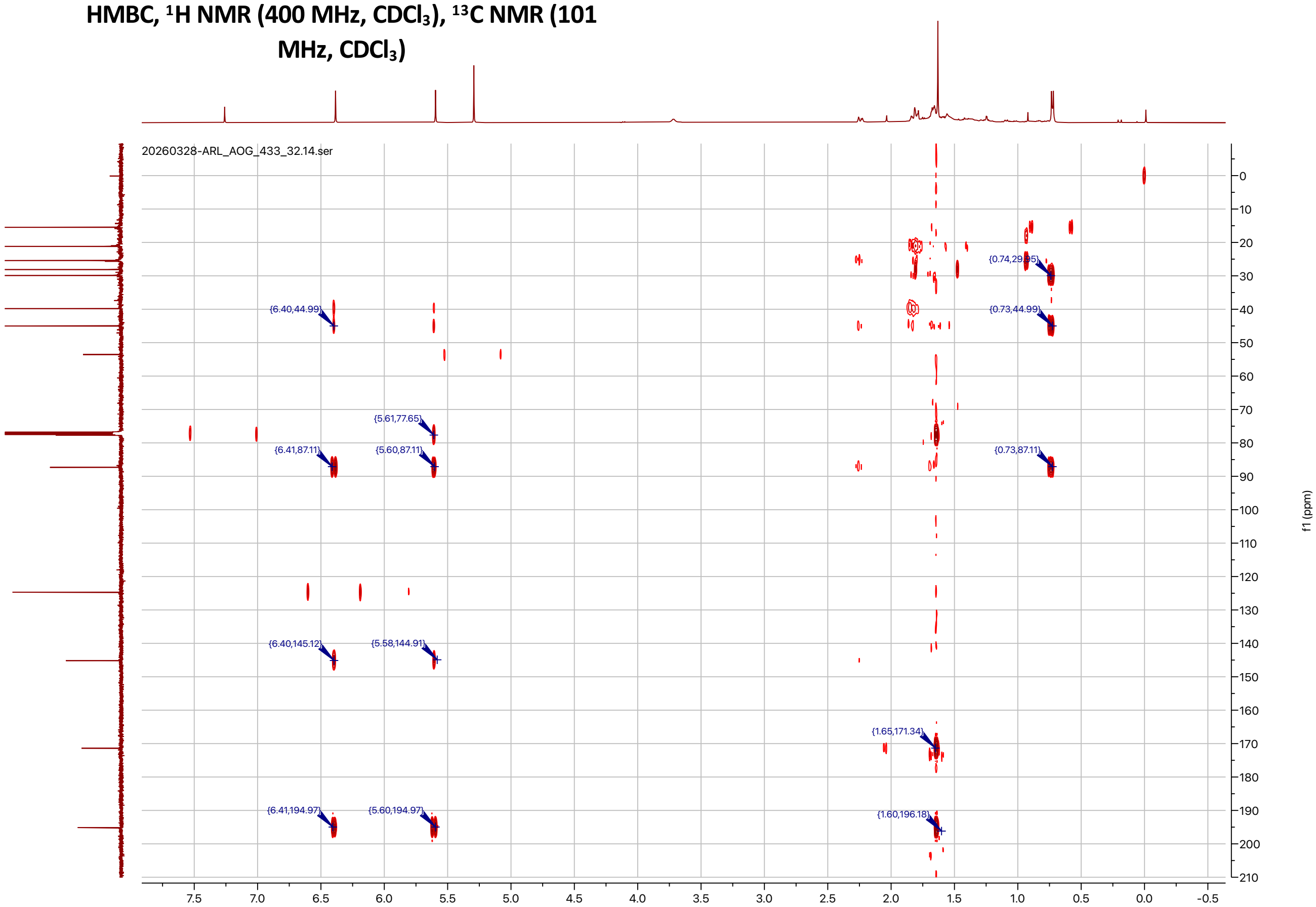

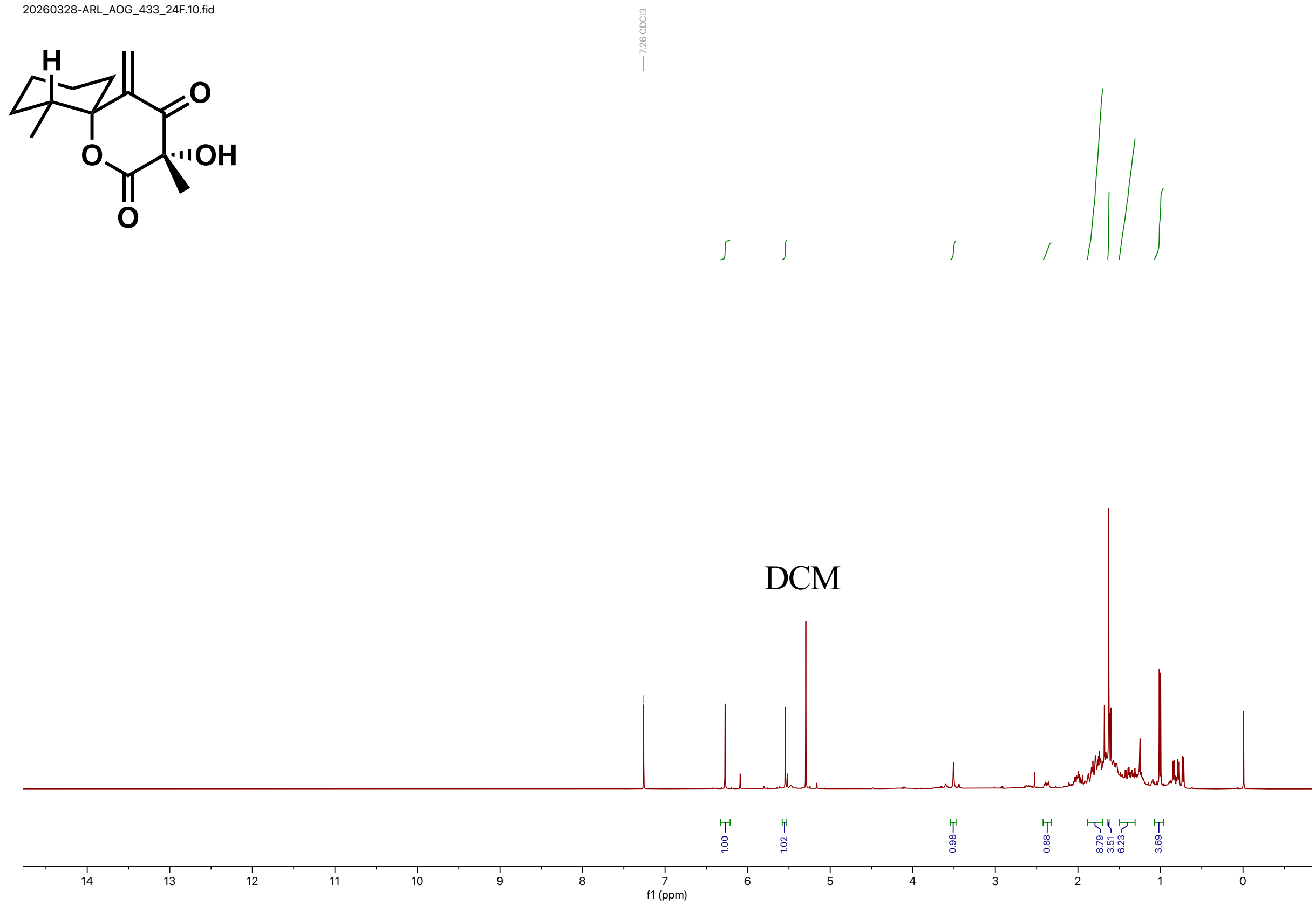
20260328-ARL_AOG_433_24F.10.fid
7.26 CDCl3
DCM
1.00
1.02
0.98
0.88
8.79
3.51
6.23
3.69
f1 (ppm)

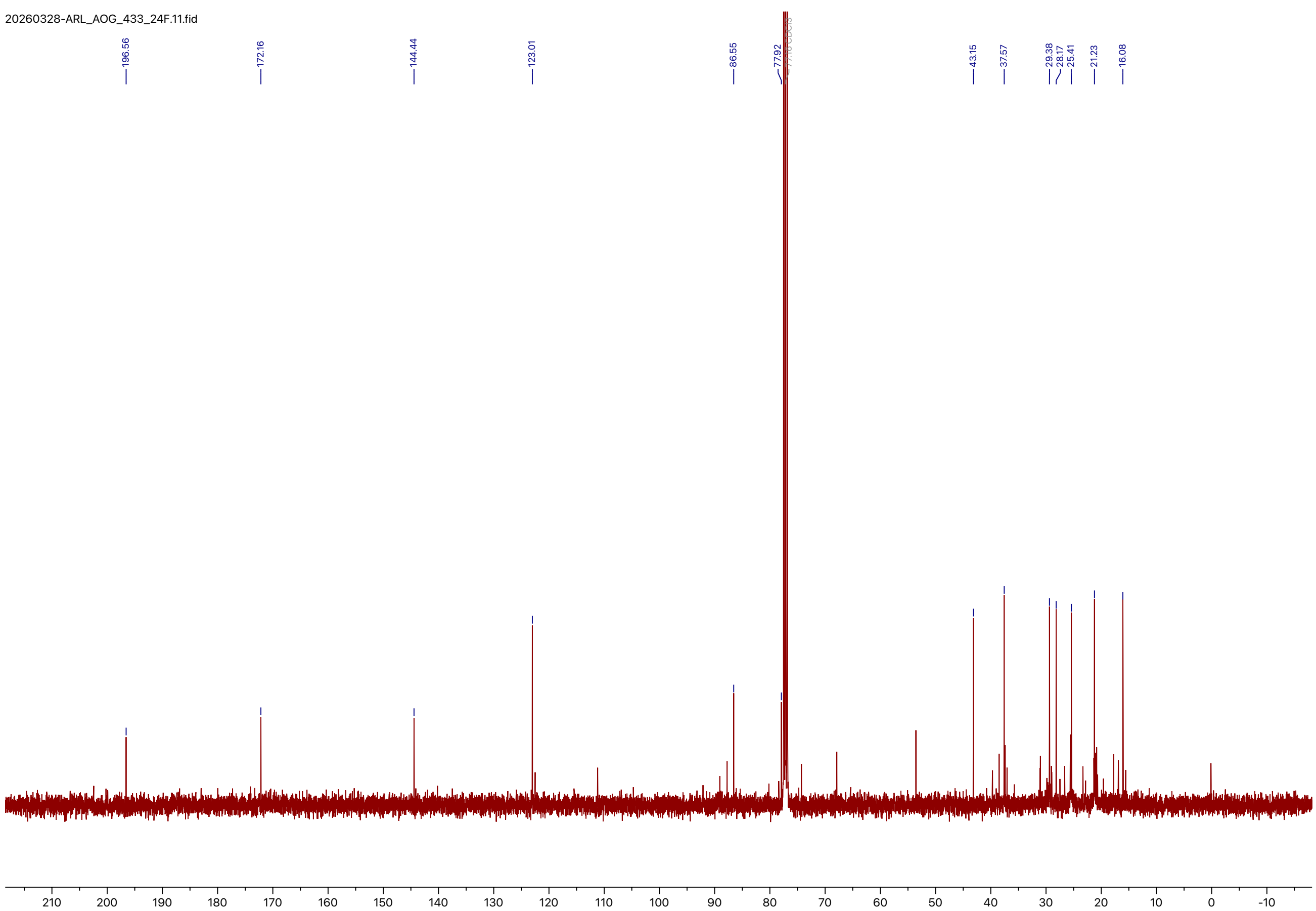
20260328-ARL_AOG_433_24F.11.fid
196.56
172.16
144.44
123.01
86.55
77.92
43.15
37.57
29.38
28.17
25.41
21.23
16.08
f1 (ppm)

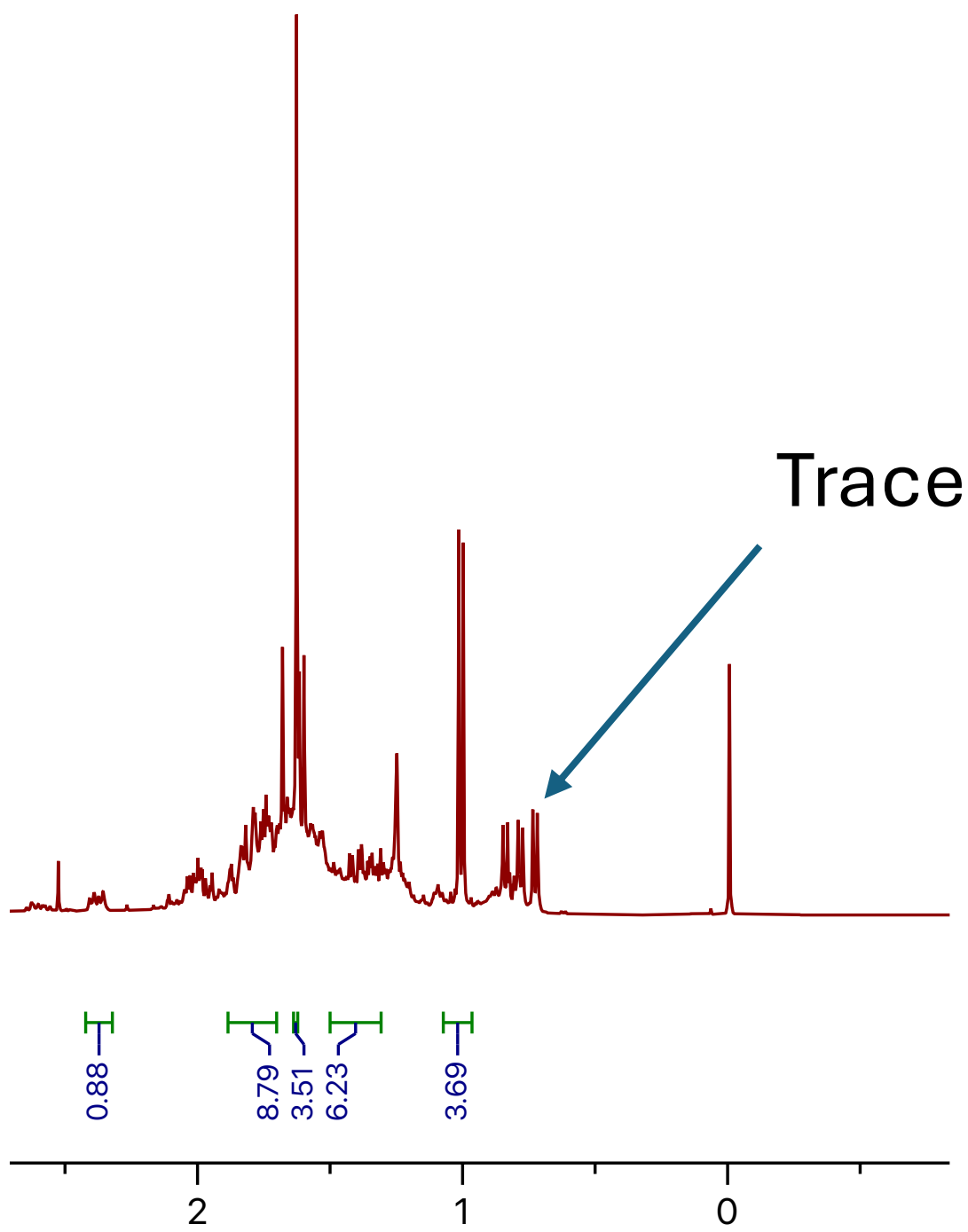

Trace dimerization in $CDCl_3$

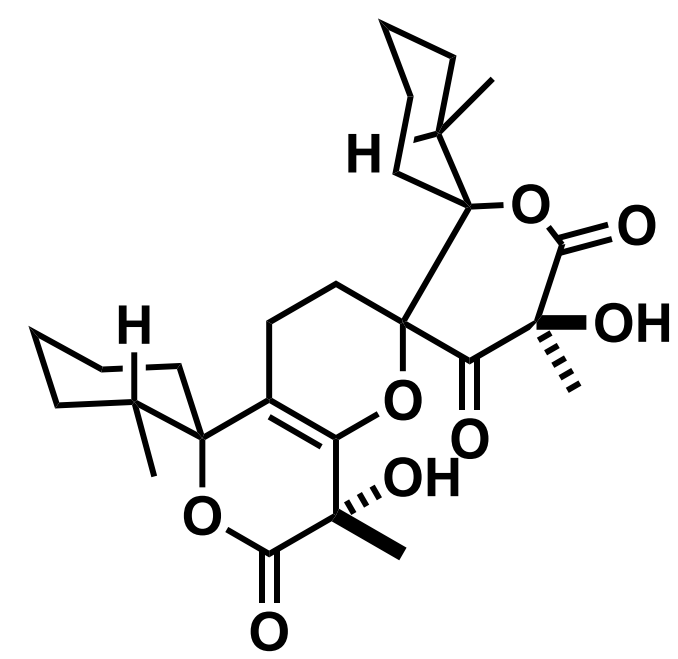

^{1}H NMR (400 MHz, $CDCl_3$),

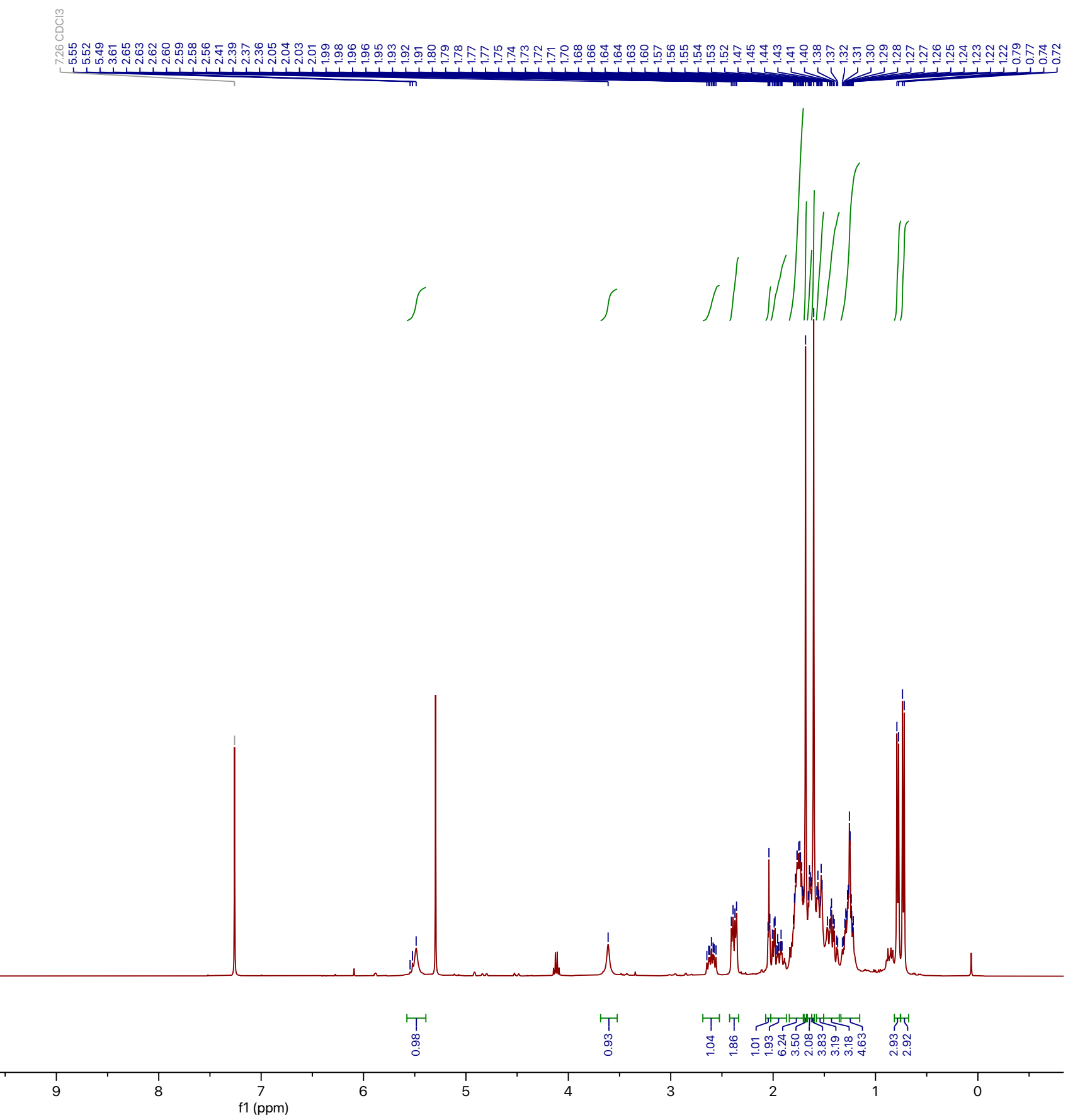

^{13}C NMR (101 MHz, $CDCl_3$)

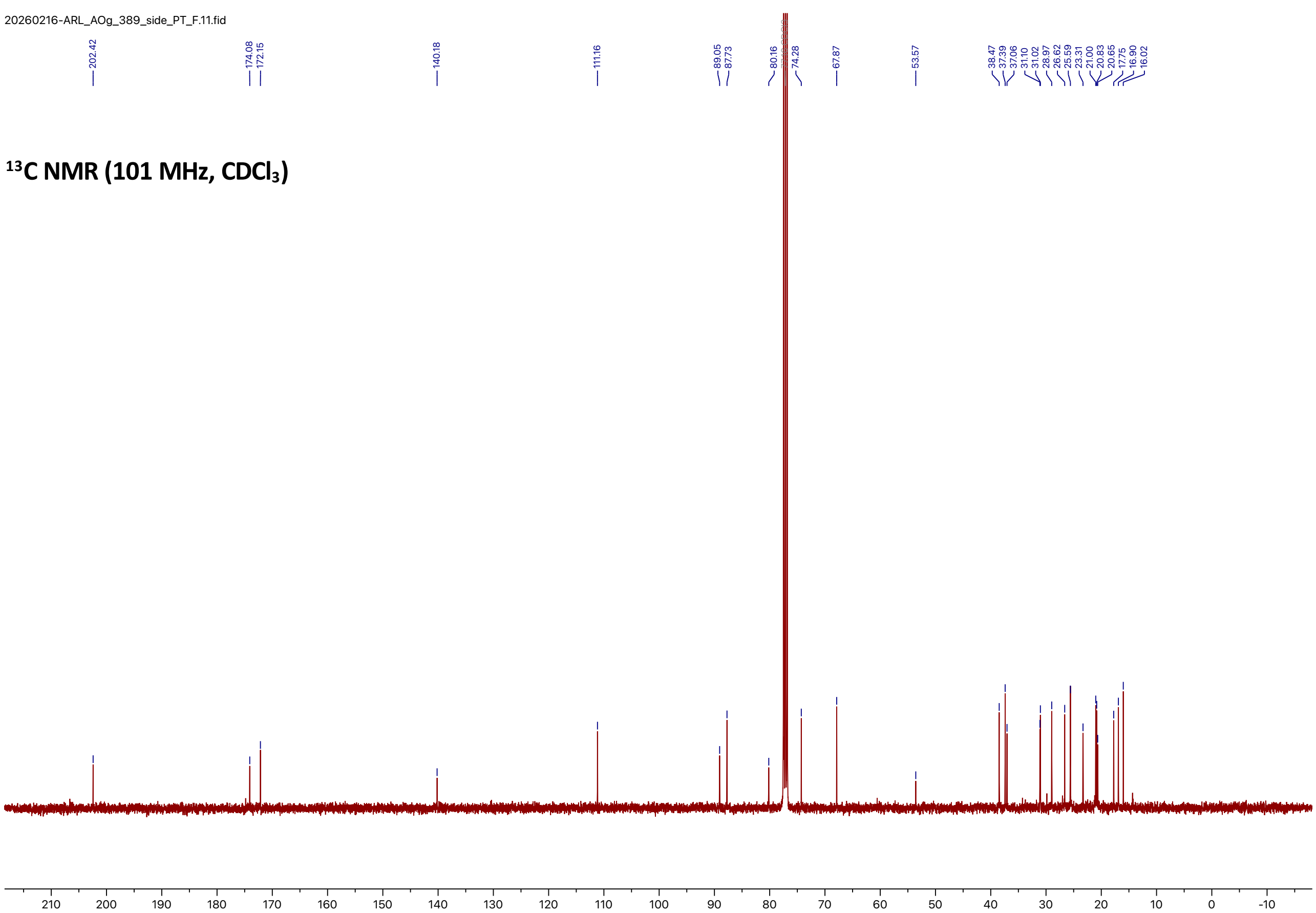

COSY, ^{1}H NMR (400 MHz, $CDCl_3$)

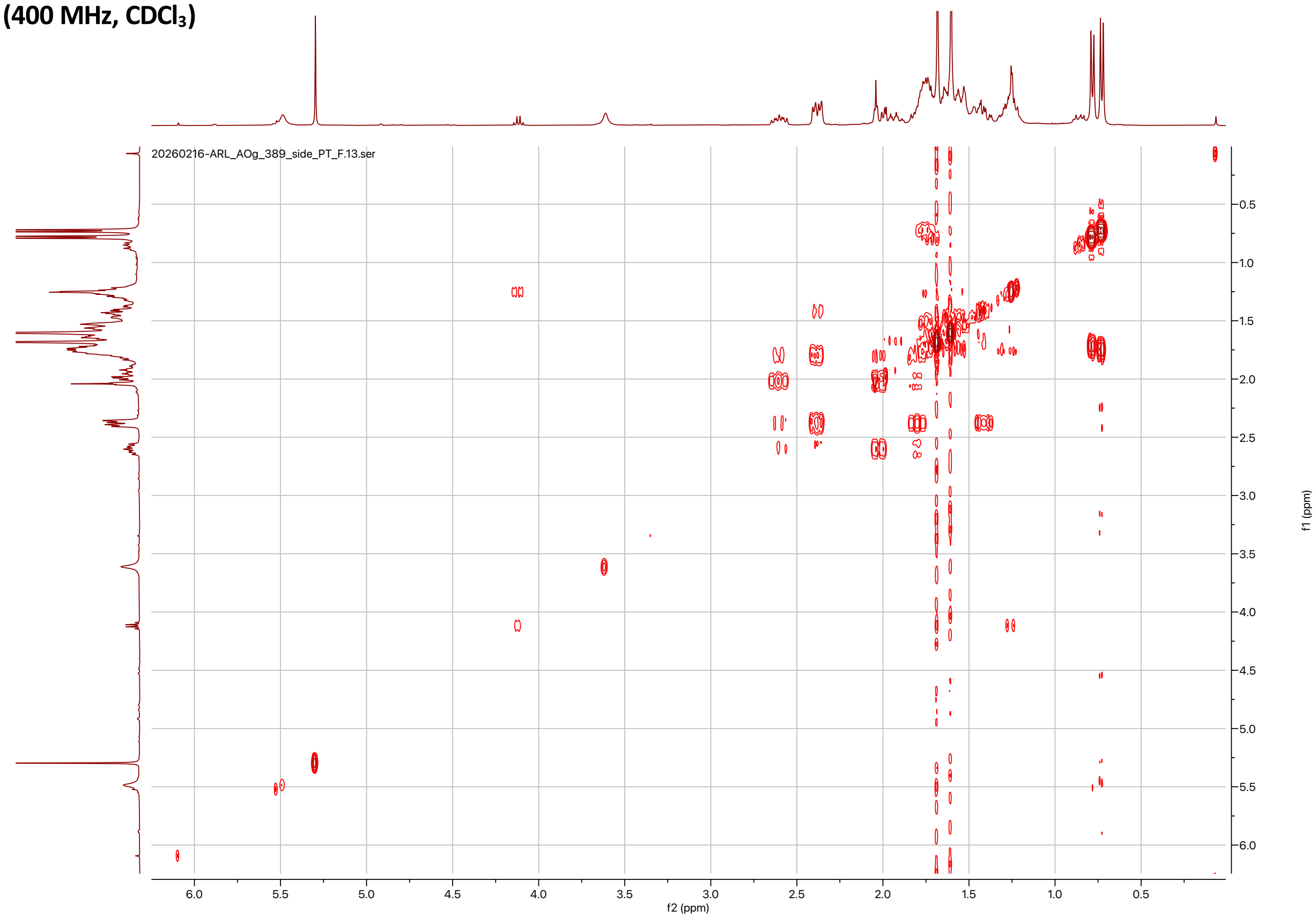

HMBC, ^{1}H NMR (400 MHz, $CDCl_3$), ^{13}C NMR (101 MHz, $CDCl_3$)

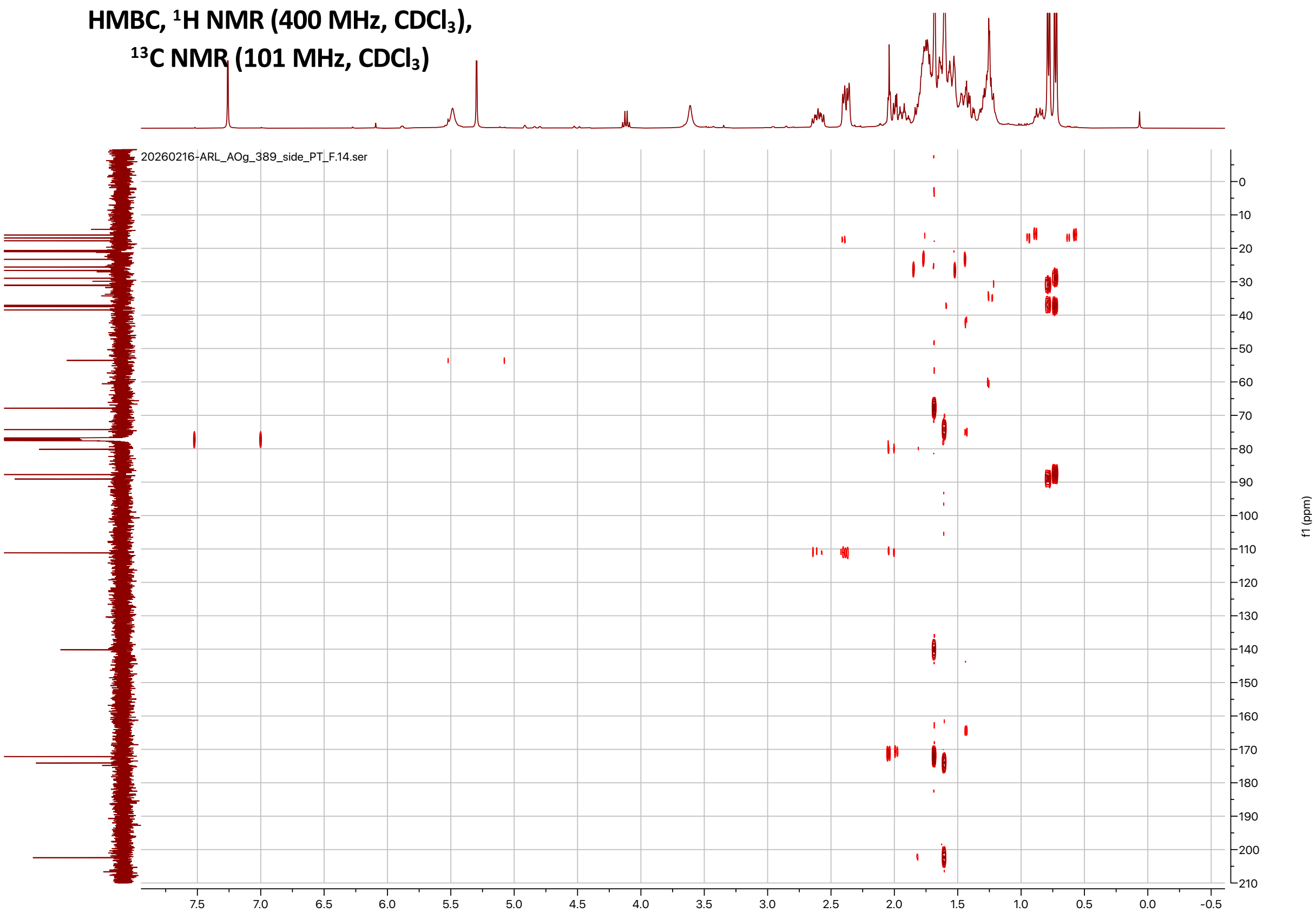

HSQC, ^{1}H NMR (400 MHz, $CDCl_3$), ^{13}C NMR (101 MHz, $CDCl_3$)

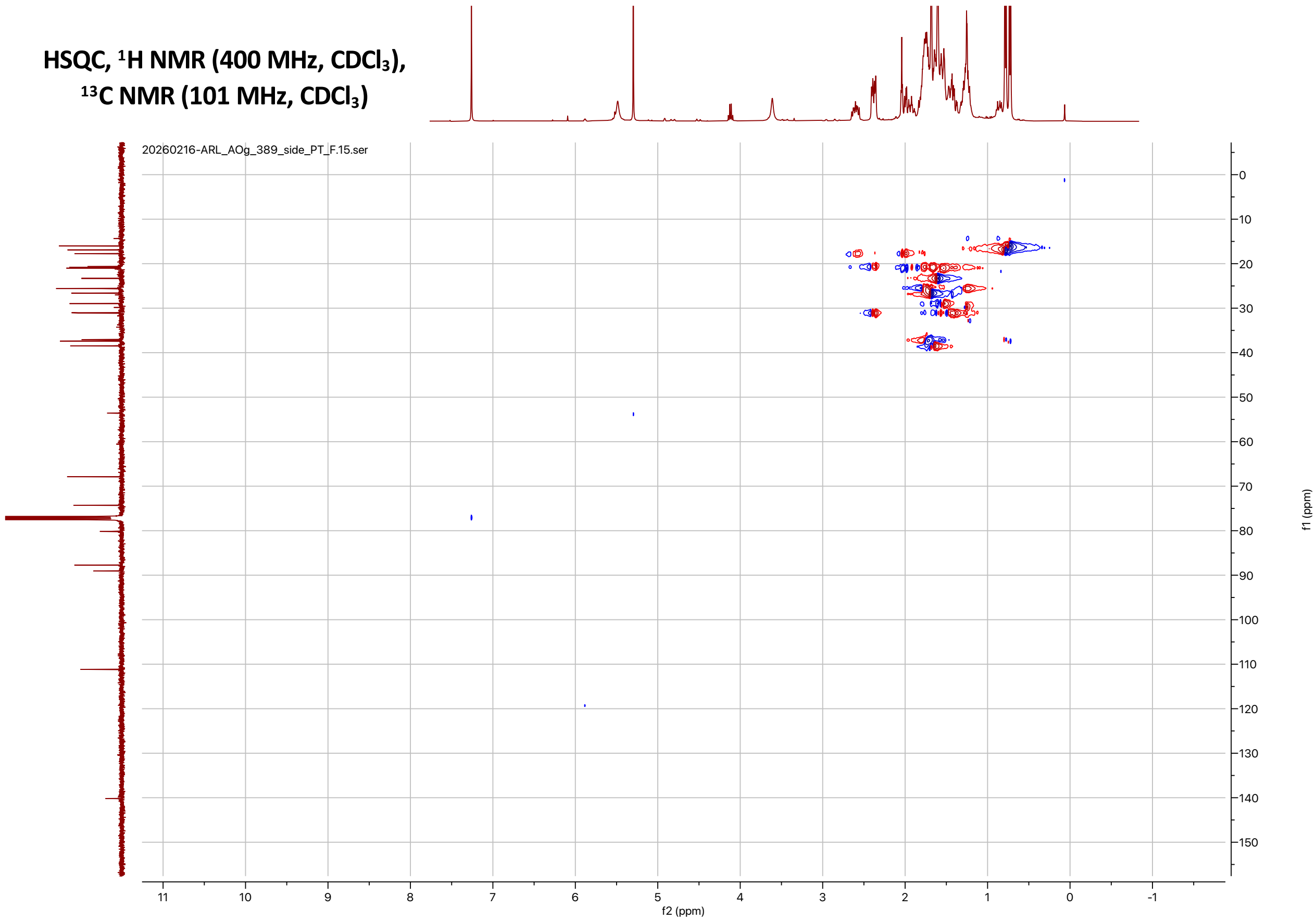

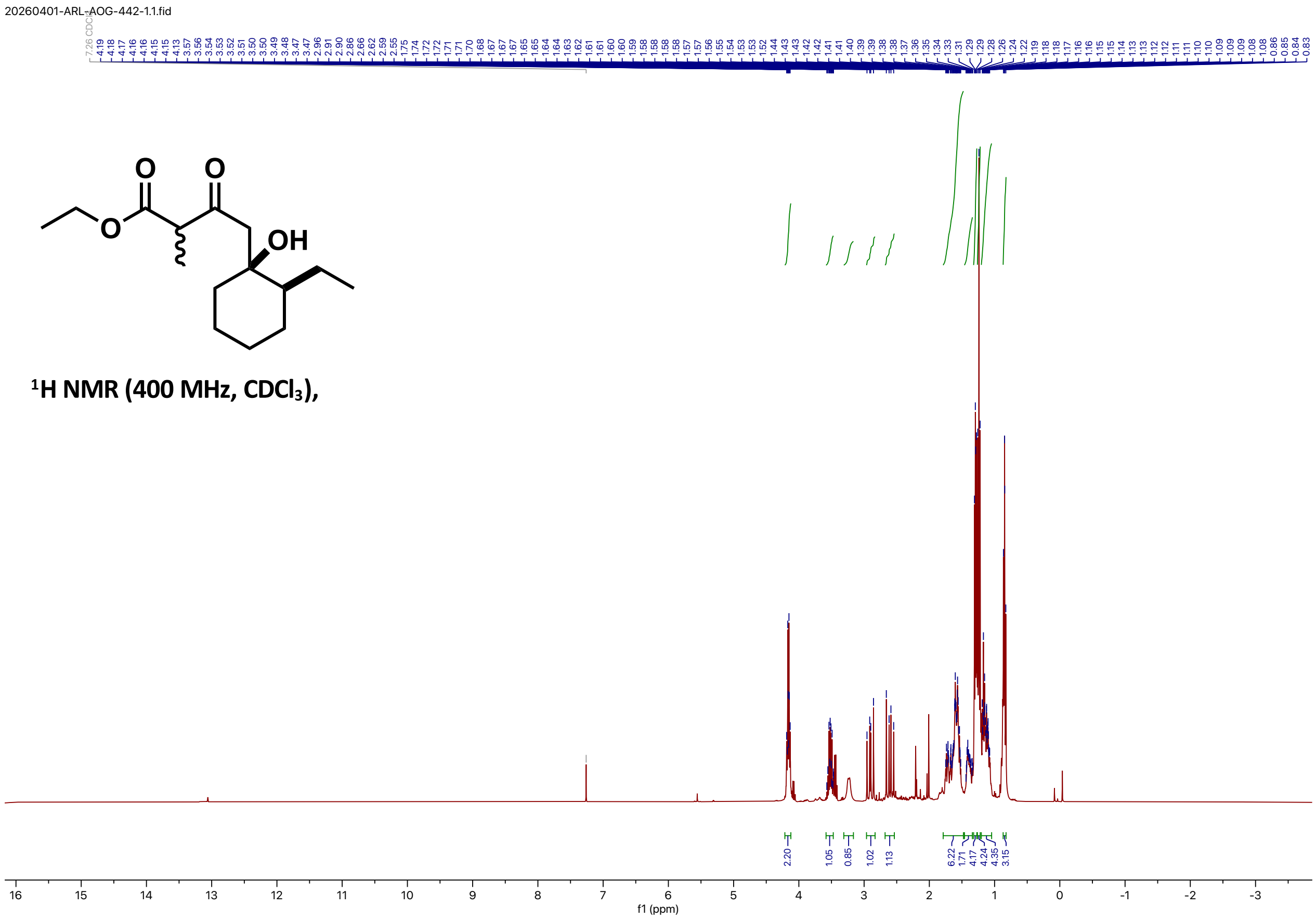
20260401-ARL-AOG-442-1.1.fid
7.26 CDCl3
4.19 4.18 4.17 4.16 4.16 4.15 4.15 4.13 3.57 3.56 3.54 3.53 3.52 3.51 3.50 3.50 3.49 3.48 3.47 3.47 2.96 2.91 2.90 2.86 2.66 2.62 2.59 2.55 1.75 1.74 1.72 1.72 1.71 1.70 1.68 1.67 1.67 1.65 1.65 1.64 1.64 1.63 1.62 1.61 1.61 1.60 1.60 1.59 1.58 1.58 1.58 1.58 1.57 1.57 1.56 1.55 1.54 1.53 1.53 1.52 1.44 1.43 1.43 1.42 1.42 1.41 1.41 1.40 1.39 1.39 1.38 1.38 1.37 1.36 1.35 1.34 1.33 1.31 1.29 1.29 1.28 1.26 1.24 1.22 1.19 1.18 1.18 1.18 1.17 1.16 1.16 1.15 1.15 1.14 1.13 1.13 1.13 1.12 1.12 1.11 1.11 1.10 1.10 1.09 1.09 1.09 1.08 1.08 0.86 0.85 0.84 0.83
O
O
O
OH
¹H NMR (400 MHz, CDCl₃),
2.20 1.05 0.85 1.02 1.13 6.22 1.71 4.17 4.24 4.35 3.15
16 15 14 13 12 11 10 9 8 7 6 5 4 3 2 1 0 -1 -2 -3
f1 (ppm)

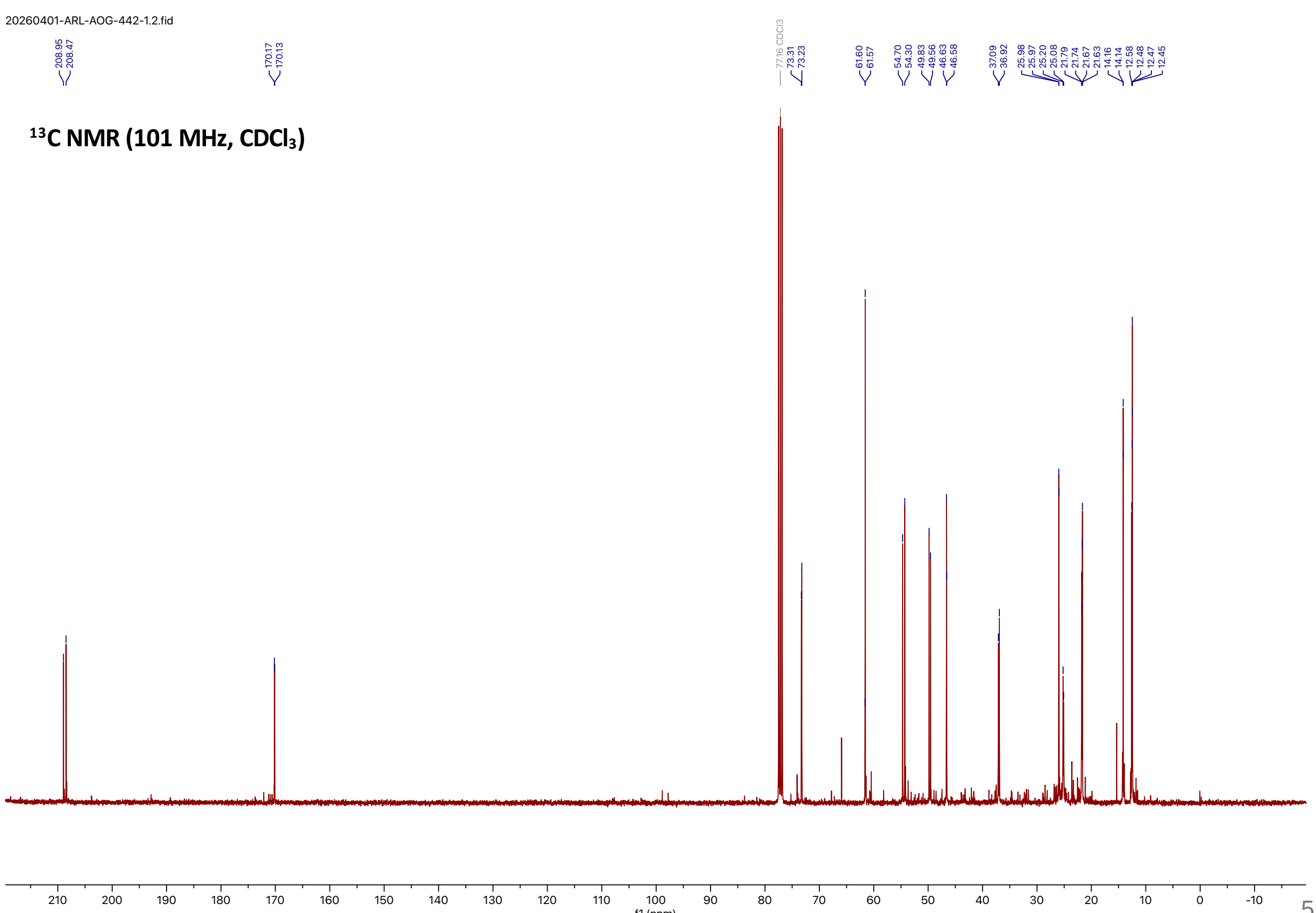
20260401-ARL-AOG-442-1.2.fid
208.95 208.47
170.17 170.13
77.16 CDCl3
73.31 73.23
61.60 61.57
54.70 54.30 49.83 49.56 46.63 46.58
37.09 36.92 25.98 25.97 25.20 25.08 21.79 21.74 21.67 21.63 14.16 14.14 12.58 12.48 12.47 12.45
¹³C NMR (101 MHz, CDCl₃)
210 200 190 180 170 160 150 140 130 120 110 100 90 80 70 60 50 40 30 20 10 0 -10
f1 (ppm)

20260407-ARL_AOG_443_1.1.fid

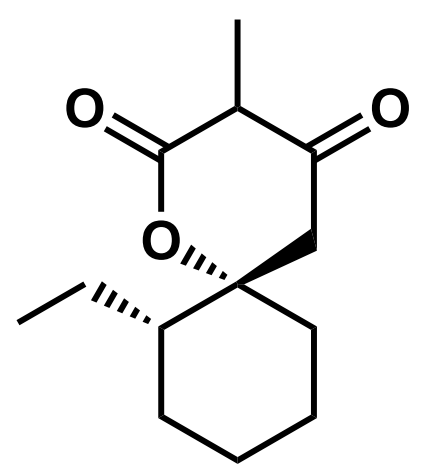

¹H NMR (400 MHz, DMSO)

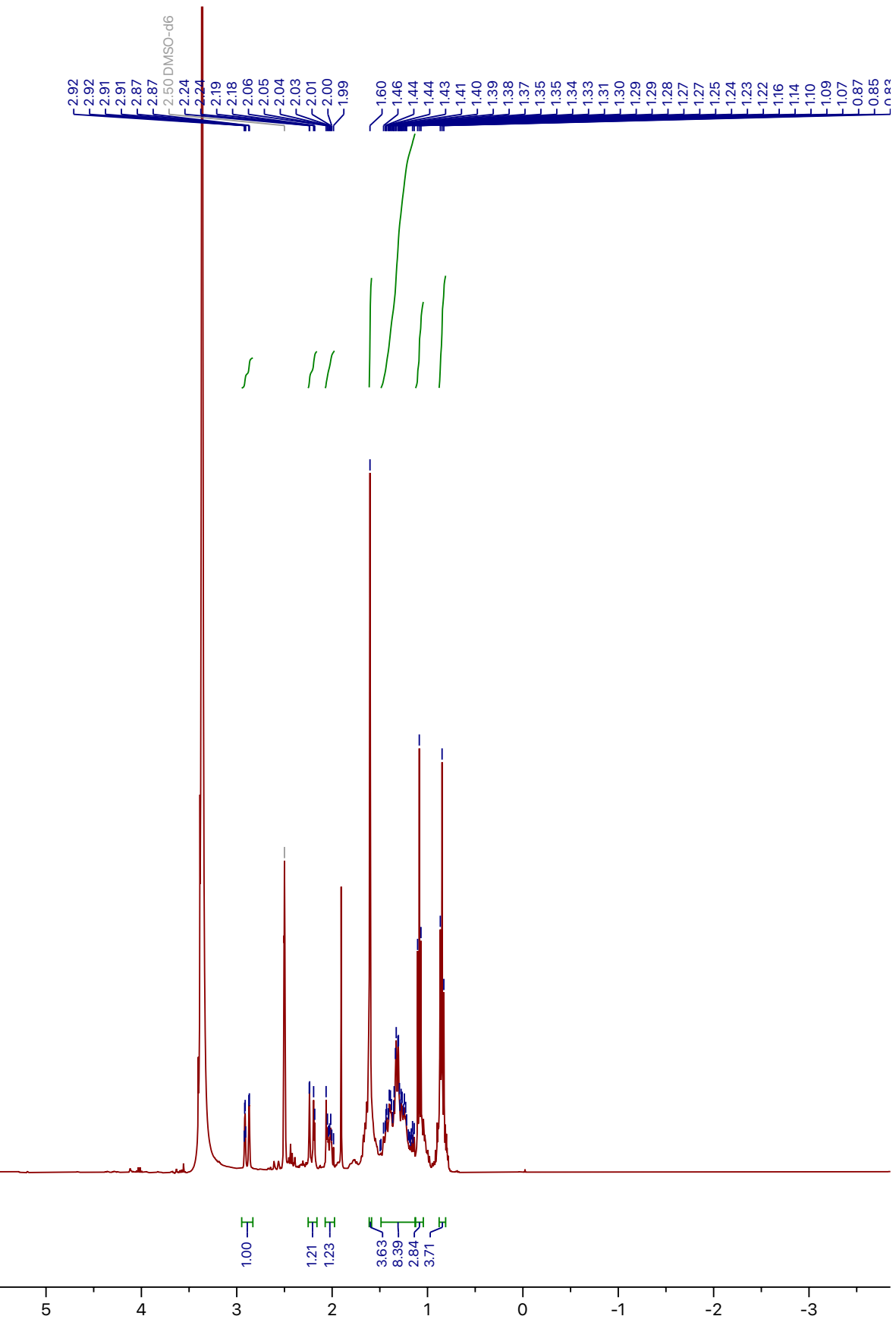

20260407-ARL_AOG_443_1.2.fid

¹³C NMR (101 MHz, DMSO)

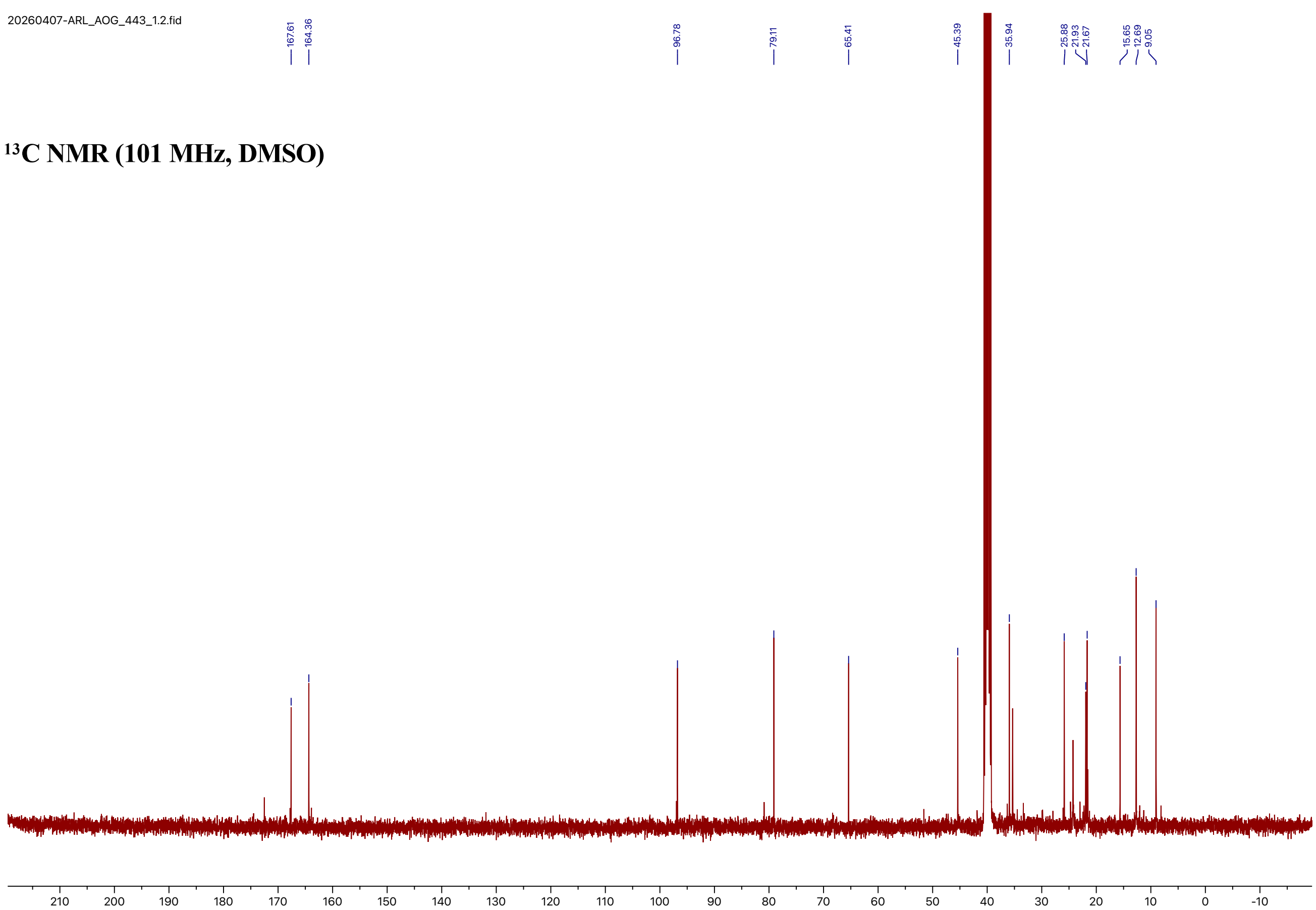

20251113-ARL_AOG_338_OH.10.fid

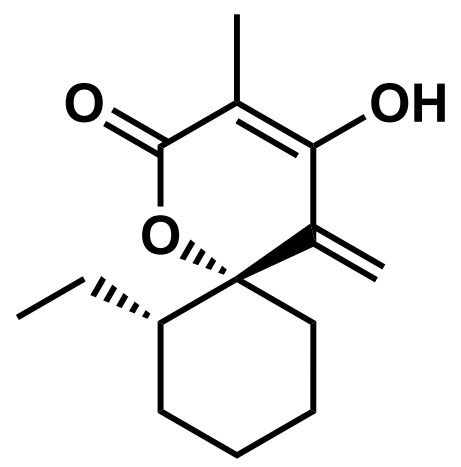

^{1}H NMR (400 MHz, $CDCl_3$),

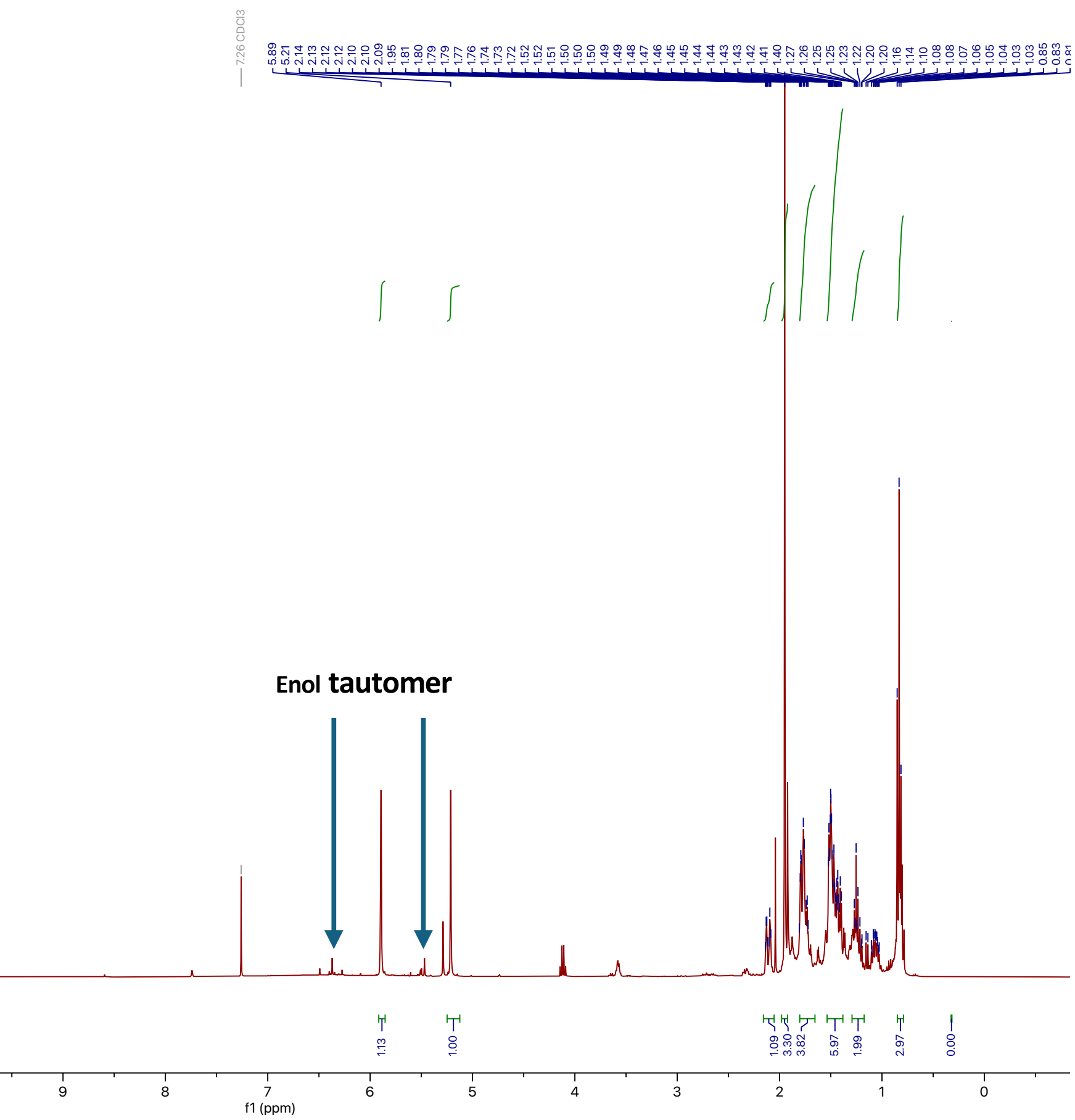

20251113-ARL_AOG_338_OH.11.fid

^{13}C NMR (101 MHz, $CDCl_3$)

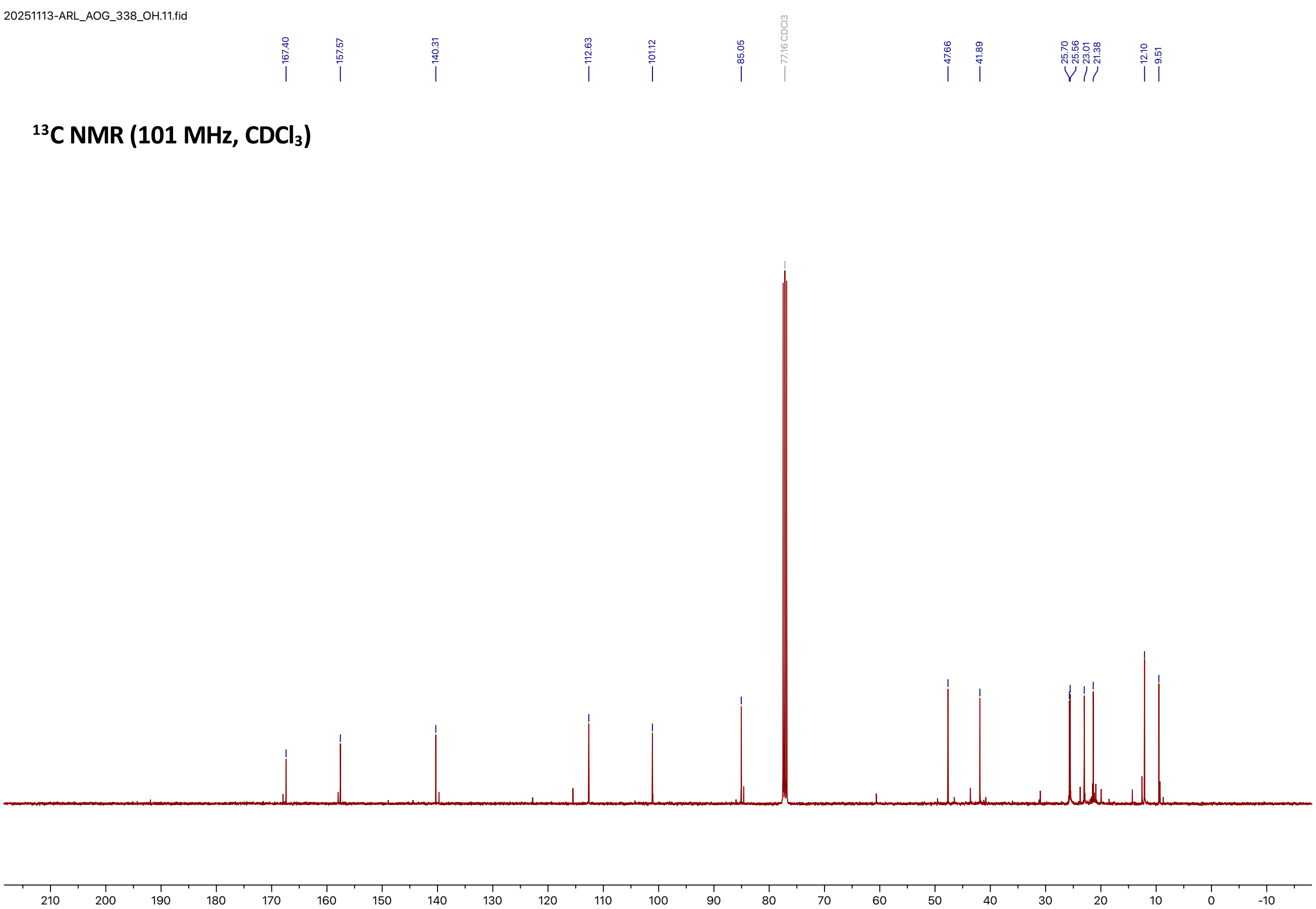

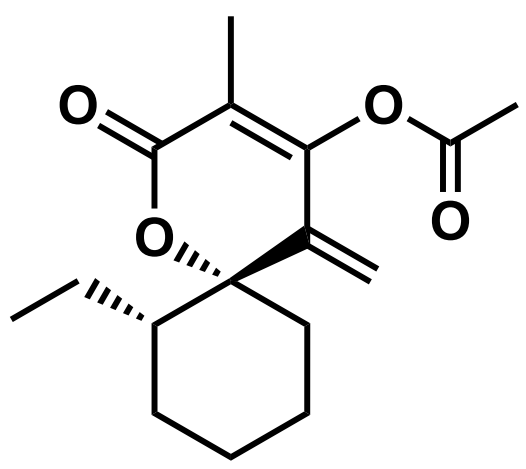

¹H NMR (400 MHz, CDCl$_3$),

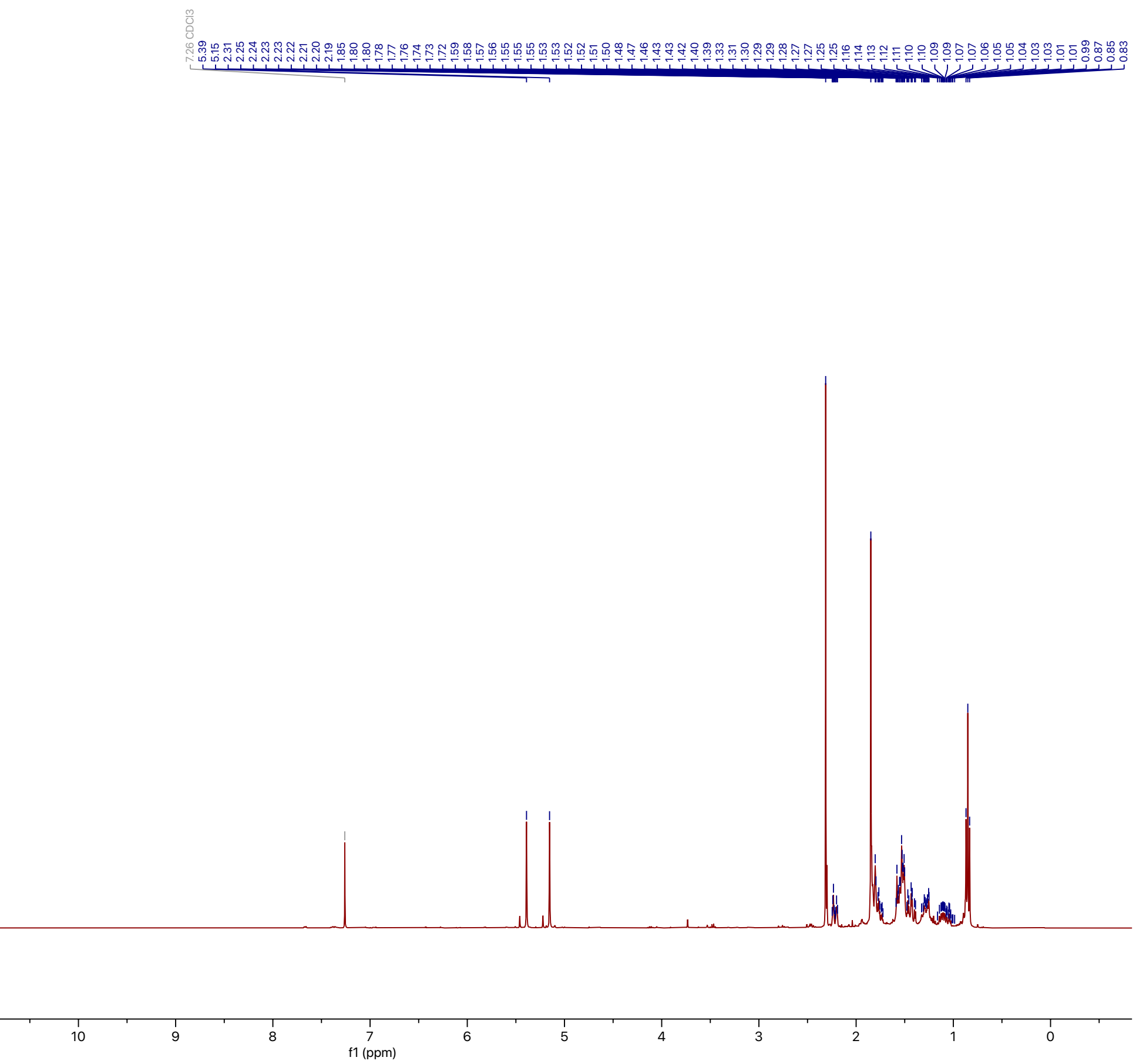

¹³C NMR (101 MHz, CDCl$_3$)

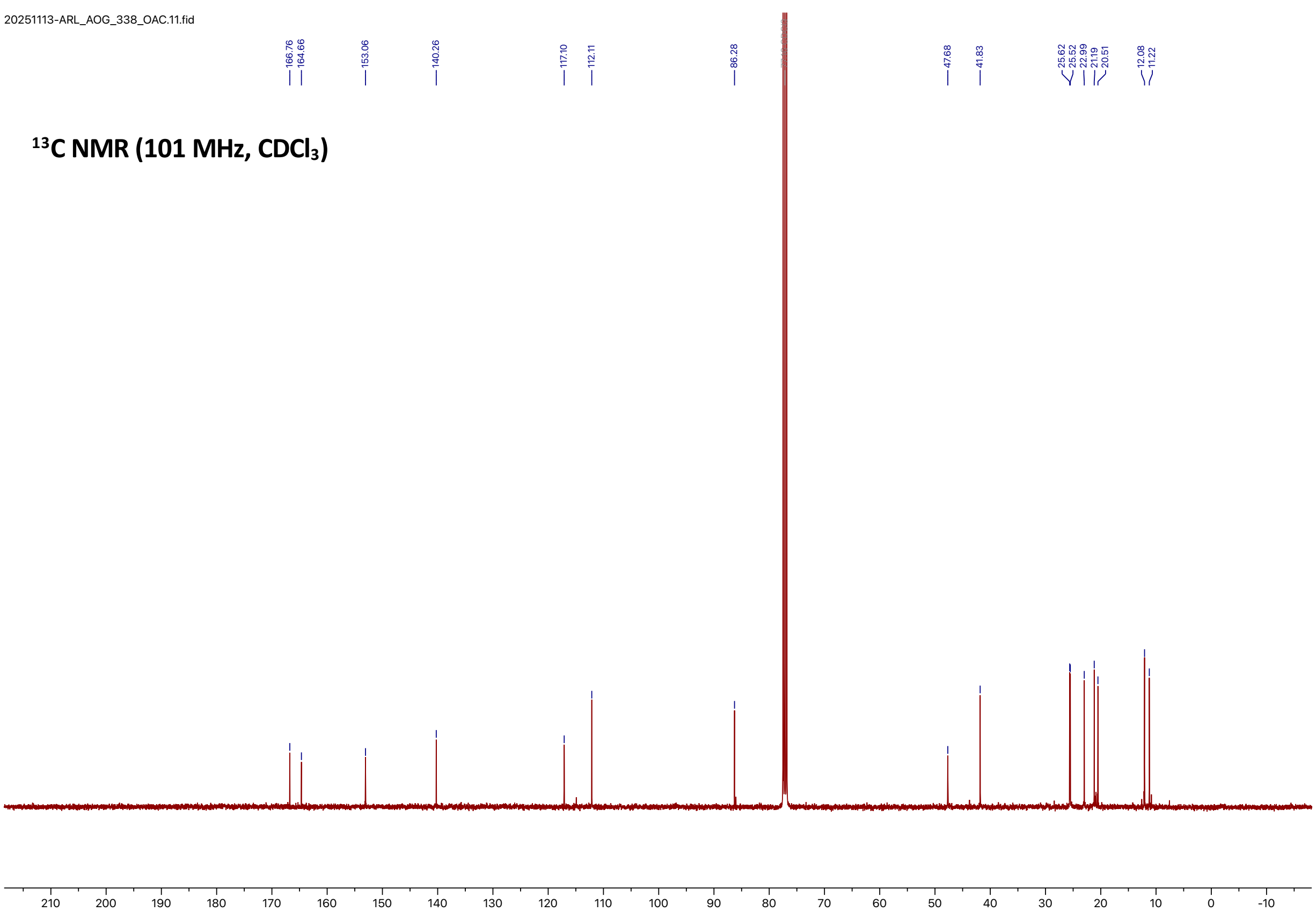

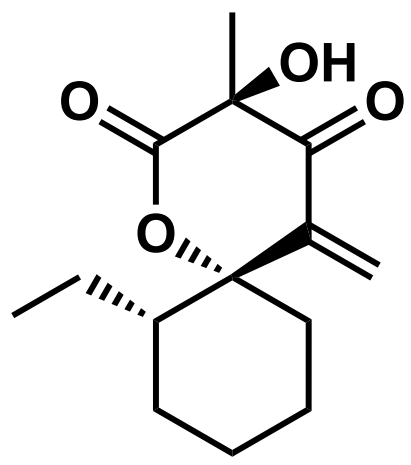

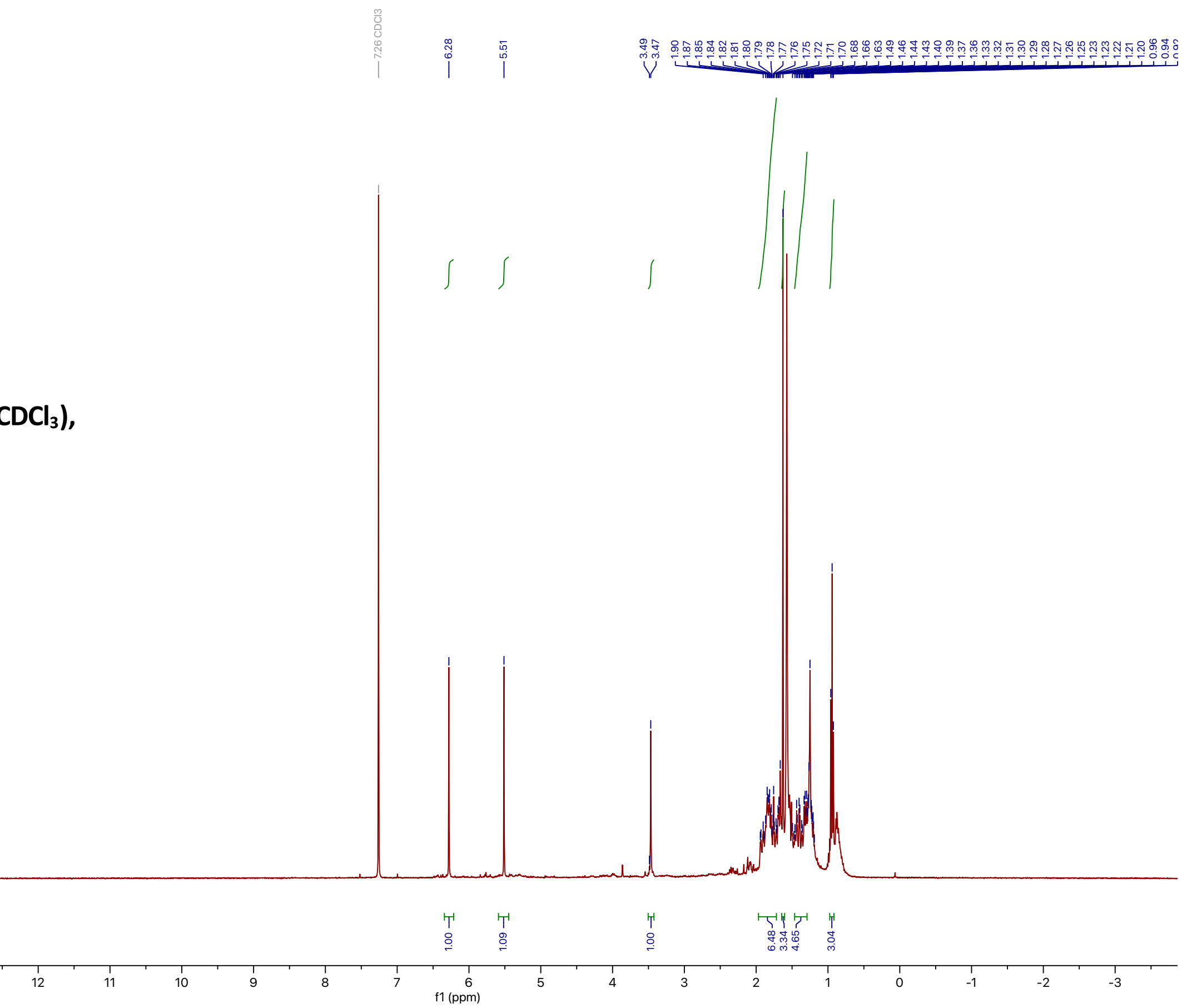

^{1}H NMR (400 MHz, $CDCl_3$),

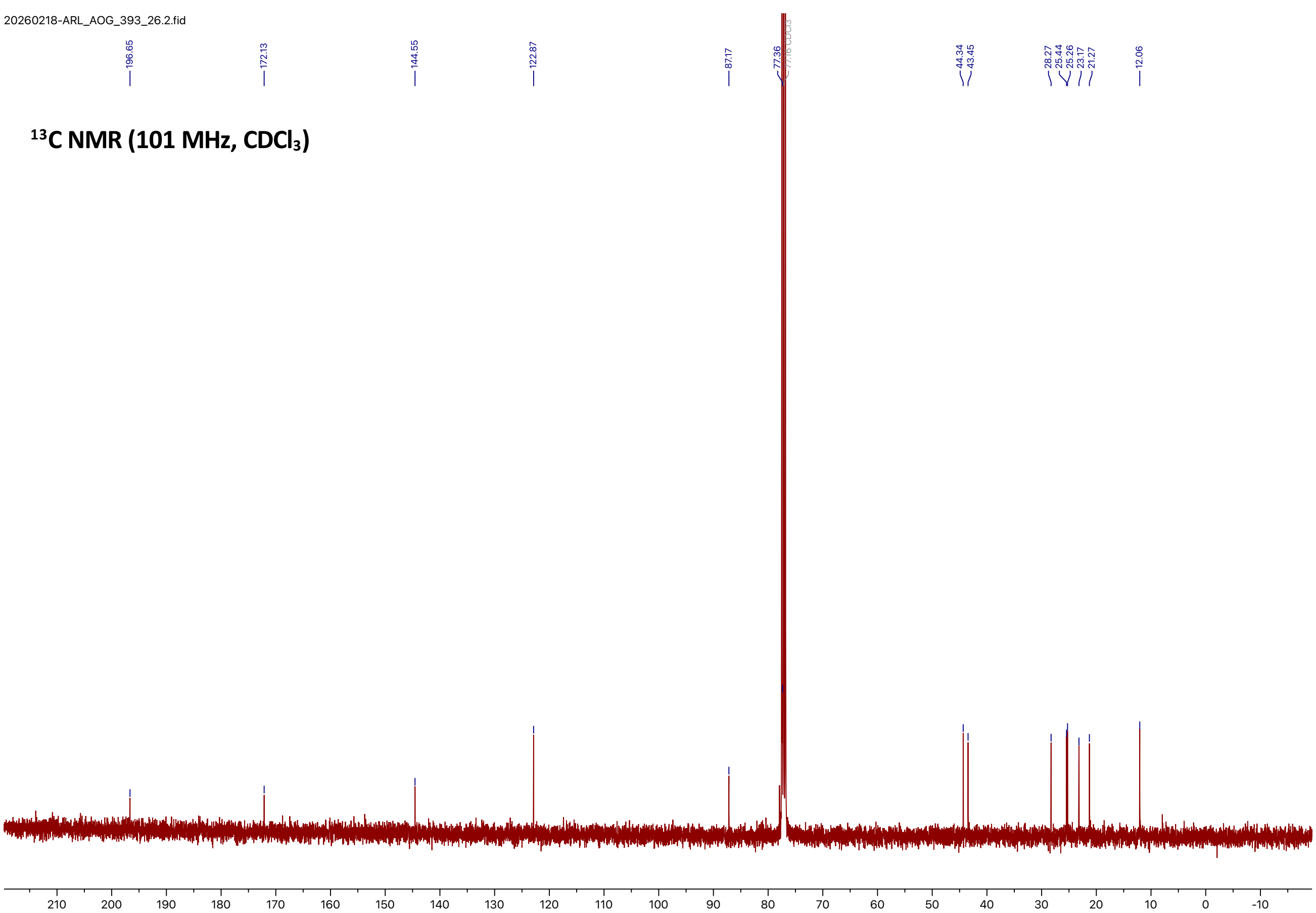

^{13}C NMR (101 MHz, $CDCl_3$)

20260218-ARL_AOG_393_36.1.fid

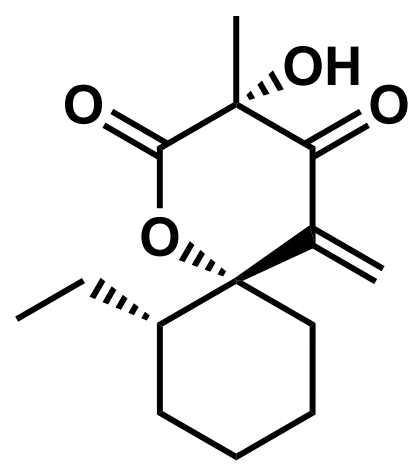

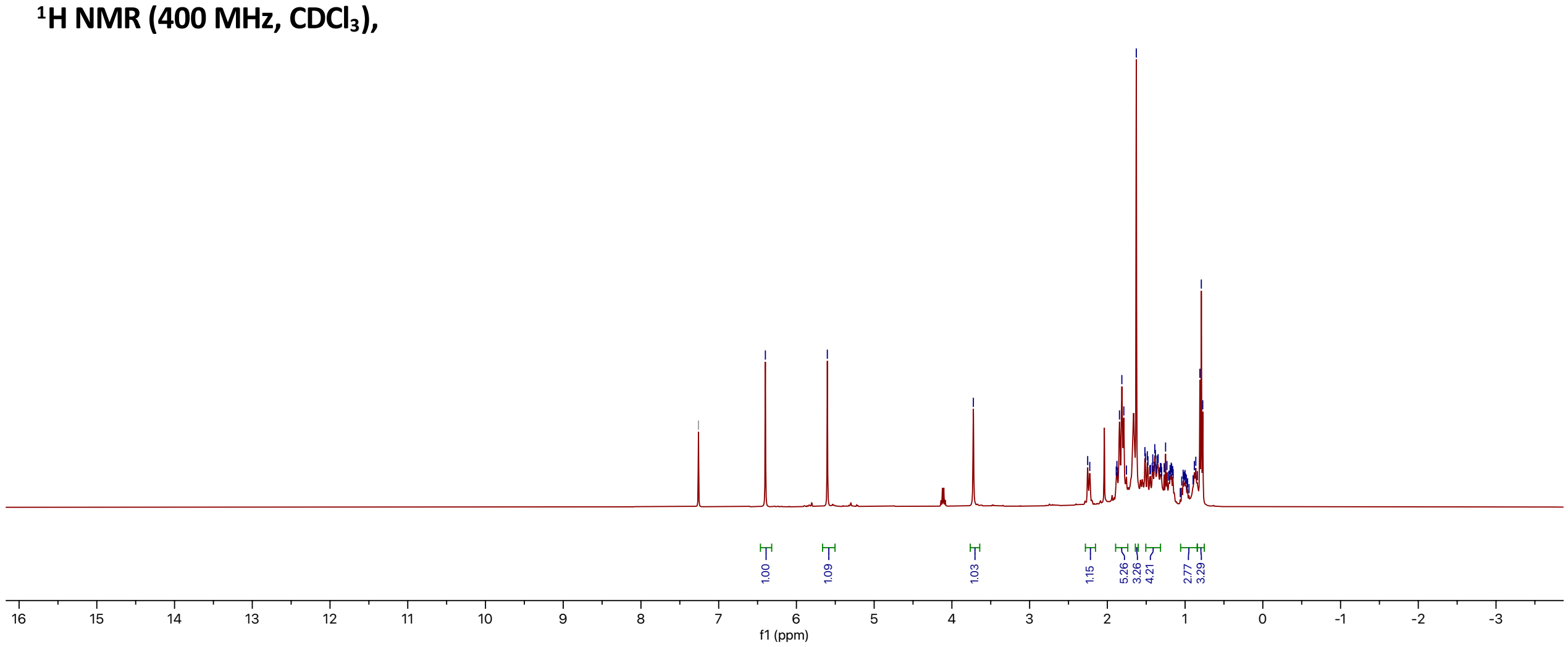

^{1}H NMR (400 MHz, $CDCl_3$),

20260218-ARL_AOG_393_36.2.fid

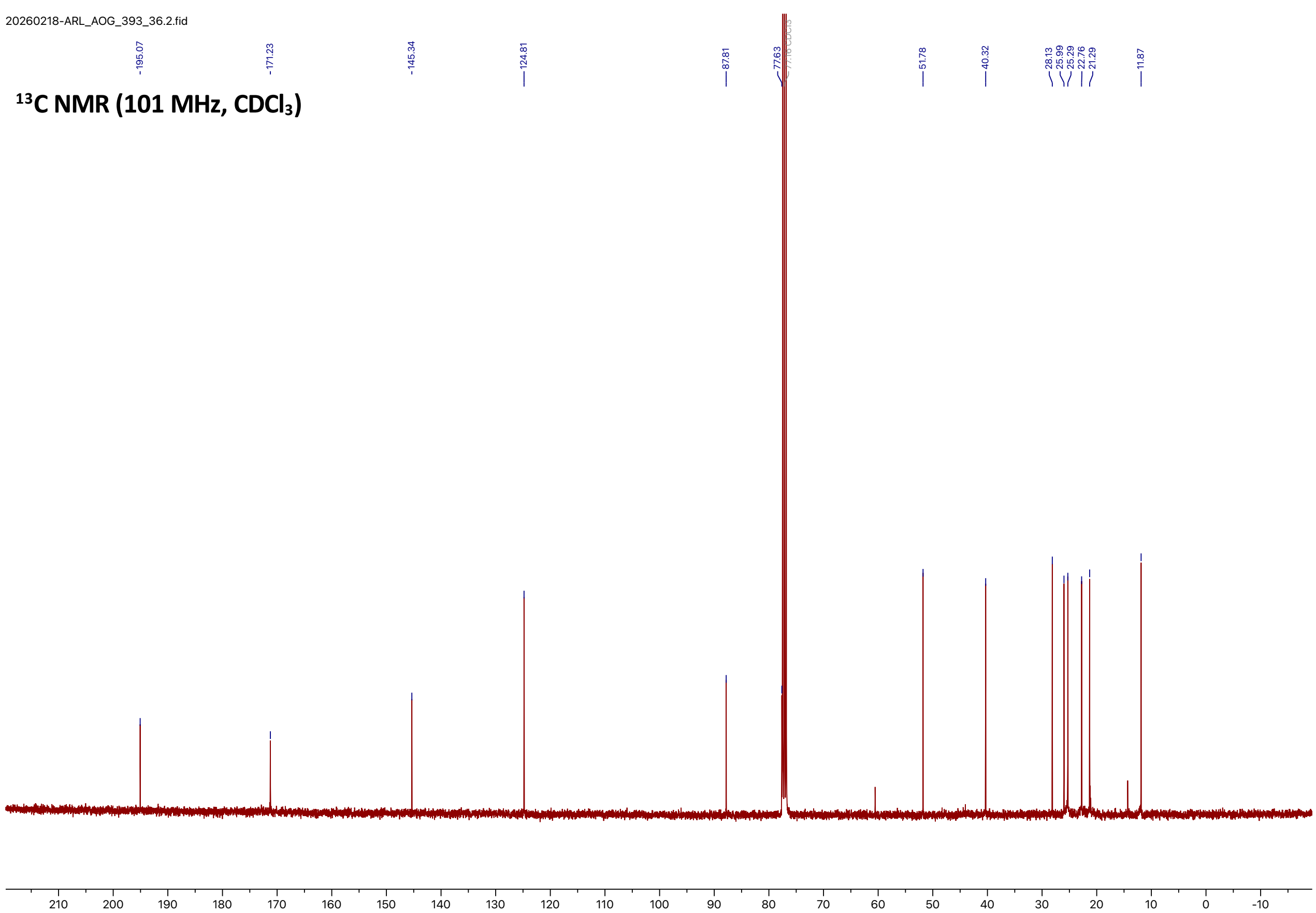

^{13}C NMR (101 MHz, $CDCl_3$)

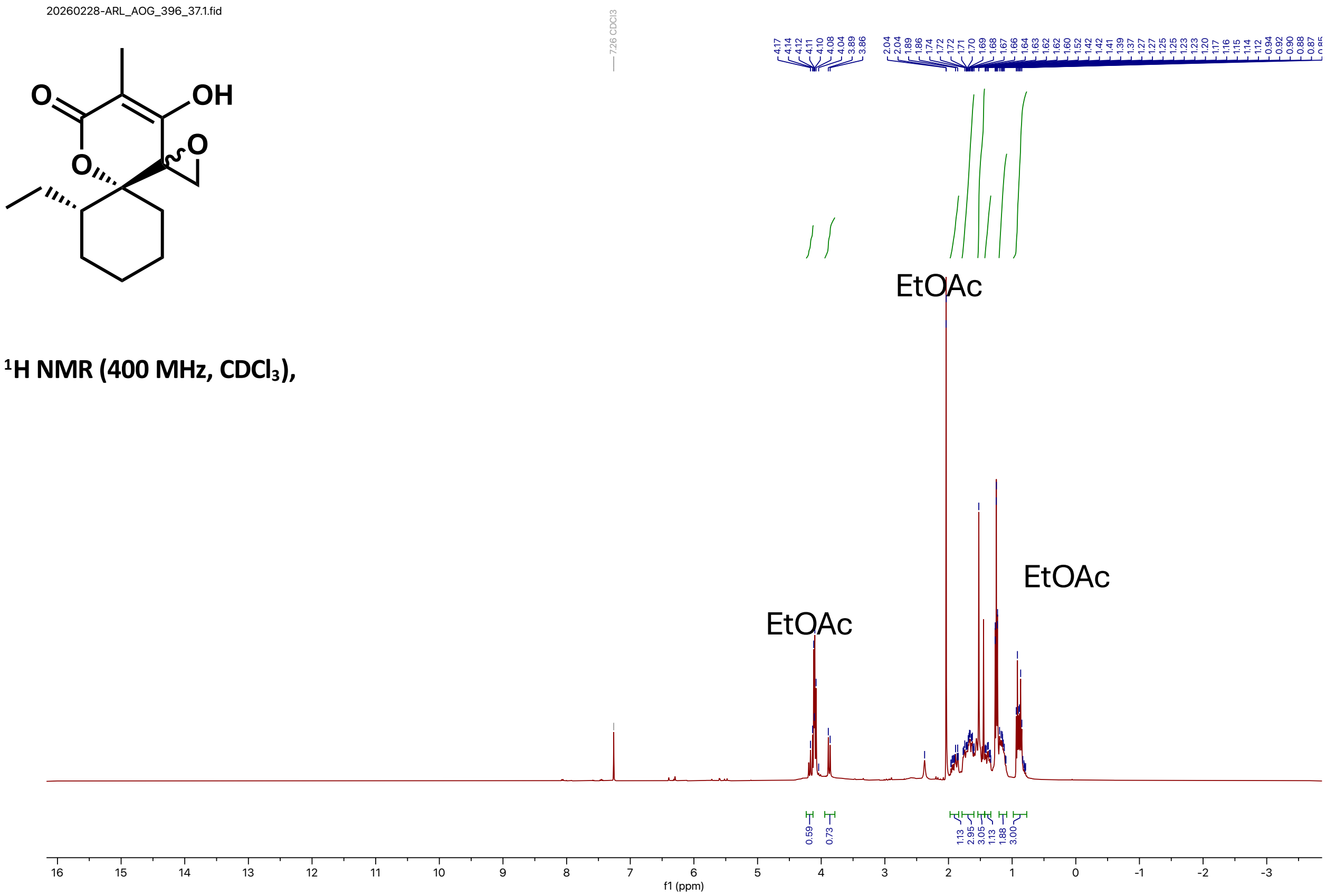

¹H NMR (400 MHz, $CDCl_3$),

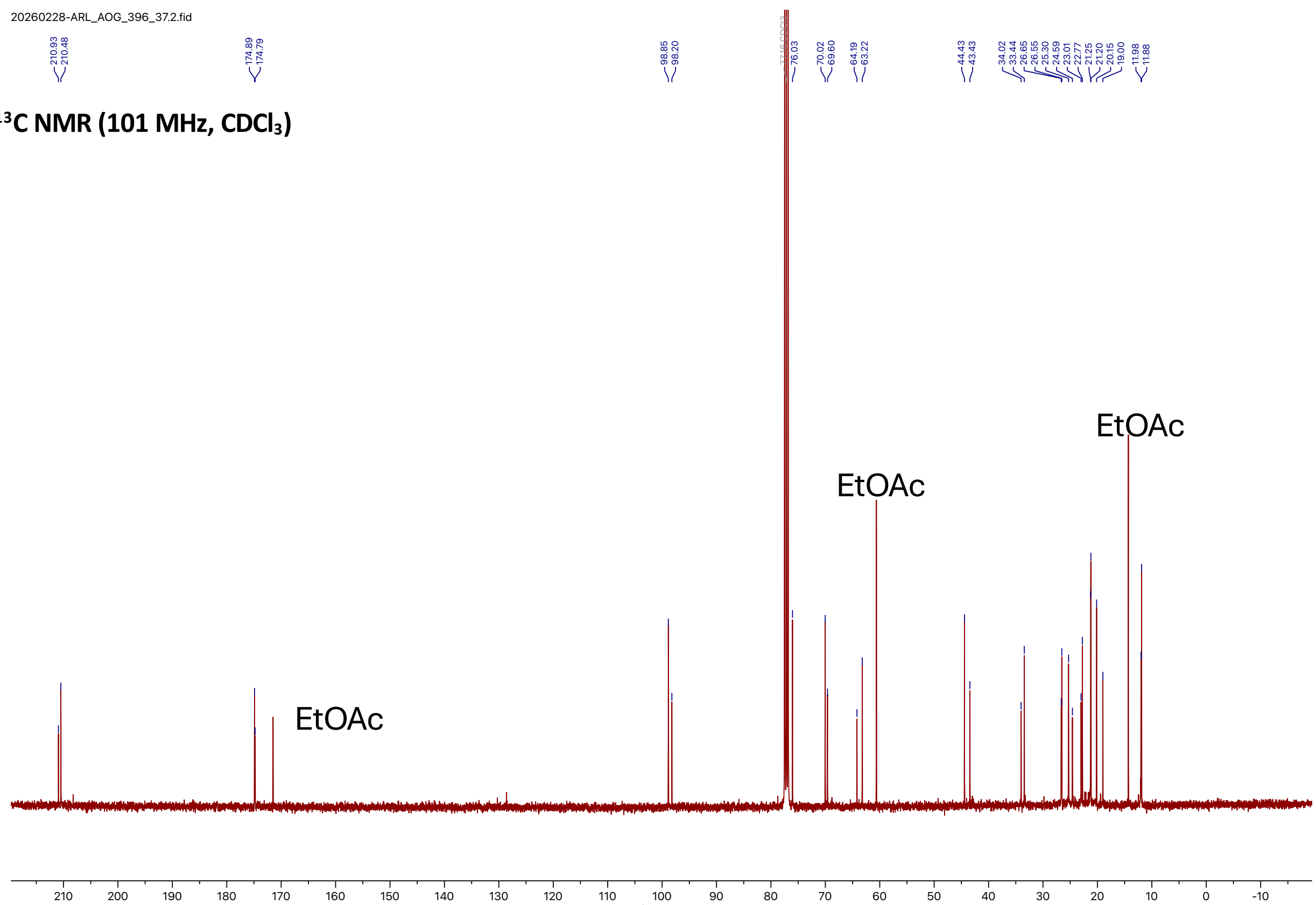

¹³C NMR (101 MHz, $CDCl_3$)

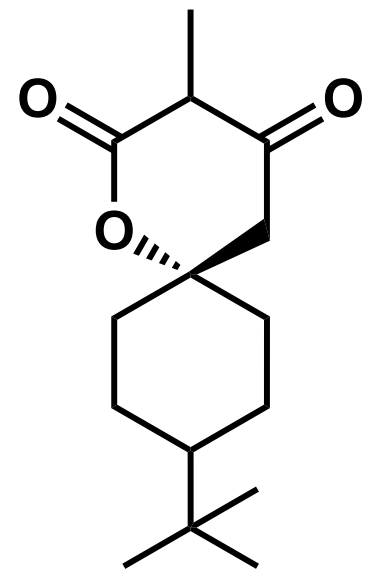

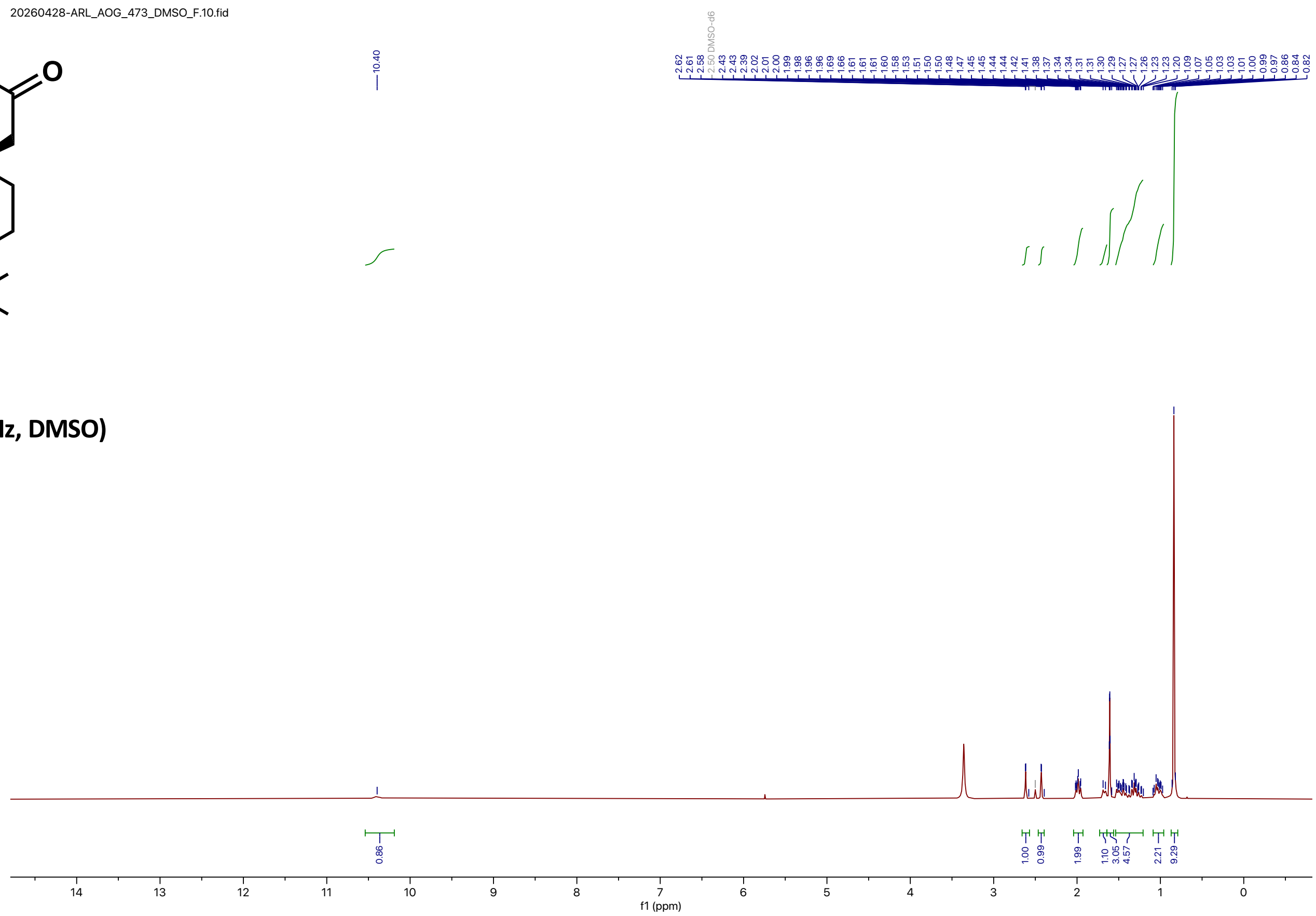

^{1}H NMR (400 MHz, DMSO)

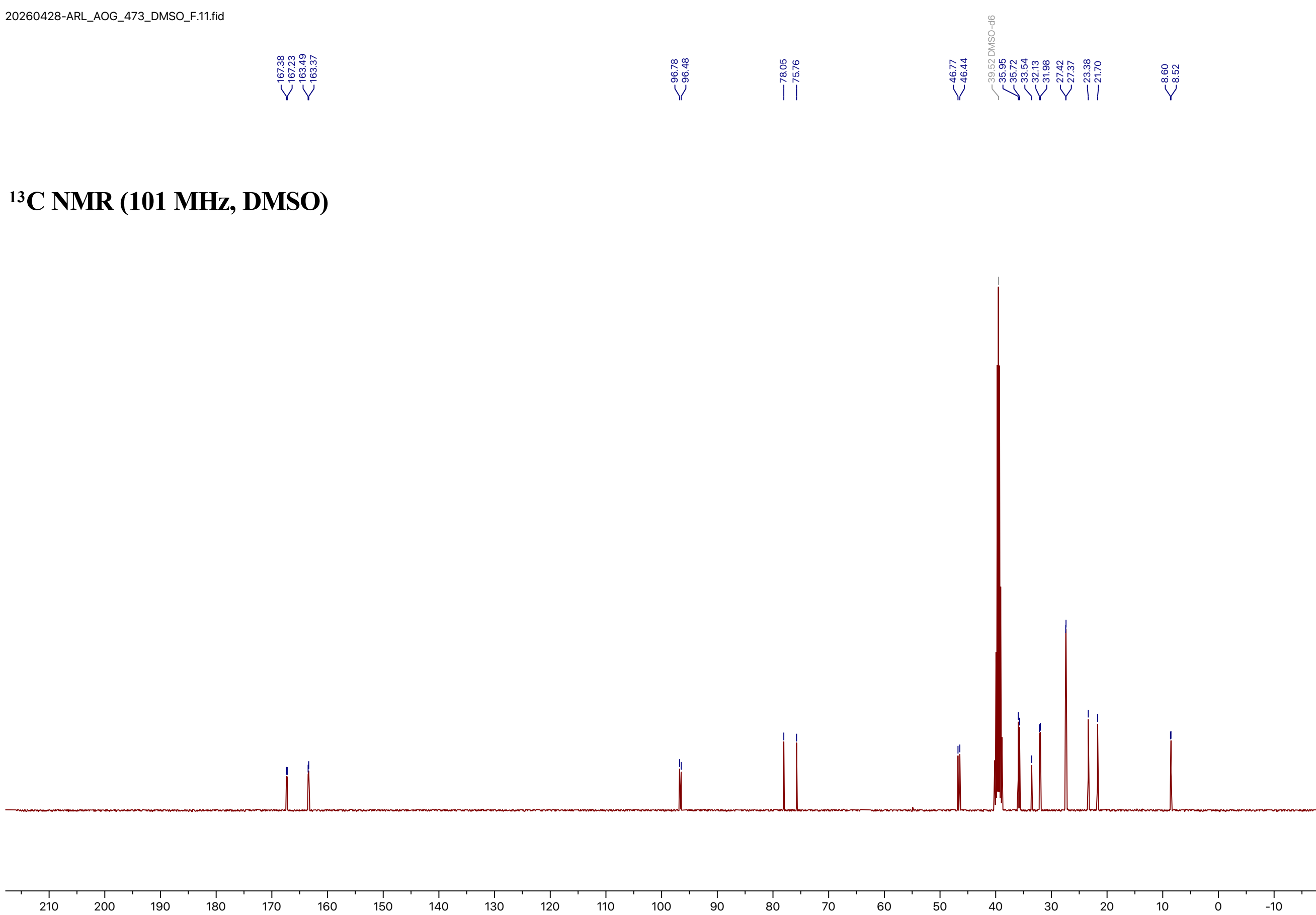

^{13}C NMR (101 MHz, DMSO)

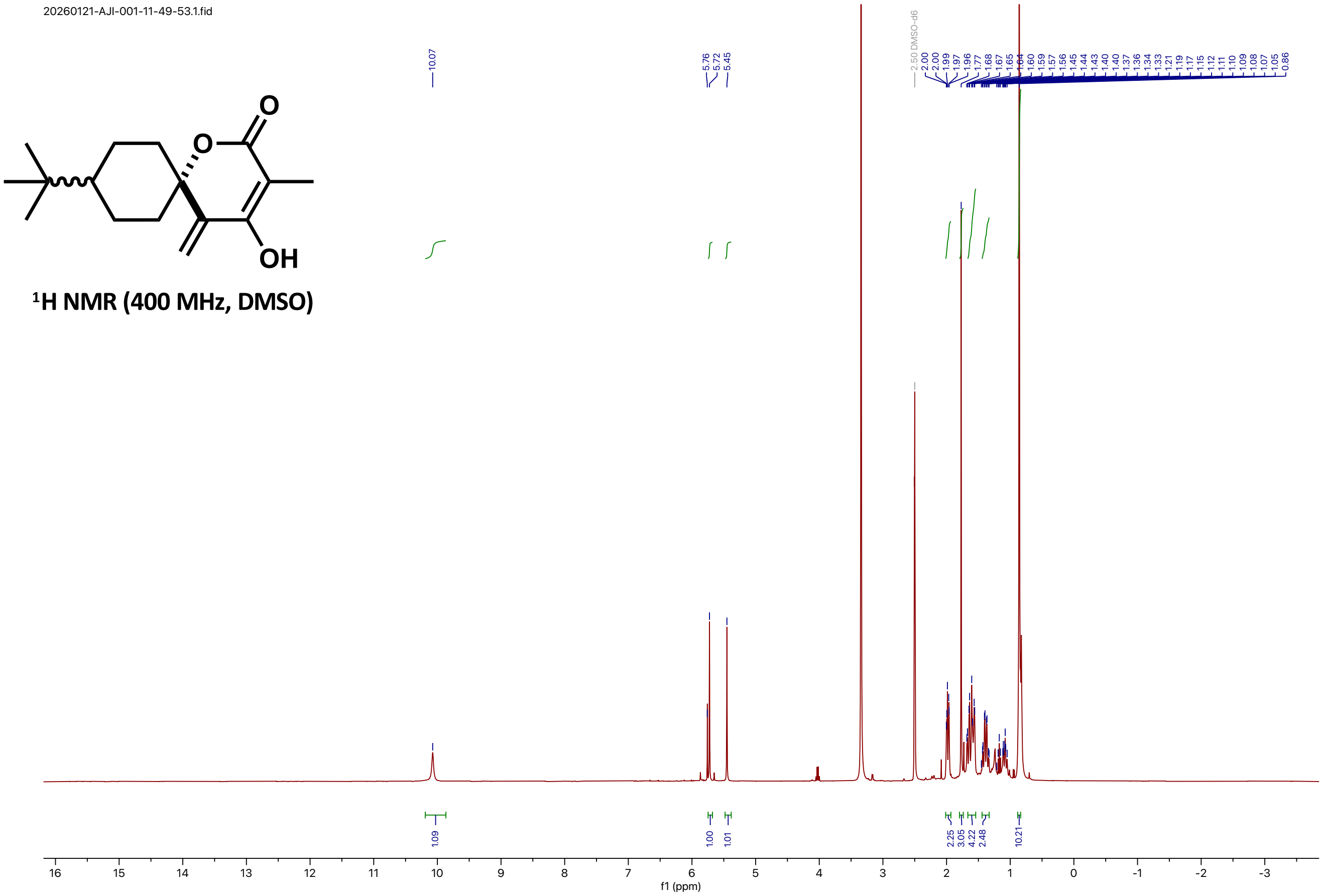

¹H NMR (400 MHz, DMSO)

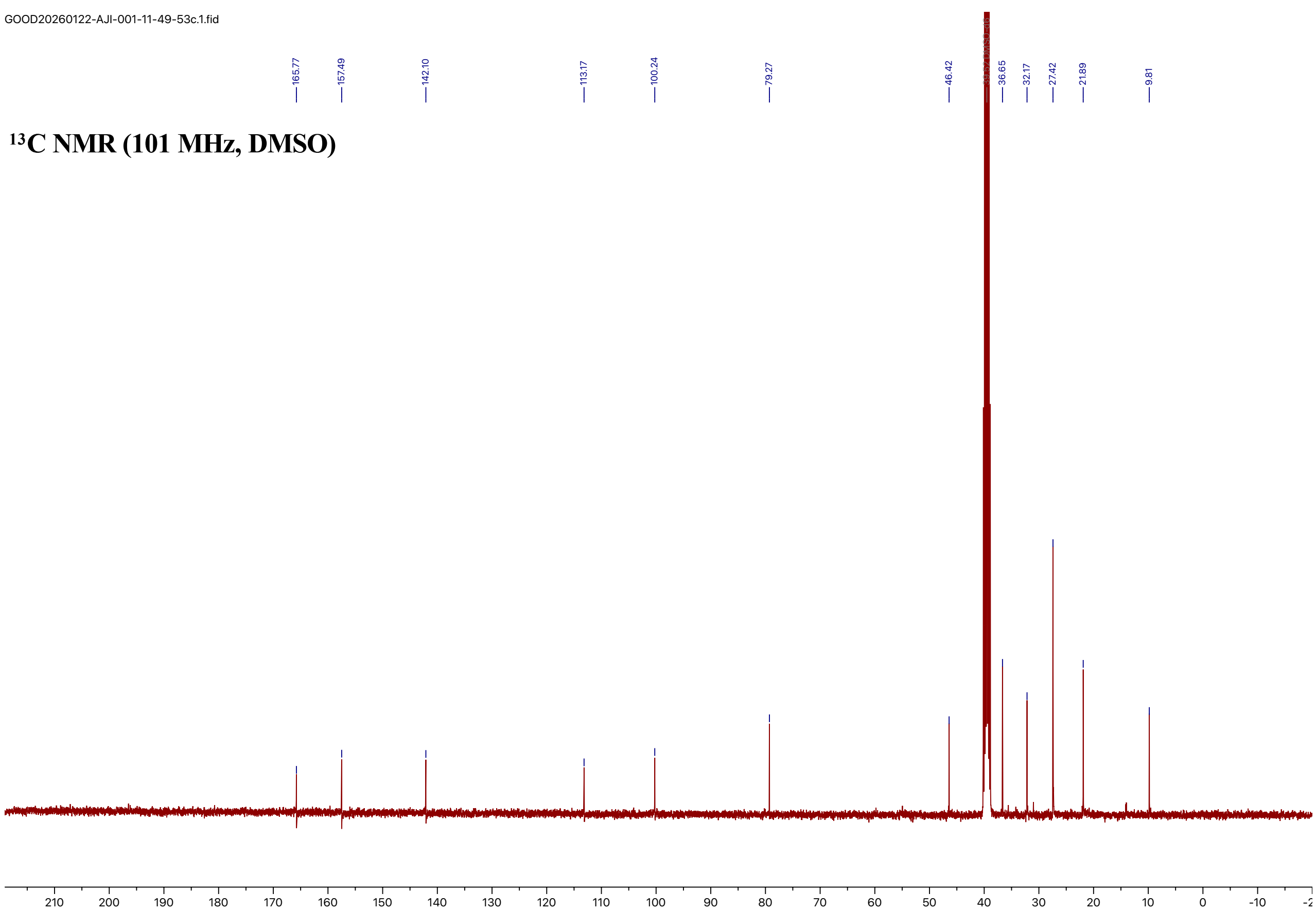

¹³C NMR (101 MHz, DMSO)

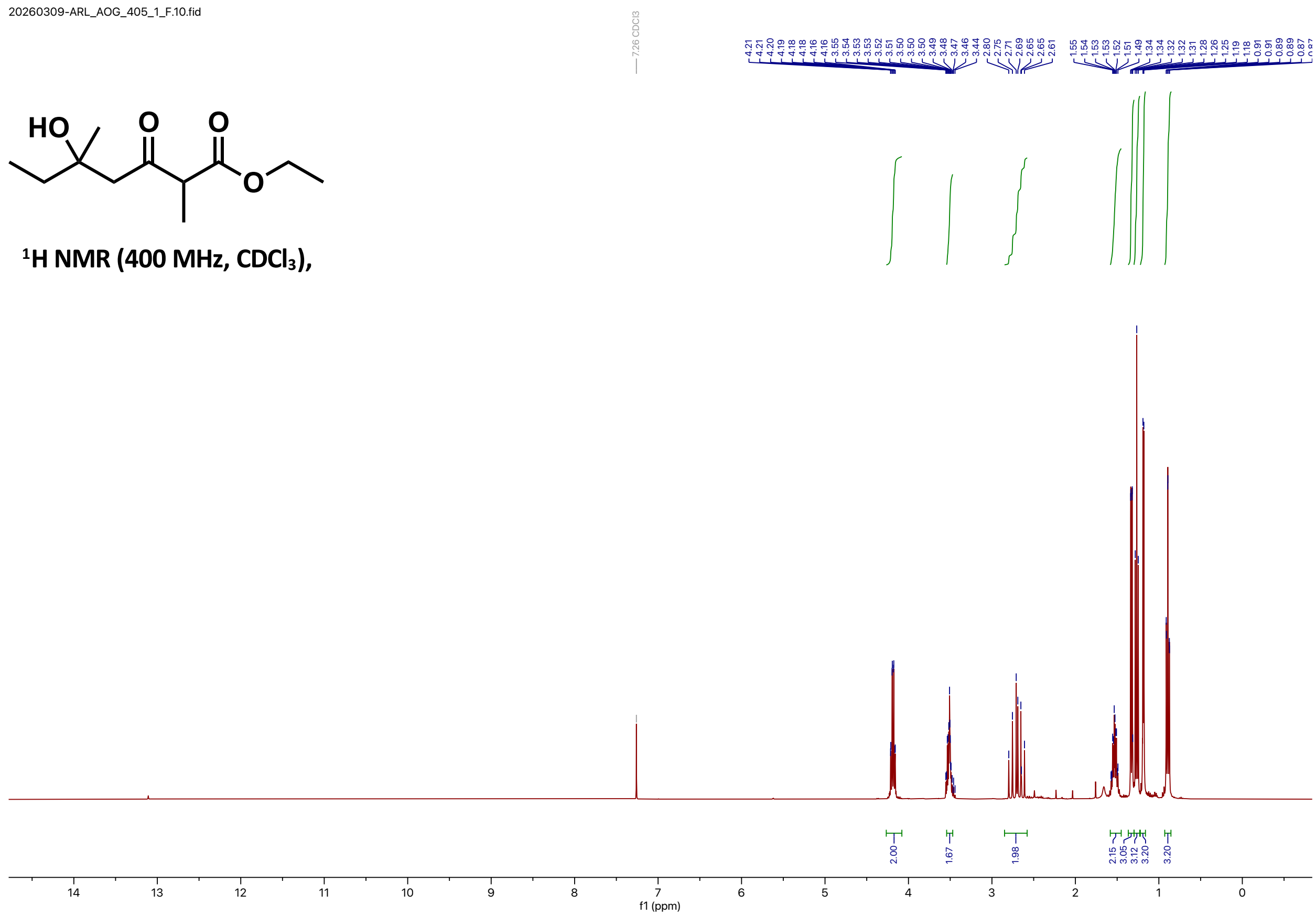

^{1}H NMR (400 MHz, $CDCl_3$),

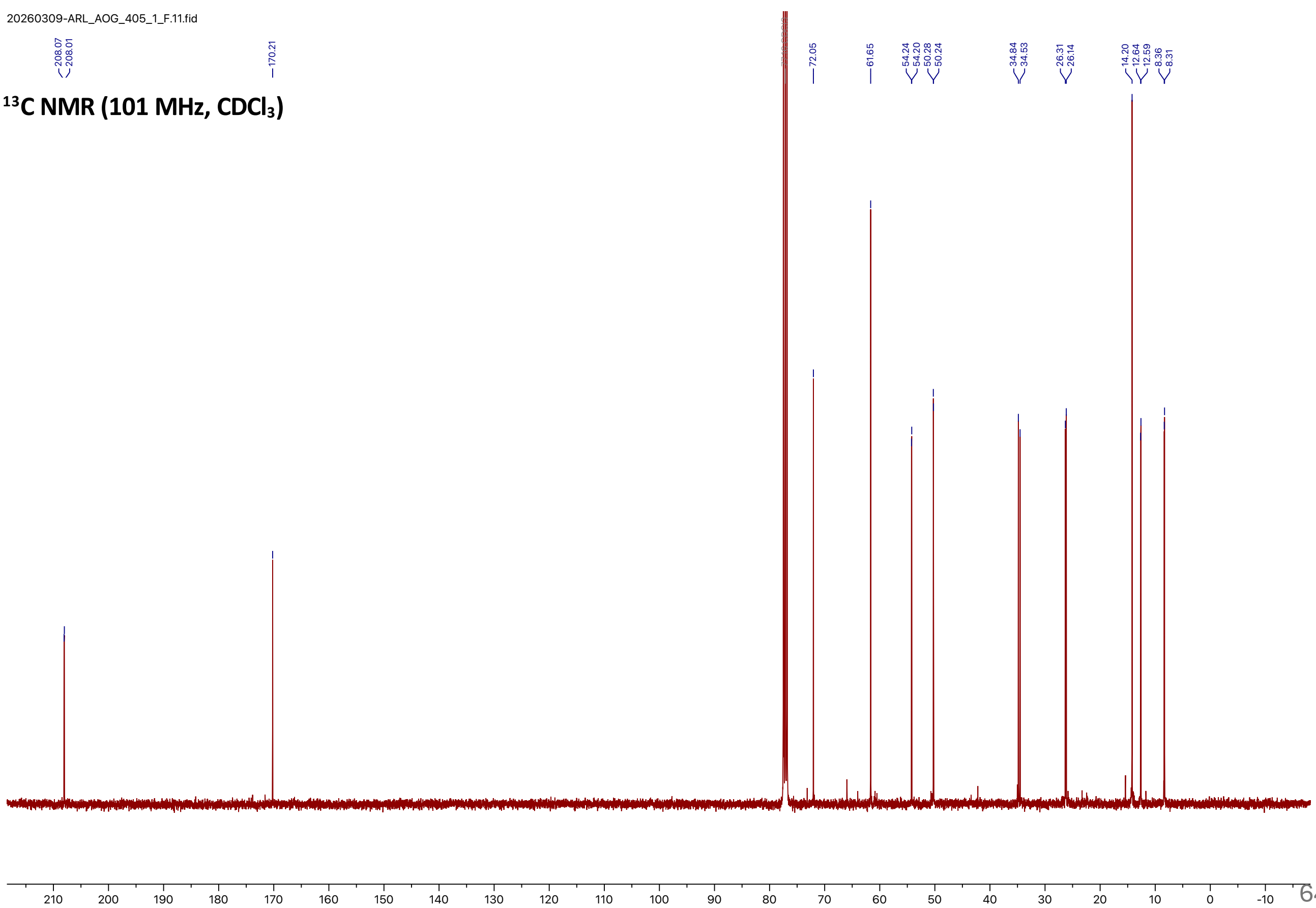

^{13}C NMR (101 MHz, $CDCl_3$)

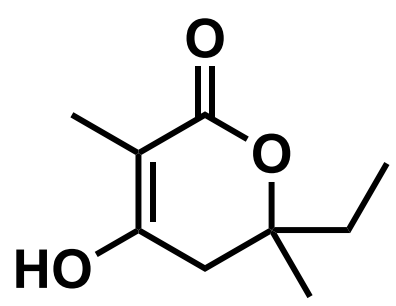

^{1}H NMR (400 MHz, DMSO)

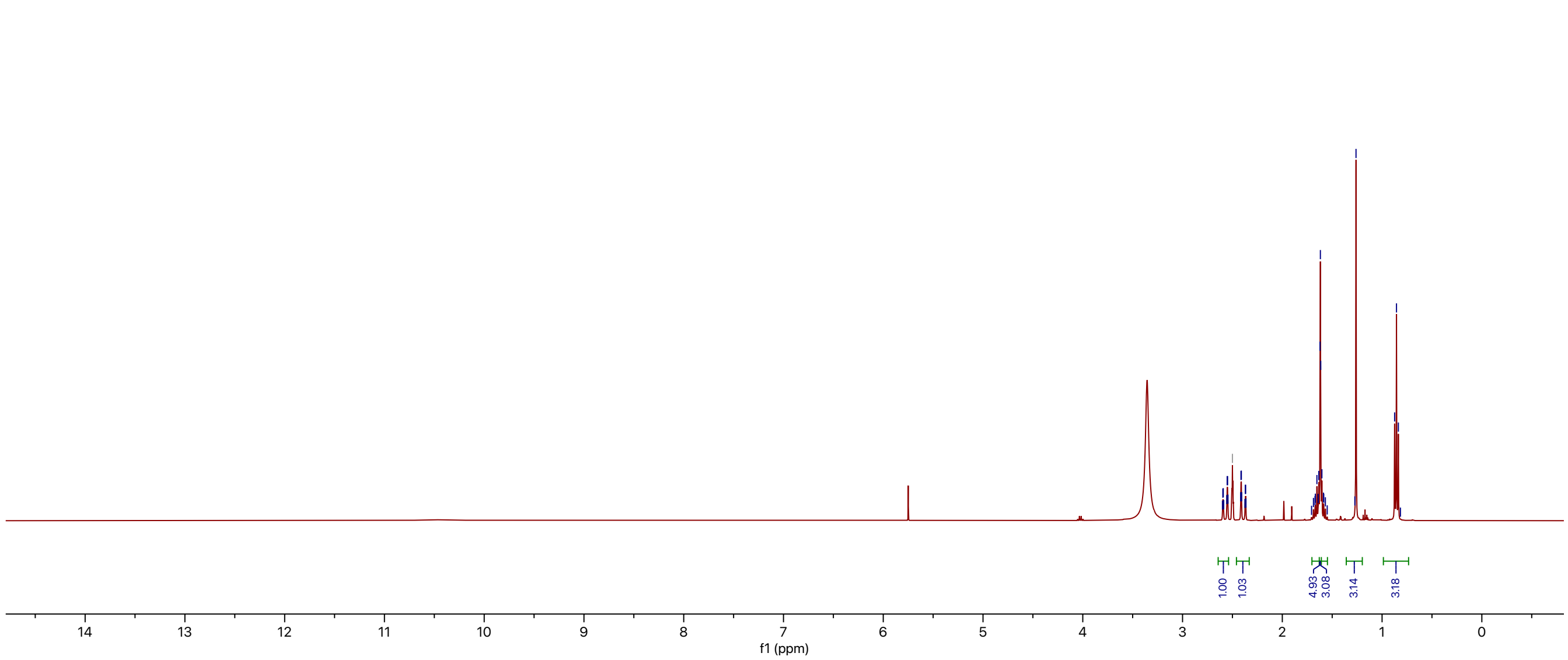

^{13}C NMR (101 MHz, DMSO)

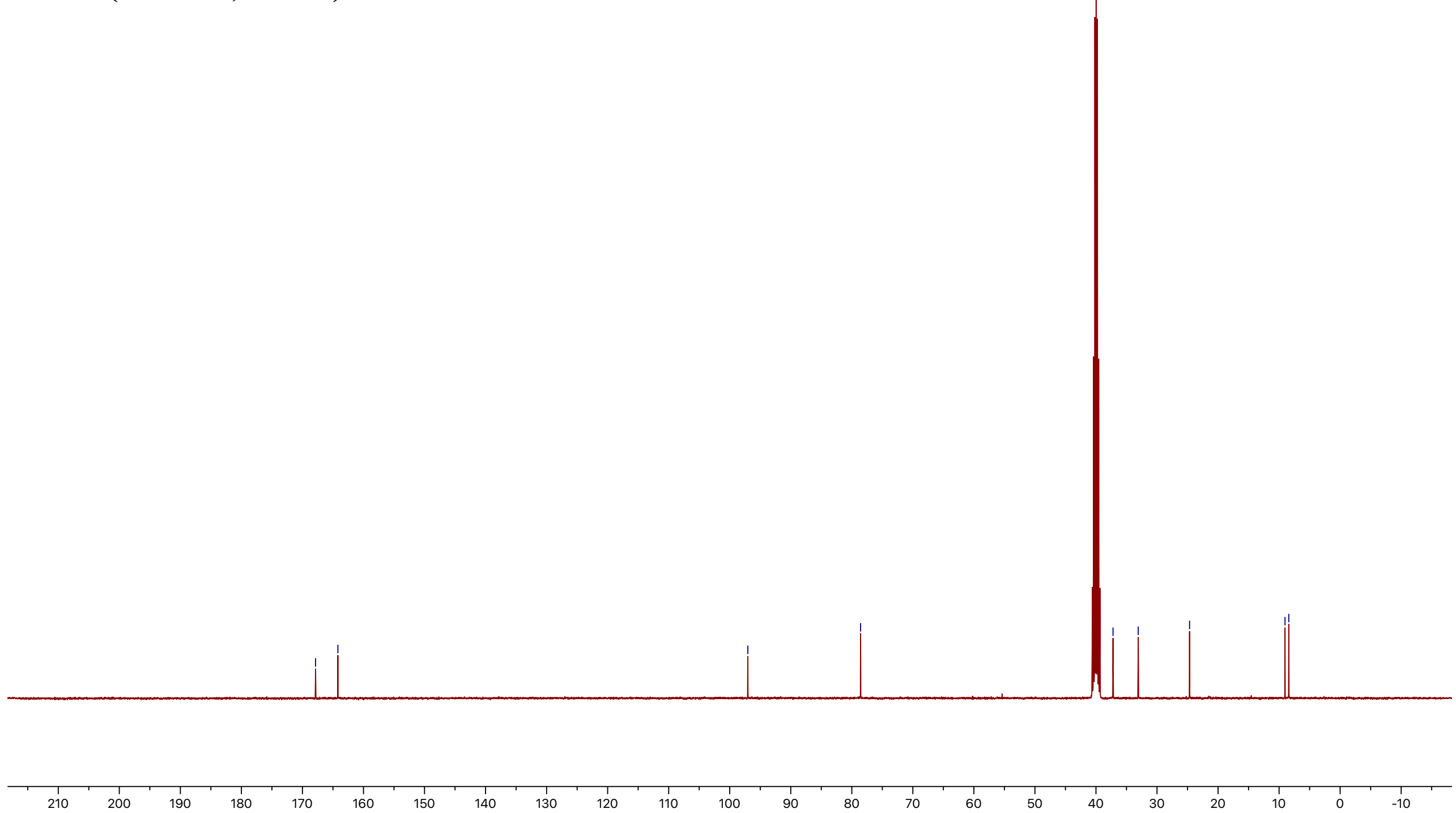

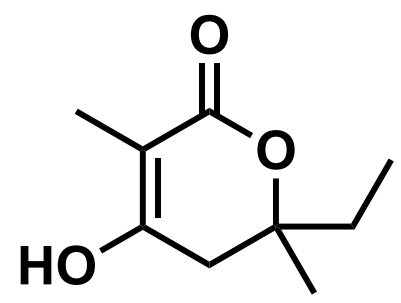

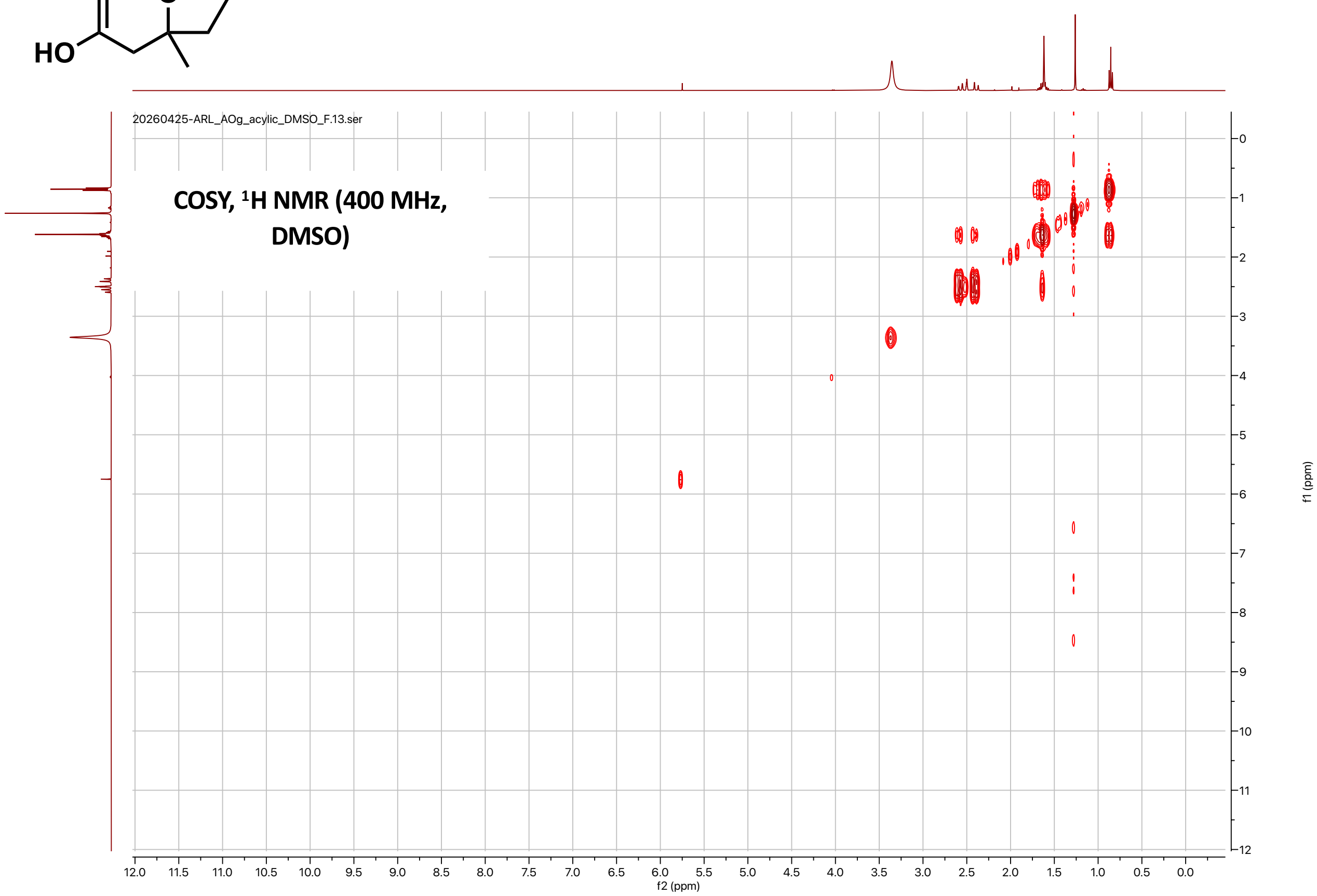
20260425-ARL_AOg_acylic_DMSO_F.13.ser
COSY, ¹H NMR (400 MHz, DMSO)
f2 (ppm)
f1 (ppm)

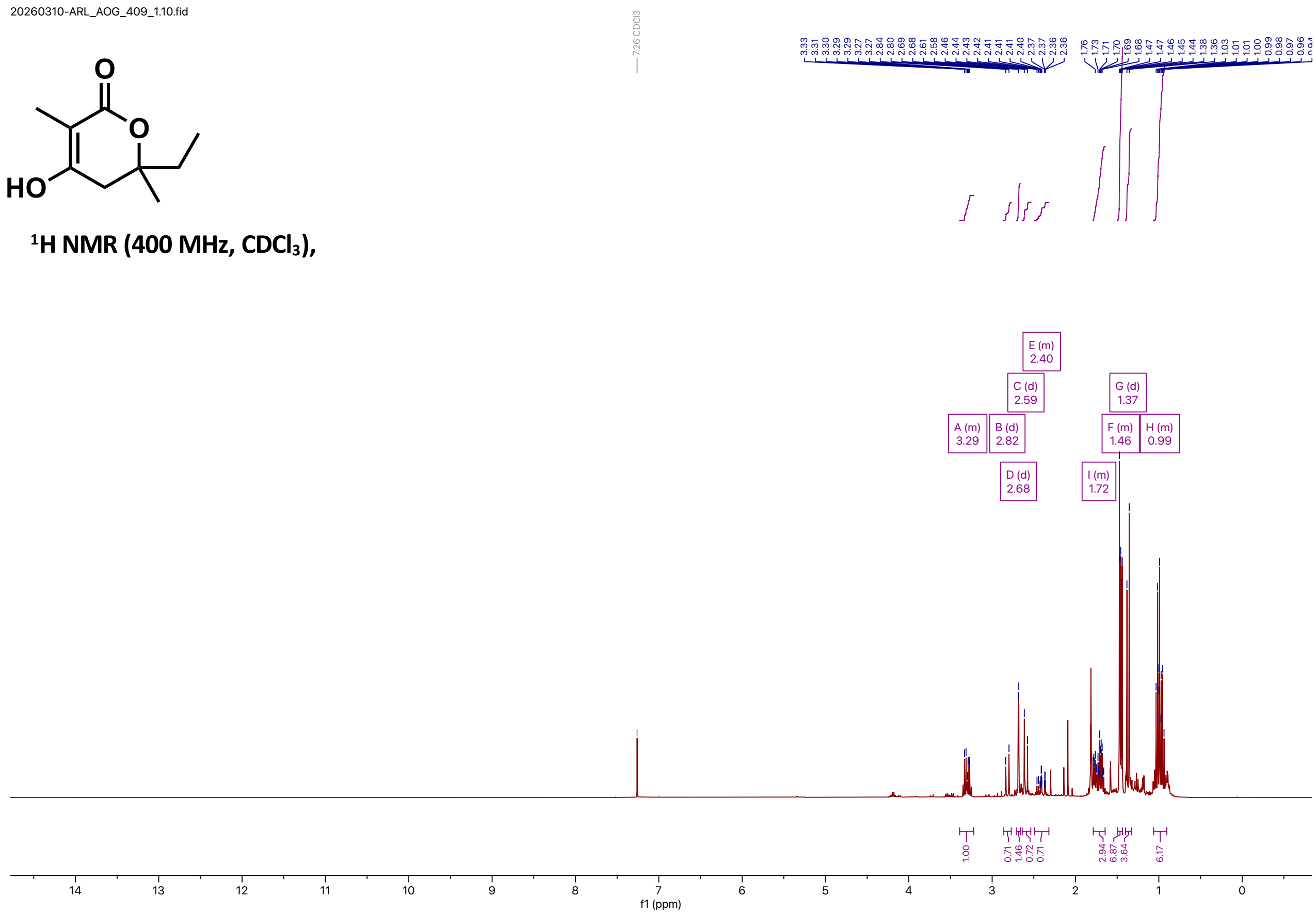

^{1}H NMR (400 MHz, $CDCl_3$),

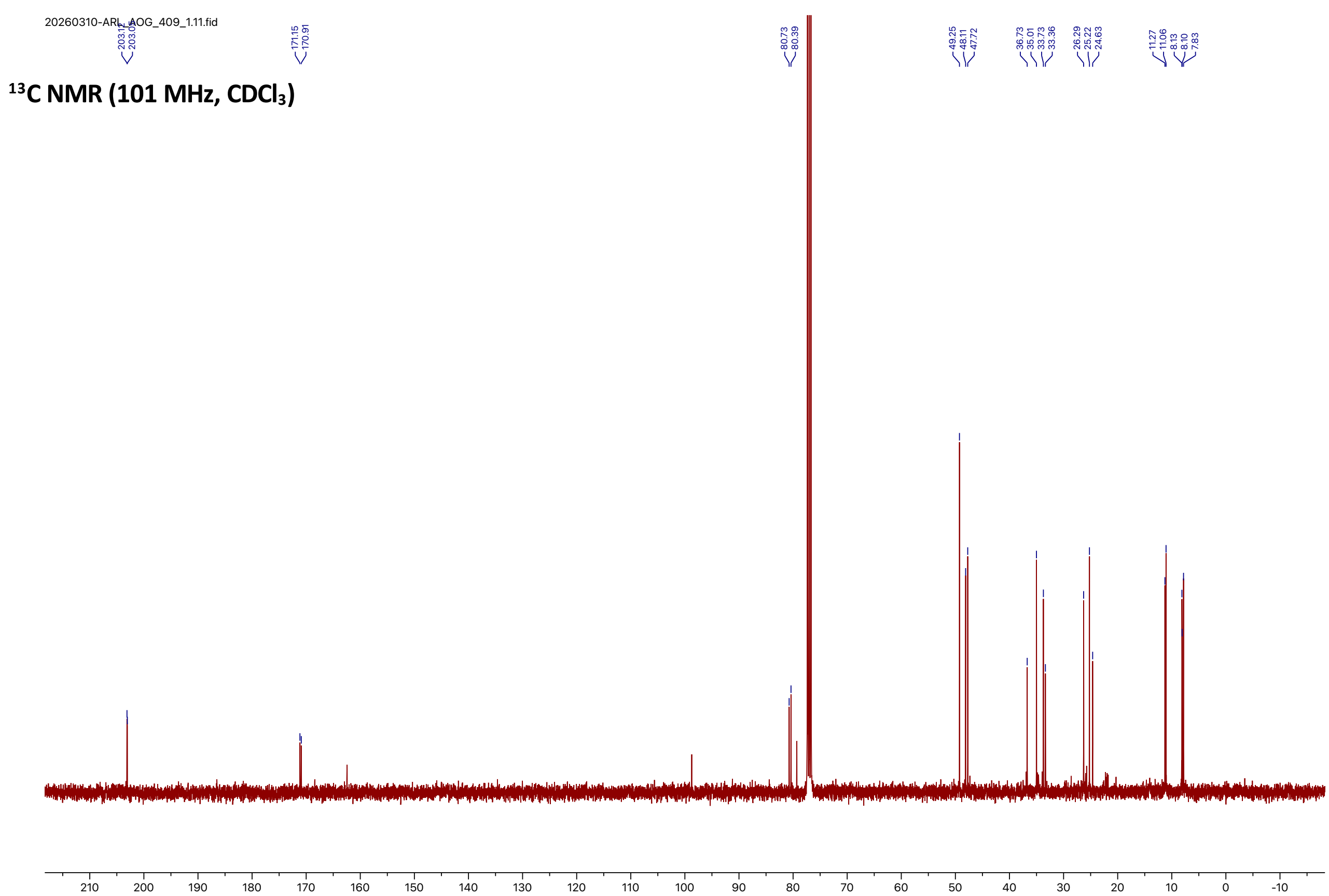

^{13}C NMR (101 MHz, $CDCl_3$)

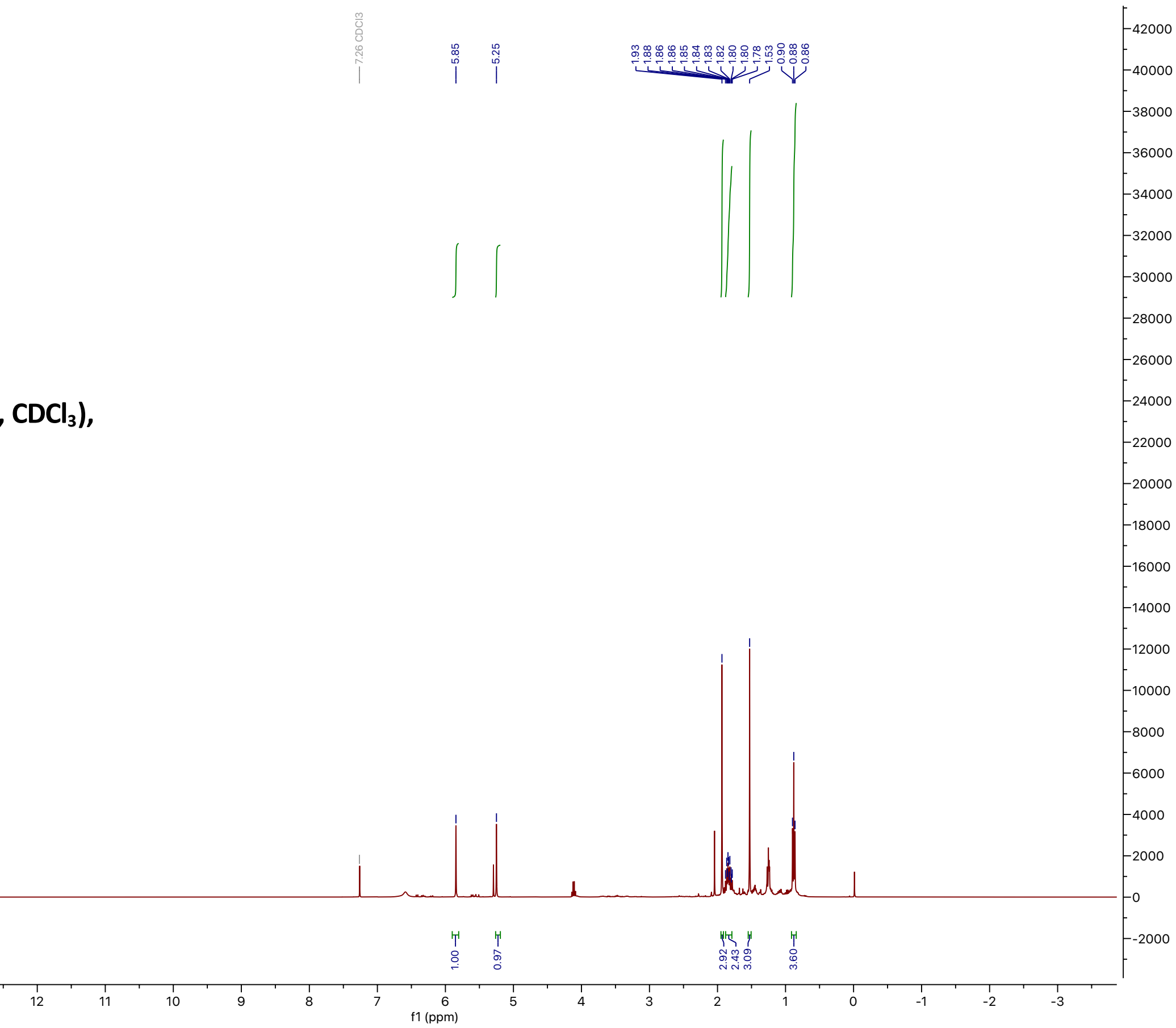

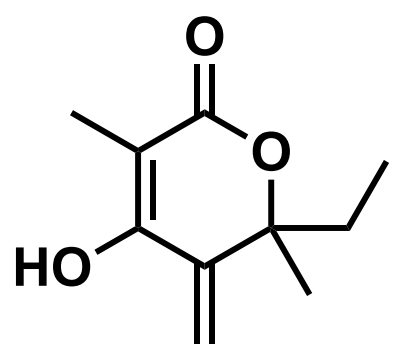

^{1}H NMR (400 MHz, $CDCl_3$),

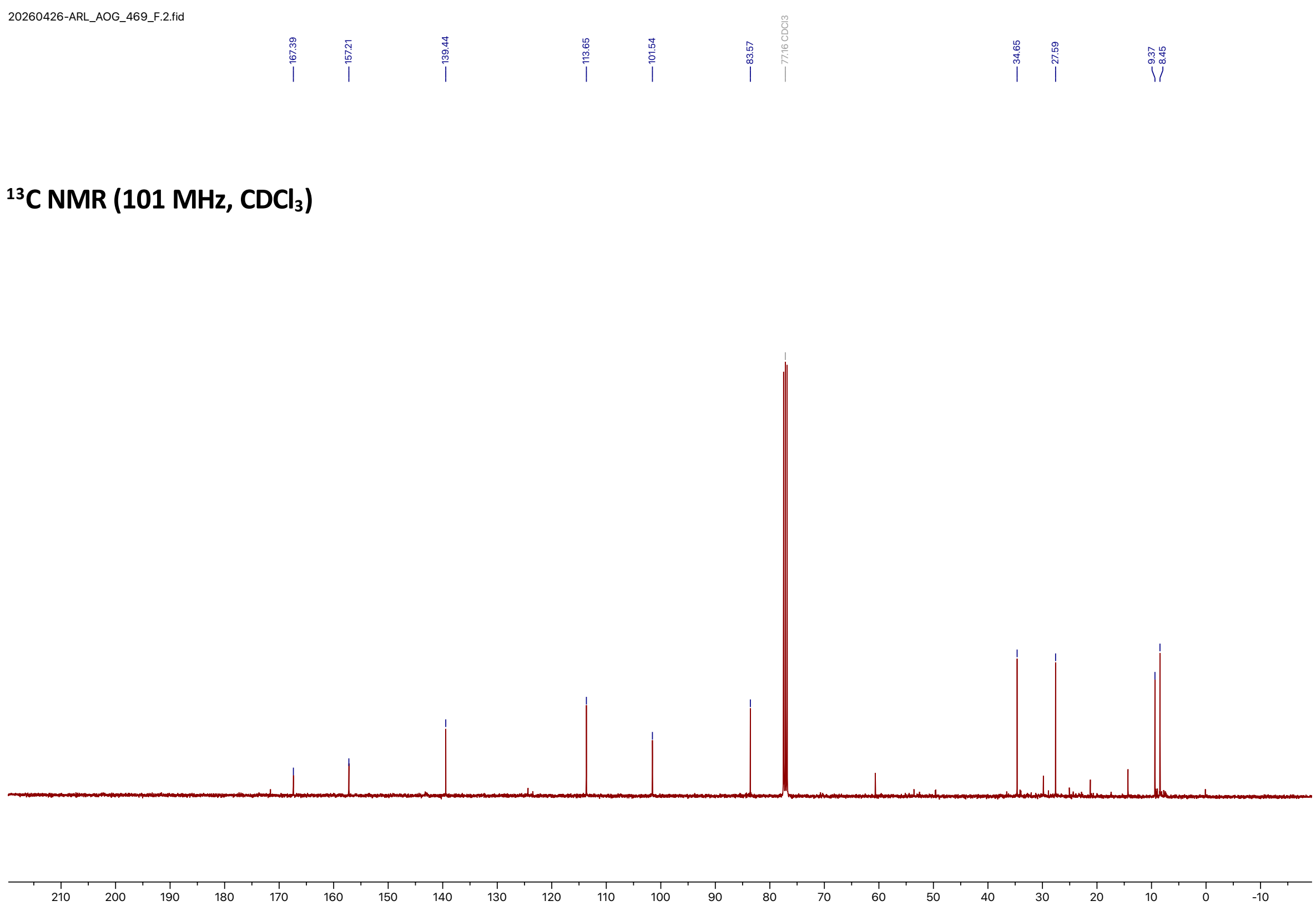

^{13}C NMR (101 MHz, $CDCl_3$)

20260308-ARL_AOG_401_F.1.fid

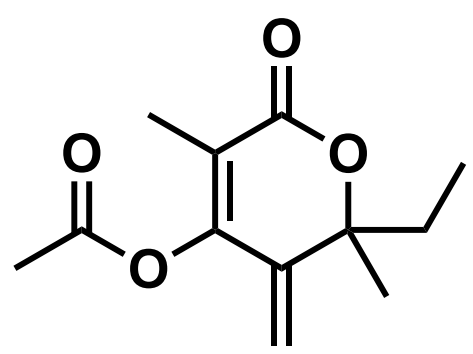

^{1}H NMR (400 MHz, $CDCl_3$),

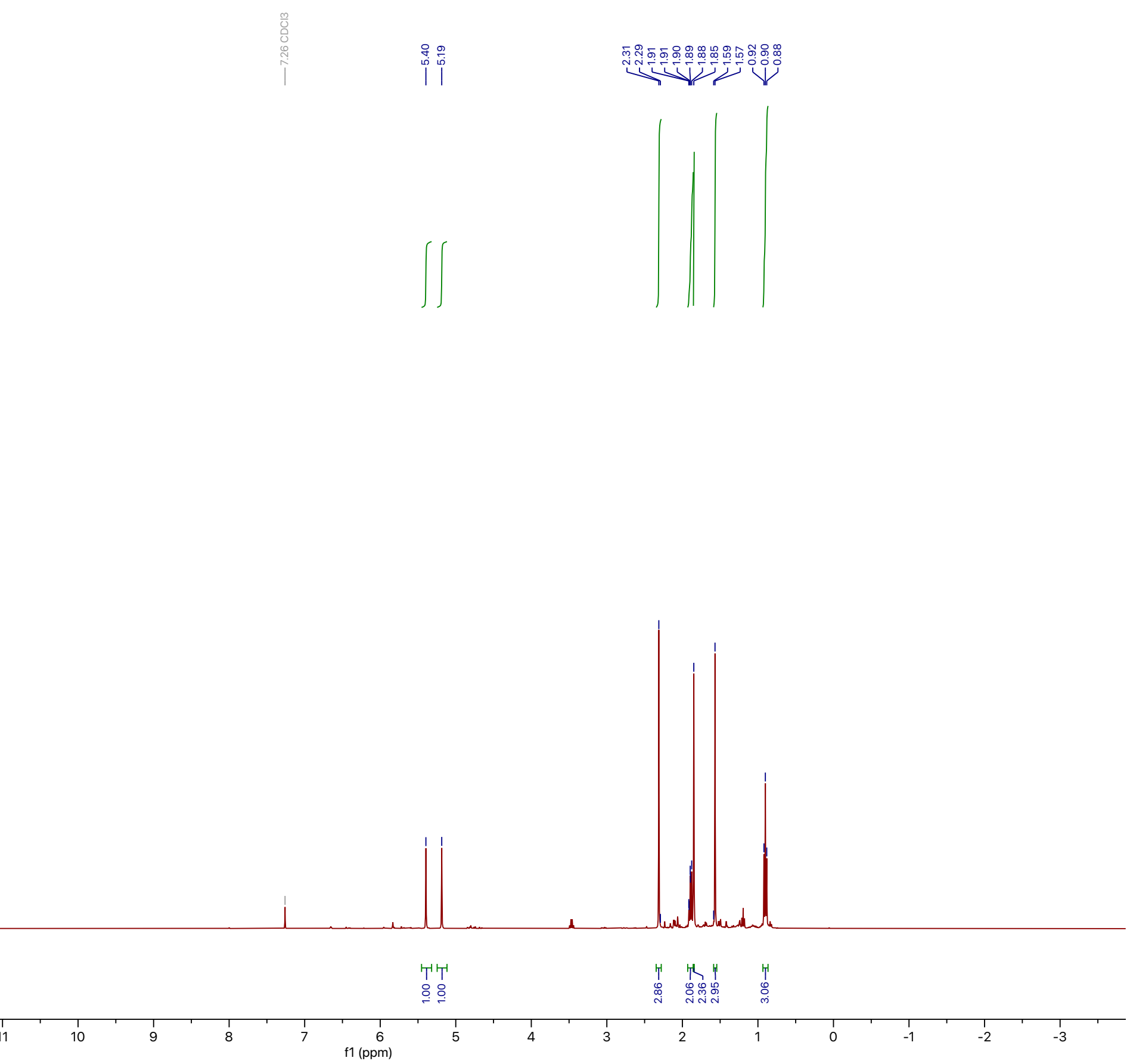

20260308-ARL_AOG_401_F.2.fid

^{13}C NMR (101 MHz, $CDCl_3$)

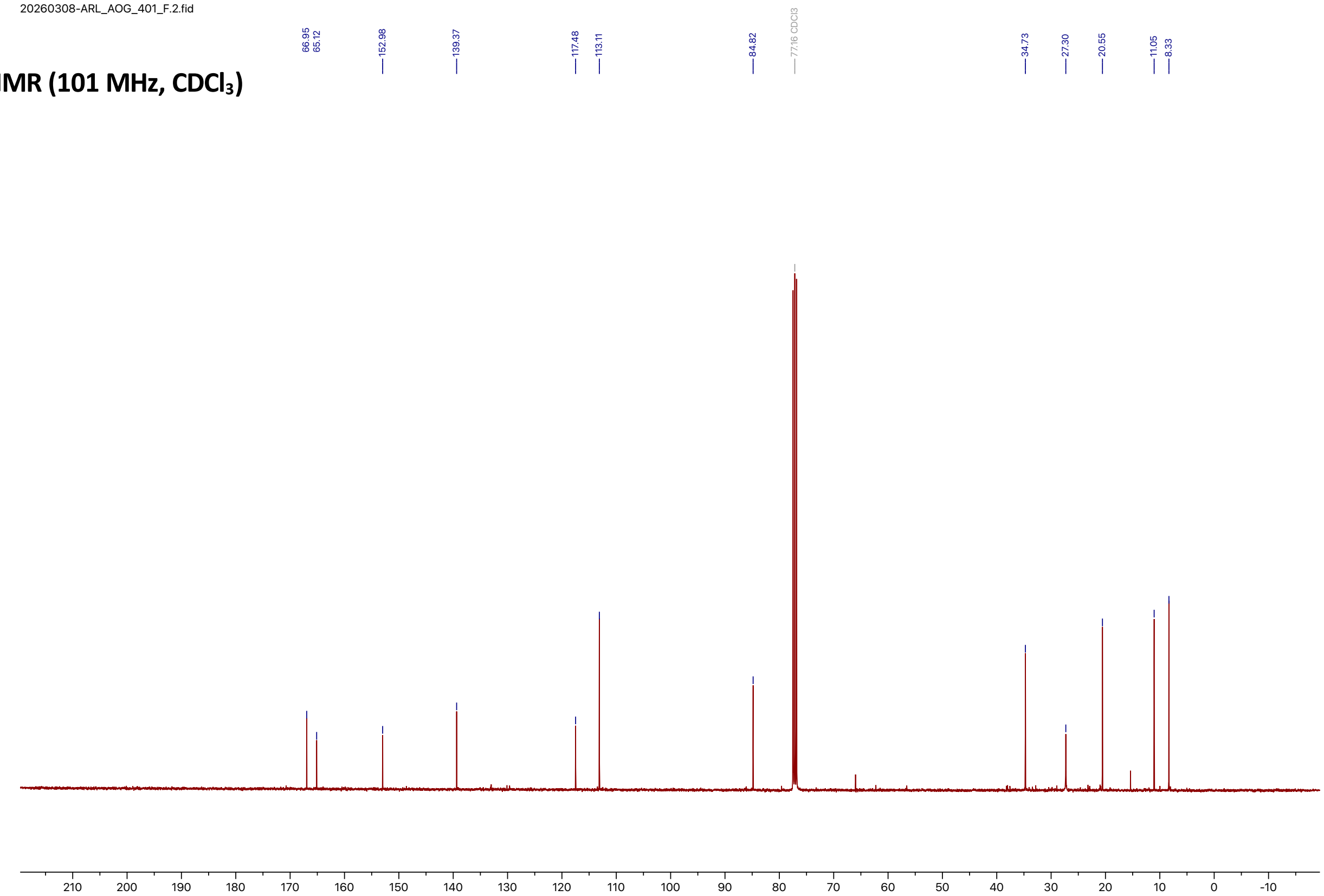

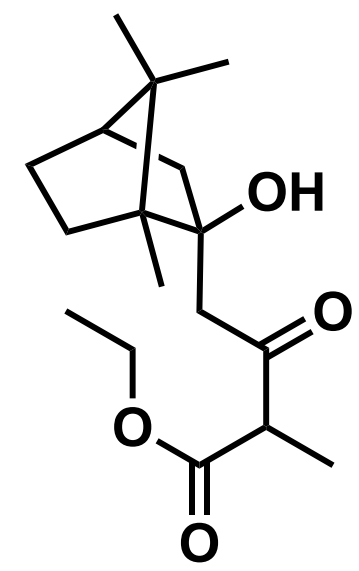

^{1}H NMR (400 MHz, $CDCl_3$),

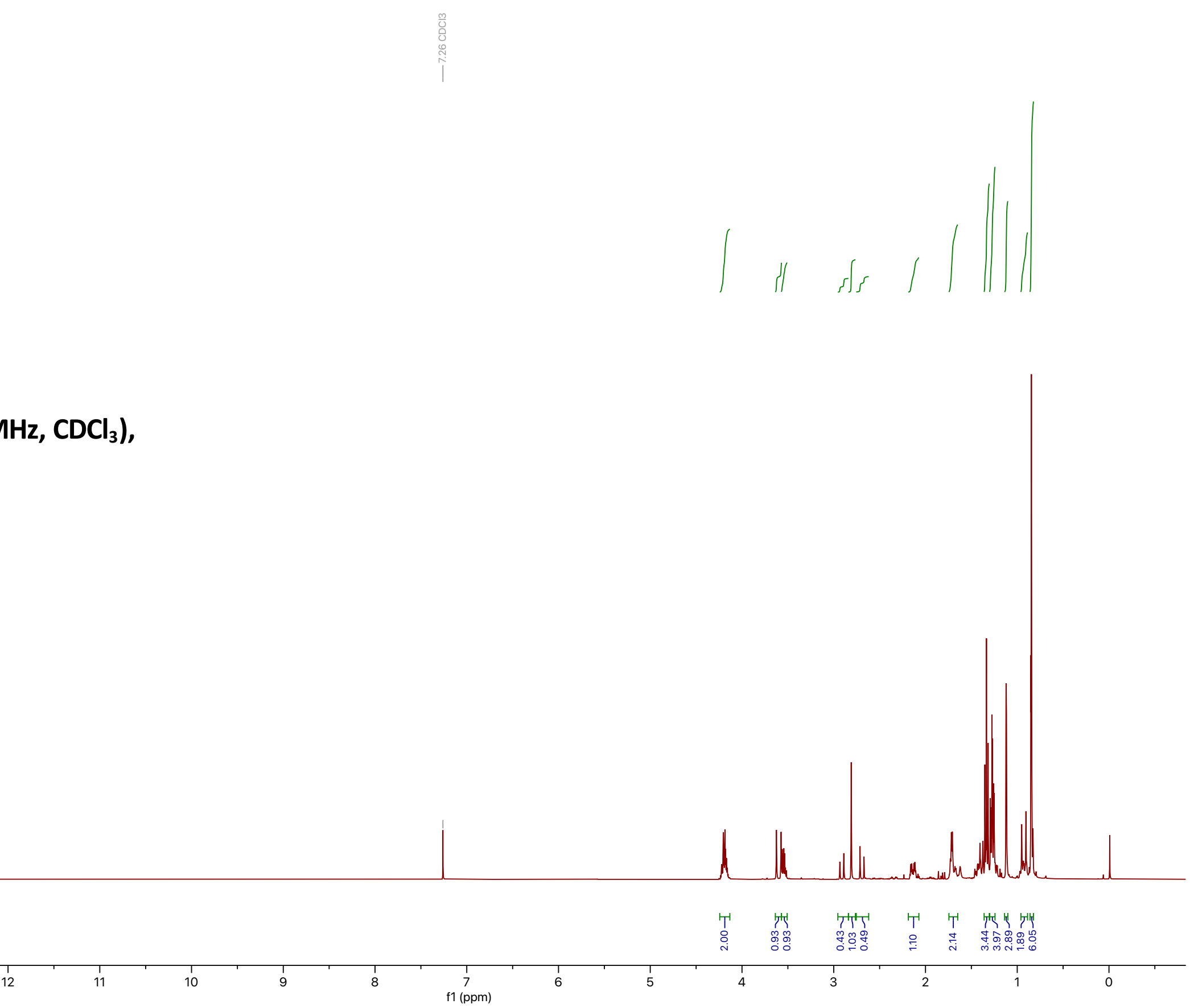

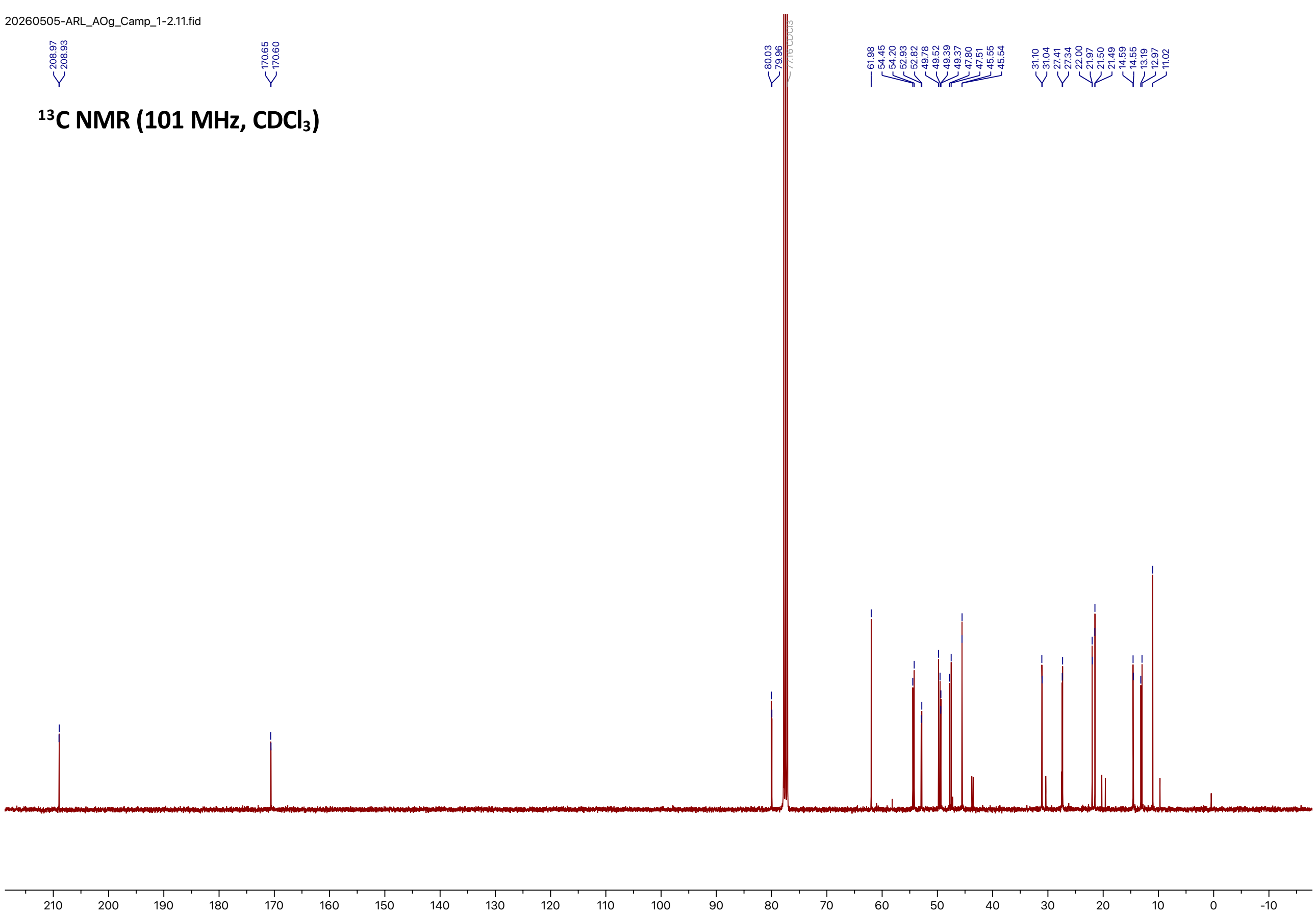

^{13}C NMR (101 MHz, $CDCl_3$)

20260308-ARL_AOG_404_1.10.fid

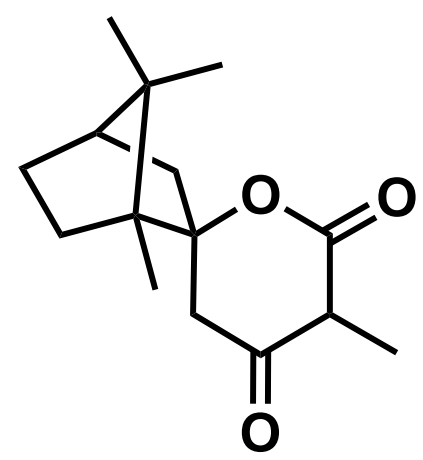

^{1}H NMR (400 MHz, $CDCl_3$),

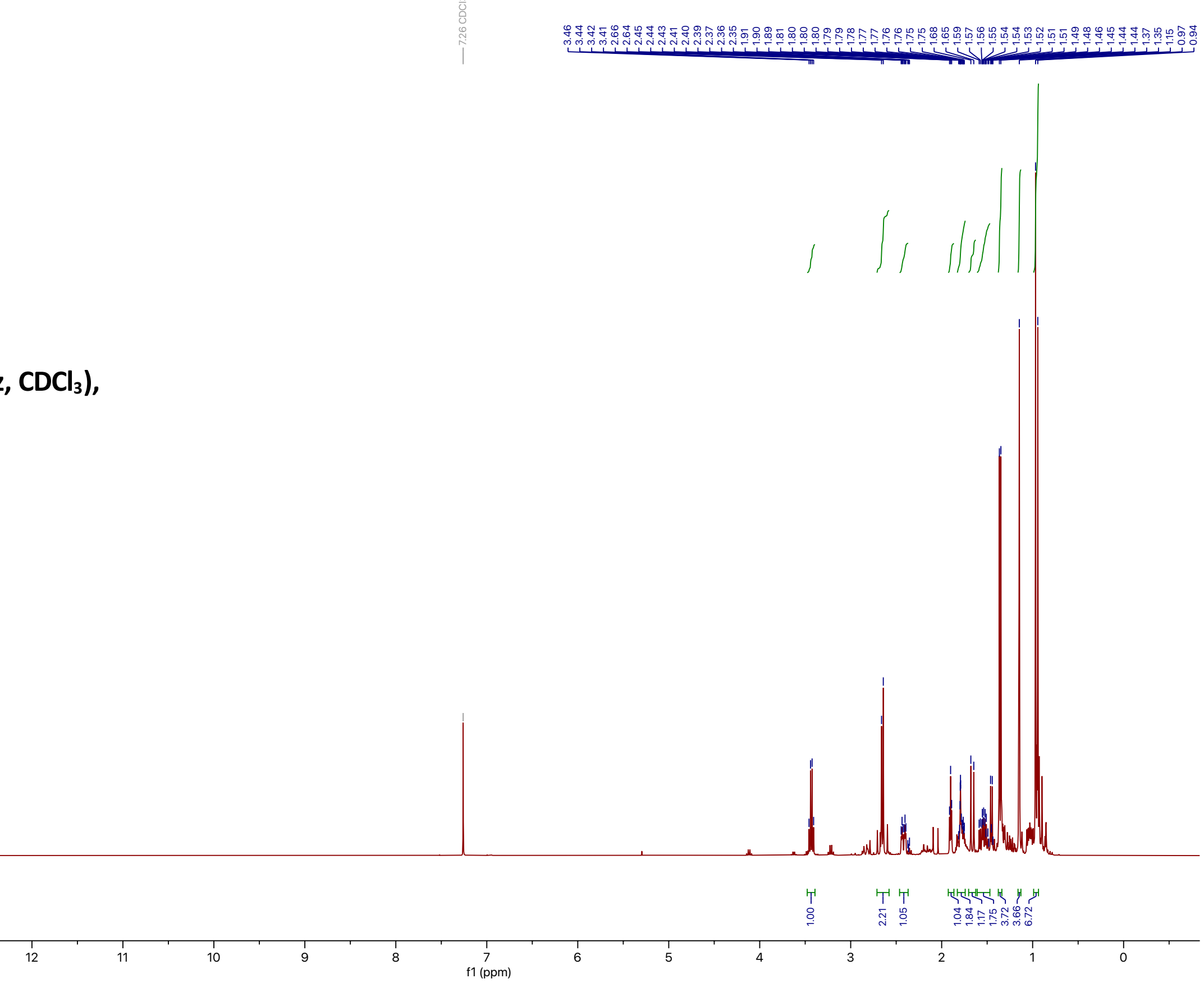

20260308-ARL_AOG_404_1.11.fid

^{13}C NMR (101 MHz, $CDCl_3$)

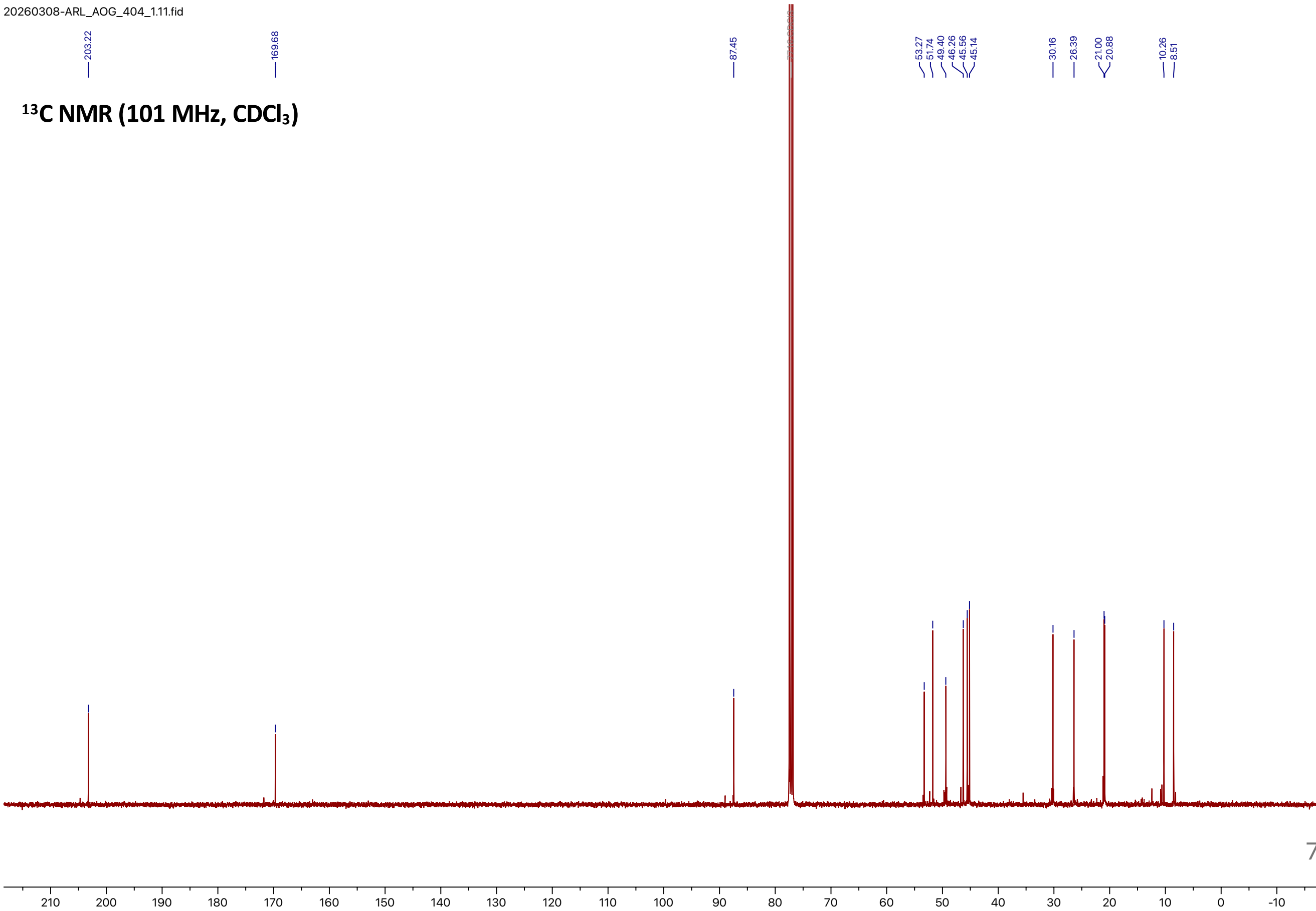

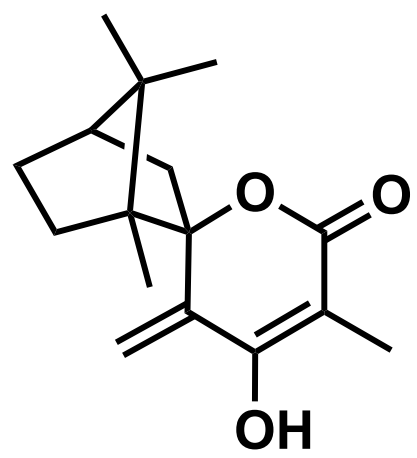

^{1}H NMR (400 MHz, $CDCl_3$),

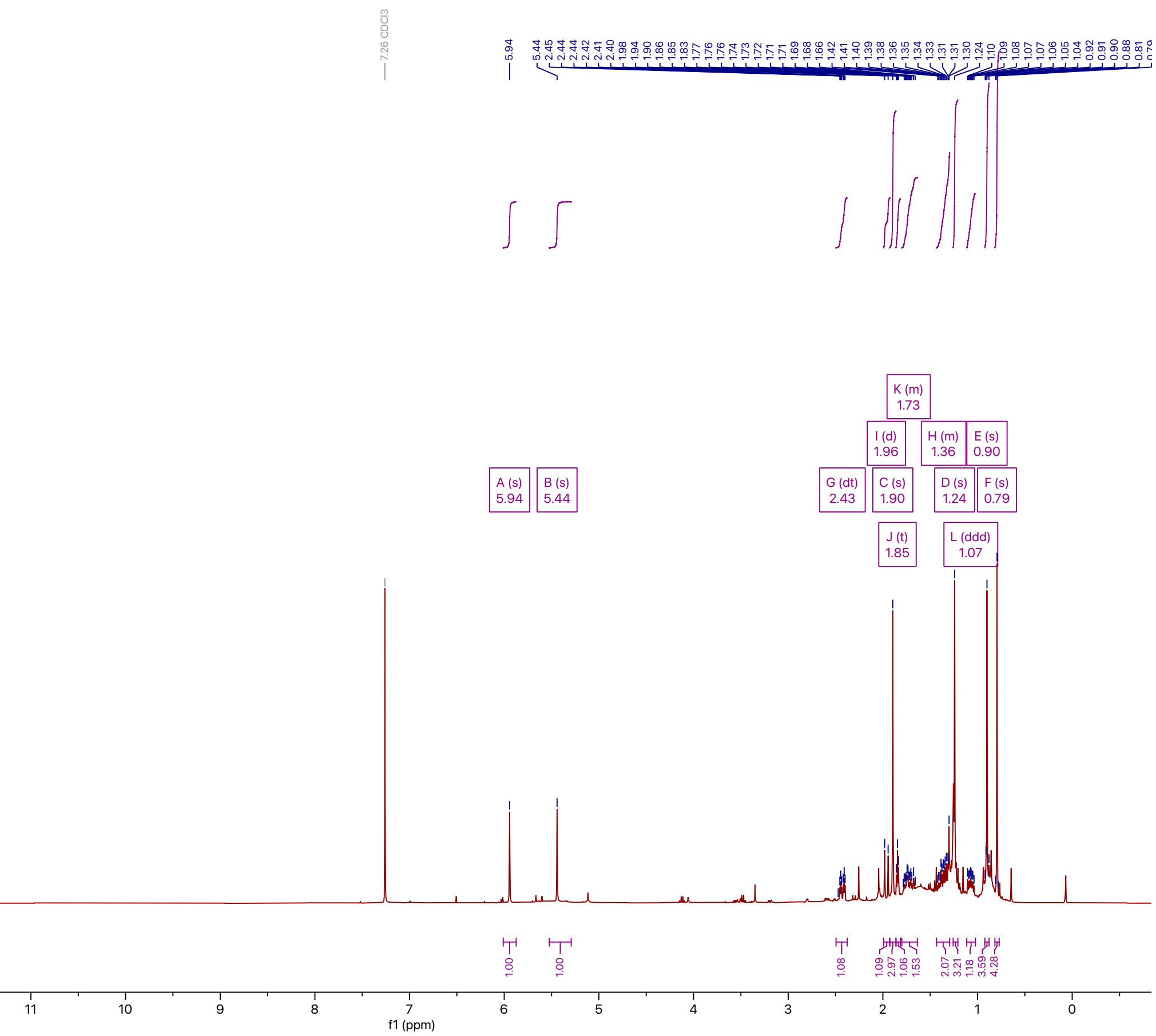

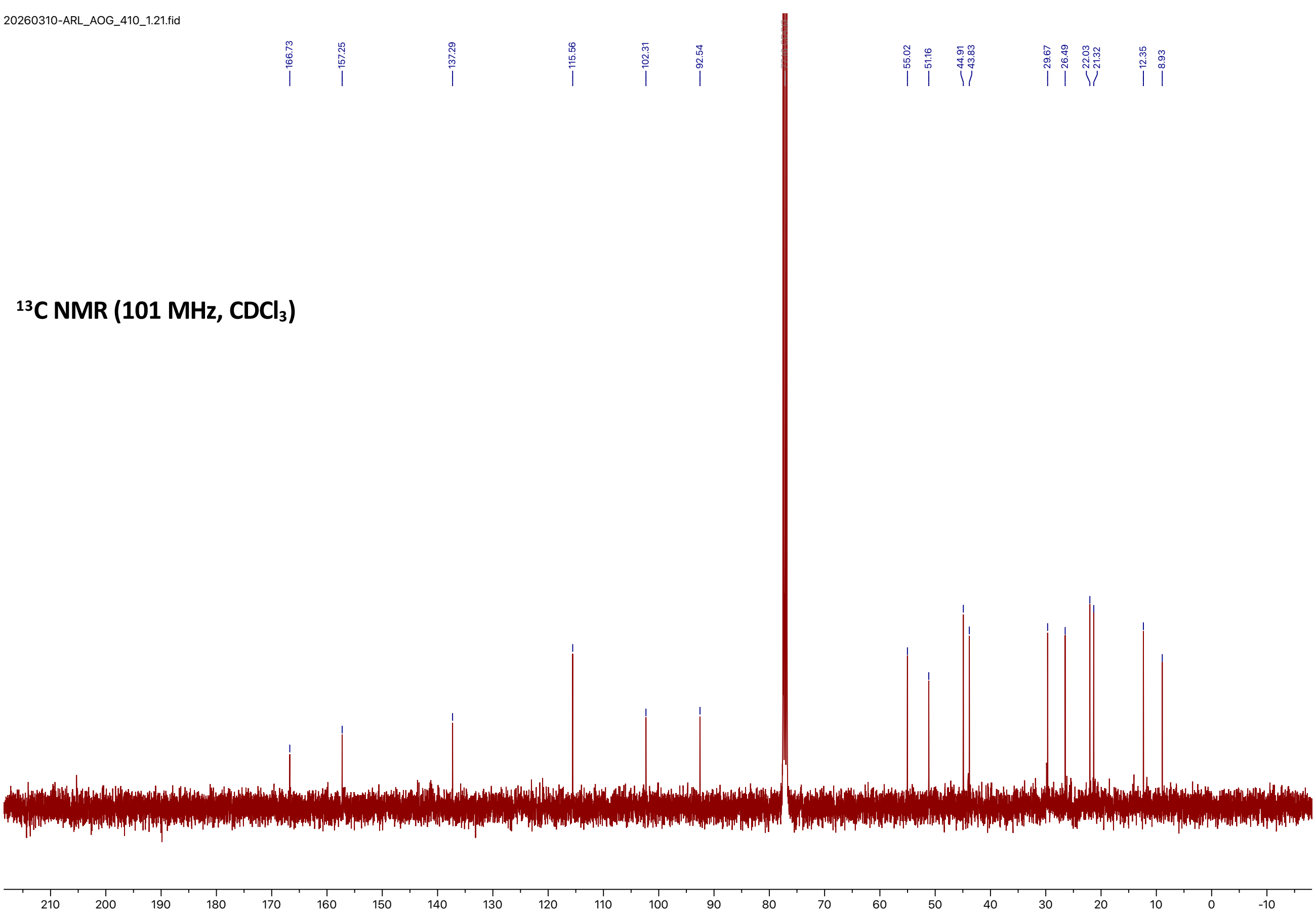

^{13}C NMR (101 MHz, $CDCl_3$)

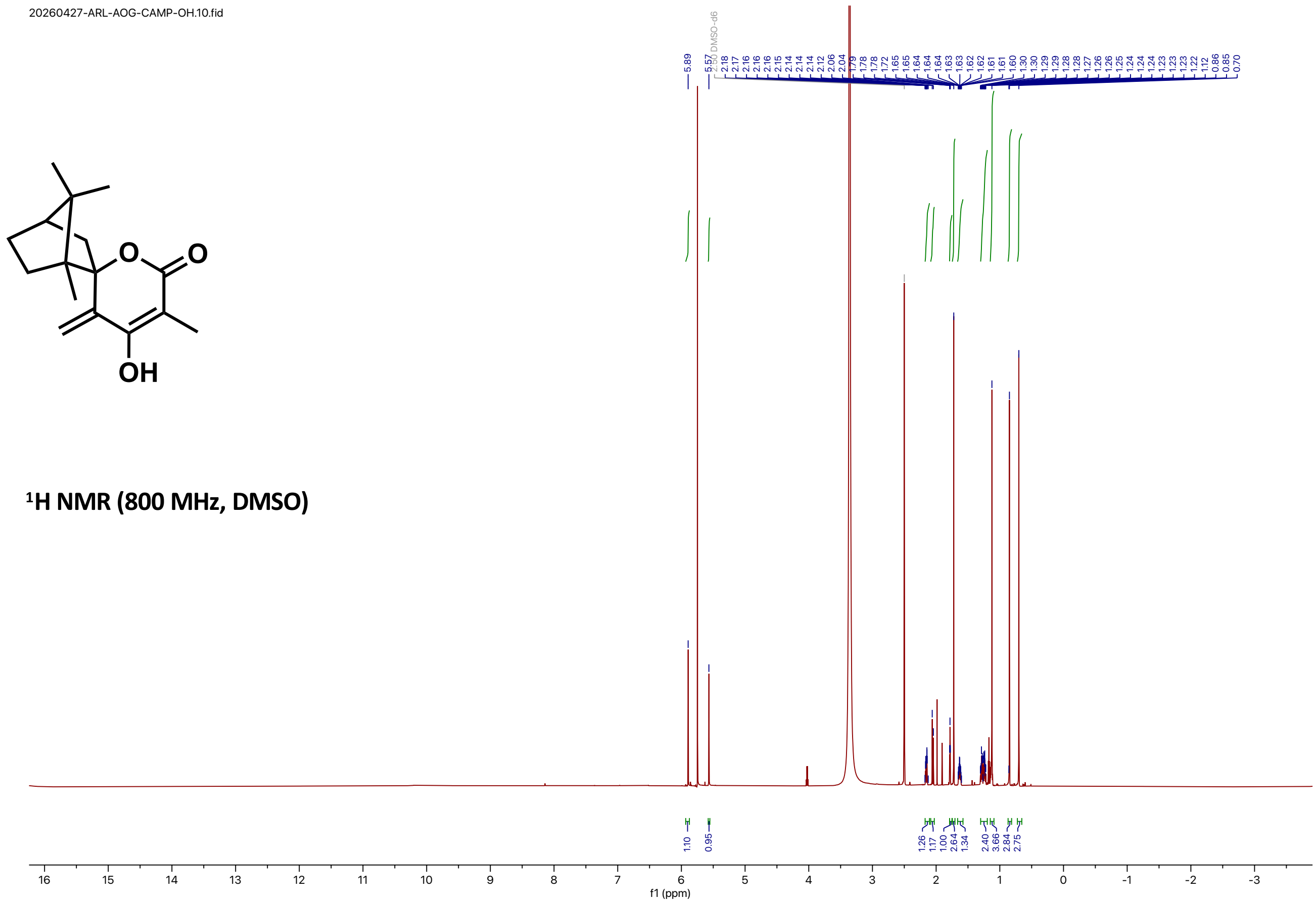

¹H NMR (800 MHz, DMSO)

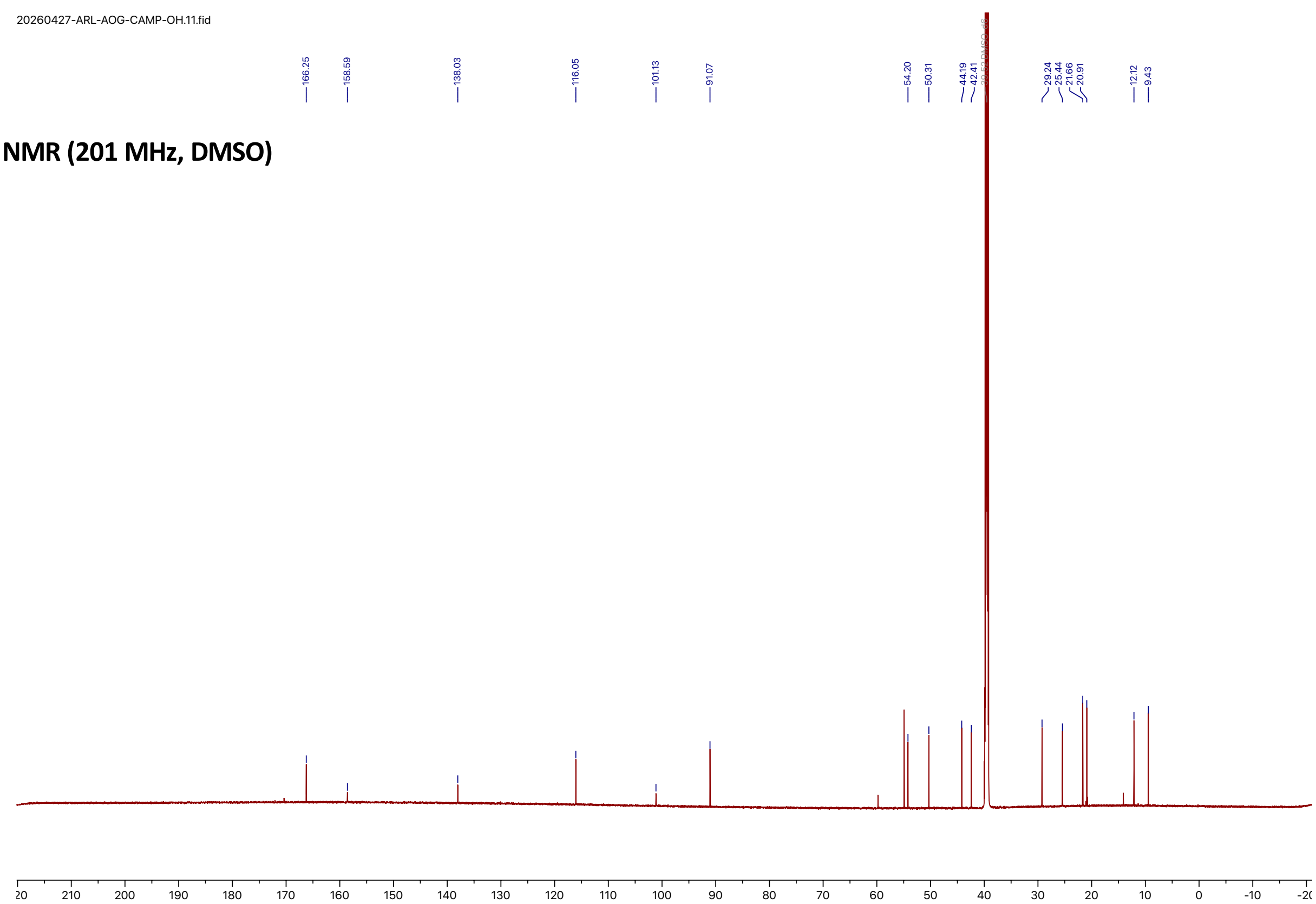

¹³C NMR (201 MHz, DMSO)

20260327-ARL_AOG_431_F_1.1.fid

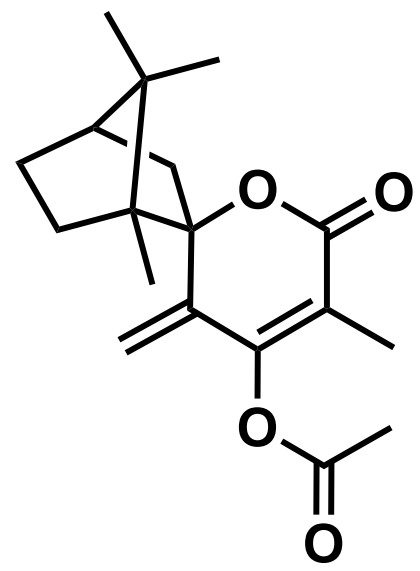

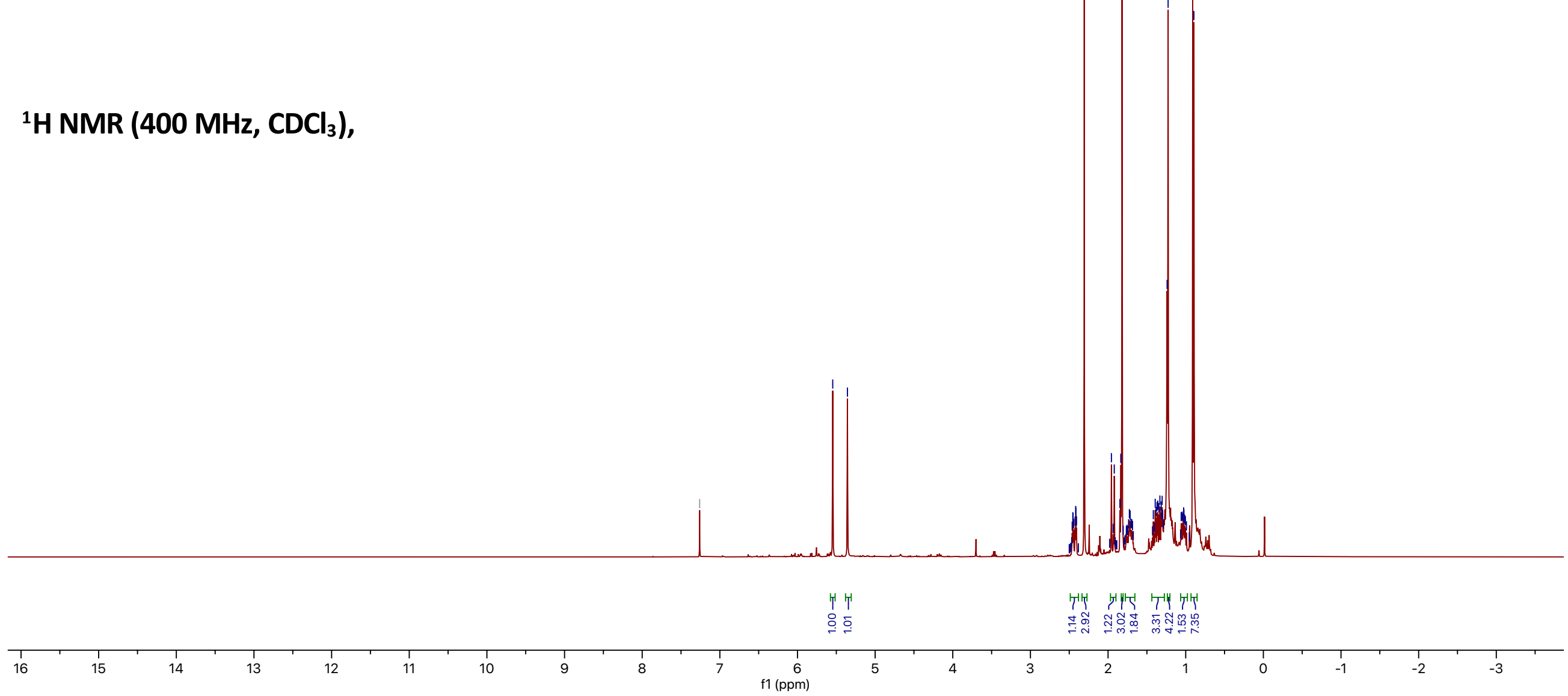

^{1}H NMR (400 MHz, $CDCl_3$),

20260327-ARL_AOG_431_F_1.2.fid

^{13}C NMR (101 MHz, $CDCl_3$)

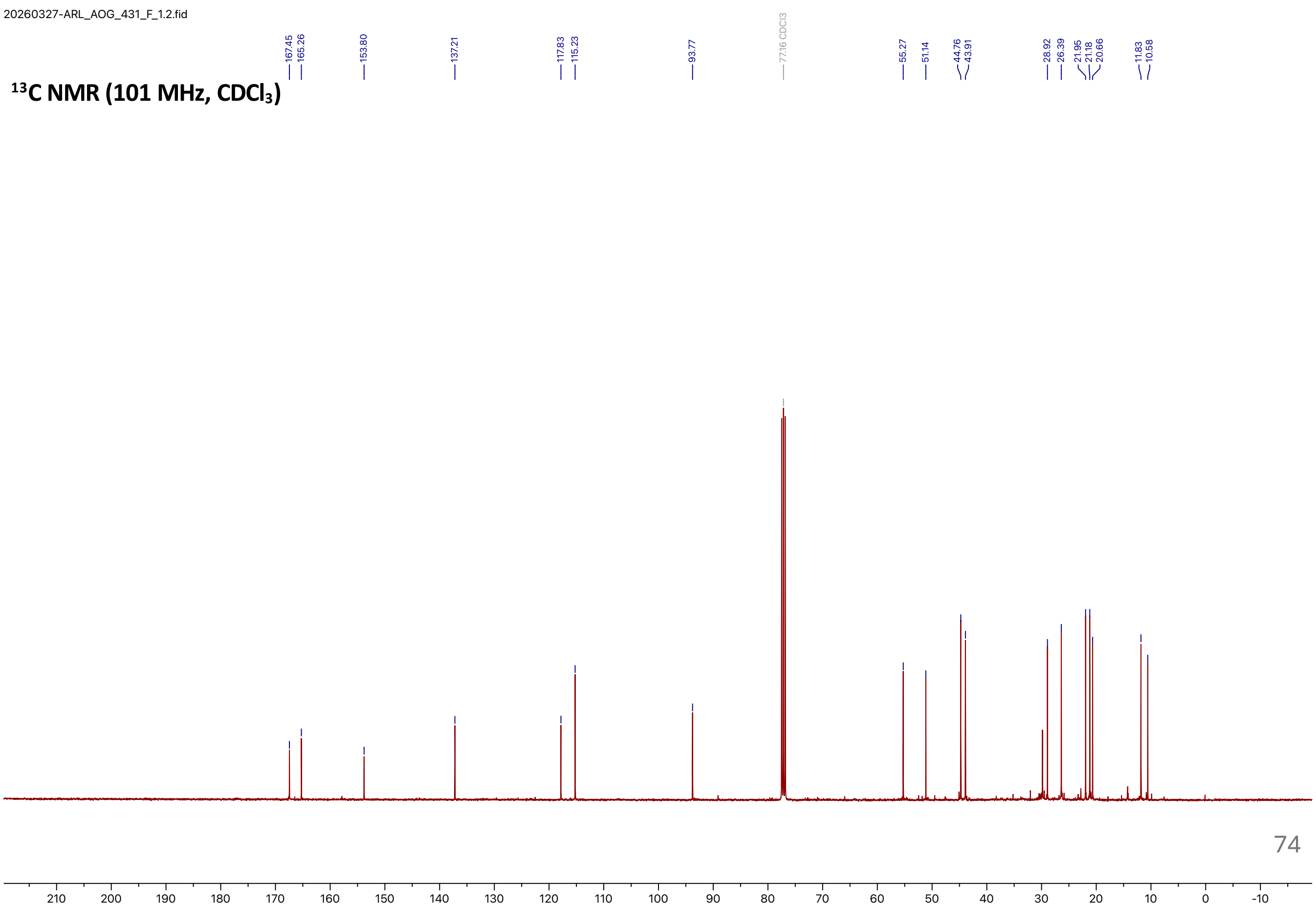

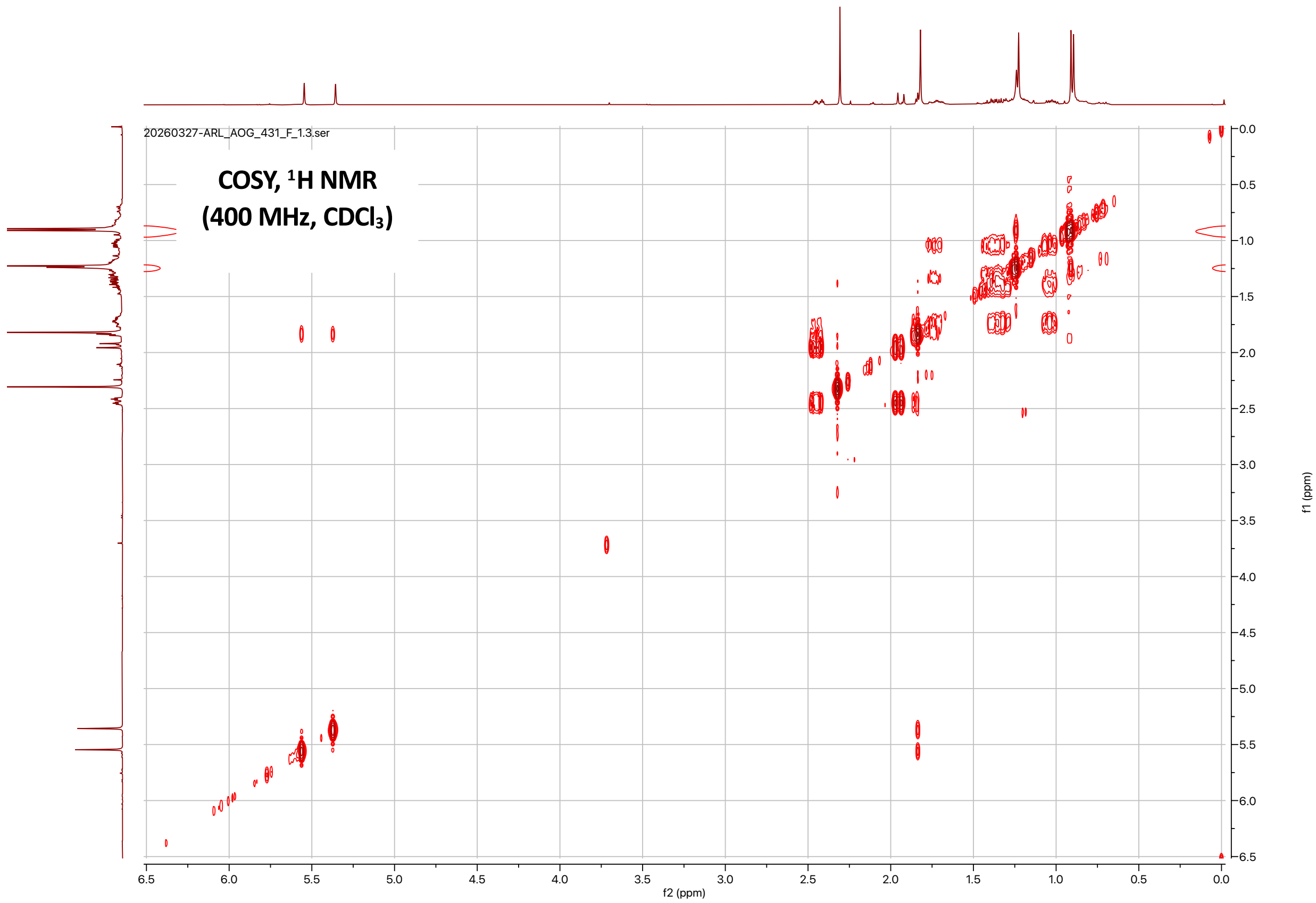
20260327-ARL_AOG_431_F_1.3.ser
COSY, ¹H NMR
(400 MHz, CDCl₃)
f2 (ppm)
f1 (ppm)

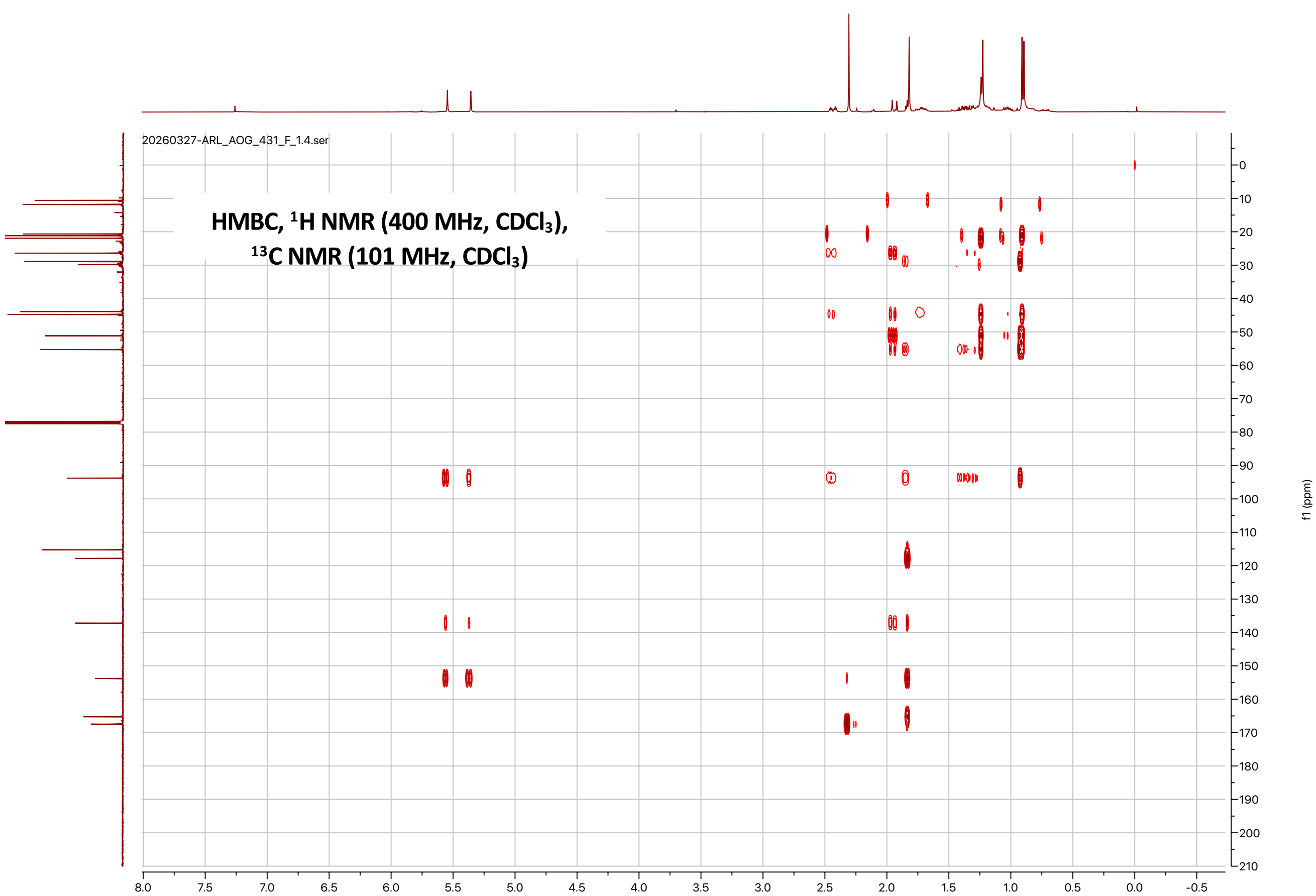
20260327-ARL_AOG_431_F_1.4.ser
HMBC, ¹H NMR (400 MHz, CDCl₃),
¹³C NMR (101 MHz, CDCl₃)
f2 (ppm)
f1 (ppm)

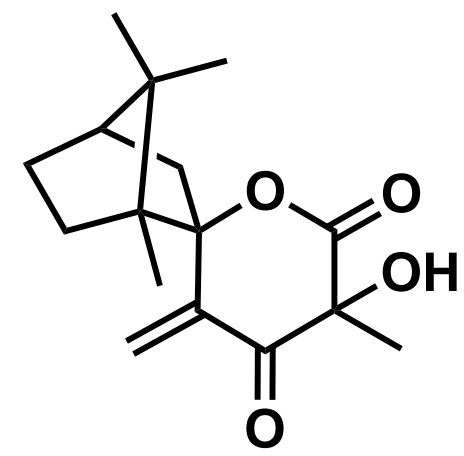

^1H NMR (400 MHz, $CDCl_3$),

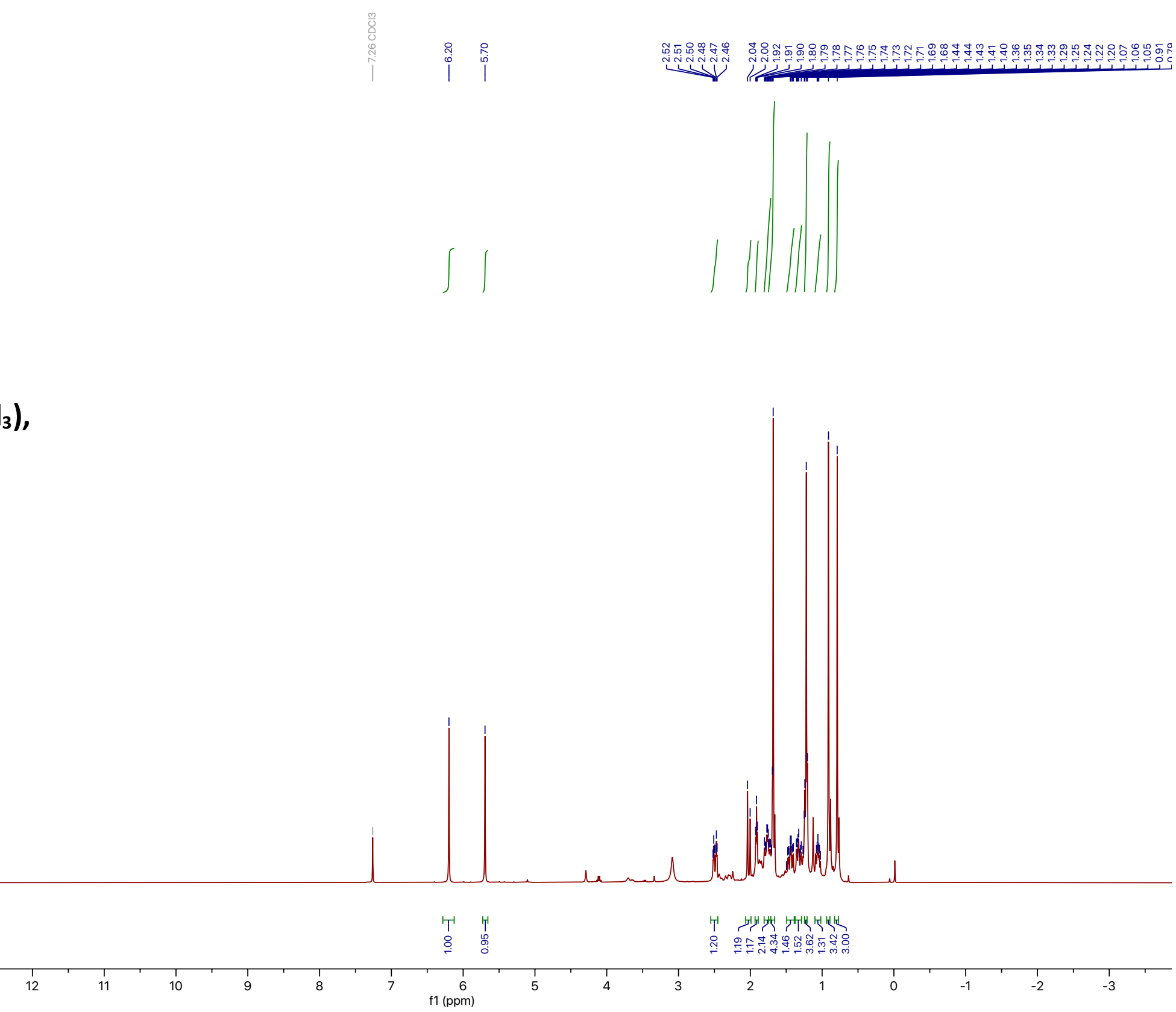

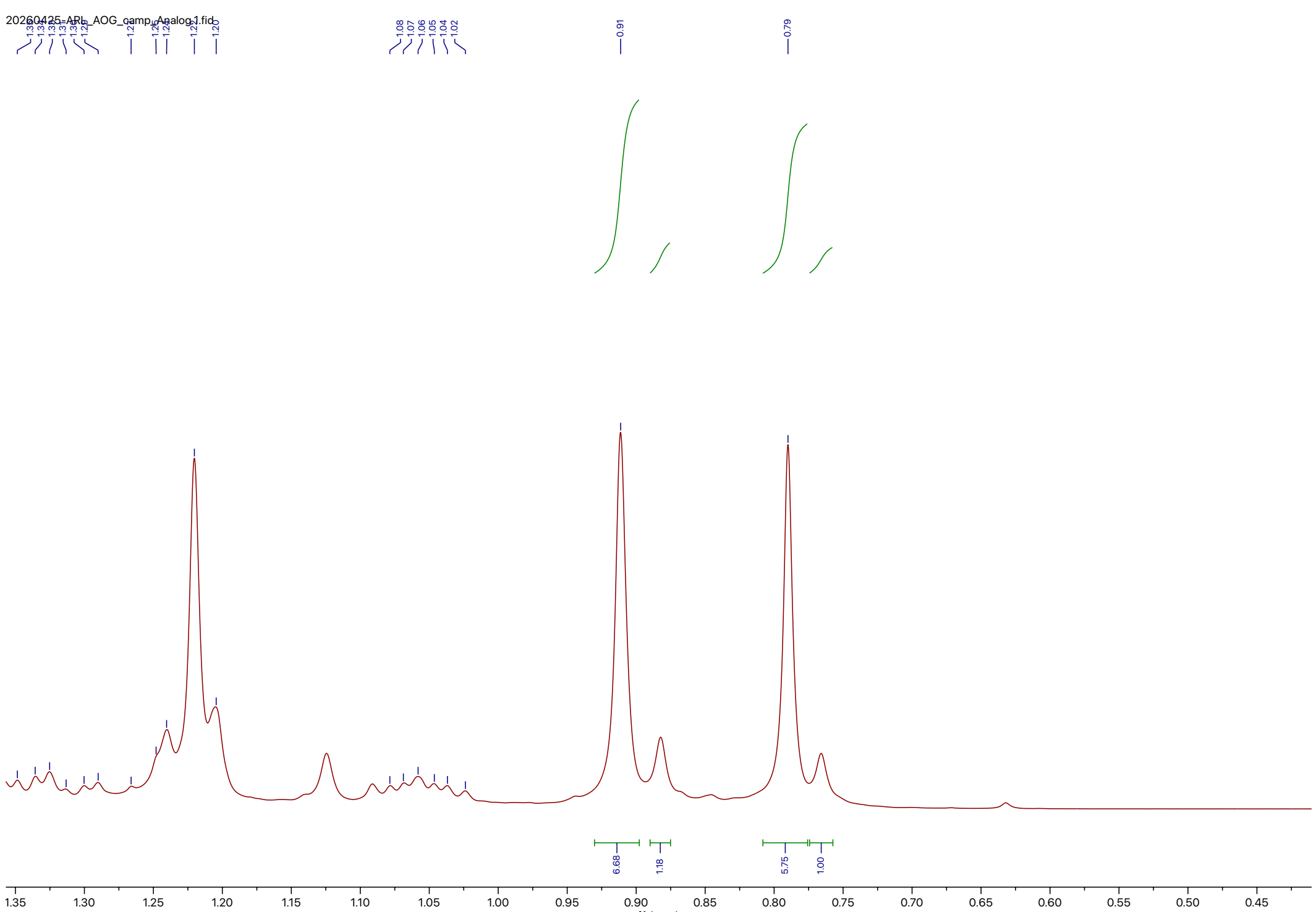

^{13}C NMR (101 MHz, CDCl$_3$)

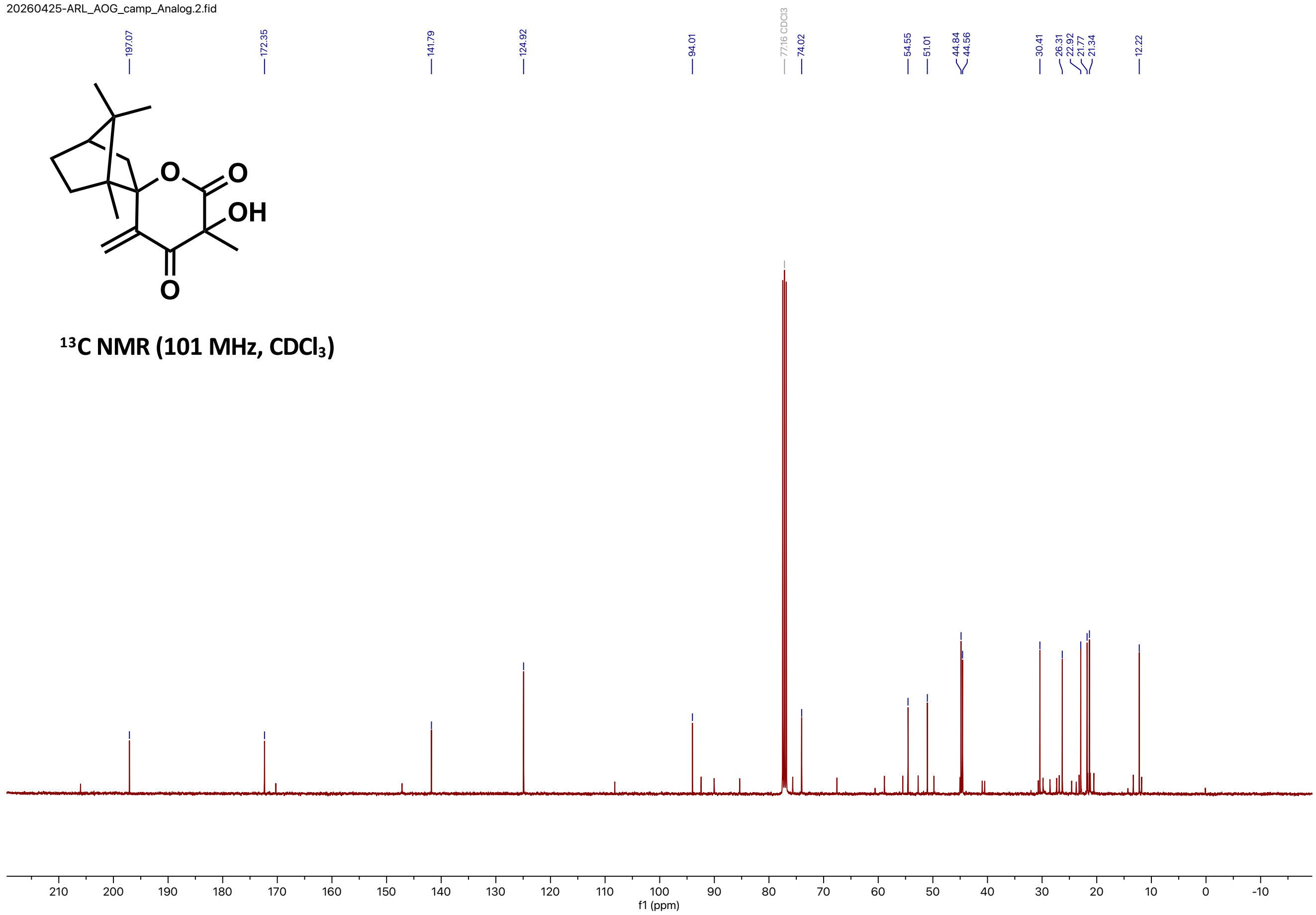

HMBC, ^{1}H NMR (400 MHz, CDCl$_3$),
^{13}C NMR (101 MHz, CDCl$_3$)

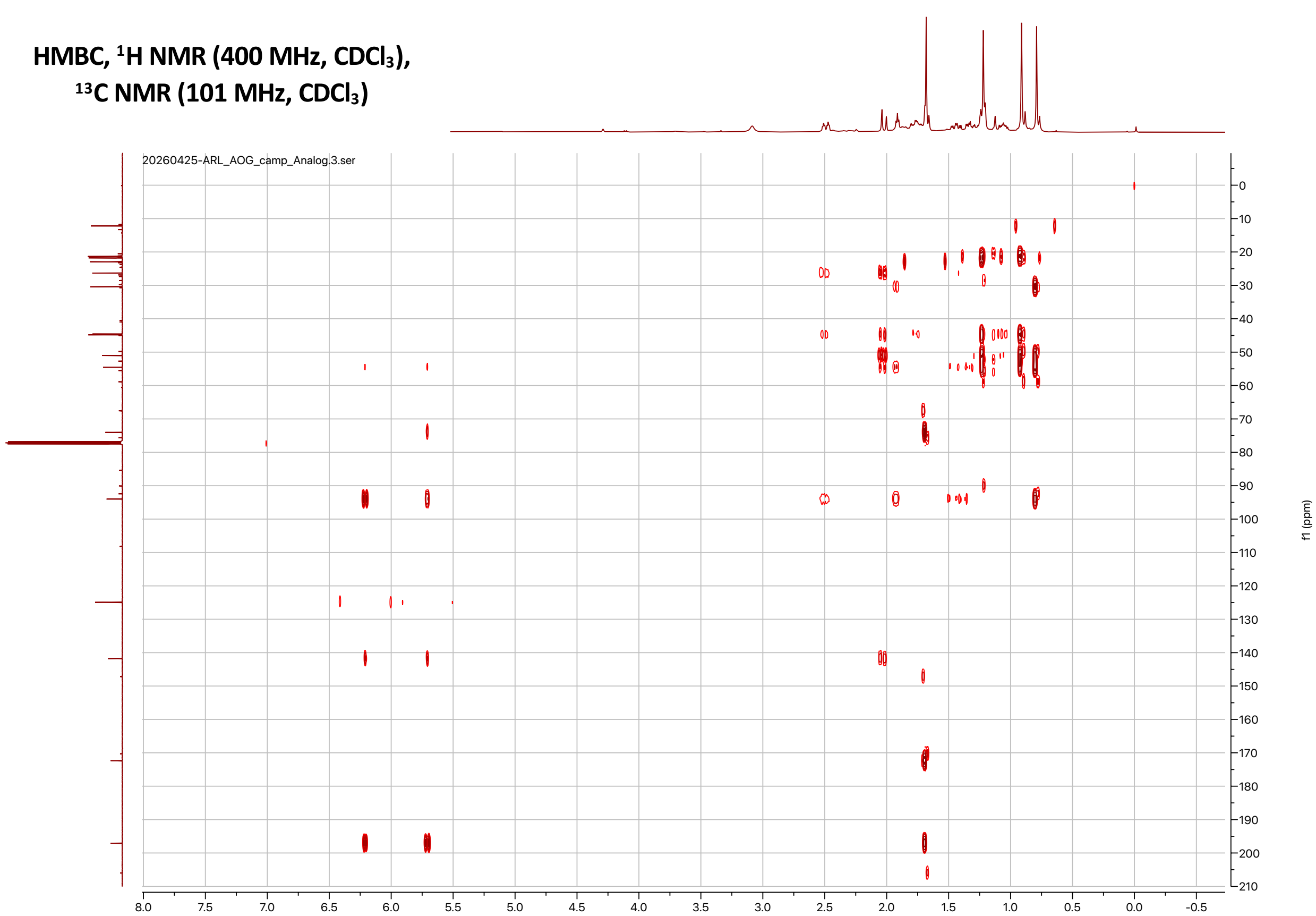

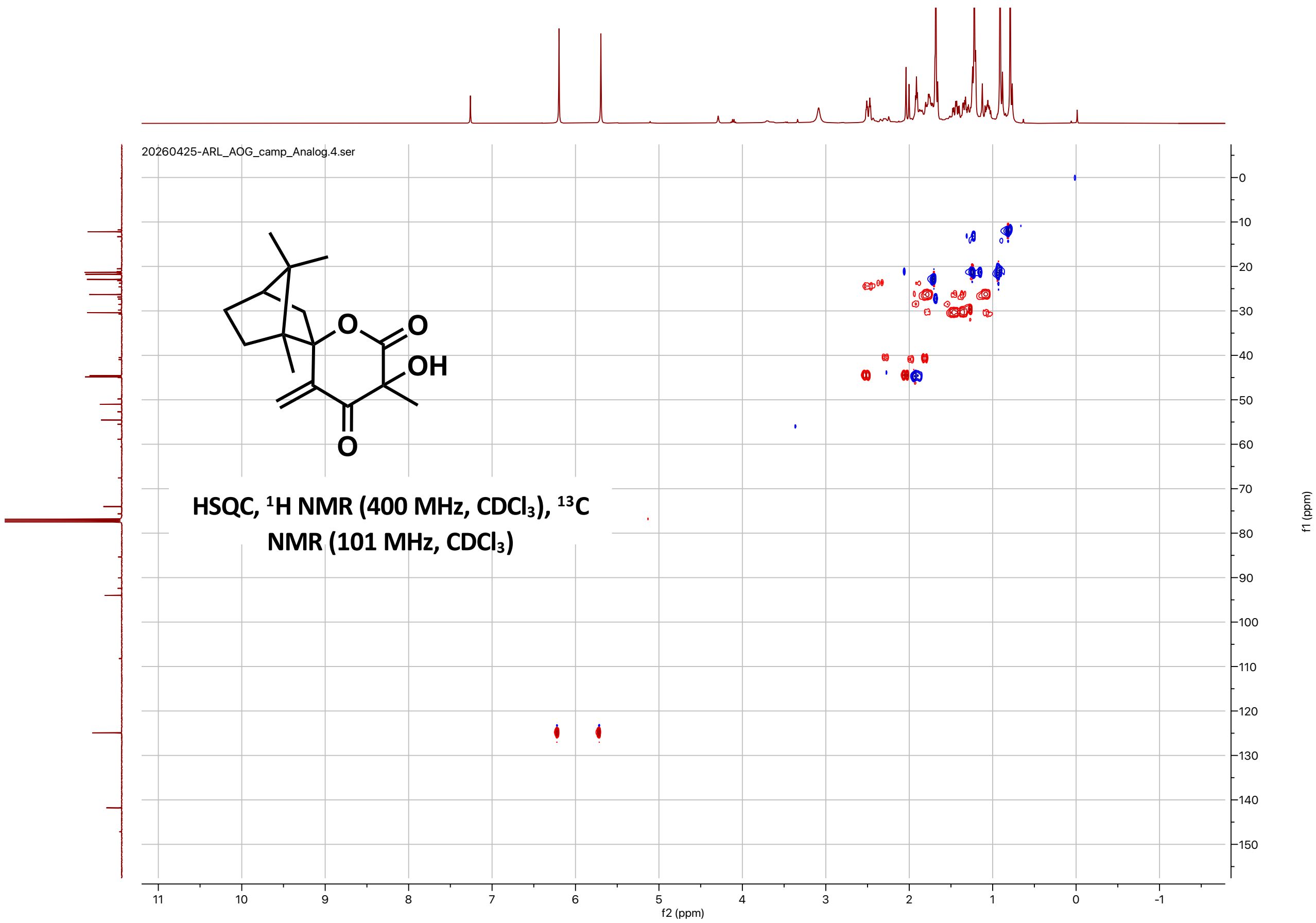
20260425-ARL_AOG_camp_Analog.4.ser
HSQC, ¹H NMR (400 MHz, CDCl₃), ¹³C NMR (101 MHz, CDCl₃)
O
O
OH
O
f2 (ppm)
f1 (ppm)

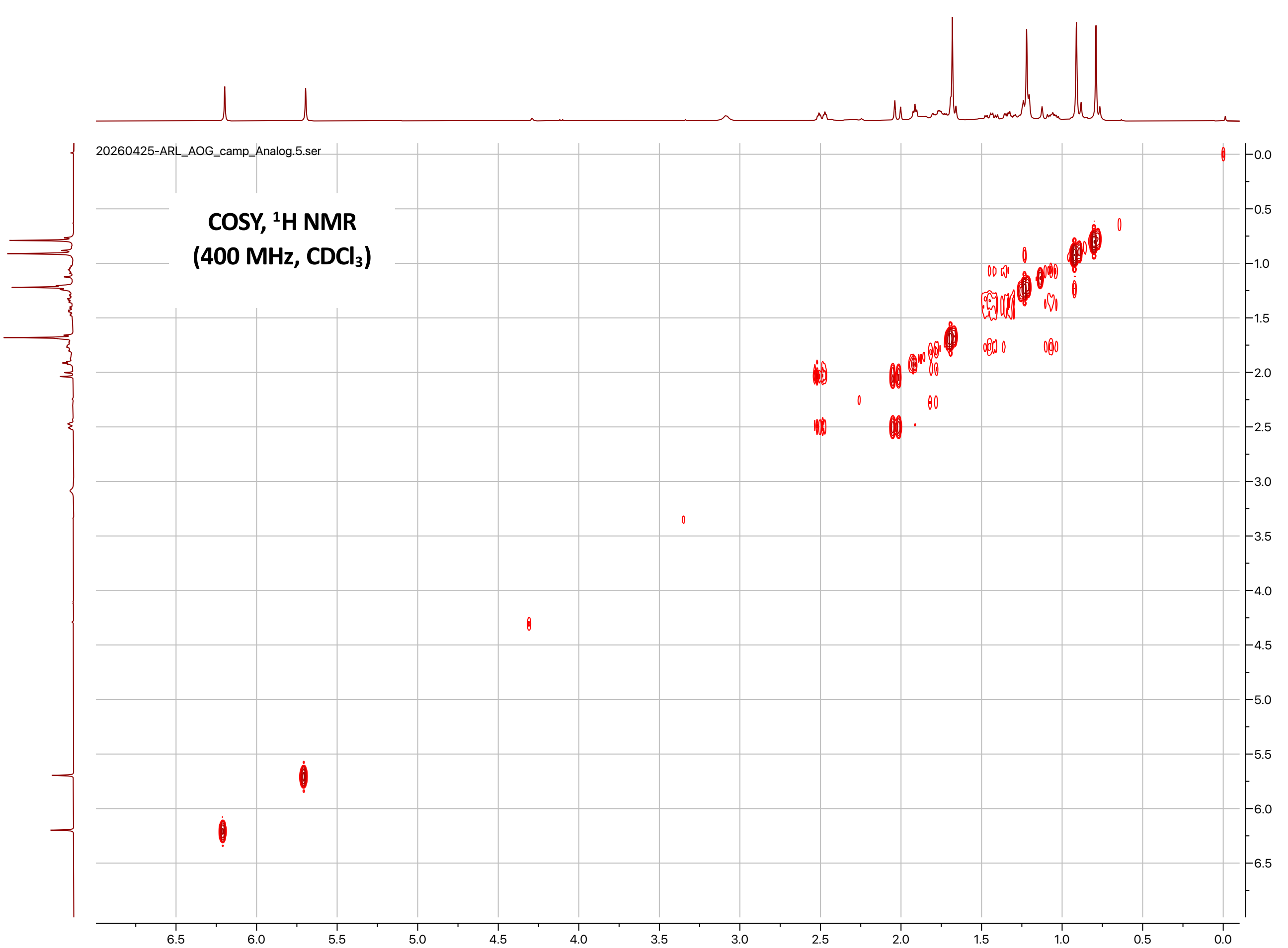
20260425-ARL_AOG_camp_Analog.5.ser
COSY, ¹H NMR (400 MHz, CDCl₃)
f2 (ppm)

20260329-ARL_AOG_435_F2.10.fid

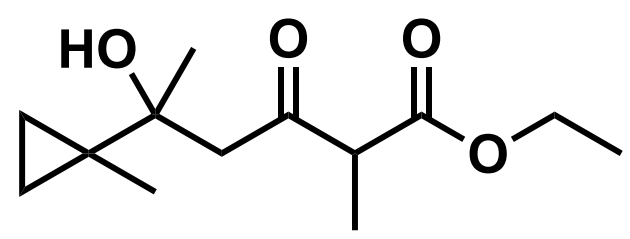

^{1}H NMR (400 MHz, $CDCl_3$),

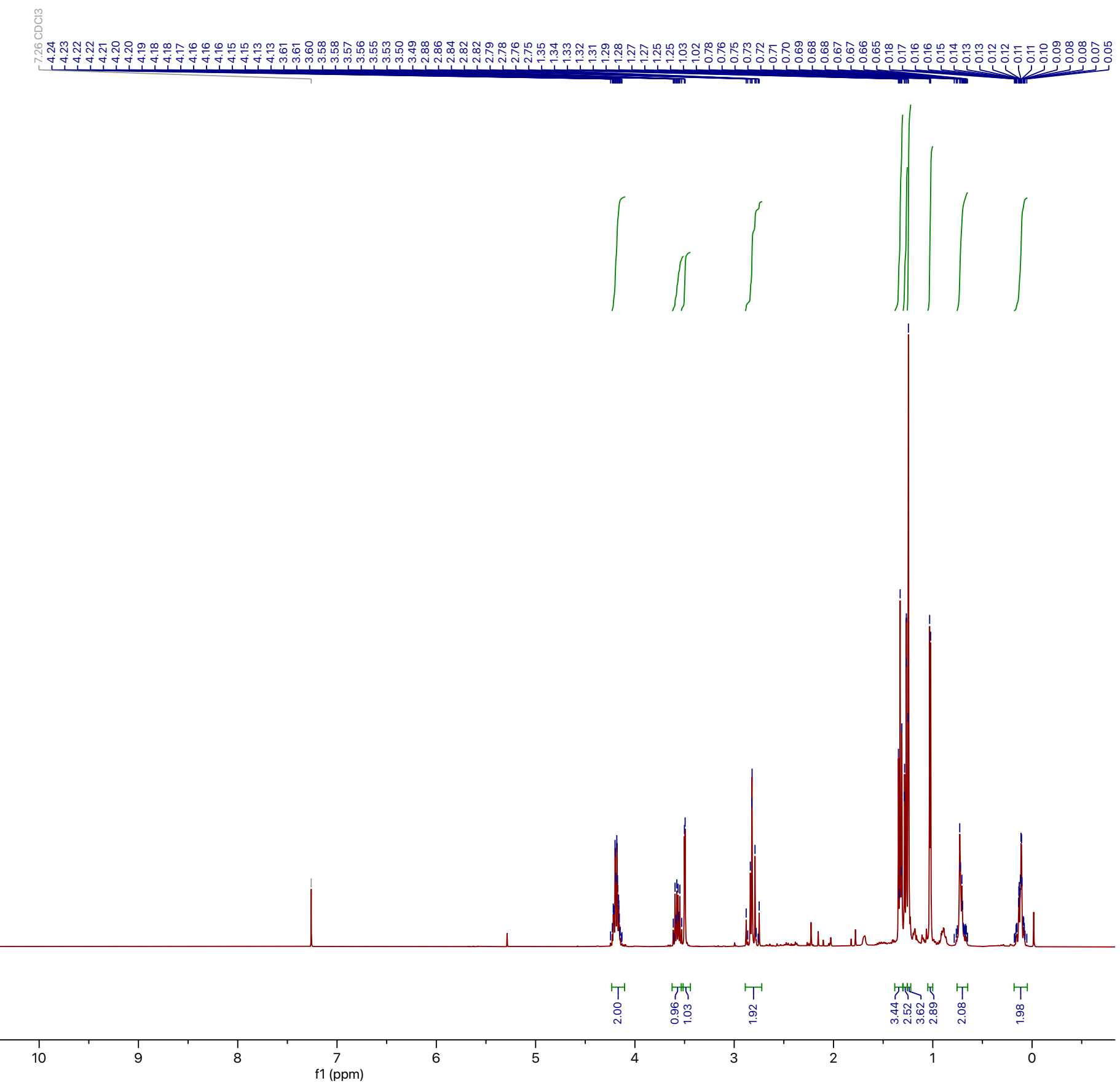

20260329-ARL_AOG_435_F2.11.fid

^{13}C NMR (101 MHz, $CDCl_3$)

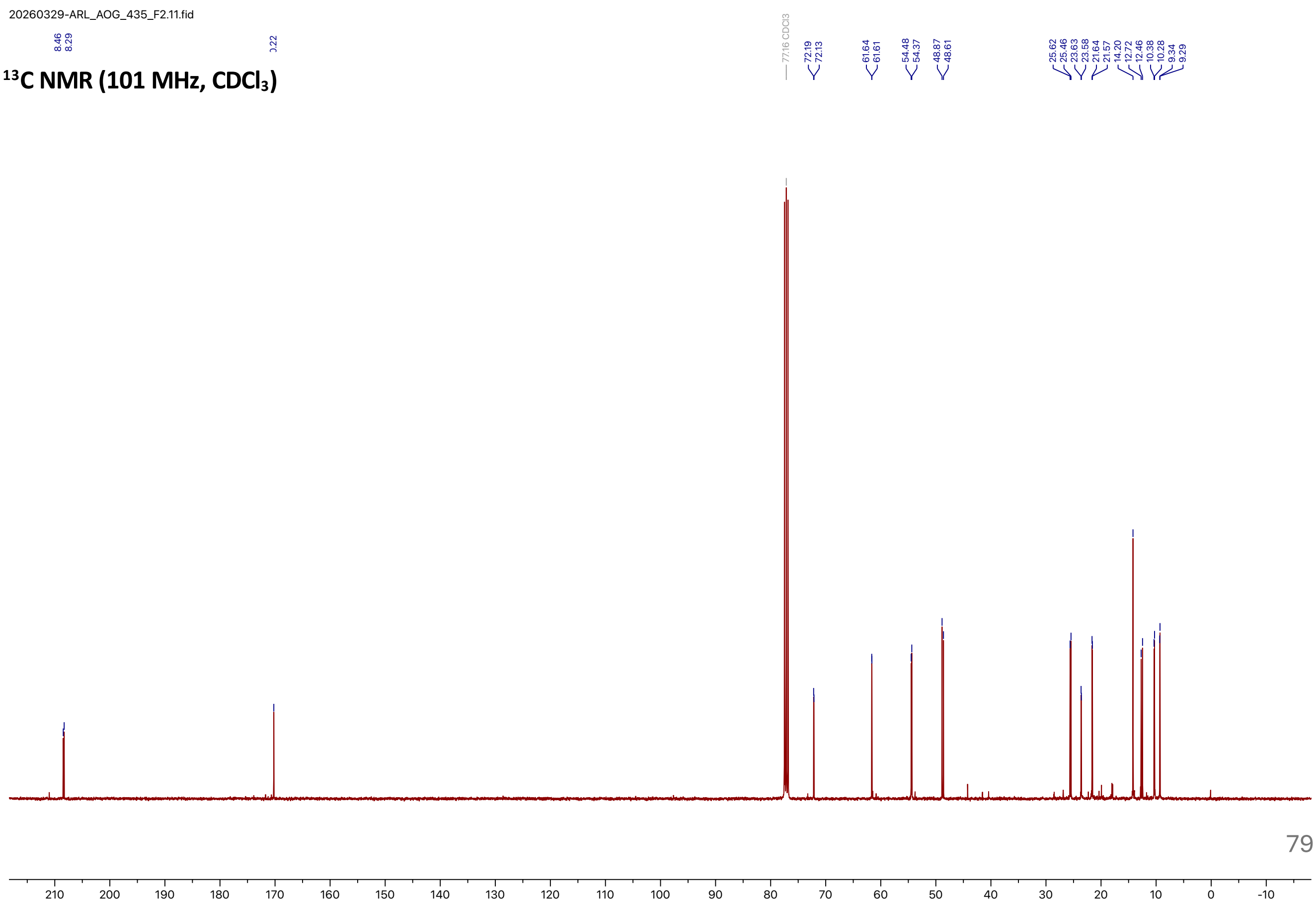

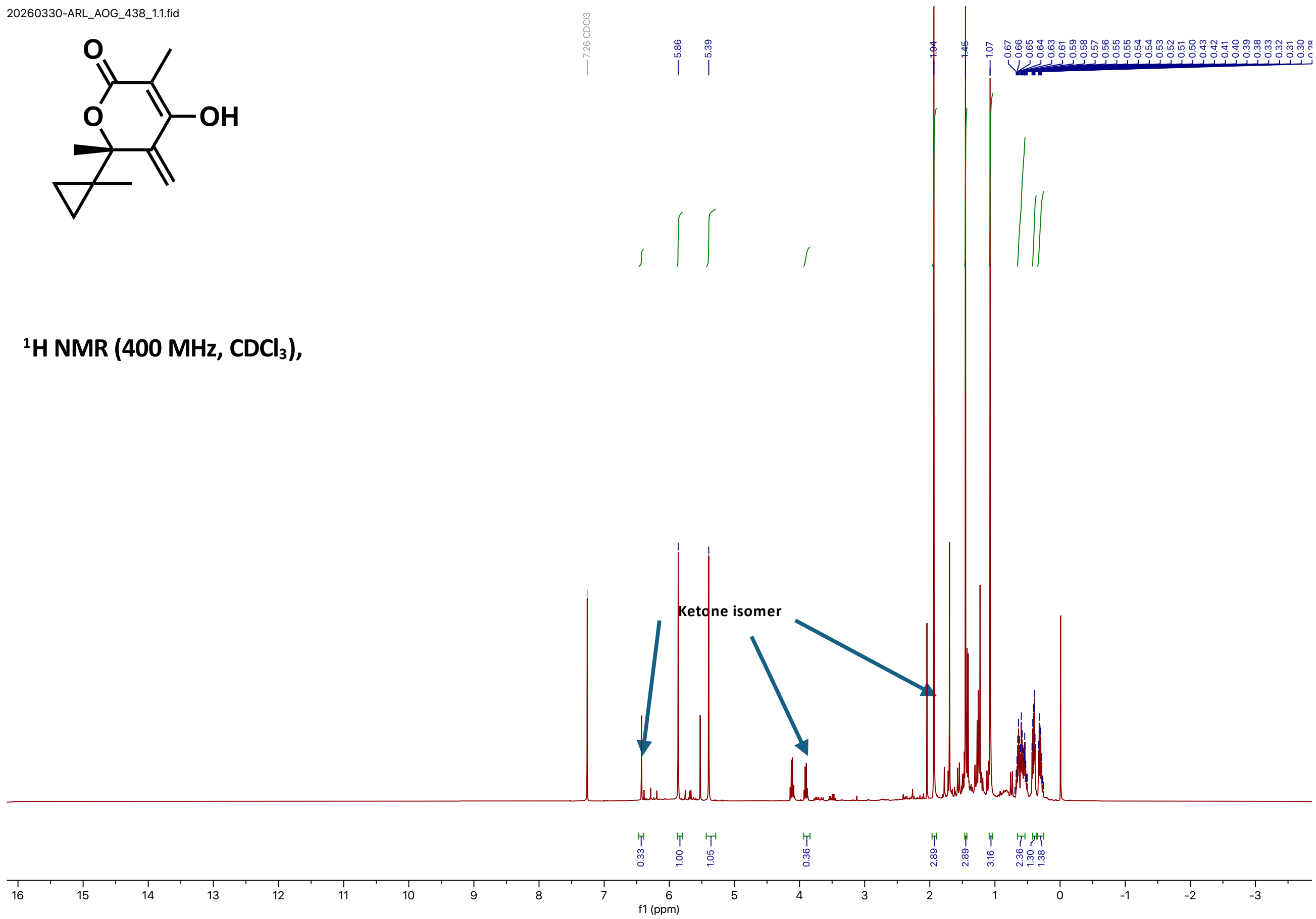

^{1}H NMR (400 MHz, $CDCl_3$),

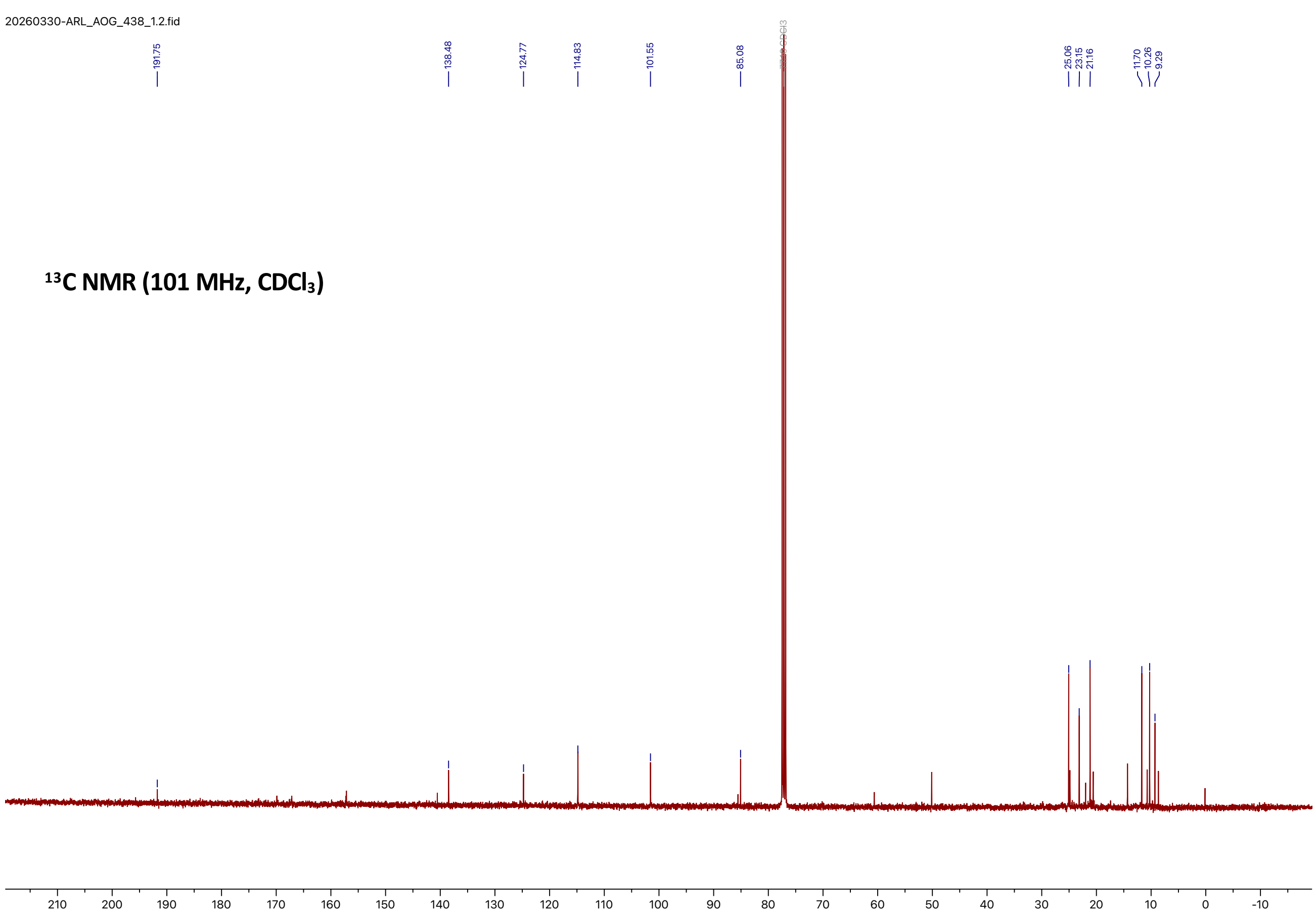

^{13}C NMR (101 MHz, $CDCl_3$)

20260407-ARL_AOG_440_50.1.fid

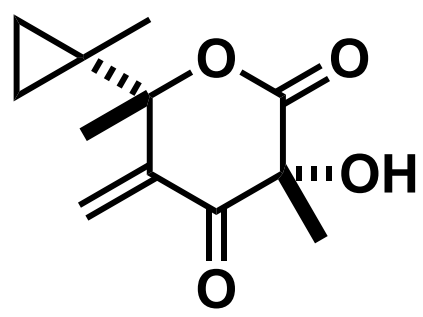

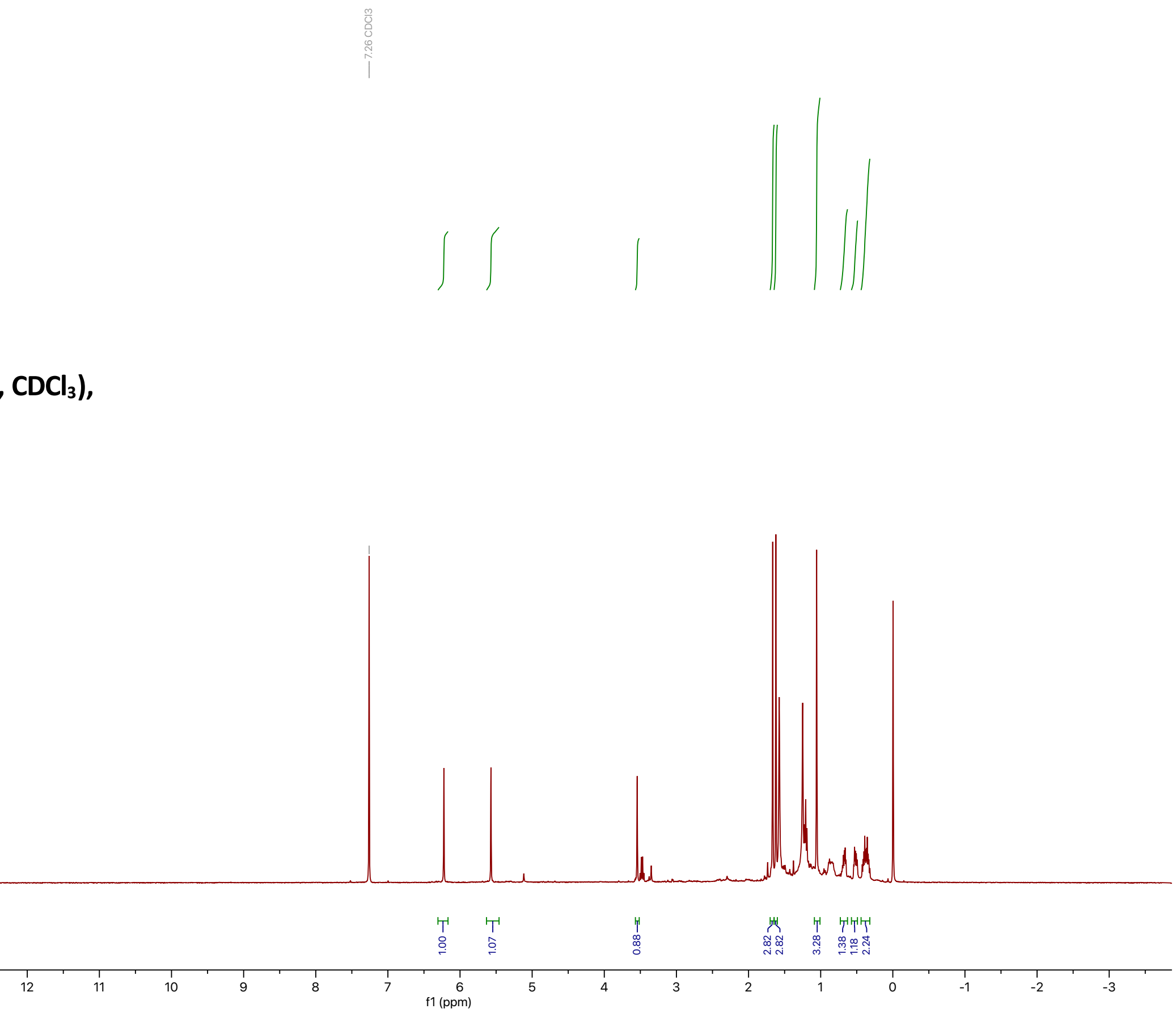

^{1}H NMR (400 MHz, $CDCl_3$),

20260407-ARL_AOG_440_50.2.fid

^{13}C NMR (101 MHz, $CDCl_3$)

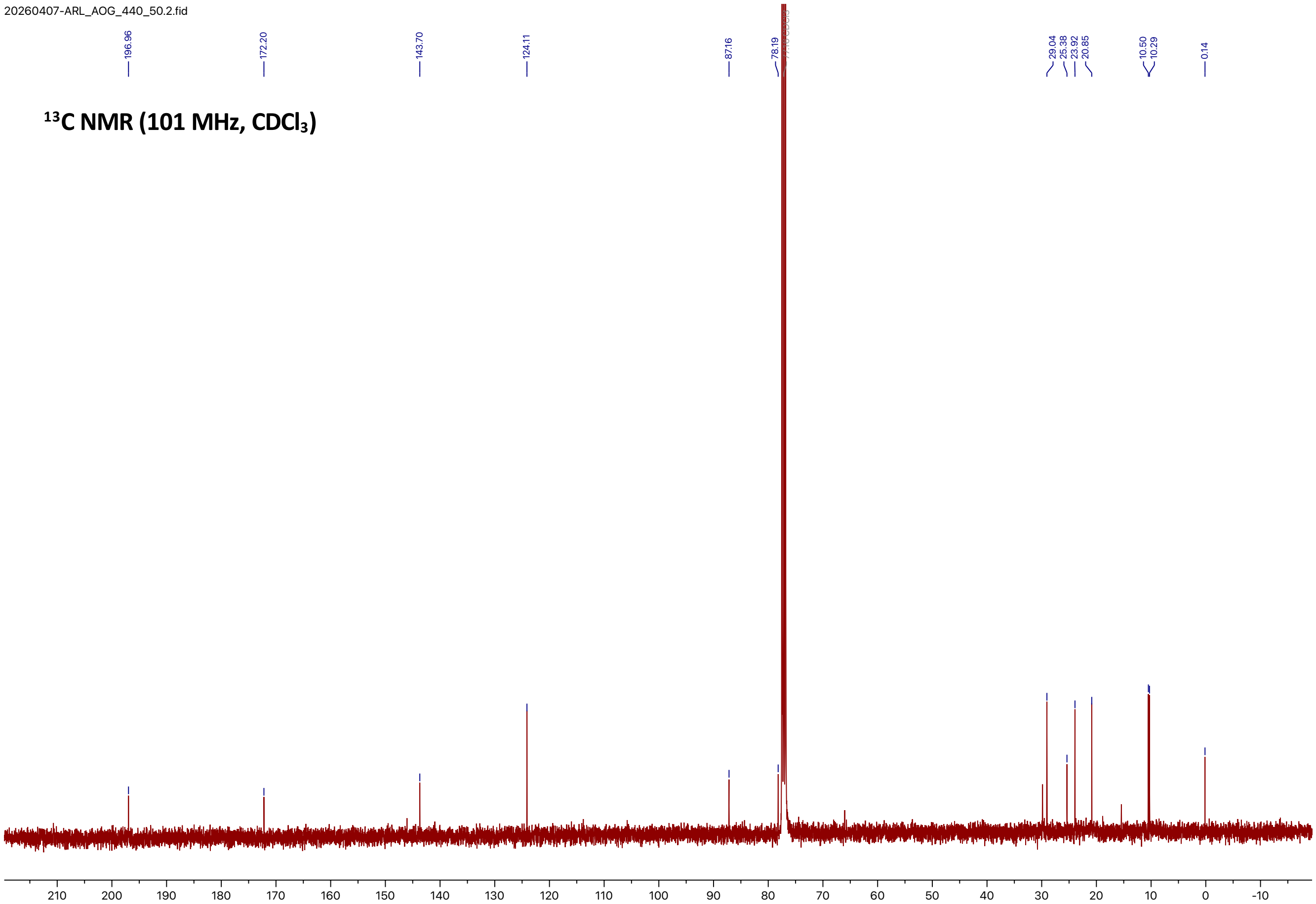

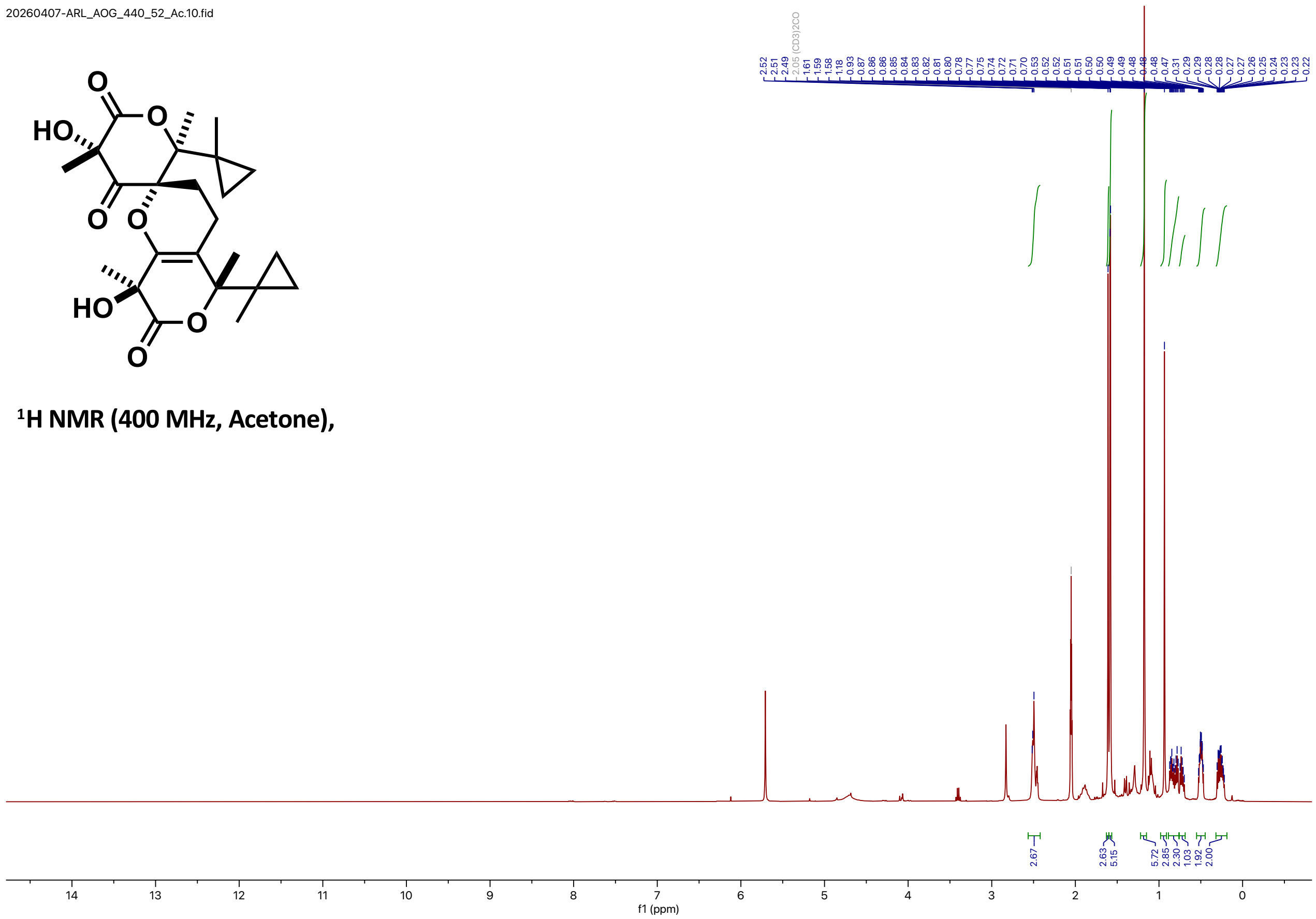
20260407-ARL_AOG_440_52_Ac.10.fid
HO
O
O
O
O
HO
O
O
O
^{1}H NMR (400 MHz, Acetone),
2.52 2.51 2.49 2.05 (CD3)2CO 1.61 1.59 1.58 1.18 0.93 0.87 0.86 0.86 0.85 0.84 0.83 0.82 0.81 0.80 0.78 0.77 0.75 0.74 0.72 0.71 0.70 0.53 0.52 0.52 0.51 0.51 0.50 0.50 0.49 0.49 0.48 0.48 0.47 0.31 0.29 0.29 0.28 0.28 0.27 0.27 0.26 0.25 0.24 0.23 0.23 0.22
2.67 2.63 5.15 5.72 2.85 2.30 1.03 1.92 2.00
f1 (ppm)

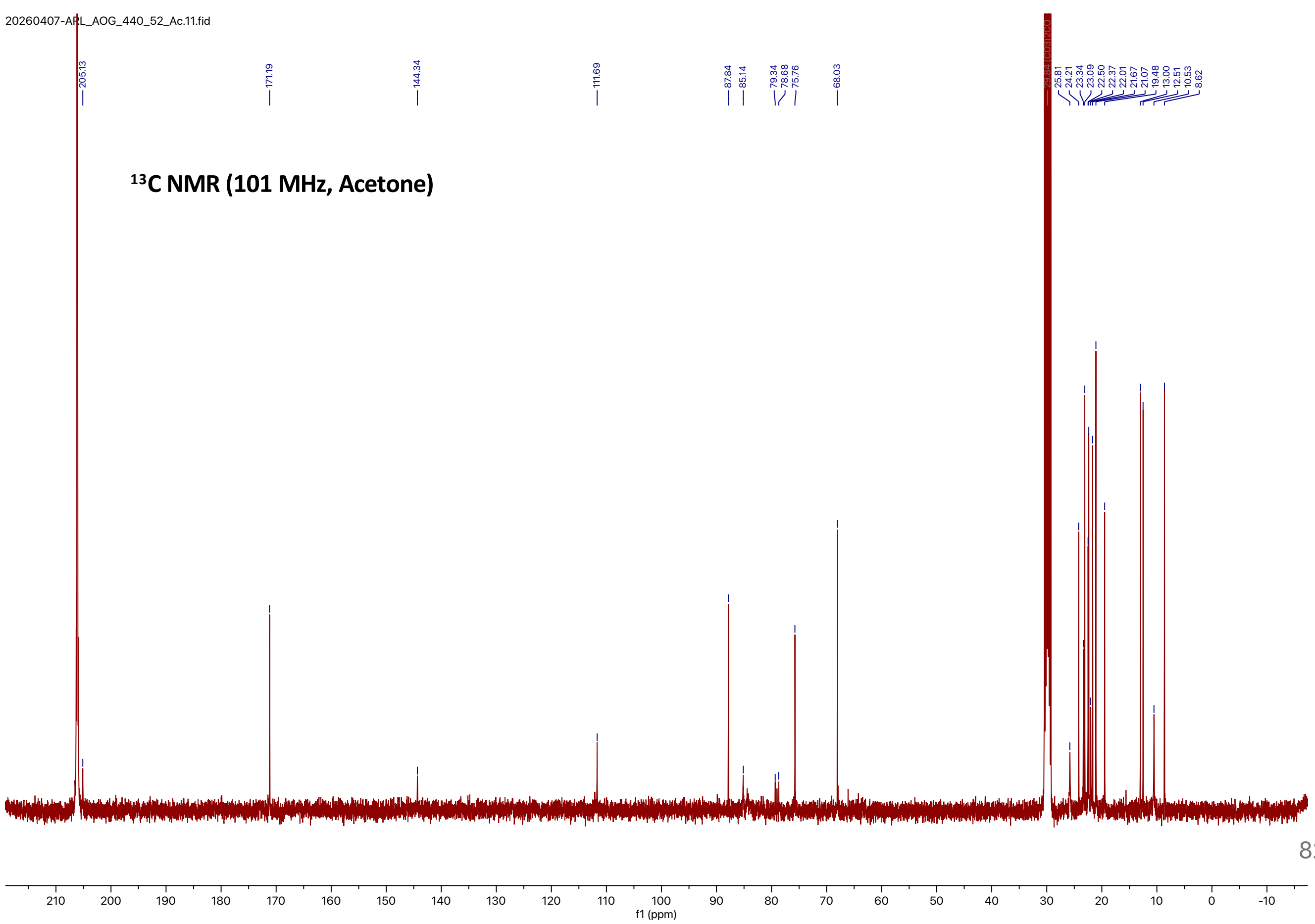
20260407-ARL_AOG_440_52_Ac.11.fid
^{13}C NMR (101 MHz, Acetone)
206.13 171.19 144.34 111.69 87.84 85.14 79.34 78.68 75.76 68.03 25.81 24.21 23.34 23.09 22.50 22.37 22.01 21.67 21.07 19.48 13.00 12.51 10.53 8.62
f1 (ppm)

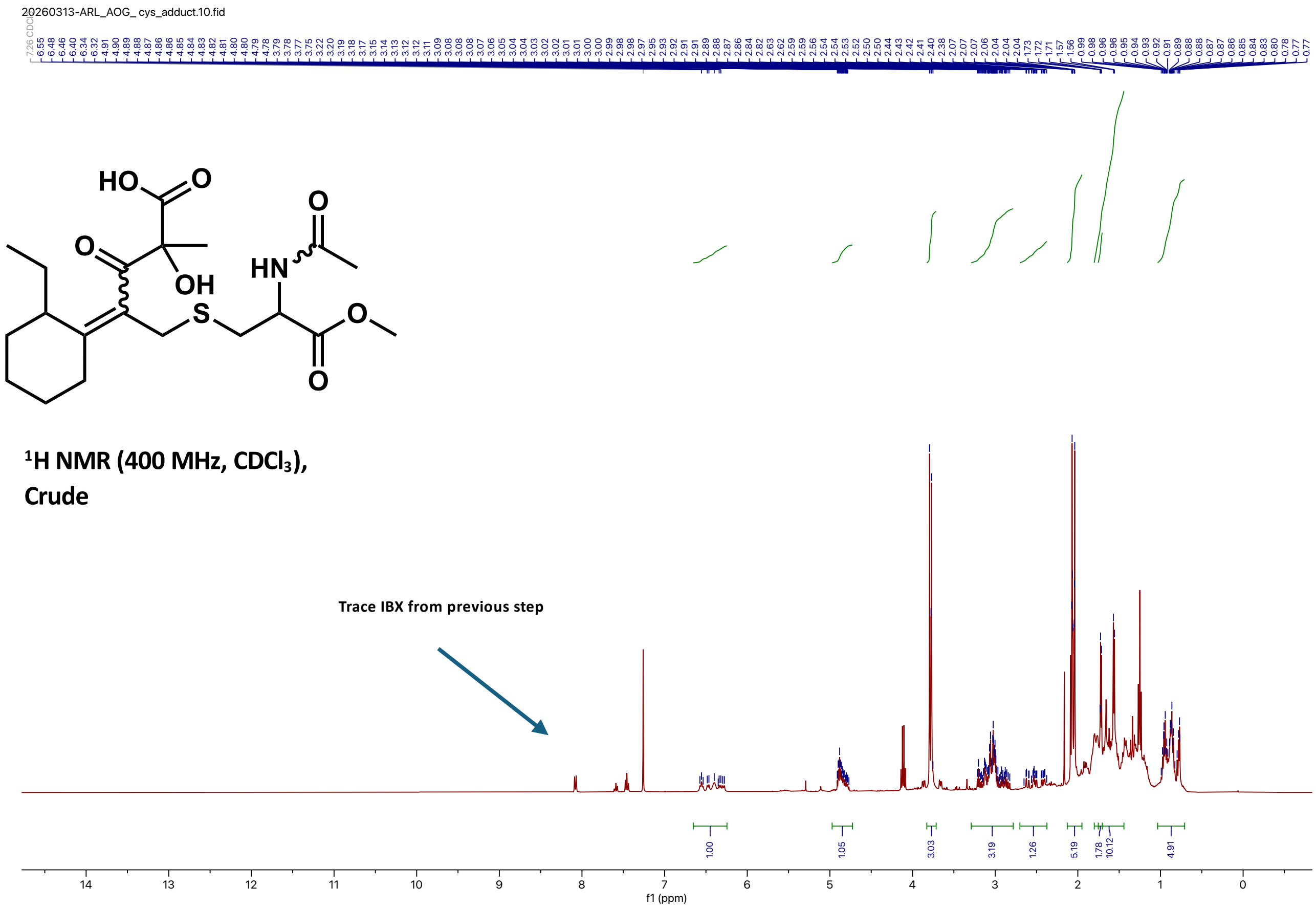

^{1}H NMR (400 MHz, $CDCl_3$), Crude

^{1}H NMR (400 MHz, $CDCl_3$),

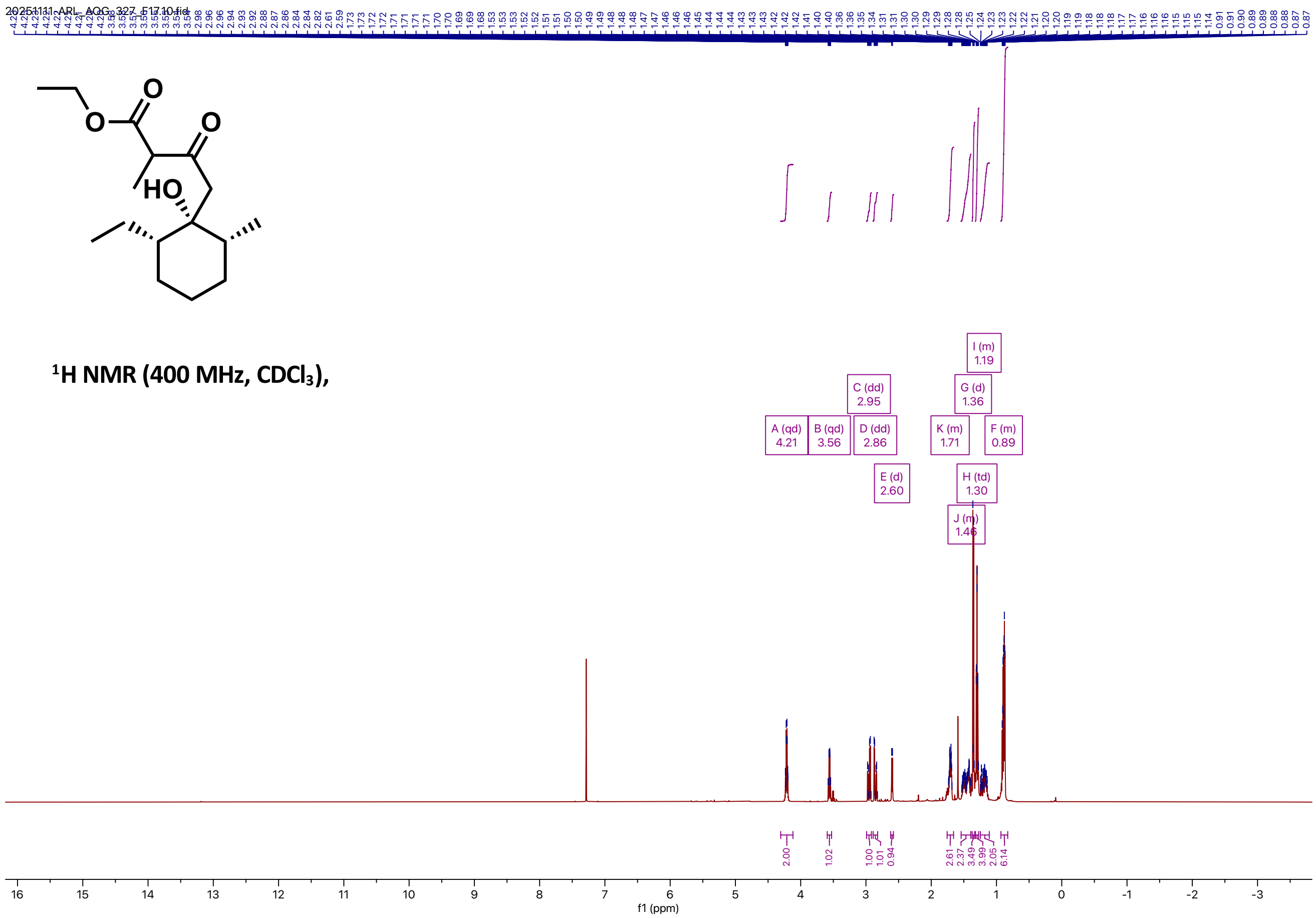

^{13}C NMR (101 MHz, $CDCl_3$)

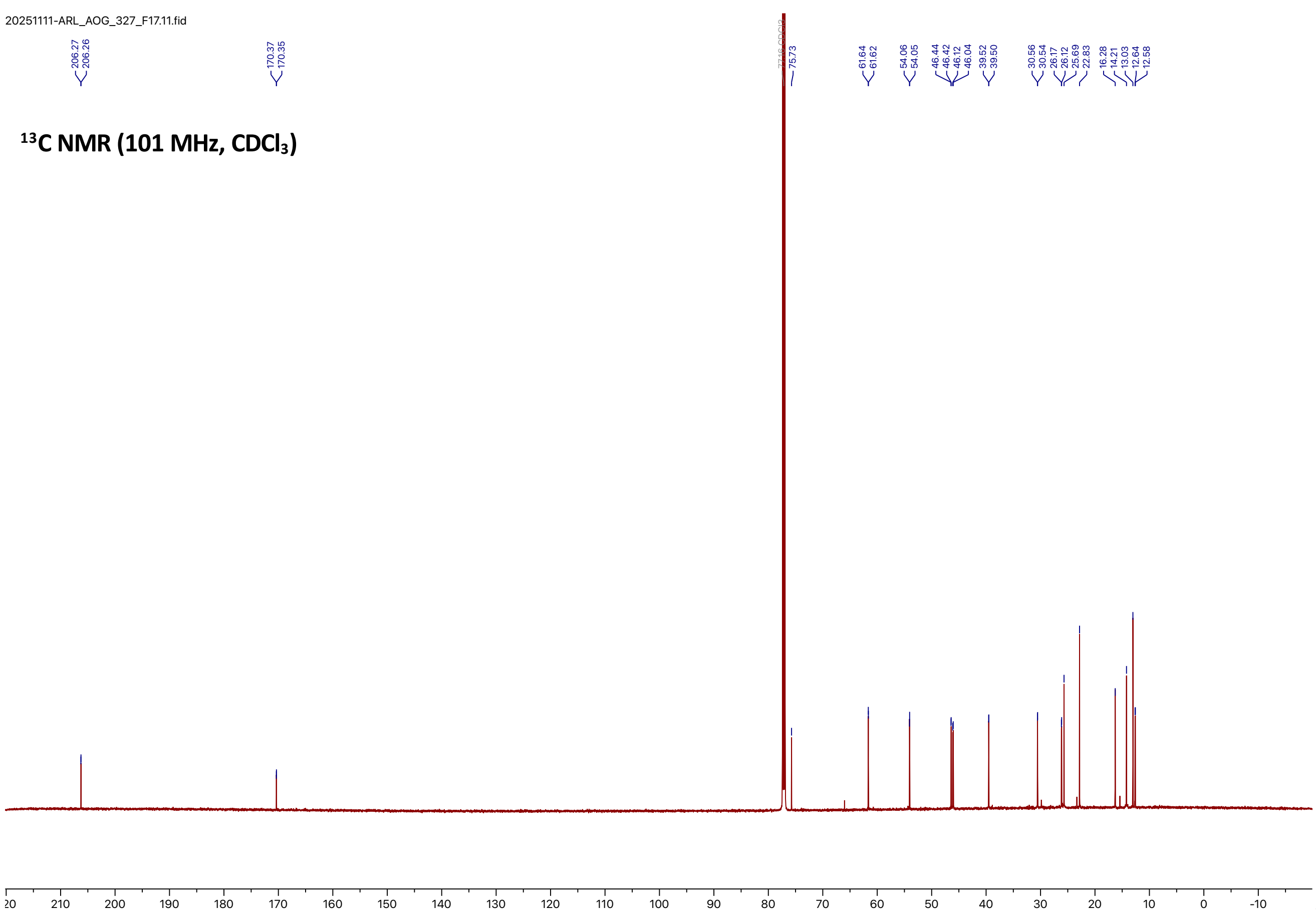

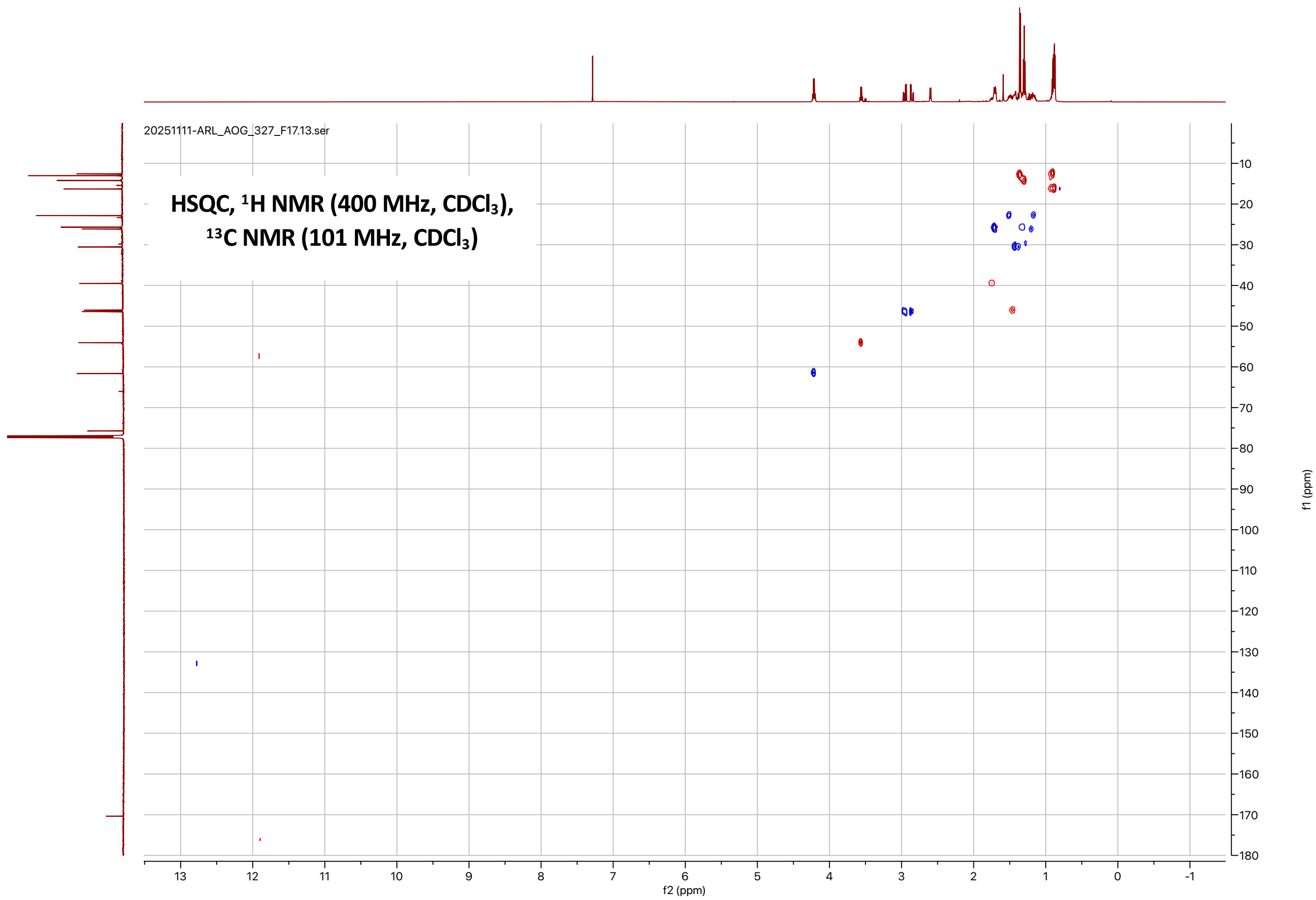
20251111-ARL_AOG_327_F17.13.ser
HSQC, ¹H NMR (400 MHz, CDCl₃),
¹³C NMR (101 MHz, CDCl₃)
f2 (ppm)
f1 (ppm)

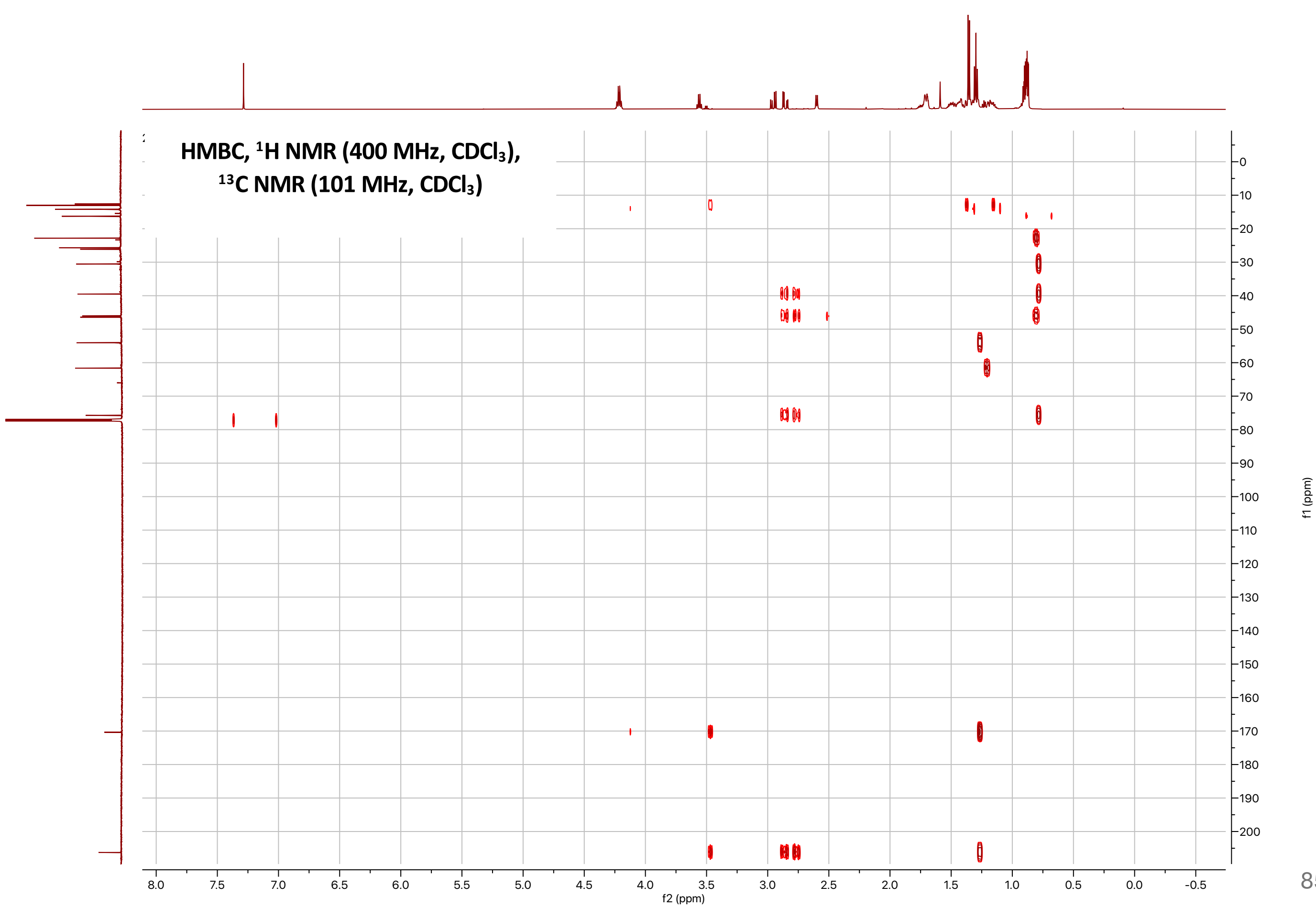
HMBC, ¹H NMR (400 MHz, CDCl₃),
¹³C NMR (101 MHz, CDCl₃)
f2 (ppm)
f1 (ppm)

Citations

(1) Takata, K.; Iwatsuki, M.; Yamamoto, T.; Shirahata, T.; Nonaka, K.; Masuma, R.; Hayakawa, Y.; Hanaki, H.; Kobayashi, Y.; Petersson, G. A.; Ōmura, S.; Shiomi, K. Aogacillins A and B Produced by *Simplicillium* Sp. FKI-5985: New Circumventors of Arbekacin Resistance in MRSA. *Org. Lett.* **2013**, *15* (18), 4678–4681. https://doi.org/10.1021/ol401975z.

(2) Gu, H.; Li, Z.; Zhang, X.; Sang, R.; Li, Z.; Ren, J.; Chen, X.; Ma, B.; Qiu, W.; Yang, Z.; Li, X.; Tong, R.; Zhang, W. Total Syntheses of *Ent* -Aogacillin A and Aogacillin B. *Chem. Sci.* **2026**, *17* (4), 2164–2168. https://doi.org/10.1039/D5SC04554A.

(3) Frolov, A. I.; Chuchvera, Y. O.; Ostapchuk, E. N.; Druzhenko, T. V.; Volochnyuk, D. M.; Ryabukhin, S. V. Toward a Chemical Constructor: A Lego-Like Approach for Formal α-Alkylation of Cyclic Ketones. *J. Org. Chem.* **2024**, *89* (11), 8208–8219. https://doi.org/10.1021/acs.joc.3c02628.

(4) Böttcher, T. An Additive Definition of Molecular Complexity. *J. Chem. Inf. Model.* **2016**, *56* (3), 462–470. https://doi.org/10.1021/acs.jcim.5b00723.

(5) Demoret, R. M.; Baker, M. A.; Ohtawa, M.; Chen, S.; Lam, C. C.; Khom, S.; Roberto, M.; Forli, S.; Houk, K. N.; Shenvi, R. A. Synthetic, Mechanistic, and Biological Interrogation of *Ginkgo Biloba* Chemical Space En Route to (−)-Bilobalide. *J. Am. Chem. Soc.* **2020**, *142* (43), 18599–18618. https://doi.org/10.1021/jacs.0c08231.

(6) Woo, S.; Shenvi, R. A. Synthesis and Target Annotation of the Alkaloid GB18. *Nature* **2022**, *606* (7916), 917–921. https://doi.org/10.1038/s41586-022-04840-9.
